# Structural Optimization and Biological Evaluation of Isoxazolo[5,4*-d*]pyrimidines as Selective Toll-Like Receptor 7 Agonists

**DOI:** 10.1021/acsomega.3c06343

**Published:** 2024-01-04

**Authors:** Nika Strašek Benedik, Ana Dolšak, Urban Švajger, Izidor Sosič, Stanislav Gobec, Matej Sova

**Affiliations:** † Faculty of Pharmacy, Department of Pharmaceutical Chemistry, University of Ljubljana, Aškerčeva 7, Ljubljana 1000, Slovenia; ‡ Blood Transfusion Centre of Slovenia, Šlajmerjeva 6, Ljubljana 1000, Slovenia

## Abstract

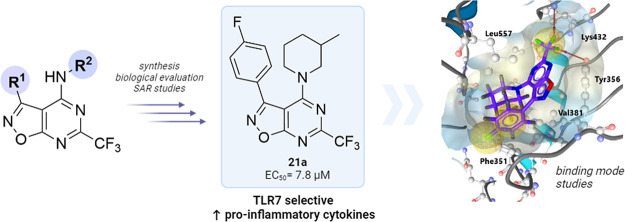

Toll-like receptors (TLRs) are components of innate immunity that play a crucial role in several diseases, including chronic inflammatory and infectious diseases, autoimmune diseases, and cancer. In particular, TLR7 has been identified as a key player in the innate immune response against viral infections and small-molecule TLR7 agonists have shown potential for vaccine therapy, for treatment of asthma and allergies, and as anticancer drugs. Inspired by our previous discovery of selective TLR7 agonists, our goal was to develop and introduce a new chemotype of TLR7 agonists by replacing the quinazoline ring with a new heterocycle isoxazolo[5,4*-d*]pyrimidine. Here, we report design, optimized synthesis, and structure–activity relationship studies of a novel class of TLR7 agonists based on the 6-(trifluoromethyl)isoxazolo[5,4*-d*]pyrimidine-4-amine scaffold that demonstrate high selectivity and low micromolar potencies. The best-in-class agonist **21a**, with an EC_50_ value of 7.8 μM, also proved to be noncytotoxic and induced secretion of cytokines, including IL-1β, IL-12p70, IL-8, and TNF-α, indicating its potential to modulate the immune response.

## Introduction

Toll-like receptors (TLRs) are members of innate immunity receptors that recognize and bind damage-associated patterns (DAMPs) and pathogen-associated patterns (PAMPs).^1^ In humans, there are 10 different types of TLRs; TLR 1, 2, 5, 6, and 10 are located on the cell membrane, whereas TLR 3, 7, 8, and 9 are found on the membranes of endosomes. TLR 4 is found on both the cell membrane and the membrane of endosomes.^2^ The location of TLRs within the cell is related to the types of ligands they recognize and bind. TLRs expressed on the cell membrane recognize external microorganisms, whereas TLR 3 and TLR 7–9 mainly recognize viral nucleic acids.^3,4^ TLR 7 plays an important role in the innate immune response by recognizing short GU-rich and AU-rich single-stranded RNA sequences (ssRNA) from RNA viruses. Upon binding of an endogenous ligand or a small synthetic nucleoside analog, TLR 7 dimerizes and subsequent conformational changes trigger the myeloid differentiation primary response protein 88 (MyD88) signaling cascade. This leads to the release and translocation of activated NF-κB or AP-1 to the nucleus, where the expression of pro-inflammatory cytokines and type I interferons is induced.^5−7^ It was shown previously that upregulated or downregulated TLR7 signaling is involved in the development and progression of numerous diseases, including chronic inflammatory and infectious diseases, autoimmune diseases, and cancer.^8−10^ However, the use of TLR7 agonists in the treatment of these diseases is still in the research phase, so further clinical studies are needed to fully understand their role and efficacy.

**Figure 1:**
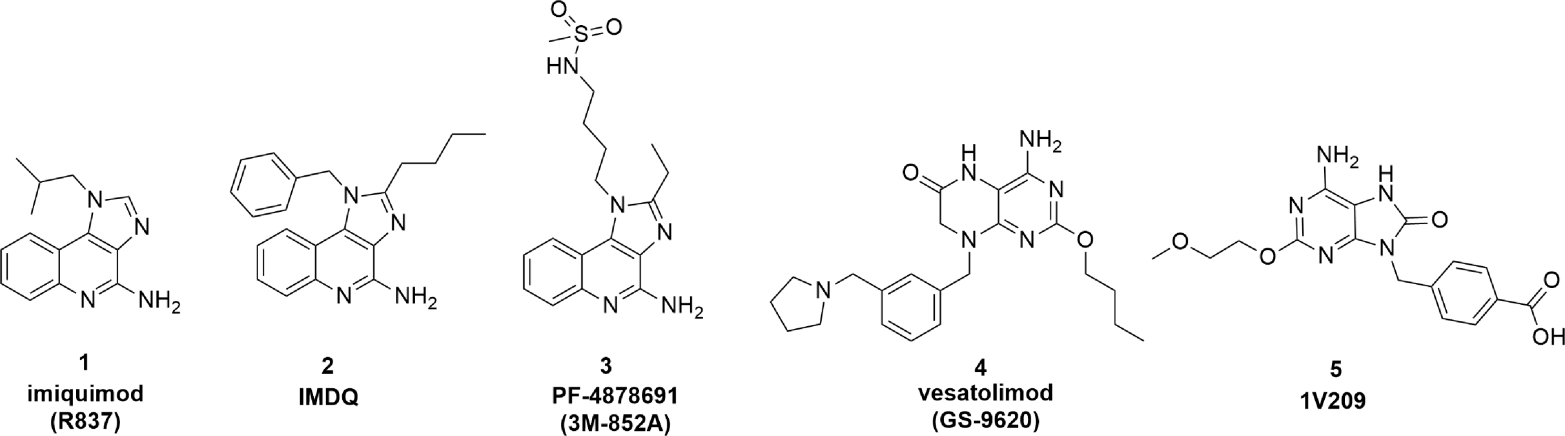
Representative structures of small-molecule TLR7 agonists.

Low molecular weight TLR7 agonists were extensively studied for their potential as anticancer drugs.^11,12^ Despite considerable efforts in this area, only one small-molecule TLR7 agonist, imiquimod (**1**),^13,14^ has successfully reached the market thus far. Imiquimod has been prescribed for the topical treatment of genital warts, superficial basal cell carcinoma, and actinic keratosis.^15^ A major challenge in the development of TLR7 agonists is in the limited availability of novel chemotypes, with most of the research focused on imidazoquinolines (representative compounds **1**–**3**, Figure 1),^16,17^ which suffer from poor pharmacokinetic properties and toxic side effects after systemic administration.^18,19^ Other chemotypes as small-molecule TLR7 agonists were thoroughly described in some recent review articles.^19−22^ These include analogs of guanosine, adenine (**5**),^23^ pteridinone (**4**),^24^ pyrido[3,2-*d*]pyrimidine,^25^ pyrrolopyrimidine, and benzonaphthyridines (Figure 1). However, despite their high affinity, many of the small molecules developed face challenges in clinical development due to lack of efficacy and negative effects on the host immune response. Overall, the search for small-molecule TLR7 agonists with improved pharmacokinetic properties, reduced side effects, and increased therapeutic efficacy remains a significant challenge in this field.^18,19^

Inspired by selective TLR7 agonists based on 2-(trifluoromethyl)quinolin-4-amine and 2-(trifluoromethyl)quinazolin-4-amine scaffolds discovered in our group in 2019,^26^ our aim in the present study was to introduce structural modification of the main quinazoline ring to obtain a novel chemotype of potent TLR7 agonists. Herein, we report the synthesis, structure–activity relationship study, and biological evaluation of 6-(trifluoromethyl)isoxazolo[5,4*-d*]pyrimidine-4-amines as novel selective TLR7 agonists with low micromolar potencies.

## Results and Discussion

### Design and Synthesis

**Figure 2:**
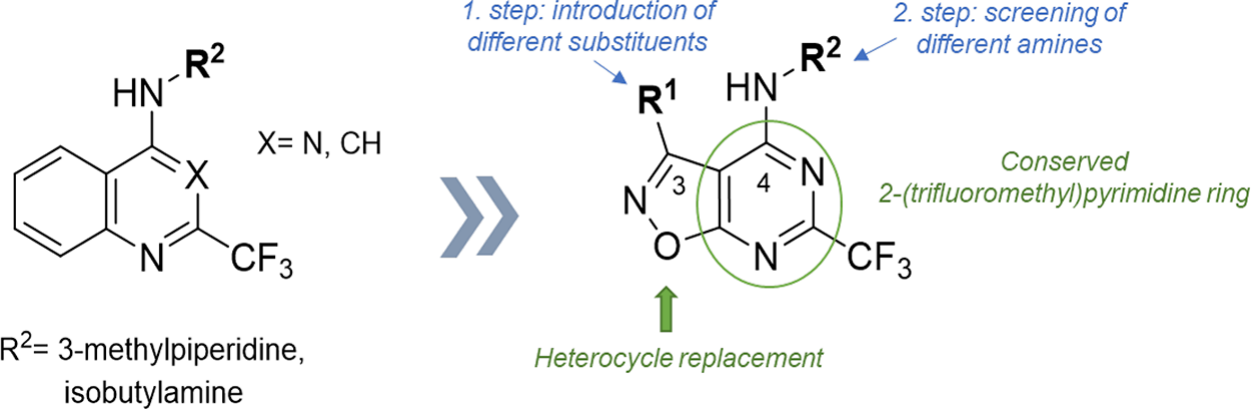
Design of novel TLR7 agonists.

The idea for developing a new chemotype of TLR7 agonists came from previously published TLR7 agonists.^26^ These compounds were based on 2-(trifluoromethyl)quinolin-4-amine and 2-(trifluoromethyl)quinazolin-4-amine scaffolds; however, the majority of active compounds were derived from the latter (Figure 2). Since the quinazoline scaffold was found to be more favorable compared to the quinoline scaffold in terms of affinity, and the CF_3_ group formed favorable interactions in the active site in our previous study, the 2-(trifluoromethyl)quinazolin-4-amine scaffold was chosen as the starting point. Initially, we decided to focus on the left side of the scaffold and explore the chemical space around it, since the right part was already investigated in our previous study.^26^ We decided to keep the 4-amino-2-(trifluoromethyl)pyrimidine part of a quinazoline ring to retain the activity while replacing the benzene ring fused to the pyrimidine nucleus with a heterocycle. The isoxazole ring was selected as a heterocyclic replacement for benzene due to synthetic accessibility and the possibility of introducing additional substituents at the C3 position (Figure 2, R^1^),^27^ and potential additional interactions within the active site due to the presence of additional heteroatoms. Second, we decided to synthesize derivatives with different R^2^ substituents to find the most optimal substitution at the C4 position of our new scaffold.

**Scheme 1:**
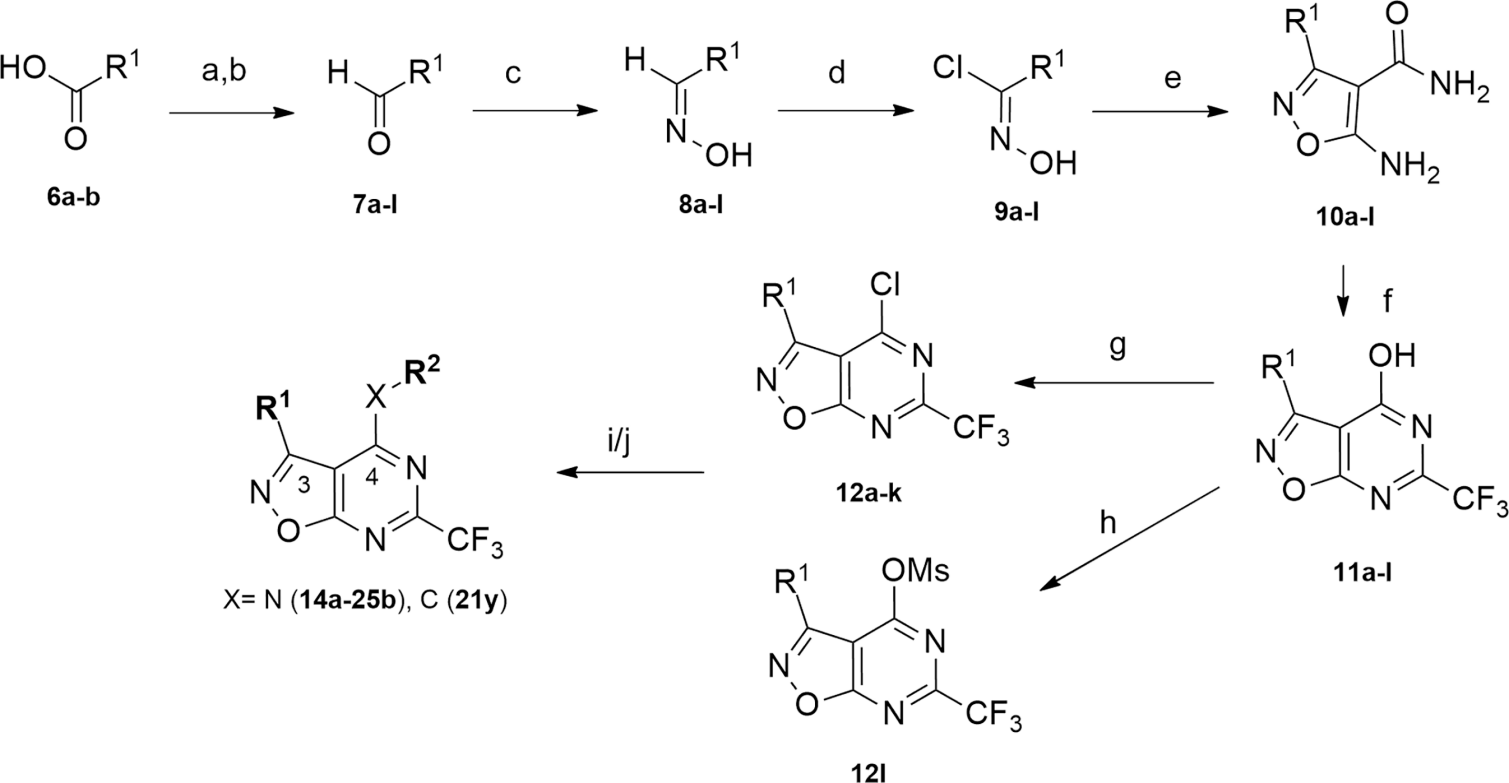
Synthesis of Isoxazolo[5,4*-d*]pyrimidines Reagents and conditions: (a) TBTU, Et_3_N, CH_2_Cl_2_, *N*,*O-*dimethylhydroxylamine × HCl, 1 h at 0 °C, and then 18 h at RT; (b) LiAlH_4_, THF, 3 h at 0 °C; (c) NH_2_OH × HCl, THF/EtOH/H_2_O, RT, 0.5 h; (d) NCS, DMF, 0 °C to RT, 16 h; (e) 2-cyanoacetamide, NaOEt/EtOH, 1 h at 0 °C, 18 h at reflux; (f) ethyl trifluoroacetate, NaOEt/EtOH, 24 h at reflux; (g) POCl_3_, 16 h at 120 °C; (h) DMAP, DIPEA, MsCl, CH_2_Cl_2_, 0 °C to RT, 16 h; (i) amine R^2^, K_2_CO_3_, MeCN, 1 h at 50 °C, and then 24 h at RT; (j) 1-bromo-3-methylcyclohexane, Mg, 1,2*-*dibromoethane, THF, 2 h at 70 °C, and then amine and K_2_CO_3_, 16 h at 70 °C.

The six-step synthetic procedure was used to prepare a series of 45 6-(trifluoromethyl)isoxazolo[5,4*-d*]pyrimidines with different substituents at positions R^1^ and R^2^, as shown in Scheme 1. In the first step, we started from the commercially available acids **6a**,**b** or the corresponding aldehydes **7a**–**l**. The acids were converted to aldehydes via formation of a Weinreb amide and subsequent reduction with LiAlH_4_. Next, the aldehydes were reacted with hydroxylamine to obtain suitable oximes **8a**–**l**, which were then converted to *N*-hydroxyimidoyl chlorides **9a**–**l** with *N*-chlorosuccinimide. The next step of the synthesis was cyclization to the isoxazole ring with 2-cyanoacetamide and freshly prepared sodium ethanolate to obtain 5-aminoisoxazole-4-carboxamides **10a**–**l**, which were used as starting reagents in the second cyclization with ethyl trifluoroacetate and sodium ethanolate. Isoxazolo[5,4*-d*]pyrimidin-4-ols **11a**–**l** were further converted to chloro derivatives **12a**–**k** or methanesulfonate **12l**, in case of final compounds **25a**,**b**. For **25a**,**b**, a different synthetic approach was required in the fifth step because it contained Boc-protected piperidine as the R^1^ substituent–Boc-protecting group would potentially be removed by POCl_3_ treatment due to acidic reaction conditions, which could lead to the formation of a dimeric side product. Therefore, we used mesyl chloride in the reaction with **11l** to form a good leaving group. The final step of the synthesis was the nucleophilic aromatic substitution of **12a**–**l** with the corresponding amines to obtain final compounds **14a**–**25b**. Initially, we focused only on two different amines—3-methylpiperidine and isobutylamine—because these substituents exhibited the lowest EC_50_ values when present on the 2-(trifluoromethyl)quinazoline scaffold from our previous study.^26^ Later, we also introduced other amines at the C4 position with a 4-fluorophenyl group at the C3 of the 2-(trifluoromethyl)isoxazolo[5,4*-d*]pyrimidine scaffold. The synthesis of compound **21y** required a Grignard reaction to form a C–C bond. The Grignard reagent was prepared from 1-bromo-3-methylcyclohexane, Mg, and 1,2*-*dibromoethane as the initiator of the reaction. The freshly synthesized Grignard reagent was then reacted with 4-chloroisoxazolo[5,4*-d*]pyrimidine to give compound **21y**. The general synthesis was optimized to the extent that purification by column chromatography was generally required only in the last two steps of the synthesis.

### Biological Evaluation

All 45 compounds were screened for their agonist activity on the HEK293 cell line cotransfected with the hTLR7 gene and an inducible SEAP reporter gene. The first compounds synthesized and tested were **14a** and **14b** with the phenyl ring as R^1^ and 3-methylpiperidine and isobutylamine as R^2^, both being inactive on TLR7 (Table 1). The next pair of compounds possessed 4-chlorophenyl as R^1^. Compound **15a** with the 3-methylpiperidine substituent as R^2^ showed promising agonist activity with an EC_50_ value of 21.4 μM, while in the case of isobutylamine derivative **15b**, agonist activity was lost. The introduction of a halogen atom on the phenyl ring improved activity in comparison to the inactive **14a** with the phenyl substituent.

**Table 1:**
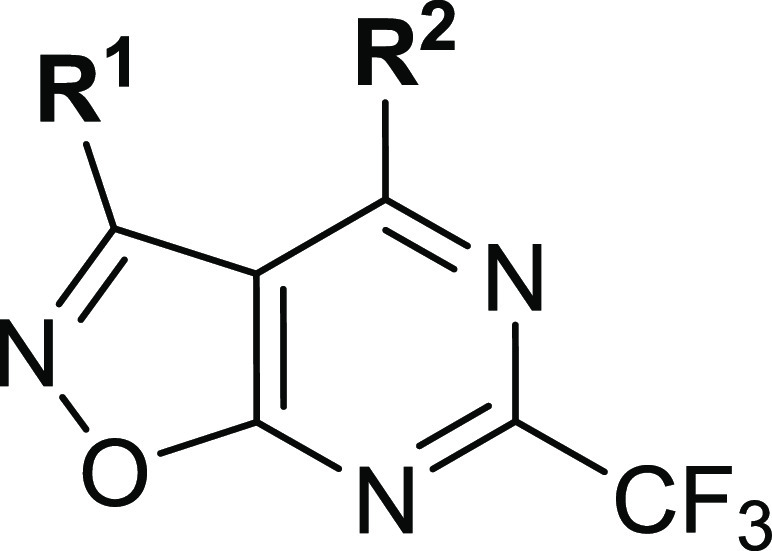

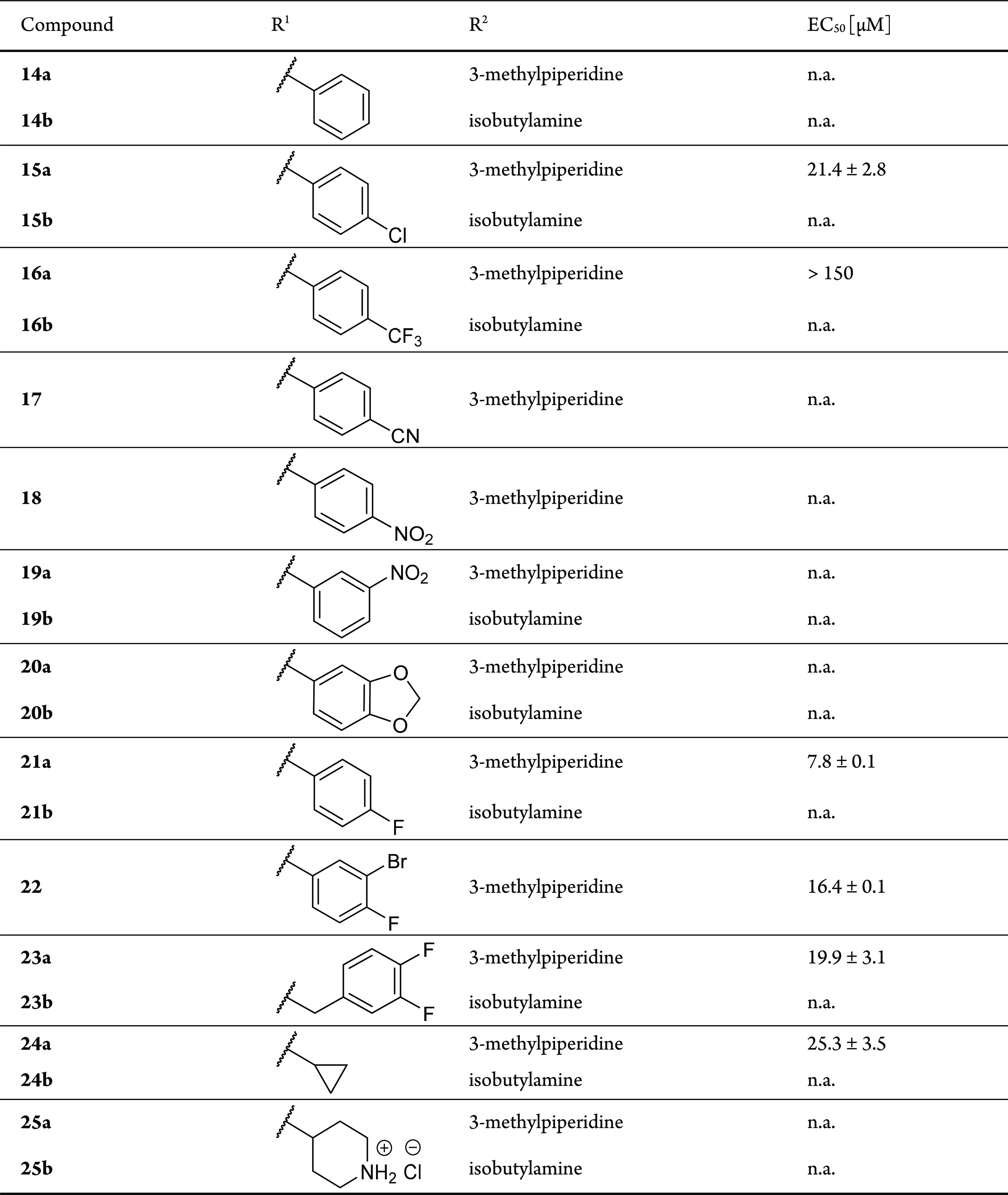
EC_50_ Values of the First Set of Compounds^a^

a n.a.—not active.

Next, we tried switching the chlorine atom with electron-withdrawing substituents such as trifluoromethyl, cyano, and nitro groups (**16a**–**18**), but all compounds lacked activity. Furthermore, changing the position of a nitro group did not offer any change in activity as seen for both amine derivatives **19a**,**b**. The same result was obtained when testing the compound with a fused benzo[*d*][1,3]dioxole ring on a phenyl substituent. Replacement of the chlorine atom with flourine resulted in compound **21a**, which showed improved potency with an EC_50_ of 7.8 μM. However, there has been no activity observed on TLR7 in the case of isobutyl derivative **21b**, even though it possessed 4-fluorophenyl as R^1^ substituent. Compounds with an additional bromine atom on the R^1^ phenyl ring (**22**) or methyl linker between the main scaffold and R^1^ difluorophenyl ring (**23a**) retained the activity (EC_50_ of 16.4 and 19.9 μM, respectively), with no improvement of the potency. Finally, we wanted to investigate whether the aromatic ring at position C4 was critical for activity; therefore, we replaced it with two aliphatic rings, i.e., cyclopropane and piperidine. The lipophilic cyclopropyl derivative **24a** with the 3-methylpiperidine substituent at C4 did show agonist activity on TLR7 with an EC_50_ value of 25.3 μM. Among the first series of 6-(trifluoromethyl)isoxazolo[5,4*-d*]pyrimidines, all compounds with an isobutylamine substituent at the C4 were either inactive or cytotoxic while some derivatives with 3-methylpiperidine ring showed potent TLR7 agonist activity with EC_50_ values between 7.8 and 25.3 μM.

After exploring the chemical space around the position C3 of the isoxazole scaffold, we focused on position C4 to investigate the impact of different amines on potency. First, cyclopropylamine was introduced, as it gave promising results in our previous study;^26^ however, this time it led to an inactive compound **21c** (Table 2). Since the 3-methylpiperidine substituent appeared to be essential for activity, we wanted to investigate the role of a methyl substituent on piperidine ring by removing it or changing its position (compounds **21d**, **21e**, and **21f**). The methyl group removal led to an inactive derivative, whereas changed positions led to nearly 10-fold loss of activity (EC_50_s for **21e** and **21f** were 63.3 and 68.0 μM, respectively). Next, the replacement of 3-methylpiperidine with 3-methylmorpholine retained the potency (**21g**) while more polar derivatives with a piperazine ring were either cytotoxic (**21i**) or lacked activity (**21h**, **21j**). Similarly, loss of activity was observed when changing the methyl group with trifluoromethyl, ester, carboxylic acid, or amine. Furthermore, we decided to increase the rigidity of the 3-methylpiperidine substituent and therefore introduced 3,3*-*dimethylpiperidine (**21o**) and 5-azaspiro[2.5]octane (**21p**), which were slightly less potent TLR7 agonists than a parent compound. Approximately 10-fold loss in potency was also observed, when the 3-methyl was replaced with the 3-isopropyl substituent in compound **21q**. Finally, compounds with a smaller aliphatic ring, such as a pyrrolidine, suffered from a significant loss of activity. To investigate whether a nitrogen in a cyclic amine is essential for activity, we synthesized compound **21y**, where the C–N bond was replaced by a C–C bond. Compound **21y** retained its activity, suggesting that the tertiary amine is not important for formation of interactions with the amino acid residues within the active site.

**Table 2:**
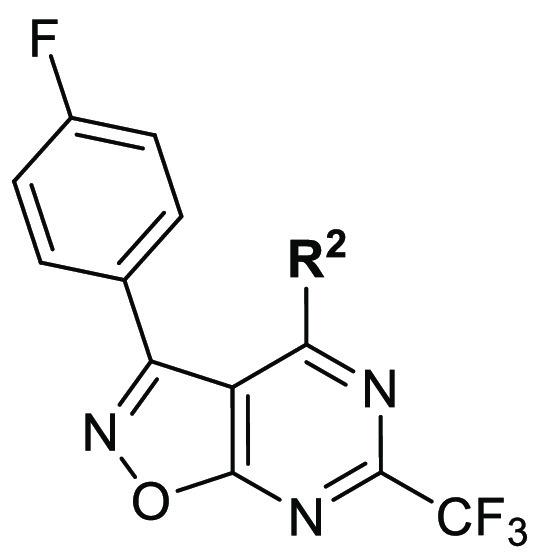

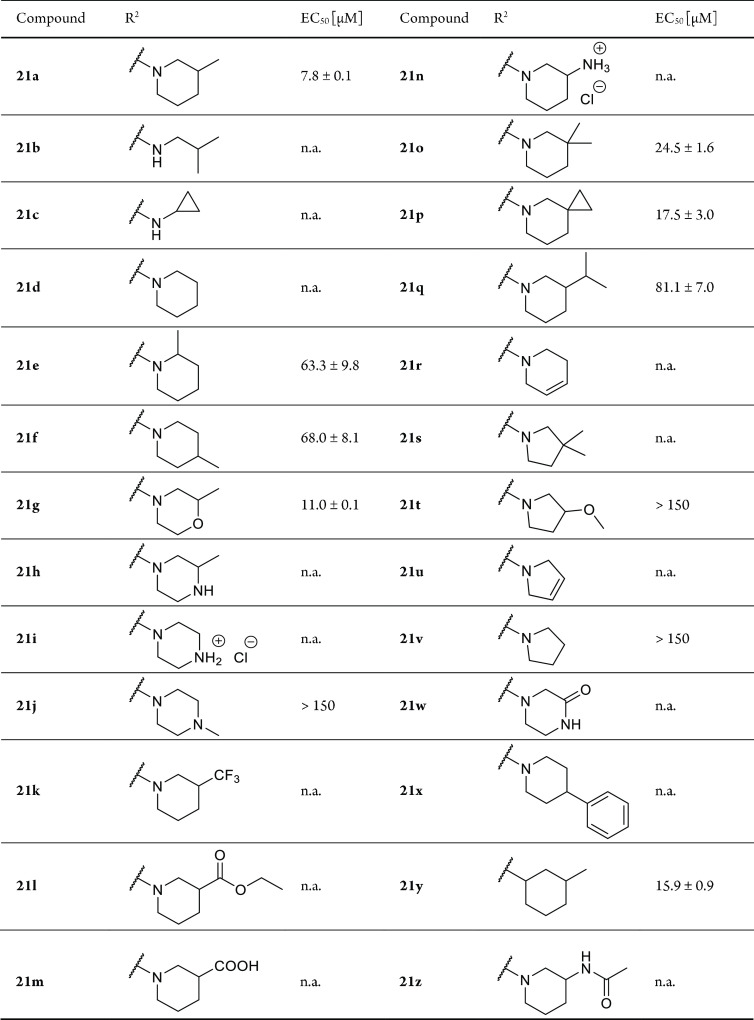
EC_50_ Values of the Second Set of Compounds^a^

a n.a.—not active.

However, compounds **21e**, **21f**, **21g**, and **21y** showed a low plateau response (less than twofold increase in maximum response), indicating that although some of them showed promising EC_50_ values, the efficacy was low. This suggests that they are not suitable for further investigation. To determine selectivity toward TLR8, all active compounds were also evaluated for their agonist activity on the HEK293 cell line cotransfected with the hTLR8 gene and an inducible SEAP reporter gene. None of them exhibited any activity in the TLR8 assay. Therefore, the 6-(trifluoromethyl)isoxazolo[5,4*-d*]pyrimidine derivatives represent selective TLR7 agonists with a novel chemical scaffold.

### Cytotoxicity

**Figure 3:**
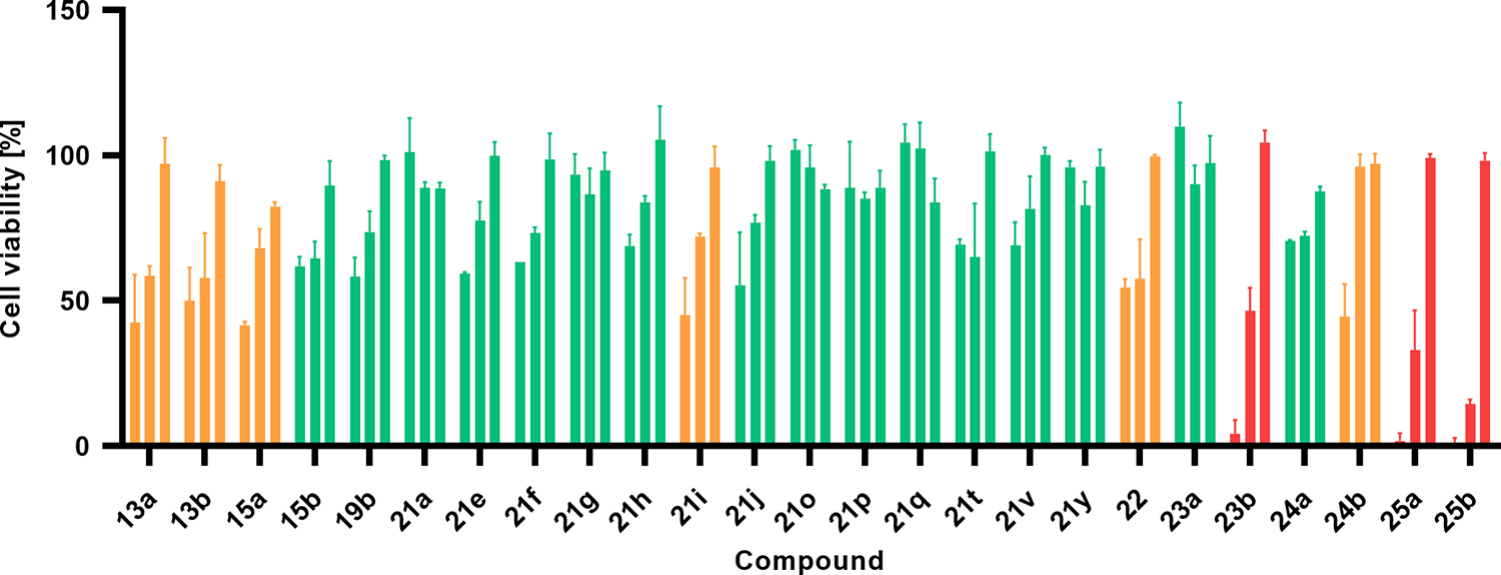
Graph shows the percentage of viability of the HEK293 cell line cotransfected with the hTLR7 gene after treatment with 150, 50, and 10 μM of compounds (concentrations in the graph are shown from highest to lowest; for the negative control (NC), cells were treated with DMSO). Highly cytotoxic compounds are depicted in red, moderately cytotoxic (<50% cell viability at the highest concentration) in orange, and noncytotoxic in green.

The cytotoxicity of the compounds was determined by the MTS assay at concentrations of 150, 50, and 10 μM on the HEK293 cell line cotransfected with the hTLR7 gene (Figure 3). Cells were treated for 24 h with compounds that showed either agonist activity on TLR7 or had visible impact on cell growth under microscope at concentrations higher than 150 μM (see Figure S92 in the Supporting Information). As shown in Figure 3, compounds containing free amines, such as **25a**, **25b** with a piperidine moiety at R^1^, and **21i** with piperazine at R^2^, are highly cytotoxic at 150 μM. Compounds with the isobutyl moiety at R^2^ also proved to be moderately to highly cytotoxic. However, compounds that showed an agonistic effect on TLR7 were found to be noncytotoxic at lower concentrations or only slightly cytotoxic at higher concentrations to the HEK293 cell line cotransfected with the hTLR7 gene.

### Cytokine Secretion

**Figure 4:**
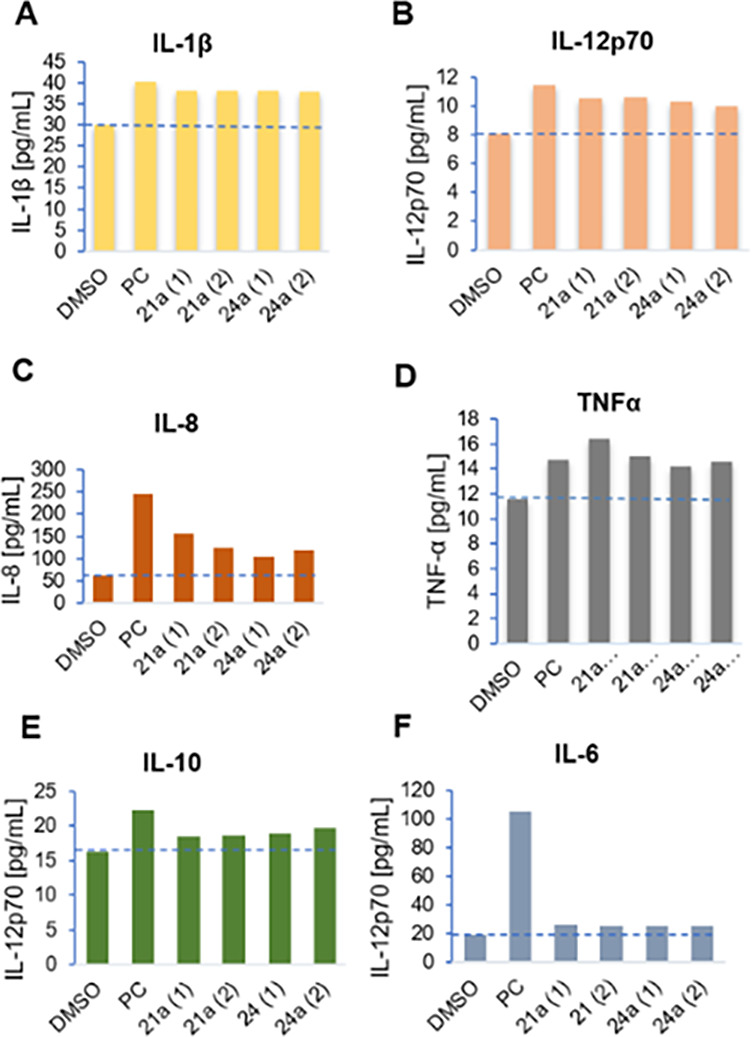
Measurement of cytokine secretion in PBMCs after treatment with **21a**, **24a**, and imiquimod (PC).

To determine if compounds induce cytokine secretion in human peripheral blood mononuclear cells (PBMCs), we selected two representative compounds (**21a** as the most potent one from 4-fluorophenyl series and **24a** as the active compound with cyclopropyl substitution on the isoxazole ring). PBMCs were seeded on a 24-well plate and treated with compounds **21a** (62.5 μM (**21a** (1)) and 12.5 μM (**21a** (2))) and **24a** (125 μM (**24a** (1)) and 25 μM (**24a** (2))). For a positive control (PC), imiquimod (**1**) was used (final concentration is 10 μg/mL) and the negative control were cells treated with DMSO. As shown in Figure 4, compounds **21a** and **24a** induced the secretion of cytokines IL-1β, IL-12p70, IL-8, and TNF-α while the concentrations of IL-6 and IL-10 did not increase significantly. Similar to **1**, both **21a** and **24a** stimulated the release of IL-1β, a pivotal pro-inflammatory cytokine that activates macrophages, monocytes, and neutrophils, and is also involved in adaptive cellular immune responses.^28,29^

Similarly, comparable results were observed for the production of IL-12p70, a cytokine known for its stimulating properties of natural killer cells and T cells. IL-12p70 induces production of interferon-γ in both cell types and is one of the main cytokines that support establishment of type 1 adaptive immune responses via differentiation of Th1 and cytotoxic CD8^+^ T cells.^30−32^ Additionally, compound **21a** demonstrated higher induction of TNF-α secretion at 62.5 μM in comparison to **1**. TNF-α is considered one of the major acute phase pro-inflammatory cytokines. Its production is known as a key step to inducing local inflammation and can subsequently contribute to initiation of adaptive immune responses as well.^33^ Finally, the secretion of IL-8 was also observed; however, the levels were at least twofold lower compared to **1**.

### Binding Mode of Compound **21a** in the TLR7 Active Site

**Figure 5:**
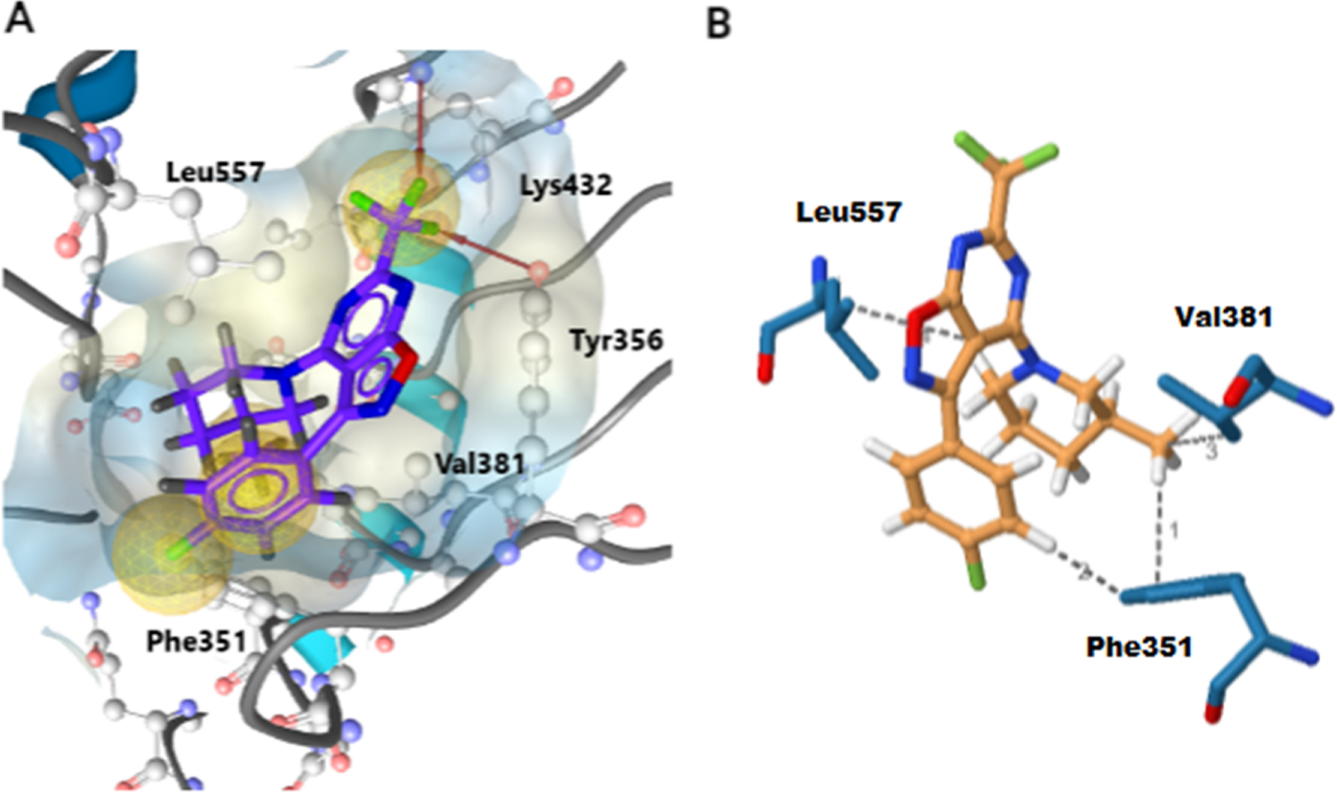
(A) Docking of **21a** in the active site of TLR7 (pdb code 6LVX). Yellow spheres represent hydrophobic interactions, and H-bonds are marked with red arrows. (B) Hydrophobic interactions predicted with the protein–ligand interaction profiler (PLIP) between compound **21a** and the amino acid residues in the active site.

Hit compound **21a** was docked into the active site of TLR7 to investigate potential interactions and predict its binding mode. Docking was performed using AutoDock Vina implemented in LigandScout 4.3. Figure 5A shows the pharmacophore features of the most frequently occurring interactions and binding modes. Yellow spheres represent hydrophobic features, and H-bonds are marked with red arrows. The potential binding modes were ranked according to the calculated affinities; the best-ranked pose is shown in Figure 5A. The 4-fluorophenyl substituent of compound **21a** forms a hydrophobic interaction with Phe351, and the predicted H-bonds are formed between the trifluoromethyl group and Lys432 and Tyr356. In addition, potential interactions were investigated using the protein–ligand interaction profiler (PLIP) for the highest-ranking pose. PLIP indicated hydrophobic interactions with residues Val381, Leu557, and Phe351, as shown in Figure 5B.

## Conclusions

The development of novel Toll-like receptor 7 (TLR7) agonists represents a significant challenge in the field of small-molecule immunotherapeutics. In this study, we designed and synthesized a library of 45 compounds based on the 6-(trifluoromethyl)isoxazolo[5,4*-d*]pyrimidine-4-amine scaffold. These compounds were evaluated for their TLR7 and TLR8 agonist activity with the aim of identifying a new chemotype of potent TLR7 agonists. According to biological results, our best-in-class agonist was **21a**, which showed an EC_50_ value of 7.8 μM on TLR7 and no cytotoxic effects on the HEK293 cell line, ensuring its safety for further development. Importantly, it also induced the secretion of several cytokines, including IL-1β, IL-12p70, IL-8, and TNF-α, suggesting that **21a** has the potential to modulate immune responses and initiate an immune cascade. These results make **21a** a promising compound for further development and additional studies to investigate its pharmacokinetic properties, optimize its structure, and evaluate its therapeutic efficacy.

## Experimental Section

Reagents and solvents for synthesis were purchased from commercial sources (Enamine, Apollo Scientific, Sigma-Aldrich, TCI, Acros Organics, Alfa Aesar, and Fluorochem). Analytical thin-layer chromatography was performed on silica gel aluminum sheets (0.20 mm; 60 F254; Merck, Darmstadt, Germany) and visualized under UV light and/or stained with the relevant reagents. Column chromatography was performed using silica gel 60 (particle size, 230–400 mesh) using the indicated solvents. Melting points were determined on a Reichert hotstage apparatus. ^1^H and ^13^C NMR spectra were recorded at 295 K in CDCl_3_, DMSO-*d*_*6*_, (CD_3_)_2_CO, or MeOD on a Bruker Avance III NMR spectrometer equipped with a Broadband decoupling inverse ^1^H probe. The coupling constants (*J*) are in Hz, and the splitting patterns are designated as s, singlet; bs, broad singlet; d, doublet; dd, double doublet; td, triple doublet; t, triplet; dt, double triplet; ddd, double of doublet of doublet; and m, multiplet. Mass spectra and high-resolution mass measurements were performed on an Exactive Plus orbitrap mass spectrometer at Faculty of Pharmacy, University of Ljubljana. HPLC analyses were performed on the Thermo Scientific UltiMate 3000 modular system (Thermo Fisher Scientific Inc.). The general method A used an Agilent Extend-C18 column (4.6 mm × 150 mm, 3.6 μm), thermostated at 50 °C, with injection volume, 5 μL; sample, 0.1 mg/mL in MeCN; flow rate, 1.0 mL/min; detection at 254 nm; mobile phase A: 0.1% TFA (v/v) in purified water; mobile phase B: MeCN. Gradient: 0–12 min, 10–90% B; 12–14 min, 90% B; 14–15 min, 90-10% B. The general method B used an ACQUITY UPLC HSS T3 (2.1 × 100 mm, 1.8 μm; by Waters, Milford, Massachusetts, USA) thermostated at 40 °C, with injection volume, 2 μL; sample, 0.1 mg/mL in MeCN; flow rate, 0.3 mL/min; detection at 254 nm; mobile phase A: 0.1% TFA (v/v) in purified water; mobile phase B: MeCN. Gradient: 0–6 min, 20–90% B; 6–12 min, 90%. The general method C used an ACQUITY UPLC BEH C18 column (130 Å, 2.1 mm × 50 mm, 1.7 μm; by Waters, Milford, Massachusetts, USA) thermostated at 40 °C, with injection volume, 5 μL; sample, 0.1–0.2 mg/mL in MeOH; flow rate, 0.4 mL/min; detector λ, 254 and 280 nm; mobile phase A: 0.1% TFA (v/v) in purified water; mobile phase B: MeCN. Gradient: 0–2 min, 10% B; 2–5 min, 10–90% B; 5–8 min, 90%. The purity of compounds is >95%, unless stated otherwise.

### Chemistry

#### General Procedures

##### General Procedure A for the Synthesis of Oximes (**8a**–**l**)

Hydroxylammonium chloride (1.1 equiv) was dissolved in the mixture of THF/EtOH/H_2_O (2:5:1, v/v), and then appropriate aldehyde (1.0 equiv) was added.^27^ After stirring at room temperature for 30 min, THF and EtOH were removed under reduced pressure. The remaining residue was extracted with Et_2_O (3×); the combined organic phases were washed with brine, dried over anhydrous Na_2_SO_4_, and filtered; and the solvents were evaporated under reduced pressure. The product was used in the next step without further purification unless otherwise stated.

Benzaldehyde Oxime (**8a**). Synthesized according to general procedure A using hydroxylammonium chloride (1.1 equiv., 55.0 mmol, 3.82 g) and benzaldehyde **7a** (1.0 equiv., 50.0 mmol, 5.1 mL). Yellow oil. Yield 92%. Rf (EtOAc/*n*-hexane = 2:1, v/v) = 0.58. ^1^H NMR (400 MHz, CDCl_3_) δ (ppm) 8.15 (s, 1H), 7.61–7.55 (m, 2H), 7.42–7.35 (m, 3H).

4-Chlorobenzaldehyde Oxime (**8b**). Synthesized according to general procedure A using hydroxylammonium chloride (1.1 equiv., 55.0 mmol, 3.82 g) and 4-chlorobenzaldehyde **7b** (1.0 equiv., 50.0 mmol, 7.03 g). Waxy solid. Yield: 95%. Rf (EtOAc/*n*-hexane = 1/2, v/v) = 0.36. ^1^H NMR (400 MHz, DMSO*-d*_*6*_) δ (ppm) 11.36 (s, 1H), 8.15 (s, 1H), 7.63–7.59 (m, 2H), 7.48–7.45 (m, 2H).

4-(Trifluoromethyl)benzaldehyde Oxime (**8c**). Synthesized according to general procedure A using hydroxylammonium chloride (1.1 equiv., 55.0 mmol, 3.82 g) and 4-trifluoromethyl benzaldehyde **7c** (1.0 equiv., 50.0 mmol, 6.8 mL). White solid. Yield: 92%. Mp = 66–68 °C. Rf (EtOAc/*n*-hexane = 2/1, v/v) = 0.85. ^1^H NMR (400 MHz, DMSO*-d*_*6*_) δ (ppm) 11.62 (s, 1H), 8.26 (s, 1H), 7.82 (d, *J* = 8.4 Hz, 2H), 7.77 (d, *J* = 8.4 Hz, 2H).

4-((Hydroxyimino)methyl)benzonitrile (**8d**). Synthesized according to general procedure A using hydroxylammonium chloride (1.1 equiv., 132.0 mmol, 9.17 g) and 4-cyanobenzaldehyde **7d** (1.0 equiv., 120.0 mmol, 15.74 g). White crystals. Yield: 84%. Mp = 159–162 °C. Rf (EtOAc/*n*-hexane = 2:1, v/v) = 0.57. ^1^H NMR (400 MHz, DMSO*-d*_*6*_) δ (ppm) 11.74 (s, 1H), 8.25 (s, 1H), 7.88–7.84 (m, 2H), 7.79–7.75 (m, 2H).

4-Nitrobenzaldehyde Oxime (**8e**). Synthesized according to general procedure A using hydroxylammonium chloride (1.1 equiv., 77.0 mmol, 5.35 g) and 4-nitrobenzaldehyde **7e** (1.0 equiv., 70.0 mmol, 10.58 g). Yellow crystals. Yield: 96%. Mp = 89–91 °C. Rf (EtOAc/*n*-hexane = 2/1, v/v) = 0.84. ^1^H NMR (400 MHz, acetone*-d*_*6*_) δ (ppm) 10.98 (s, 1H), 8.33 (s, 1H), 8.29 (d, *J* = 8.9 Hz, 2H), 7.96–7.89 (m, 2H).

3-Nitrobenzaldehyde Oxime (**8f**). Synthesized according to general procedure A using hydroxylammonium chloride (1.1 equiv., 77.0 mmol, 5.35 g) and 3-nitrobenzaldehyde **7f** (1.0 equiv., 70.0 mmol, 10.58 g). Yellow crystals. Yield: 80%. Mp = 102–104 °C. Rf (EtOAc/*n*-hexane = 1/2, v/v) = 0.33. ^1^H NMR (400 MHz, CDCl_3_) δ (ppm) 8.44 (t, *J* = 2.0 Hz, 1H), 8.26–8.23 (m, 1H), 8.22 (s, 1H), 7.93–7.90 (m, 1H), 7.80 (s, 1H), 7.58 (t, *J* = 8.0 Hz, 1H).

Benzo[*d*][1,3]dioxole-5-carbaldehyde Oxime (**8g**). Synthesized according to general procedure A using hydroxylammonium chloride (1.1 equiv., 77.0 mmol, 5.35 g) and 3,4-(methylenedioxy)benzaldehyde **7g** (1.0 equiv., 70.0 mmol, 10.51 g). Off-white solid. Yield: 95%. Mp = 86–88 °C. Rf (EtOAc/*n*-hexane = 1/2, v/v) = 0.53. ^1^H NMR (400 MHz, DMSO*-d*_*6*_) δ (ppm) 8.05 (s, 1H), 7.70 (d, *J* = 1.6 Hz, 1H), 7.41 (dd, *J* = 8.1, 1.6 Hz, 1H), 7.32 (s, 1H), 7.05–6.93 (m, 1H), 6.06 (d, *J* = 7.7 Hz, 2H).

4-Fluorobenzaldehyde Oxime (**8h**). Synthesized according to general procedure A using hydroxylammonium chloride (1.1 equiv., 66.0 mmol, 4.59 g) and 4-fluorobenzaldehyde **7h** (1.0 equiv., 60.0 mmol, 4.9 mL). White solid. Yield: 85%. Mp = 71–74 °C. Rf (EtOAc/*n*-hexane = 1/3, v/v) = 0.33. ^1^H NMR (400 MHz, CDCl_3_) δ (ppm) 8.56 (bs, 1H), 8.14 (s, 1H), 7.53–7.60 (m, 2H), 7.05–7.11 (m, 2H).

3-Bromo-4-fluorobenzaldehyde Oxime (**8i**). Synthesized according to general procedure A using hydroxylammonium chloride (1.1 equiv., 49.5 mmol, 3.44 g) and 3-bromo-4-fluorobenzaldehyde **7i** (1.0 equiv., 45.0 mmol, 9.14 g). White solid. Yield: 90%. Mp = 80–83 °C. Rf (EtOAc/*n*-hexane = 1/3, v/v) = 0.36. ^1^H NMR (400 MHz, DMSO*-d*_*6*_) δ (ppm) 11.44 (s, 1H), 8.15 (s, 1H), 7.91 (dd, *J* = 2,1 Hz, *J* = 6.9 Hz, 1H), 7.66 (ddd, *J* = 2.1 Hz, *J* = 4.9 Hz, *J* = 8.6 Hz, 1H), 7.42 (t, *J* = 8.7 Hz, 1H).

2-(3,4*-*Difluorophenyl)acetaldehyde Oxime (**8j**). 3,4*-*Difluorophenylacetic acid **6a** (1.0 equiv., 23.3 mmol, 4.0 g) was dissolved in anhydrous CH_2_Cl_2_. TBTU (1.05 equiv., 24.46 mmol, 7.49 g) and Et_3_N (3.0 equiv., 70.0 mmol, 9.75 mL) were added at 0 °C. After stirring for 1 h at 0 °C, *N*,*O-*dimethylhydroxylamine hydrochloride (1.0 equiv., 23.3 mmol, 2.28 g) was added and the mixture was removed from the ice bath and stirred at room temperature for 18 h. The next day, H_2_O was added and the phases were separated. The organic phase was washed with 10% citric acid, saturated NaHCO_3_, and brine; dried over Na_2_SO_4_; filtered; and concentrated under reduced pressure. The product obtained was used in the next step without further purification. It was dissolved in anhydrous THF and cooled to 0 °C, and LiAlH_4_ (1.5 equiv., 34.95 mmol, 1.328 g) was added portionwise. After 3 h, the reaction was quenched with 10% citric acid. CH_2_Cl_2_ was added, and the phases were separated. The organic phase was washed with saturated NaHCO_3_ and brine, dried over Na_2_SO_4_, filtered, and concentrated under reduced pressure. The obtained product (2-(3,4*-*difluorophenyl)acetaldehyde (**7j**) was used in the next step without further purification. The 2-(3,4*-*difluorophenyl)acetaldehyde oxime (**8j**) was synthesized according to general procedure A using hydroxylammonium chloride (1.1 equiv., 28.1 mmol, 1.96 g) and 2-(3,4*-d*ifluorophenyl)acetaldehyde (**7j**, 1.0 equiv., 25.6 mmol, 4.0 g). The product was purified by column chromatography, using EtOAc/*n*-hexane = 1/3 as the mobile phase. Colorless oil. Yield: 42%. Rf (EtOAc/*n*-hexane = 1/1, v/v) = 0.55. ^1^H NMR (400 MHz, CDCl_3_) δ 8.43 (s, 1H), 7.91 (s, 1H), 7.19–7.07 (m, 1H), 6.98–6.92 (m, 1H), 6.89–6.83 (m, 1H), 3.75–3.65 (m, 2H).

Cyclopropanecarbaldehyde Oxime (**8k**). Synthesized according to general procedure A using hydroxylammonium chloride (1.1 equiv., 73.0 mmol, 5.11 g) and cyclopropanecarboxaldehyde (**7k**, 1.0 equiv., 67.0 mmol, 5.0 mL). Colorless oil. Yield: 25%. Rf (EtOAc/*n*-hexane = 1/1, v/v) = 0.50. ^1^H NMR (400 MHz, CDCl_3_) δ 6.03 (d, *J* = 8.8 Hz, 1H), 2.38–2.17 (m, 1H), 1.03–0.91 (m, 2H), 0.70–0.58 (m, 2H).

*tert*-Butyl 4-((Hydroxyimino)methyl) Piperidine-1-carboxylate (**8l**). 1-(*tert*-Butoxycarbonyl)piperidine-4-carboxylic acid **6b** (1.0 equiv., 15.2 mmol, 3.5 g) was dissolved in anhydrous CH_2_Cl_2_. TBTU (1.05 equiv., 16.8 mmol, 4.90 g) and Et_3_N (3.0 equiv., 45.6 mmol, 6.38 mL) were added at 0 °C. After stirring for 1 h at 0 °C, *N*,*O-*dimethylhydroxylamine hydrochloride (1.0 equiv., 15.2 mmol, 1.49 g) was added and the mixture was removed from the ice bath and stirred for additional 18 h at room temperature. The next day, H_2_O was added and the phases were separated. The organic phase was washed with 10% citric acid, saturated NaHCO_3_, and brine; dried over Na_2_SO_4_; filtered; and concentrated under reduced pressure. The product obtained was used in the next step without further purification. It was dissolved in anhydrous THF and cooled to 0 °C, and LiAlH_4_ (1.5 equiv., 22.9 mmol, 0.870 g) was added portionwise. After 3 h, the reaction was quenched with 10% citric acid. CH_2_Cl_2_ was added, and the phases were separated. The organic phase was washed with saturated NaHCO_3_ and brine, dried over Na_2_SO_4_, filtered, and concentrated under reduced pressure. The product (*tert*-butyl 4-formylpiperidine-1-carboxylate, **7l**) was used in the next step without further purification. The *tert*-butyl 4-((hydroxyimino)methyl)piperidine-1-carboxylate (**8l**) was synthesized according to general procedure A using hydroxylammonium chloride (1.1 equiv., 12.2 mmol, 0.85 g) and *tert*-butyl 4-formylpiperidine-1-carboxylate (**7l**, 1.0 equiv., 11.1 mmol, 2.368 g). Colorless oil. Yield: 83%. Rf (EtOAc/*n*-hexane = 1/3, v/v) = 0.30. ^1^H NMR (400 MHz, CDCl_3_) δ 8.00 (s, 1H), 7.35 (d, *J* = 5.7 Hz, 1H), 2.84–2.71 (m, 1H), 2.47–2.34 (m, 1H), 1.86–1.72 (m, 4H), 1.46 (s 9H), 1.52–1.44 (m, 2H), 1.32–1.18 (m, 1H).

#### General Procedure B for the Synthesis of *N*-Hydroxyimidoyl Chlorides (**9a**–**l**)

Appropriate aldehyde oxime (**8a**–**l**) (1.0 equiv) was dissolved in anhydrous DMF and cooled to 0 °C in an ice batch. NCS (1.0 equiv) was added portionwise to the reaction mixture, which was stirred for 16 h at room temperature.^27^ Then, the mixture of *n*-hexane/Et_2_O (2:1, v/v) and H_2_O was added and transferred to a separating funnel. The phases were separated, and the water phase was extracted with Et_2_O. Combined organic phases were washed with brine, dried over anhydrous Na_2_SO_4_, and filtered; and the solvents were removed under reduced pressure to yield the *N*-hydroxyimidoyl chloride (**9a**–**l**), which was immediately used in the next step without any purification.

*N*-Hydroxybenzimidoyl Chloride (**9a**). Synthesized according to general procedure B using compound **8a** (1.0 equiv., 45.9 mmol, 5.57 g) and NCS (1.0 equiv., 45.9 mmol, 6.14 g). Yellow crystals. Yield: 43%. Mp = 106–108 °C. Rf (EtOAc/*n*-hexane = 1:1, v/v) = 0.55. ^1^H NMR (400 MHz, CDCl_3_) δ (ppm) 8.11 (s, 1H), 7.90–7.80 (m, 2H), 7.48–7.35 (m, 3H).

4-Chloro-*N*-hydroxybenzimidoyl Chloride (**9b**). Synthesized according to general procedure B using compound **8b** (1.0 equiv., 50.0 mmol, 7.78 g) and NCS (1.0 equiv., 50.0 mmol, 9.35 g). Yellow oil. Yield: 85%. Rf (EtOAc/*n*-hexane = 1/2, v/v) = 0.17. ^1^H NMR (400 MHz, DMSO*-d*_*6*_) δ (ppm) 12.57 (s, 1H), 7.83–7.79 (m, 2H), 7.57–7.53 (m, 2H).

*N*-Hydroxy-4-(trifluoromethyl)benzimidoyl Chloride (**9c**). Synthesized according to general procedure B using compound **8c** (1.0 equiv., 46.0 mmol, 4.99 g) and NCS (1.0 equiv., 46.0 mmol, 3.2 g). Yellow oil. Yield: 83%. Rf (EtOAc/*n*-hexane = 1/2, v/v) = 0.78. ^1^H NMR(400 MHz, DMSO*-d*_6_) δ (ppm) 12.79 (s, 1H), 8.05–7.99 (m, 2H), 7.88–7.83 (m, 2H).

4-Cyano-*N*-hydroxybenzimidoyl Chloride (**9d**). Synthesized according to general procedure B using compound **8d** (1.0 equiv., 101.0 mmol, 14.76 g) and NCS (1.0 equiv., 101.0 mmol, 13.49 g). White solid. Yield: 29%. Mp = 133–135 °C. Rf (EtOAc/*n*-hexane = 2:1, v/v) = 0.79. ^1^H NMR (400 MHz, DMSO*-d*_*6*_) δ (ppm) 12.88 (s, 1H), 8.04–7.85 (m, 4H).

*N*-Hydroxy-4-nitrobenzimidoyl Chloride (**9e**). Synthesized according to general procedure B using compound **8e** (1.0 equiv., 67.3 mmol, 11.18 g) and NCS (1.0 equiv., 67.3 mmol, 8.98 g). Colorless oil. Yield: 95%. Rf (EtOAc/*n*-hexane = 1/2, v/v) = 0.57. ^1^H NMR (400 MHz, DMSO*-d*_*6*_) δ (ppm) 12.99 (s, 1H), 8.36–8.30 (m, 2H), 8.08–8.04 (m, 2H).

*N*-Hydroxy-3-nitrobenzimidoyl Chloride (**9f**). Synthesized according to general procedure B using compound **8f** (1.0 equiv., 56.1 mmol, 9.31 g) and NCS (1.0 equiv., 56.1 mmol, 7.49 g). Yellow oil. Yield: 95%. Rf (EtOAc/*n*-hexane = 1/2, v/v) = 0.35. ^1^H NMR (400 MHz, CDCl_3_) δ (ppm) 12.86 (s, 1H), 8.52 (t, *J* = 2,0 Hz, 1H), 8.36–8.33 (m, 1H), 8.25–8.22 (m, 1H), 7.79 (t, *J* = 8.1 Hz, 1H).

*N*-Hydroxybenzo[*d*][1,3]dioxole-5-carbimidoyl Chloride (**9g**). Synthesized according to general procedure B using compound **8g** (1.0 equiv., 70.0 mmol, 12.9 g) and NCS (1.0 equiv., 70.0 mmol, 9.35 g). Orange oil. Yield: 85%. Rf (EtOAc/*n*-hexane = 1/2, v/v) = 0.61. ^1^H NMR (400 MHz, DMSO*-d*_*6*_) δ (ppm) 12.24 (s, 1H), 7.32 (dd, *J* = 8.2, 1.9 Hz, 1H), 7.27 (d, *J* = 1.8 Hz, 1H), 7.00 (d, *J* = 8.2 Hz, 1H), 6.11 (s, 2H).

4-Fluoro-*N*-hydroxybenzimidoyl Chloride (**9h**). Synthesized according to general procedure B using compound **8h** (1.0 equiv., 51.0 mmol, 7.09 g) and NCS (1.0 equiv., 51.0 mmol, 6.80 g). Light-yellow crystals. Yield: 75%. Mp = 54–57 °C. Rf (EtOAc/*n*-hexane = 1/3, v/v) = 0.50. ^1^H NMR (400 MHz, DMSO*-d*_*6*_) δ (ppm) 12.44 (s, 1H), 7.80–7.88 (m, 2H), 7.29–7.36 (m, 2H).

3-Bromo-4-fluoro-*N*-hydroxybenzimidoyl Chloride (**9i**). Synthesized according to general procedure B using compound **8i** (1.0 equiv., 40.5 mmol, 8.84 g) and NCS (1.0 equiv., 40.5 mmol, 5.41 g). White crystals. Yield: 75%. Mp = <200 °C. Rf (EtOAc/*n*-hexane = 1/3, v/v) = 0.52. ^1^H NMR (400 MHz, DMSO*-d*_*6*_) δ (ppm) 12.65 (s, 1H), 8.03 (dd, *J* = 2.3 Hz, *J* = 6.6 Hz, 1H), 7.84 (ddd, *J* = 2.3 Hz, *J* = 4.7 Hz, *J* = 8.7 Hz, 1H), 7.50 (t, *J* = 8.7 Hz, 1H).

2-(3,4*-*Difluorophenyl)-*N*-hydroxyacetimidoyl Chloride (**9j**). Synthesized according to general procedure B using compound **8j** (1.0 equiv., 10.1 mmol, 1.74 g) and NCS (1.0 equiv., 10.1 mmol, 1.35 g). Yellow oil. Yield: 95%. Rf (EtOAc/*n*-hexane = 1/1, v/v) = 0.65. ^1^H NMR (400 MHz, CDCl_3_) δ 8.52 (bs, 1H), 7.19–7.06 (m, 2H), 7.03–6.91 (m, 1H), 3.76 (d, *J* = 0.7 Hz, 2H).

*N*-Hydroxycyclopropanecarbimidoyl Chloride (**9k**). Synthesized according to general procedure B using compound **8k** (1.0 equiv., 16.8 mmol, 1.42 g) and NCS (1.0 equiv., 16.8 mmol, 2.24 g). Colorless oil. Yield: 95%. Rf (EtOAc/*n*-hexane = 1/1, v/v) = 0.50. ^1^H NMR (400 MHz, CDCl_3_) δ 8.57 (bs, 1H), 1.89–1.73 (m, 1H), 1.10–0.89 (m, 2H), 0.89–0.73 (m, 2H).

*tert*-butyl 4-(Chloro(hydroxyimino)methyl)piperidine-1-carboxylate (**9l**). Synthesized according to general procedure B using compound **8l** (1.0 equiv., 9.1 mmol, 2.09 g) and NCS (1.0 equiv., 9.1 mmol, 1.23 g). Yellow oil. Yield: 93%. Rf (EtOAc/*n*-hexane = 1/1, v/v) = 0.50. ^1^H NMR (400 MHz, CDCl_3_) δ 10.12 (bs, 1H), 4.13 (s, 2H), 2.80–2.72 (m, 1H), 2.68–2.49 (m, 1H), 2.15–2.01 (m, 1H), 1.89–1.79 (m, 2H), 1.77–1.56 (m, 2H), 1.44 (s, 9H).

#### General Procedure C for the Synthesis of 5-Aminoisoxazole-4-carboxamides (**10a**–**l**)

A solution of NaOEt in EtOH was prepared from sodium (1.5 equiv) and absolute EtOH (∼30 equiv). The freshly prepared NaOEt was added dropwise to a stirred solution of 2-cyanoacetamide (1.0 equiv) in absolute EtOH at 50 °C.^27^ After the addition of NaOEt, the resulting suspension was cooled to 0 °C. Appropriate *N*-hydroxyimidoyl chloride (**9a**–**l**) (1.0 equiv) from general procedure B was dissolved in absolute EtOH and added dropwise to the suspension. The reaction mixture was stirred for additional 30 min at 0 °C and then overnight (16 h) at 80 °C. The next day, EtOH was removed under reduced pressure and H_2_O was added to the remaining residue. The solid that precipitated was filtered off and recrystallized from MeOH, if necessary, unless otherwise stated.

4-Amino-3-phenylisoxazole-5-carboxamide (**10a**). Synthesized according to general procedure C from NaOEt solution (1.5 equiv., 29.7 mmol, 0.680 g of Na, 30 mL of absolute ethanol), 2-cyanoacetamide (1.0 equiv., 19.82 mmol, 1.66 g), and compound **9a** (1.0 equiv., 19.8 mmol, 3.08 g). Brown solid. Mp = 162–164 °C. Yield: 20%. Rf (EtOAc/*n*-hexane = 2:1, v/v) = 0.55. ^1^H NMR (400 MHz, DMSO*-d*_*6*_) δ (ppm) 7.67 (bs, 2H), 7.58–7.50 (m, 5H), 2H are exchanged with the solvent.

4-Amino-3-(4-chlorophenyl)isoxazole-5-carboxamide (**10b**). Synthesized according to general procedure C from NaOEt solution (1.5 equiv., 75.0 mmol, 1.72 g of Na, 50 mL of absolute ethanol), 2-cyanoacetamide (1.0 equiv., 50.0 mmol, 4.3 g), and compound **9b** (1.0 equiv., 50.0 mmol, 9.5 g). Brown solid. Yield: 45%. Rf (EtOAc/*n*-hexane = 1/2, v/v) = 0.09. ^1^H NMR (400 MHz, DMSO*-d*_*6*_) δ (ppm) 7.66 (s, 2H), 7.58 (s, 4H), 6.40 (bs, 2H).

4-Amino-3-(4-(trifluoromethyl)phenyl) Isoxazole-5-carboxamide (**10c**). Synthesized according to general procedure C from NaOEt solution (1.5 equiv., 57.6 mmol, 1.32 g of Na, 50 mL of absolute ethanol), 2-cyanoacetamide (1.0 equiv., 38.4 mmol, 3.23 g), and compound **9c** (1.0 equiv., 38.4 mmol, 8.58 g). Waxy brown solid. Yield: 75%. Rf (EtOAc/*n*-hexane = 2/1, v/v) = 0.50. ^1^H NMR (400 MHz, DMSO*-d*_*6*_) δ (ppm) 7.87 (d, *J* = 8.1 Hz, 2H), 7.78 (d, *J* = 8.1 Hz, 2H), 7.68 (s, 1H), 6.60 (bs, 1H), 2H are exchanged with the solvent.

4-Amino-3-(4-cyanophenyl)isoxazole-5-carboxamide (**10d**). Synthesized according to general procedure C from NaOEt solution (1.5 equiv., 44.6 mmol, 1.02 g of Na, 30 mL of absolute ethanol), 2-cyanoacetamide (1.0 equiv., 29.7 mmol, 2.50 g), and compound **9d** (1.0 equiv., 29.7 mmol, 5.36 g). Light-brown solid. Mp = 198–200 °C. Yield: 95%. Rf (EtOAc/*n*-hexane = 2:1, v/v) = 0.45. ^1^H NMR (400 MHz, DMSO*-d*_*6*_) δ (ppm) 7.98–7.93 (m, 2H), 7.79–7.71 (m, 2H), 7.66 (bs, 2H), 2H are exchanged with the solvent.

4-Amino-3-(4-nitrophenyl)isoxazole-5-carboxamide (**10e**). Synthesized according to general procedure C from NaOEt solution (1.5 equiv., 100.96 mmol, 2.32 g of Na, 100 mL of absolute ethanol), 2-cyanoacetamide (1.0 equiv., 67.3 mmol, 5.66 g), and compound **9e** (1.0 equiv., 67.3 mmol, 18.45 g). The product was purified by column chromatography, using EtOAc as the mobile phase. Brown oil. Yield: 4%. Rf (EtOAc) = 0.59. ^1^H NMR (400 MHz, DMSO*-d*_*6*_) δ (ppm) 8.35–8.30 (m, 2H), 7.88–7.80 (m, 2H), 7.71 (s, 2H), 6.60 (bs, 2H).

4-Amino-3-(3-nitrophenyl)isoxazole-5-carboxamide (**10f**). Synthesized according to general procedure C from NaOEt solution (1.5 equiv., 85.05 mmol, 1.95 g of Na, 50 mL of absolute ethanol), 2-cyanoacetamide (1.0 equiv., 56.7 mmol, 4.77 g), and compound **9f** (1.0 equiv., 56.7 mmol, 17.62 g). Brown solid. Yield: 76%. Rf (EtOAc/*n*-hexane = 1/2, v/v) = 0.70. ^1^H NMR (400 MHz, DMSO*-d*_*6*_) δ (ppm) 8.38–8.34 (m, 2H), 8.03–8.00 (m, 1H), 7.82–7.77 (m, 1H), 6.79 (s, 2H), 6.60 (bs, 2H).

4-Amino-3-(benzo[*d*][1,3]dioxol-5-yl)isoxazole-5-carboxamide (**10g**). Synthesized according to general procedure C from NaOEt solution (1.5 equiv., 89.55 mmol, 2.06 g of Na, 100 mL of absolute ethanol), 2-cyanoacetamide (1.0 equiv., 59.7 mmol, 5.02 g), and compound **9g** (1.0 equiv., 59.7 mmol, 11.91 g). Off-white solid. Yield: 62%. Rf (EtOAc/*n*-hexane = 2/1, v/v) = 0.07. ^1^H NMR (400 MHz, DMSO*-d*_*6*_) δ (ppm) 7.65 (s, 2H), 7.09–7.03 (m, 3H), 6.11 (s, 2H), 5.75 (bs, 2H).

4-Amino-3-(4-fluorophenyl)isoxazole-5-carboxamide (**10h**). Synthesized according to general procedure C from NaOEt solution (1.5 equiv., 57.2 mmol, 1.32 g of Na, 30 mL of absolute ethanol), 2-cyanoacetamide (1.0 equiv., 38.1 mmol, 3.2 g), and compound **9h** (1.0 equiv., 38.1 mmol, 6.62 g). Yellow solid. Mp = 171–173 °C. Yield: 32%. Rf (EtOAc/*n*-hexane = 2/1, v/v) = 0.24. ^1^H NMR (400 MHz, DMSO*-d*_*6*_) δ (ppm) 7.66 (s, 2H), 7.59–7.63 (m, 2H), 7.32–7.39 (m, 2H), 2H are exchanged with the solvent.

4-Amino-3-(3-bromo-4-fluorophenyl)isoxazole-5-carboxamide (**10i**). Synthesized according to general procedure C from NaOEt solution (1.5 equiv., 60.8 mmol, 1.4 g of Na, 30 mL of absolute ethanol), 2-cyanoacetamide (1.0 equiv., 40.5 mmol, 3.41 g), and compound **9i** (1.0 equiv., 40.5 mmol, 10.23 g). Light-yellow solid. Mp = 163–166 °C. Yield: 30%. Rf (EtOAc/*n*-hexane = 2/1, v/v) = 0.30. ^1^H NMR (400 MHz, DMSO*-d*_*6*_) δ (ppm) 7.87 (dd, *J* = 6.7, 2.1 Hz, 1H), 7.70 (s, 2H), 7.61 (ddd, *J* = 8.6, 4.9, 2.1 Hz, 1H), 7.51 (t, *J* = 8.7 Hz, 2H), 2H are exchanged with the solvent.

4-Amino-3-(3,4*-*difluorobenzyl)isoxazole-5-carboxamide (**10j**). Synthesized according to general procedure C from NaOEt solution (1.5 equiv., 17.4 mmol, 0.40 g of Na, 10 mL of absolute ethanol), 2-cyanoacetamide (1.0 equiv., 11.6 mmol, 0.98 g), and compound **9j** (1.0 equiv., 11.6 mmol, 2.39 g). Yellow solid. Yield: 72%. Rf (EtOAc/*n*-hexane = 2/1, v/v) = 0.20. ^1^H NMR (400 MHz, DMSO*-d*_*6*_) δ 7.53 (bs, 2H), 7.44–7.24 (m, 2H), 7.06 (ddt, *J* = 8.4, 4.3, 1.8 Hz, 1H), 6.78 (bs, 2H), 4.11 (s, 2H).

4-Amino-3-cyclopropylisoxazole-5-carboxamide (**10k**). Synthesized according to general procedure C from NaOEt solution (1.5 equiv., 30.2 mmol, 0.69 g of Na, 15 mL of absolute ethanol), 2-cyanoacetamide (1.0 equiv., 20.1 mmol, 1.70 g), and compound **9k** (1.0 equiv., 20.1 mmol, 2.40 g). After completion of the reaction, EtOH was removed under reduced pressure. Ethyl acetate and CH_2_Cl_2_ were added (v/v = 1/1) to the remaining oily residue. The solid that precipitated was filtered off. Filtrate was evaporated under reduced pressure and was used in the next step without further purification. Yellow oil. Yield: 67%. Rf (EtOAc/*n*-hexane = 2/1, v/v) = 0.20. ^1^H NMR (400 MHz, DMSO*-d*_*6*_) δ 7.50 (bs, 2H), 6.91 (bs, 2H), 2.08 (tt, *J* = 8.3, 5.1 Hz, 1H), 0.97–0.86 (m, 2H), 0.81–0.69 (m, 2H).

*tert*-Butyl 4-(4-Amino-5-carbamoylisoxazol-3-yl)piperidine-1-carboxylate (**10l**). Synthesized according to general procedure C from NaOEt solution (1.5 equiv., 13.7 mmol, 0.32 g, 10 mL of absolute ethanol), 2-cyanoacetamide (1.0 equiv., 9.1 mmol, 0.77 g), and compound **9l** (1.0 equiv., 9.1 mmol, 2.41 g). After completion of the reaction, EtOH was removed under reduced pressure. H_2_O and MTBE were added to the remaining oily residue, and the phases were separated. The water phase was washed with 10 mL of MTBE. The organic phase was dried with Na_2_SO_4_ and filtered, and the solvent was evaporated under reduced pressure. White solid. Yield: 78%. Rf (EtOAc) = 0.20. ^1^H NMR (400 MHz, CDCl_3_) δ 6.35 (bs, 2H), 5.45 (bs, 2H), 4.18 (bs, 2H), 2.91–2.81 (m, 1H), 2.79–2.69 (m, 1H), 2.15–1.97 (m, 2H), 1.91–1.75 (m, 2H), 1.46 (s, 9H), 1.43–1.38 (m, 1H).

#### General Procedure D for the Synthesis of Isoxazolo[5,4*-d*]pyrimidin-4-oles (**11a**–**l**)

To freshly prepare NaOEt from Na (3.0 equiv) and abs. EtOH (∼50 mL) were added dropwise appropriate 5-aminoisoxazole-4-carboxamide (**10a**–**l**) (1.0 equiv) and ethyl trifluoroacetate (1.2 equiv).^26^ The reaction mixture was stirred and heated for 24–48 h under reflux. After completion of the reaction, the solvents were removed under reduced pressure and water (50 mL) was added to the remaining residue. If a precipitate was formed, it was filtered off and 1 M HCl was added to the alkaline solution to adjust the pH to 2–3. The resulting precipitate was filtered off and recrystallized from MeOH if necessary.

3-Phenyl-6-(trifluoromethyl)isoxazolo[5,4*-d*]pyrimidin-4-ol (**11a**). Synthesized according to general procedure D from NaOEt solution (3.0 equiv., 11.9 mmol, 0.27 g of Na, 50 mL of absolute ethanol), compound **10a** (1.0 equiv., 3.95 mmol, 0.80 g), and ethyl trifluoroacetate (1.2 equiv., 4.74 mmol, 0.6 mL). Off-white solid. Yield: 46%. Mp = 160–162 °C. Rf (EtOAc/*n*-hexane = 2:1, v/v) = 0.27. ^1^H NMR (400 MHz, DMSO*-d*_*6*_) δ (ppm) 8.25–8.27 (m, 2H), 7.66–7.55 (m, 3H), 3.74 (bs, 1H), 1H (OH) is exchanged with the solvent.

3-(4-Chlorophenyl)-6-(trifluoromethyl)isoxazolo[5,4*-d*]pyrimidin-4-ol (**11b**). Synthesized according to general procedure D from NaOEt solution (3.0 equiv., 68.4 mmol, 1.57 g of Na, 50 mL of absolute ethanol), compound **10b** (1.0 equiv., 22.8 mmol, 5.43 g), and ethyl trifluoroacetate (1.2 equiv., 46.2 mmol, 5.5 mL). White solid. Yield: 93%. Rf (EtOAc/*n*-hexane = 2/1, v/v) = 0.05. ^1^H NMR (400 MHz, DMSO*-d*_*6*_) δ (ppm) 8.35–8.29 (m, 1H), 7.72–7.67 (m, 2H), 7.54–7.49 (m, 1H), 4.07 (bs, 1H).

6-(Trifluoromethyl)-3-(4-(trifluoromethyl)phenyl)isoxazolo[5,4*-d*]pyrimidin-4-ol (**11c**). Synthesized according to general procedure D from NaOEt solution (3.0 equiv., 87 mmol, 2.01 g of Na, 50 mL of absolute ethanol), compound **10c** (1.0 equiv., 29 mmol, 7.85 g), and ethyl trifluoroacetate (1.2 equiv., 34.7 mmol, 4.13 mL). White solid. Yield: 95%. Mp = 149–151 °C. Rf (EtOAc/*n*-hexane = 2/1, v/v) = 0.43. ^1^H NMR (400 MHz, DMSO*-d*_*6*_) δ (ppm) 8.57–8.53 (m, 2H), 7.97 (d, *J* = 8.3 Hz, 2H), 1H (OH) is exchanged with the solvent.

4-(4-Hydroxy-6-(trifluoromethyl)isoxazolo[5,4*-d*]pyrimidin-3-yl)benzonitrile (**11d**). Synthesized according to general procedure D from NaOEt solution (3.0 equiv., 51.0 mmol, 1.18 g of Na, 50 mL of absolute ethanol), compound **10d** (1.0 equiv., 17.0 mmol, 3.89 g), and ethyl trifluoroacetate (1.2 equiv., 3.6 mmol, 2.4 mL). The product was purified by column chromatography, using EtOAc/*n*-hexane (4:1, v/v) as the mobile phase. Light-yellow waxy solid. Yield: 13%. Rf (EtOAc/*n*-hexane = 2:1, v/v) = 0.30. ^1^H NMR (400 MHz, DMSO*-d*_*6*_) δ (ppm) 8.75–8.71 (m, 2H), 8.03–7.99 (m, 2H), 6.55 (bs, 1H).

3-(4-Nitrophenyl)-6-(trifluoromethyl)isoxazolo[5,4*-d*]pyrimidin-4-ol (**11e**). Synthesized according to general procedure D from NaOEt solution (3.0 equiv., 8.76 mmol, 0.21 g of Na, 30 mL of absolute ethanol), compound **10e** (1.0 equiv., 2.92 mmol, 0.73 g), and ethyl trifluoroacetate (1.2 equiv., 4.96 mmol, 0.59 mL). Yellow solid. Yield: 41%. Mp = 198–200 °C. Rf (EtOAc/*n*-hexane = 2/1, v/v) = 0.16. ^1^H NMR (400 MHz, DMSO*-d*_*6*_) δ (ppm) 8.60–8.55 (m, 2H), 8.47–8.41 (m, 2H), 1H (OH) is exchanged with the solvent.

3-(3-Nitrophenyl)-6-(trifluoromethyl)isoxazolo[5,4*-d*]pyrimidin-4-ol (**11f**). Synthesized according to general procedure D from NaOEt solution (3.0 equiv., 128.4 mmol, 2.95 g of Na, 100 mL of absolute ethanol), compound **10f** (1.0 equiv., 42.8 mmol, 10.42 g), and ethyl trifluoroacetate (1.2 equiv., 72.7 mmol, 8.7 mL). Brown solid. Yield: 29%. Rf (EtOAc/*n*-hexane = 1/3, v/v) = 0.50. ^1^H NMR (400 MHz, DMSO*-d*_*6*_) δ (ppm) 8.40–8.35 (m, 2H), 7.85–7.80 (m, 1H), 7.75–7.68 (m, 1H), 5.30 (bs, 1H).

3-(Benzo[*d*][1,3]dioxol-5-yl)-6-(trifluoromethyl)isoxazolo[5,4*-d*]pyrimidin-4-ol (**11g**). Synthesized according to general procedure D from NaOEt solution 3.0 equiv., 111.9 mmol, 2.57 g of Na, 100 mL of absolute ethanol), compound **10g** (1.0 equiv., 37.3 mmol, 9.23 g), and ethyl trifluoroacetate (1.2 equiv., 74.7 mmol, 8.9 mL). Yellow solid. Yield: 69%. Rf (EtOAc/*n*-hexane = 2/1, v/v) = 0.15. ^1^H NMR (400 MHz, DMSO*-d*_*6*_) δ (ppm) 8.12 (bs, 1H), 7.23–7.20 (m, 2H), 7.14 (d, *J* = 8.2 Hz, 1H), 6.15 (s, 2H).

3-(4-Fluorophenyl)-6-(trifluoromethyl)isoxazolo[5,4*-d*]pyrimidin-4-ol (**11h**). Synthesized according to general procedure D from NaOEt solution (3.0 equiv., 50.1 mmol, 1.15 g of Na, 50 mL of absolute ethanol), compound **10h** (1.0 equiv., 16.7 mmol, 3.69 g), and ethyl trifluoroacetate (2.0 equiv., 33.4 mmol, 4 mL). Off-white solid. Yield: 87%. Mp = 162–164 °C. Rf (EtOAc) = 0.48. ^1^H NMR (400 MHz, DMSO*-d*_*6*_) δ (ppm) 8.34–8.41 (m, 2H), 7.41–7.48 (m, 2H), 1H (OH) is exchanged with the solvent.

3-(3-Bromo-4-fluorophenyl)-6-(trifluoromethyl)isoxazolo[5,4*-d*]pyrimidin-4-ol (**11i**). Synthesized according to general procedure D from NaOEt solution (3.0 equiv., 36.9 mmol, 0.85 g of Na, 50 mL of absolute ethanol), compound **10i** (1.0 equiv., 12.3 mmol, 3.68 g), and ethyl trifluoroacetate (2.0 equiv., 24.6 mmol, 2.9 mL). White solid. Yield: 66%. Mp = 181–185 °C. Rf (EtOAc) = 0.18. ^1^H NMR (400 MHz, DMSO*-d*_*6*_) δ (ppm) 8.89 (dd, *J* = 6.9, 2.2 Hz, 1H), 8.44 (ddd, *J* = 8.7, 4.8, 2.2 Hz, 1H), 7.59 (t, *J* = 8.7 Hz, 1H), 1H (OH) is exchanged with the solvent.

3-(3,4*-*Difluorobenzyl)-6-(trifluoromethyl)isoxazolo[5,4*-d*]pyrimidin-4-ol (**11j**). Synthesized according to general procedure D from NaOEt solution (3.0 equiv., 25.2 mmol, 0.58 g of Na, 50 mL of absolute ethanol), compound **10j** (1.0 equiv., 8.4 mmol, 2.13 g), and ethyl trifluoroacetate (1.2 equiv., 10.8 mmol, 2.0 mL). Off-white solid. Yield: 38%. Rf (CH_2_Cl_2_/MeOH = 9/1, v/v) = 0.2. ^1^H NMR (400 MHz, CDCl_3_) δ 7.36–7.24 (m, 2H), 7.20–7.03 (m, 1H), 4.26 (s, 2H).

3-Cyclopropyl-6-(trifluoromethyl)isoxazolo[5,4*-d*]pyrimidin-4-ol (**11k**). Synthesized according to general procedure D from NaOEt solution (3.0 equiv., 35.1 mmol, 0.40 g of Na, 10 mL of absolute ethanol), compound **10k** (1.0 equiv., 11.7 mmol, 2.39 g), and ethyl trifluoroacetate (1.2 equiv., 14.0 mmol, 0.98 mL). Yellow solid. Yield: 72%. Rf (EtOAc/*n*-hexane = 2/1, v/v) = 0.25. ^1^H NMR (400 MHz, DMSO*-d*_*6*_) δ 2.29 (tt, *J* = 8.3, 5.3 Hz, 1H), 1.38–0.99 (m, 4H).

*tert*-Butyl 4-(4-Hydroxy-6-(trifluoromethyl)isoxazolo[5,4*-d*]pyrimidin-3-yl)piperidine-1-carboxylate (**11l**). Synthesized according to general procedure D from NaOEt solution (3.0 equiv., 21.3 mmol, 0.49 g of Na, 50 mL absolute ethanol), compound **10l** (1.0 equiv., 7.1 mmol, 2.21 g), and ethyl trifluoroacetate (2.2 equiv., 15.62 mmol, 2.02 mL). Off-white solid. Yield: 74%. Rf (CH_2_Cl_2_/MeOH = 9/1, v/v) = 0.30. ^1^H NMR (400 MHz, DMSO*-d*_*6*_) δ 4.38 (s, 1H), 4.01 (d, *J* = 13.1 Hz, 2H), 3.53–3.42 (m, 1H), 3.10 (dt, *J* = 11.5, 3.7 Hz, 1H), 2.93–2.87 (m, 1H), 1.92 (dd, *J* = 12.8, 3.5 Hz, 2H), 1.85–1.75 (m, 1H), 1.42 (s, 9H).

#### General Procedure E for the Synthesis of 4-Chloroisoxazolo[5,4*-d*]pyrimidines (**12a**–**l**)

POCl_3_ was added to isoxazolo[5,4*-d*]pyrimidin-4-ols (**11a**–**l**) in a sealed tube.^26^ The reaction was slowly heated to 120 °C and then stirred at 120° for 16–48 h. After completion of the reaction, the sealed tube was cooled to 0 °C. Toluene (100 mL) was added, and the resulting solution was evaporated under reduced pressure. The oily residue was dissolved in EtOAc (150 mL) and stirred at 0 °C while a saturated solution of NaHCO_3_ (50 mL) was slowly added. The phases were separated, and the organic phase was washed again with saturated solution of NaHCO_3_ (50 mL) and brine (50 mL). The organic phase was dried over anhydrous Na_2_SO_4_ andfiltered, and EtOAc was evaporated under reduced pressure. The residue was purified by flash column chromatography (EtOAc/*n*-hexane).

4-Chloro-3-phenyl-6-(trifluoromethyl)isoxazolo[5,4*-d*]pyrimidine (**12a**). Synthesized according to general procedure E from compound **11a** (1.0 equiv., 1.82 mmol, 0.51 g) and excess POCl_3_ (∼54 mmol, 5 mL). The product was purified by column chromatography using EtOAc/*n*-hexane (1:1, v/v) as the mobile phase. Brown solid. Yield 47%. Mp = 63–65 °C. Rf (EtOAc/*n*-hexane = 2:1, v/v) = 0.81. ^1^H NMR (400 MHz, DMSO*-d*_*6*_) δ (ppm) 8.26–8.21 (m, 2H), 7.59–7.63 (m, 3H).

4-Chloro-3-(4-chlorophenyl)-6-(trifluoromethyl)isoxazolo[5,4*-d*]pyrimidine (**12b**). Synthesized according to general procedure E from compound **11b** (1.0 equiv., 21.2 mmol, 6.69 g) and excess POCl_3_ (∼218 mmol, 20 mL). The product was purified by column chromatography using EtOAc/*n*-hexane (1:3, v/v) as the mobile phase. Yellow solid. Yield: 39%. Mp = 87–89 °C. Rf (EtOAc/*n*-hexane = 1/3, v/v) = 0.6. ^1^H NMR (400 MHz, DMSO*-d*_*6*_) δ (ppm) 8.32–8.23 (m, 2H), 7.74–7.64 (m, 2H).

4-Chloro-6-(trifluoromethyl)-3-(4-(trifluoromethyl)phenyl)isoxazolo[5,4*-d*]pyrimidine (**12c**). Synthesized according to general procedure E from compound **11c** (1.0 equiv., 9.0 mmol, 3.14 g) and excess POCl_3_ (∼218 mmol, 20 mL). Product was used in the next step without further purification. Yield: 35%. Mp = 79–92 °C. Rf (EtOAc/*n*-hexane = 2/1, v/v) = 0.97. ^1^H NMR (400 MHz, DMSO*-d*_*6*_) δ (ppm) 8.50–8.43 (m, 2H), 8.05–7.97 (m, 2H).

4-(4-Chloro-6-(trifluoromethyl)isoxazolo[5,4*-d*]pyrimidin-3-yl)benzonitrile (**12d**). Synthesized according to general procedure E from compound **11d** (1.0 equiv., 2.14 mmol, 0.66 g) and excess POCl_3_ (∼54 mmol, 5 mL). The product was purified by column chromatography using EtOAc/*n*-hexane (1:1, v/v) as the mobile phase. Brown solid. Yield: 9.1%. Mp = 77–79 °C. Rf = 0.74 (EtOAc/*n*-hexane = 1:1, v/v). ^1^H NMR (400 MHz, CDCl_3_) δ (ppm) 8.00–7.96 (m, 2H), 7.94–7.90 (m, 2H).

4-Chloro-3-(4-nitrophenyl)-6-(trifluoromethyl)isoxazolo[5,4*-d*]pyrimidine (**12e**). Synthesized according to general procedure E from compound **11e** (1.0 equiv., 1.2 mmol, 0.39 g) and excess POCl_3_ (∼54 mmol, 5 mL). The product was purified by column chromatography using EtOAc/*n*-hexane (1:2, v/v) as the mobile phase. Yellow oil. Yield: 19%. Rf (EtOAc/*n*-hexane = 1:2, v/v) = 0.56. ^1^H NMR (400 MHz, DMSO*-d*_*6*_) δ (ppm) 8.59–8.50 (m, 2H), 8.47–8.42 (m, 2H).

4-Chloro-3-(3-nitrophenyl)-6-(trifluoromethyl)isoxazolo[5,4*-d*]pyrimidine (**12f**). Synthesized according to general procedure E from compound **11f** (1.0 equiv., 12.8 mmol, 4.44 g) and excess POCl_3_ (∼218 mmol, 20 mL). The product was purified by column chromatography using EtOAc/*n*-hexane (1:1, v/v) as the mobile phase. Off-white solid. Yield: 7%. Rf (EtOAc/*n*-hexane = 1:4, v/v) = 0.37. ^1^H NMR (400 MHz, DMSO*-d*_*6*_) δ (ppm) 9.20 (t, *J* = 1.9 Hz, 1H), 8.68–8.65 (m, 1H), 8.49–8.45 (m, 1H), 7.92 (t, *J* = 8.1 Hz, 1H).

3-(Benzo[*d*][1,3]dioxol-5-yl)-4-chloro-6-(trifluoromethyl)isoxazolo[5,4*-d*]pyrimidine (**12g**). Synthesized according to general procedure E from compound **11g** (1.0 equiv., 35.5 mmol, 4.0 g) and excess POCl_3_ (∼218 mmol, 20 mL). The product was purified by column chromatography using EtOAc/*n*-hexane (1:1, v/v) as the mobile phase. Brown oil. Yield: 22%. Rf (EtOAc/*n*-hexane = 1:1, v/v) = 0.61. ^1^H NMR (400 MHz, DMSO*-d*_*6*_) δ (ppm) 7.91 (dd, *J* = 8.2, 1.7 Hz, 1H), 7.76 (d, *J* = 1.7 Hz, 1H), 7.14 (d, *J* = 8.2 Hz, 1H), 6.16 (s, 2H).

4-Chloro-3-(4-fluorophenyl)-6-(trifluoromethyl)isoxazolo[5,4*-d*]pyrimidine (**12h**). Synthesized according to general procedure E from compound **11h** (1.0 equiv., 14.6 mmol, 4.37 g) and excess POCl_3_ (∼218 mmol, 20 mL). The product was purified by column chromatography using EtOAc/*n*-hexane (1:1, v/v) as the mobile phase. Brown solid. Yield: 37%. Mp = 62–66 °C. Rf (EtOAc/*n*-hexane = 1:1, v/v) = 0.70. ^1^H NMR (400 MHz, DMSO*-d*_*6*_) δ (ppm) 8.30–8.36 (m, 2H), 7.43–7.50 (m, 2H).

3-(3-Bromo-4-fluorophenyl)-4-chloro-6-(trifluoromethyl)isoxazolo[5,4*-d*]pyrimidine (**12i**). Synthesized according to general procedure E from compound **11i** (1.0 equiv., 8.1 mmol, 3.07 g) and excess POCl_3_ (∼97 mmol, 15 mL). The product was purified by column chromatography using EtOAc/*n*-hexane (1:3, v/v) as the mobile phase. Yellow solid. Yield 43%. Mp = 96–99 °C. Rf (EtOAc/*n*-hexane = 1:3, v/v)= 0.59. ^1^H NMR (400 MHz, DMSO*-d*_*6*_) δ (ppm) 8.67 (dd, *J* = 6.7, 2.2 Hz, 1H), 8.32 (ddd, *J* = 8.7, 4.8, 2.2 Hz, 1H), 7.65 (t, *J* = 8.7 Hz, 1H).

4-Chloro-3-(3,4*-d*ifluorobenzyl)-6-(trifluoromethyl)isoxazolo[5,4*-d*]pyrimidine (**12j**). Synthesized according to general procedure E from compound **11j** (1.0 equiv., 13.6 mmol, 0.45 g) and excess POCl_3_ (∼54 mmol, 5 mL). The product was purified by column chromatography using CH_2_Cl_2_/MeOH (15:1, v/v) as the mobile phase. Yellow oil. Yield: 54%. Rf (CH_2_Cl_2_/MeOH = 9:1, v/v) = 0.85. ^1^H NMR (400 MHz, DMSO*-d*_*6*_) δ (ppm) 7.42 (ddt, *J* = 10.9, 8.9, 6.0 Hz, 2H), 7.28–7.09 (m, 1H), 4.58 (s, 1H).

4-Chloro-3-cyclopropyl-6-(trifluoromethyl) Isoxazolo[5,4*-d*]pyrimidine (**12k**). Synthesized according to general procedure E from compound **11k** (1.0 equiv., 3.7 mmol, 0.91 g) and excess POCl_3_ (∼54 mmol, 5 mL). The product was purified by column chromatography using EtOAc/*n*-hexane (1:1, v/v) as the mobile phase. Waxy yellow solid. Yield: 54%. Rf (EtOAc/*n*-hexane = 2:1, v/v) = 0.90. ^1^H NMR (400 MHz, DMSO*-d*_*6*_) δ (ppm) 1.38–1.23 (m, 2H), 1.19–1.09 (m, 3H).

*tert*-Butyl 4-(4-((Methylsulfonyl)oxy)-6-(trifluoromethyl)isoxazolo[5,4*-d*]pyrimidin-3-yl)piperidine-1-carboxylate (**12l**). To the solution of compound **11l** (1.0 equiv., 1.2 mmol, 0.50 g) in CH_2_Cl_2_, DMAP (0.5 equiv., 0.6 mmol, 0.008 g) and DIPEA (2.0 equiv., 2.4 mmol, 0.437 mL) were added. The reaction mixture was cooled to 0 °C, and mesyl chloride (1.5 equiv., 1.8 mmol, 0.15 mL) was added dropwise. The mixture was allowed to warm to room temperature overnight. The next day, H_2_O was added and the layers were separated. The organic phase was dried over anhydrous Na_2_SO_4_ and filtered, and the solvent was removed under reduced pressure. The product was purified by column chromatography using EtOAc/*n*-hexane (1:1, v/v) as the mobile phase. Yellow oil. Yield 42%. Rf (EtOAc/*n*-hexane = 2:1, v/v) = 0.90. ^1^H NMR (400 MHz, DMSO*-d*_*6*_) δ (ppm) 4.05 (d, *J* = 9.1 Hz, 2H), 3.16 (s, 3H), 3.07–2.93 (m, 2H), 2.15–2.05 (m, 2H), 1.98–1.88 (m, 1H), 1.75–1.61 (m, 2H), 1.40 (s, 9H).

#### General Procedure F for the Synthesis of Final Compounds (**14a**–**25b**)

To a solution of 4-chloroisoxazolo[5,4*-d*]pyrimidines (**12a**–**l**) (1.0 equiv) in anhydrous MeCN, the appropriate amine (2.0 equiv) and K_2_CO_3_ (3.0 equiv) were added. The reaction mixture was stirred at 50 °C for 1 h and at room temperature overnight (16 h).^26^ MeCN was evaporated under reduced pressure. CH_2_Cl_2_ was added to the remaining oily residue, which was transferred to a separating funnel and washed with H_2_O and brine. The organic phase was dried over anhydrous Na_2_SO_4_ and filtered, and the solvent was removed under reduced pressure. The product was recrystallized from EtOAc/*n*-hexane or purified by column chromatography (EtOAc/*n*-hexane) to obtain the final compound.

4-(3-Methylpiperidin-1-yl)-3-phenyl-6-(trifluoromethyl)isoxazolo[5,4*-d*]pyrimidine (**14a**). Synthesized according to general procedure F from compound **12a** (1.0 equiv., 0.33 mmol, 0.10 g), K_2_CO_3_ (3.0 equiv., 1.00 mmol, 0.138 g), and 3-methylpiperidine (3.0 equiv., 1.00 mmol, 0.12 mL). The product was purified by recrystallization from Et_2_O and *n*-hexane. Light-orange solid. Yield: 47%. Mp = 111–113 °C. Rf (EtOAc/*n*-hexane = 1:9, v/v) = 0.31. ^1^H NMR (400 MHz, CDCl_3_) δ (ppm) 7.64–7.50 (m, 5H), 4.31–4.20 (m, 1H), 4.01–3.90 (m, 1H), 2.77 (td, *J* = 12.9, 3.1 Hz, 1H), 2.49–2.43 (m, 1H), 1.79–1.72 (m, 1H), 1.57–1.38 (m, 3H), 1.10–0.96 (m, 1H), 0.59 (d, *J* = 5.9 Hz, 3H). ^13^C NMR (100 MHz, CDCl_3_) δ (ppm) 177.30, 159.85, 157.64, 155.02 (q, *J*_C–F_ = 37.0 Hz), 130.64, 129.73, 129.21, 128.56, 119.41 (q, *J*_C–F_ = 276.1 Hz), 95.96, 56.17, 48.81, 32.36, 31.15, 25.04, 18.55. ESI-HRMS ([M + H]^+^, *m*/*z*): calcd for C_18_H_18_F_3_N_4_O 363.1427, found 363.1424. HPLC: *t*_R_ = 12.203 (method A, purity 96.49%).

*N*-Isobutyl-3-phenyl-6-(trifluoromethyl)isoxazolo[5,4*-d*]pyrimidin-4-amine (**14b**). Synthesized according to general procedure F from compound **12a** (1.0 equiv., 0.33 mmol, 0.100 g), K_2_CO_3_ (3.0 equiv., 1.00 mmol, 0.138 g), and isobutylamine (2.0 equiv., 0.67 mmol, 0.07 mL). The product was purified by recrystallization from Et_2_O and *n*-hexane. Light-brown solid. Yield: 51%. Mp = 106–108 °C. Rf (EtOAc/*n*-hexane = 1:9, v/v) = 0.10. ^1^H NMR (400 MHz, CDCl_3_) δ (ppm) 7.73–7.69 (m, 2H), 7.66–7.63 (m, 3H), 5.67 (t, *J* = 4.7 Hz, 1H), 3.48 (dd, *J* = 6.4, 6.0 Hz, 2H), 1.87 (m, 1H), 0.93 (d, *J* = 6.7 Hz, 6H). ^13^C NMR (100 MHz, acetone*-d*_6_) δ (ppm) 175.99, 158.85, 157.24, 156.94 (q, *J*_C–F_ = 36.9 Hz), 131.47, 130.11, 128.30, 128.21, 119.46 (q, *J*_C–F_ = 276.1 Hz), 95.88, 48.67, 28.29, 20.12. ESI-HRMS ([M + H]^+^, *m*/*z*): calcd for C_16_H_16_F_3_N_4_O 337.1271, found 337.1268. HPLC: *t*_R_ = 11.750 (method A, purity 95.74%).

3-(4-Chlorophenyl)-4-(3-methylpiperidin-1-yl)-6-(trifluoromethyl)isoxazolo[5,4*-d*]pyrimidine (**15a**). Synthesized according to general procedure F from compound **12b** (1.0 equiv., 0.45 mmol, 0.150 g), K_2_CO_3_ (3.0 equiv., 1.35 mmol, 0.186 g), and 3-methylpiperidine (3.0 equiv., 1.35 mmol, 0.158 mL). The product was purified by column chromatography, using EtOAc/*n*-hexane (1:4, v/v) as the mobile phase. White solid. Yield: 10%. Mp = 89–92 °C. Rf (EtOAc/*n*-hexane = 1:3, v/v) = 0.48. ^1^H NMR (400 MHz, acetone*-d*_6_) δ (ppm) 7.68–7.64 (m, 2H), 7.58–7.54 (m, 2H), 4.20–4.10 (m, 1H), 3.86 (d, *J* = 11.6 Hz, 1H), 2.84–2.77 (m, 1H), 2.55–2.49 (m, 1H), 1.67–1.60 (m, 1H), 1.50–1.41 (m, 2H), 1.38–1.33 (m, 1H), 1.06–0.96 (m, 1H), 0.50 (d, *J* = 6.6 Hz, 3H). ^13^C NMR (100 MHz, CDCl_3_) δ (ppm) 177.47, 159.92, 156.78, 155.17 (q, *J*_C–F_ = 37.1 Hz), 137.16, 129.97, 129.63, 128.20, 119.46 (q, *J*_C–F_ = 276.1 Hz), 95.86, 56.29, 49.25, 32.40, 31.33, 25.17, 18.64. ESI-HRMS ([M + H]^+^, *m*/*z*): calcd for C_18_H_16_ClF_3_N_4_O 397.0965, found 397.10352. HPLC: *t*_R_ = 7.943 (method B, purity 100%.

3-(4-Chlorophenyl)-*N*-isobutyl-6-(trifluoromethyl)isoxazolo[5,4*-d*]pyrimidin-4-amine (**15b**). Synthesized according to general procedure F from compound **12b** (1.0 equiv., 0.45 mmol, 0.150 g), K_2_CO_3_ (3.0 equiv., 1.35 mmol, 0.186 g), and isobutylamine (3.0 equiv., 1.35 mmol, 0.134 mL). The product was purified by column chromatography, using EtOAc/*n*-hexane (1:4, v/v) as the mobile phase. White solid. Yield: 10%. Mp = 146–149 °C. Rf (EtOAc/*n*-hexane = 1:4, v/v) = 0.42. ^1^H NMR (400 MHz, acetone*-d*_6_) δ (ppm) 7.87–7.82 (m, 2H), 7.73–7.67 (m, 2H), 7.08 (s, 1H), 3.52–3.45 (m, 2H), 2.22–2.19 (m, 1H), 0.96 (d, *J* = 6.7 Hz, 6H). ^13^C NMR (100 MHz, CDCl_3_) δ (ppm) 176.01, 158.64, 156.10, 146.99, 137.85, 130.31, 129.50, 126.52, 119.25 (q, *J*_C–F_ = 276.6 Hz), 95.57, 87.24, 48.63, 28.21, 20.03. ESI-HRMS ([M + H]^+^, *m*/*z*): calcd for C_16_H_14_ClF_3_N_4_O 371.0808, found 371.08778. HPLC: *t*_R_ = 7.600 (method B, purity 100%).

4-(3-Methylpiperidin-1-yl)-6-(trifluoromethyl)-3-(4-(trifluoromethyl)phenyl)isoxazolo[5,4*-d*]pyrimidine (**16a**). Synthesized according to general procedure F from compound **12c** (1.0 equiv., 0.41 mmol, 0.150 g), K_2_CO_3_ (3.0 equiv., 1.23 mmol, 0.169 g), and 3-methylpiperidine (3.0 equiv., 1.23 mmol, 0.144 mL). The product was purified by column chromatography, using Et_2_O/petroleum ether (1:10, v/v) as the mobile phase. Yellow solid. Yield: 50%. Mp = 92–95 °C. Rf (Et_2_O/petroleum ether = 1:10, v/v) = 0.38. ^1^H NMR (400 MHz, DMSO*-d*_*6*_) δ (ppm) 8.16–7.75 (m, 4H), 4.30–4.20 (m, 1H), 3.69 (s, 1H), 2.88–2.79 (m, 1H), 2.58–2.53 (m, 1H), 1.69–1.64 (m, 1H), 1.58–1.54 (m, 1H), 1.42–1.35 (m, 2H), 1.09–1.02 (m, 1H), 0.44 (s, 3H). ^13^C NMR (100 MHz, DMSO*-d*_*6*_) δ (ppm) 176.52, 158.93, 157.00, 153.54 (q, *J*_C–F_ = 36.2 Hz), 133.50, 130.86 (q, *J*_C–F_ = 32.2 Hz), 129.80, 126.03 (q, *J*_C–F_ = 3.7 Hz), 123.84 (q, *J*_C–F_ = 272.5 Hz), 119.31 (q, *J* = 275.9 Hz), 95.99, 55.37, 47.69, 31.52, 30.44, 24.39, 17.96. ESI-HRMS ([M + H]^+^, *m*/*z*): calcd for C_19_H_16_F_6_N_4_O 431.1301, found 431.1302. HPLC: *t*_R_ = 5.390 (method C, purity 95.46%).

*N*-Isobutyl-6-(trifluoromethyl)-3-(4-(trifluoromethyl)phenyl)isoxazolo[5,4*-d*]pyrimidin-4-amine (**16b**). Synthesized according to general procedure F from compound **12c** (1.0 equiv., 0.46 mmol, 0.170 g), K_2_CO_3_ (3.0 equiv., 1.38 mmol, 0.192 g), and isobutylamine (3.0 equiv., 1.38 mmol, 0.138 mL). The product was purified by column chromatography, using Et_2_O/petroleum ether (1:10, v/v) as the mobile phase. Yellow solid. Yield: 22%. Mp = 122–125 °C. Rf (Et_2_O/petroleum ether = 1:10, v/v) = 0.10. ^1^H NMR (400 MHz, DMSO*-d*_*6*_) δ (ppm) 8.02–7.95 (m, 4H), 7.75 (t, *J* = 5.8 Hz, 1H), 2.02–1.92 (m, 1H), 1.43–1.21 (m, 1H), 1.02–1.92 (m, 1H), 0.92 (d, *J* = 6.8 Hz, 6H). ^13^C NMR (100 MHz, DMSO*-d*_*6*_) δ (ppm) 175.45, 158.25, 156.82, 154.96 (q, *J*_C–F_ = 36.0 Hz), 131.50, 130.93 (q, *J*_C–F_ = 32.2 Hz), 129.45, 126.31 (q, *J*_C–F_ = 3.8 Hz), 124.00 (q, *J*_C–F_ = 272.5 Hz), 119.35 (q, *J*_C–F_ = 275.9 Hz), 95.31, 48.22, 27.73, 20.06. ESI-HRMS ([M + H]^+^, *m*/*z*): calcd for C_17_H_14_F_6_N_4_O 405.1072, found 405.1139. HPLC: *t*_R_ = 5.250 (method C, purity 95.49%).

4-(4-(3-Methylpiperidin-1-yl)-6-(trifluoromethyl)isoxazolo[5,4*-d*]pyrimidin-3-yl)benzonitrile (**17**). Synthesized according to general procedure F from compound **12d** (1.0 equiv., 0.184 mmol, 0.060 g), K_2_CO_3_ (3.0 equiv., 0.552 mmol, 0.077 g), and 3-methylpiperidine (2.0 equiv., 0.368 mmol, 0.043 mL). The product was purified by recrystallization from Et_2_O. White solid. Yield: 53%. Mp = 147–149 °C. Rf (EtOAc/*n*-hexane = 1:1, v/v) = 0.50. ^1^H NMR (400 MHz, DMSO*-d*_*6*_) δ (ppm) 8.14–8.09 (m, 2H), 7.95–7.90 (m, 2H), 4.25–4.02 (m, 1H), 3.82–3.60 (m, 1H), 2.88–2.79 (m, 1H), 2.62–2.52 (m, 1H), 1.66 (d, *J* = 13.0 Hz, 1H), 1.56–1.23 (m, 3H), 1.09–1.01 (m, 1H), 0.50 (bs, 3H). ^13^C NMR (100 MHz, DMSO*-d*_*6*_) δ (ppm) 176.64, 158.96, 157.02, 155.02 (q, *J*_C–F_ = 37.0 Hz), 133.64, 133.15, 129.92, 119.43 (q, *J*_C–F_ = 276.0 Hz), 118.29, 113.39, 96.03, 55.33, 48.08, 31.56, 30.77, 24.47, 18.24. ESI-HRMS ([M + H]^+^, *m*/*z*): calcd for C_19_H_17_F_3_N_5_O 388.1380, found 388.1367. HPLC: *t*_R_ = 4.830 (method C, purity 96.41%).

4-(3-Methylpiperidin-1-yl)-3-(4-nitrophenyl)-6-(trifluoromethyl)isoxazolo[5,4*-d*]pyrimidine (**18**). Synthesized according to general procedure F from compound **12e** (1.0 equiv., 0.23 mmol, 0.150 g), K_2_CO_3_ (3.0 equiv., 0.69 mmol, 0.94 g), and 3-methylpiperidine (3.0 equiv., 0.69 mmol, 0.80 mL). The product was purified by column chromatography, using EtOAc/*n*-hexane (1:3, v/v) as the mobile phase. White waxy solid. Yield: 10%. Rf (EtOAc/*n*-hexane = 1:2, v/v) = 0.52. ^1^H NMR (400 MHz, CDCl_3_) δ (ppm) 8.46–8.43 (m, 2H), 7.87–7.84 (m, 2H), 4.19–4.05 (m, 1H), 4.00–3.87 (m, 1H), 2.88–2.81 (m, 1H), 2.55 (dd, *J* = 13.1, 10.9 Hz, 1H), 1.81–1.78 (m, 1H), 1.63–1.60 (m, 1H), 1.47–1.40 (m, 1H), 1.28–1.24 (m, 1H), 1.15–1.04 (m, 1H), 0.66 (d, *J* = 6.6 Hz, 3H). ^13^C NMR (100 MHz, CDCl_3_) δ (ppm) 177.67, 159.76, 155.81, 155.75 (m), 149.14, 135.78, 129.69, 124.33, 118.69 (m), 95.60, 56.28, 49.46, 32.18, 31.22, 25.04, 18.60. ESI-HRMS ([M + H]^+^, *m*/*z*): calcd for C_18_H_16_F_3_N_5_O_3_ 408.1205, found 408.1274. HPLC: *t*_R_ = 7.287 (method B, purity 99.26%).

4-(3-Methylpiperidin-1-yl)-3-(3-nitrophenyl)-6-(trifluoromethyl)isoxazolo[5,4*-d*]pyrimidine (**19a**). Synthesized according to general procedure F from compound **12f** (1.0 equiv., 0.58 mmol, 0.200 g), K_2_CO_3_ (3.0 equiv., 1.74 mmol, 0.241 g), and 3-methylpiperidine (3.0 equiv., 1.74 mmol, 0.204 mL). The product was purified by column chromatography, using EtOAc/*n*-hexane (1:4, v/v) as the mobile phase. White waxy solid. Yield: 52%. Rf (EtOAc/*n*-hexane = 1:4, v/v) = 0.20. ^1^H NMR (400 MHz, DMSO*-d*_*6*_) δ (ppm) 8.58–8.56 (m, 1H), 8.49 (ddd, *J* = 8.3, 2.4, 1.0 Hz, 1H), 8.20–8.18 (m, 1H), 7.94 (t, *J* = 8.0 Hz, 1H), 4.13 (s, 1H), 3.79 (s, 1H), 2.89–2.82 (m, 1H), 2.62–2.56 (m, 1H), 1.67–1.63 (m, 1H), 1.54–1.50 (m, 1H), 1.40–1.30 (m, 2H), 1.09–0.99 (m, 1H), 0.50 (d, *J* = 6.4 Hz, 3H). ^13^C NMR (100 MHz, CDCl_3_) δ (ppm) 177.73, 159.99, 155.78, 155.45 (q, *J*_C–F_= 37.3 Hz), 148.58, 134.34, 131.41, 130.68, 125.47, 123.86, 119.40 (q, *J*_C–F_ = 276.0 Hz), 95.71, 56.42, 49.59, 32.30, 31.22, 25.08, 18.65. ESI-HRMS ([M + H]^+^, *m*/*z*): calcd for C_18_H_16_F_3_N_5_O_3_ 408.1205, found 408.1276. HPLC: *t*_R_ = 7.280 (method B, purity 100%).

*N*-Isobutyl-3-(3-nitrophenyl)-6-(trifluoromethyl)isoxazolo[5,4*-d*]pyrimidin-4-amine (**19b**). Synthesized according to general procedure F from compound **12f** (1.0 equiv., 0.35 mmol, 0.120 g), K_2_CO_3_ (3.0 equiv., 1.05 mmol, 0.144 g), and isobutylamine (3.0 equiv., 1.05 mmol, 0.104 mL). The product was purified by column chromatography, using EtOAc/*n*-hexane (1:2, v/v) as the mobile phase. White solid. Yield: 11%. Mp = 133–135 °C. Rf (EtOAc/*n*-hexane = 1:2, v/v) = 0.30. ^1^H NMR (400 MHz, DMSO*-d*_*6*_) δ (ppm) 8.59–8.48 (m, 2H), 8.21 (dd, *J* = 7.8, 1.3 Hz, 1H), 7.93 (t, *J* = 8.0 Hz, 1H), 7.84 (s, 1H), 1.95 (m, 1H), 0.92 (d, *J* = 6.7 Hz, 6H), 2H overlapping with water signal. ^13^C NMR (100 MHz, CDCl_3_) δ (ppm) 176.34, 171.34, 158.56, 157.12 (q, *J*_C–F_= 37.1 Hz), 155.13, 148.97, 134.08, 131.37, 130.00, 125.97, 123.04, 119.20 (q, *J*_C–F_ = 276.2 Hz), 95.35, 48.91, 28.21, 20.00. ESI-HRMS ([M + H]^+^, *m*/*z*): calcd for C_16_H_14_F_3_N_5_O_3_ 382.1049, found 382.0937. HPLC: *t*_R_ = 7.097 (method B, purity 100%).

3-(Benzo[*d*][1,3]dioxol-5-yl)-4-(3-methylpiperidin-1-yl)-6-(trifluoromethyl)isoxazolo[5,4*-d*]pyrimidine (**20a**). Synthesized according to general procedure F from compound **12g** (1.0 equiv., 0.54 mmol, 0.200 g), K_2_CO_3_ (3.0 equiv., 1.74 mmol, 0.241 g), and 3-methylpiperidine (3.0 equiv., 1.74 mmol, 0.205 mL). The product was purified by column chromatography, using EtOAc/*n*-hexane (1:4, v/v) as the mobile phase. Colorless oil. Yield: 8%. Rf (EtOAc/*n*-hexane = 1:4, v/v) = 0,26. ^1^H NMR (400 MHz, CDCl_3_) δ (ppm) 7.11–7.06 (m, 2H), 6.97 (d, *J* = 7.9 Hz, 1H), 6.09 (s, 2H), 4.30–4.20 (m, 1H), 4.15–3.97 (m, 1H), 2.88–2.81 (m, 1H), 2.54 (dd, *J* = 12.9, 11.1 Hz, 1H), 1.82–1.78 (m, 1H), 1.67–1.63 (m, 2H), 1.59–1.40 (m, 1H), 1.12–1.08 (m, 1H), 0.70 (d, *J* = 6.5 Hz, 3H). ^13^C NMR (100 MHz, CDCl_3_) δ (ppm) 177.37, 160.09, 157.35, 155.07 (d, *J*_C–F_= 37.1 Hz), 149.81, 148.45, 123.16, 123.11, 119.53 (q, *J*_C–F_ = 276.0 Hz), 109.16, 108.80, 101.97, 96.03, 56.31, 49.27, 32.53, 31.34, 25.23, 18.80. ESI-HRMS ([M + H]^+^, *m*/*z*): calcd for C_18_H_16_F_3_N_5_O_3_ 408.1205, found 408.1274. HPLC: *t*_R_ = 7.367 (method B, purity 98.78%).

3-(Benzo[*d*][1,3]dioxol-5-yl)-*N*-isobutyl-6-(trifluoromethyl)isoxazolo[5,4*-d*]pyrimidin-4-amine (**20b**). Synthesized according to general procedure F from compound **12g** (1.0 equiv., 0.54 mmol, 0.200 g), K_2_CO_3_ (3.0 equiv., 1.74 mmol, 0.241 g), and isobutylamine (3.0 equiv., 1.74 mmol, 0.191 mL). The product was purified by column chromatography, using EtOAc/*n*-hexane (1:4, v/v) as the mobile phase. White solid. Yield: 12%. Mp = 142–145 °C. Rf (EtOAc/*n*-hexane = 1:4, v/v) = 0.18. ^1^H NMR (400 MHz, CDCl_3_) δ (ppm) 7.16–7.14 (m, 2H), 7.03–7.01 (m, 1H), 6.12 (s, 2H), 5.82 (t, *J* = 5.8 Hz, 1H), 3.51–3.48 (m, 2H), 1.97–1.87 (m, 1H), 0.97 (d, *J* = 6.7 Hz, 6H). ^13^C NMR (100 MHz, CDCl_3_) δ (ppm) 175.97, 158.85, 156.90 (m), 150.42, 149.36, 122.38, 121.68, 119.46 (q, *J*_C–F_ = 276.0 Hz), 109.63, 108.61, 102.27, 95.78, 48.70, 28.36, 20.19. ESI-HRMS ([M + H]^+^, *m*/*z*): calcd for C_17_H_15_F_3_N_4_O_3_ 381.1096, found 381.1165. HPLC: *t*_R_ = 7.130 (method B, purity 100%).

3-(4-Fluorophenyl)-4-(3-methylpiperidin-1-yl)-6-(trifluoromethyl)isoxazolo[5,4*-d*]pyrimidine (**21a**). Synthesized according to general procedure F from compound **12h** (1.0 equiv., 0.35 mmol, 0.112 g), K_2_CO_3_ (3.0 equiv., 1.06 mmol, 0.140 g), and 3-methylpiperidine (3.0 equiv., 1.06 mmol, 0.123 mL). The product was purified by column chromatography, using EtOAc/*n*-hexane (1:4, v/v) as the mobile phase. White solid. Yield: 84%. Rf (EtOAc/*n*-hexane = 1/3, v/v) = 0.45. ^1^H NMR **(**400 MHz, CDCl_3_) δ (ppm) 7.67–7.55 (m, 2H), 7.34–7.23 (m, 2H), 4.21 (s, 1H), 4.05–3.89 (m, 1H), 2.81 (ddd, *J* = 13.3, 12.2, 3.1 Hz, 1H), 2.50 (dd, *J* = 13.1, 11.0 Hz, 1H), 1.86–1.72 (m, 1H), 1.62 (dt, *J* = 13.9, 3.4 Hz, 1H), 1.45 (dtt, *J* = 29.7, 12.5, 4.0 Hz, 1H), 1.08 (tdd, *J* = 12.7, 11.2, 3.9 Hz, 1H), 0.66 (d, *J* = 6.6 Hz, 3H). ^13^C NMR (100 MHz, CDCl_3_) δ (ppm) 177.37, 164.13 (d, *J*_C–F_ = 251.8 Hz), 159.91, 156.71, 155.12 (q, *J*_C–F_ = 37.1 Hz) 130.63 (d, *J*_C–F_ = 8.6 Hz), 125.83, 125.80, 119.37 (q, *J*_C–F_ = 276.0 Hz), 116.51 (d, *J*_C–F_ = 22.0 Hz), 95.92, 56.23, 49.08, 32.33, 31.23, 25.07, 18.61. ESI-HRMS ([M + H]^+^, *m*/*z*): calcd for C_18_H_17_ON_4_F_4_ 381.1333, found 381.1328. HPLC: *t*_R_ = 7.523 (method B, purity 100%).

*N*-Cyclopropyl-3-(4-fluorophenyl)-6-(trifluoromethyl)isoxazolo[5,4*-d*]pyrimidin-4-amine (**21b**). Synthesized according to general procedure F from compound **12h** (1.0 equiv., 0.39 mmol, 0.125 g), K_2_CO_3_ (3.0 equiv., 1.18 mmol, 0.163 g), and isobutylamine (3.0 equiv., 1.18 mmol, 0.082 mL). The product was purified by column chromatography, using EtOAc/*n*-hexane (1:3, v/v) as the mobile phase. Colorless oil. Yield: 41%. Rf (EtOAc/*n*-hexane = 1:3, v/v) = 0.40. ^1^H NMR (400 MHz, CDCl_3_) δ (ppm) 7.82–7.59 (m, 2H), 7.43–7.30 (m, 2H), 5.66 (s, 1H), 3.04 (tt, *J* = 7.3, 3.7 Hz, 1H), 1.08–0.83 (m, 2H), 0.66–0.46 (m, 2H). ^13^C NMR (100 MHz, CDCl_3_) δ (ppm) 175.83, 164.47 (d, *J*_C–F_ = 253.2 Hz), 159.66, 156.88 (q, *J*_C–F_ = 37.1 Hz), 156.12, 130.32 (d, *J*_C–F_ = 8.7 Hz), 124.08, 119.30 (q, *J*_C–F_ = 276.0 Hz), 117.32 (d, *J*_C–F_ = 22.3 Hz), 95.95, 24.60, 7.54. ESI-HRMS ([M + H]^+^, *m*/*z*): calcd for C_15_H_11_ON_4_F_4_ 339.0863, found 339.0861. HPLC: *t*_R_ = 5.163 (method C, purity 99.81%).

3-(4-Fluorophenyl)-*N*-isobutyl-6-(trifluoromethyl)isoxazolo[5,4*-d*]pyrimidin-4-amine (**21c**). Synthesized according to general procedure F from compound **12h** (1.0 equiv., 0.39 mmol, 0.125 g), K_2_CO_3_ (3.0 equiv., 1.18 mmol, 0.168 g), and cyclopropylamine (3.0 equiv., 1.18 mmol, 0.121 mL). The product was purified by column chromatography, using EtOAc/*n*-hexane (1:5, v/v) as the mobile phase. Colorless oil. Yield: 57%. Rf (EtOAc/*n*-hexane = 1:3, v/v) = 0.40. ^1^H NMR (400 MHz, CDCl_3_) δ (ppm) 7.78–7.63 (m, 2H), 7.50–7.27 (m, 2H), 5.59 (s, 1H), 3.49 (dd, *J* = 6.7, 5.8 Hz, 2H), 1.90 (dp, *J* = 13.4, 6.7 Hz, 1H), 0.95 (d, *J* = 6.7 Hz, 6H). ^13^C NMR (100 MHz, CDCl_3_) δ (ppm) 175.96, 164.45 (d, *J*_C–F_ = 253.3 Hz), 158.71, 156.91 (q, *J*_C–F_ = 37.1 Hz), 156.20, 130.35 (d, *J*_C–F_ = 8.7 Hz), 124.20 (d, *J*_C–F_ = 3.6 Hz), 119.29 (q, *J*_C–F_ = 276.0 Hz), 117.33 (d, *J*_C–F_ = 22.1 Hz), 95.68, 48.61, 28.21, 20.03. ESI-HRMS ([M + H]^+^, *m*/*z*): calcd for C_16_H_15_ON_4_F_4_ 355.1176, found 355.1173. HPLC: *t*_R_ = 4.820 (method C, purity 99.26%).

3-(4-Fluorophenyl)-4-(piperidin-1-yl)-6-(trifluoromethyl)isoxazolo[5,4*-d*]pyrimidine (**21d**). Synthesized according to general procedure F from compound **12h** (1.0 equiv., 0.5 mmol, 0.16 g), K_2_CO_3_ (3.0 equiv., 1.5 mmol, 0.21 g), and piperidine (3.0 equiv., 1.5 mmol, 0.148 mL). The product was purified by column chromatography, using EtOAc/*n*-hexane (1:3, v/v) as the mobile phase. Yellow solid. Yield: 24%. Mp = 142–145 °C. Rf (EtOAc/*n*-hexane = 1:3, v/v) = 0.40. ^1^H NMR (400 MHz, CDCl_3_) δ (ppm) 7.61–7.66 (m, 2H), 7.23–7.29 (m, 2H), 3.51 (d, *J* = 5.0 Hz, 4H), 1.60 (dt, *J* = 11.3, 5.7 Hz, 2H), 1.45–1.51 (m, 4H). ^13^C NMR (100 MHz, CDCl_3_) δ (ppm) 177.39, 164.09 (d, *J*_C–F_ = 251.9 Hz), 160.00, 156.67, 155.10 (d, *J*_C–F_ = 37.2 Hz), 130.61 (d, *J*_C–F_= 8.4 Hz), 125.65 (d, *J*_C–F_ = 3.4 Hz), 119.38 (d, *J*_C–F_ = 276.0 Hz), 116.50 (d, *J*_C–F_ = 22.0 Hz), 95.93, 50.01, 25.67, 23.80. ESI-HRMS ([M + H]^+^, *m*/*z*): calcd for C_17_H_15_F_4_N_4_O 367.1176, found 367.1174. HPLC: *t*_R_ = 7.267 (method B, purity 100%).

3-(4-Fluorophenyl)-4-(2-methylpiperidin-1-yl)-6-(trifluoromethyl)isoxazolo[5,4*-d*]pyrimidine (**21e**). Synthesized according to general procedure F from compound **12h** (1.0 equiv., 0.5 mmol, 0.16 g), K_2_CO_3_ (3.0 equiv., 1.5 mmol, 0.21 g), and 2-methylpiperidine (3.0 equiv., 1.5 mmol, 0.176 mL). The product was purified by column chromatography, using EtOAc/*n*-hexane (1:1, v/v) as the mobile phase. Yellow oil. Yield: 8%. Rf (EtOAc/*n*-hexane = 1:1, v/v) = 0.60. ^1^H NMR (400 MHz, DMSO*-d*_*6*_) δ (ppm) 8.23–8.30 (m, 2H), 7.87–7.95 (m, 2H), 5.34 (bs, 1H), 4.25 (bs, 1H), 3.56 (ddd, *J* = 13.7, 12.4, 3.7 Hz, 1H), 2.12–2.22 (m, 2H), 1.92–2.05 (m, 2H), 1.77–1.92 (m, 2H), 1.67 (d, *J* = 4.8 Hz, 3H). ^13^C NMR (100 MHz, acetone*-d*_*6*_) δ (ppm) 177.22, 163.97 (d, *J*_C–F_ = 248.8 Hz), 159.97, 157.30, 154.28 (q, *J*_C–F_ = 36.7 Hz), 131.10 (d, *J*_C–F_ = 8.7 Hz), 126.39 (d, *J*_C–F_ = 3.4 Hz), 119.74 (q, *J*_C–F_ = 275.1 Hz), 116.24 (d, *J*_C–F_ = 22.4 Hz), 96.37, 50.36, 44.58, 29.42, 25.41, 17.91, 14.59. ESI-HRMS ([M + H]^+^, *m*/*z*): calcd for C_18_H_17_F_4_N_4_O 381.1333, found 381.1330. HPLC: *t*_R_ = 7.503 (method B, purity 98.28%).

3-(4-Fluorophenyl)-4-(4-methylpiperidin-1-yl)-6-(trifluoromethyl)isoxazolo[5,4*-d*]pyrimidine (**21f**). Synthesized according to general procedure F from compound **12h** (1.0 equiv., 0.5 mmol, 0.16 g), K_2_CO_3_ (3.0 equiv., 1.5 mmol, 0.21 g), and 4-methylpiperidine (3.0 equiv., 1.5 mmol, 0.176 mL). The product was purified by column chromatography, using EtOAc/*n*-hexane (1:1, v/v) as the mobile phase. Yellow solid. Yield: 20%. Mp = 137–139 °C. Rf (EtOAc/*n*-hexane = 1:1, v/v) = 0.54. ^1^H NMR (400 MHz, DMSO*-d*_*6*_) δ (ppm) 8.25–8.33 (m, 2H), 7.84–7.93 (m, 2H), 4.68 (d, *J* = 13.2 Hz, 2H), 3.47 (ddd, *J* = 13.3, 12.4, 2.8 Hz, 2H), 2.06–2.17 (m, 1H), 1.97–2.06 (m, 2H), 1.51–1.65 (m, 2H), 1.34 (d, *J* = 6.5 Hz, 3H). ^13^C NMR (100 MHz, acetone*-d*_*6*_) δ (ppm) 177.27, 164.02 (d, *J*_C–F_ = 249.1 Hz), 159.95, 157.25, 154.31 (q, *J*_C–F_ = 36.6 Hz), 131.17 (d, *J*_C–F_ = 8.8 Hz), 126.21 (d, *J*_C–F_ = 3.2 Hz), 119.72 (q, *J*_C–F_ = 275.2 Hz), 116.22 (d, *J*_C–F_ = 22.2 Hz), 96.21, 48.95, 33.57, 30.14, 20.82. ESI-HRMS ([M + H]^+^, *m*/*z*): calcd for C_18_H_17_F_4_N_4_O 381.1333, found 381.1331. HPLC: *t*_R_ = 7.563 (method B, purity 99.48%).

3-(4-Fluorophenyl)-4-(2-methylmorpholino)-6-(trifluoromethyl)isoxazolo[5,4*-d*]pyrimidine (**21g**). Synthesized according to general procedure F from compound **12h** (1.0 equiv., 0.5 mmol, 0.16 g), K_2_CO_3_ (3.0 equiv., 1.5 mmol, 0.21 g), and 2-methylmorpholine (3.0 equiv., 1.5 mmol, 0.16 mL). The product was purified by column chromatography, using EtOAc/*n*-hexane (1:5, v/v) as the mobile phase. Yellow oil. Yield: 56%. Rf (EtOAc/*n*-hexane = 1:5, v/v) = 0.30. ^1^H NMR (400 MHz, CDCl_3_) δ (ppm) 7.68–7.60 (m, 2H), 7.37–7.26 (m, 2H), 4.07 (d, *J* = 10.0 Hz, 1H), 3.95 (d, *J* = 10.8 Hz, 1H), 3.81 (dd, *J* = 11.8, 2.3 Hz, 1H), 3.52 (td, *J* = 12.0, 2.6 Hz, 1H), 3.48–3.42 (m, 1H), 3.07 (ddd, *J* = 13.4, 12.1, 3.5 Hz, 1H), 2.71 (dd, *J* = 13.2, 10.5 Hz, 1H), 0.97 (d, *J* = 6.2 Hz, 3H). ^13^C NMR (100 MHz, CDCl_3_) δ (ppm) 177.30, 164.33 (d, *J*_C–F_ = 252.7 Hz), 160.10, 156.53, 155.18 (q, *J*_C–F_ = 37.3 Hz), 130.84 (d, *J*_C–F_ = 8.6 Hz), 125.57 (d, *J*_C–F_ = 3.5 Hz), 119.33 (q, *J*_C–F_ = 276.1 Hz), 116.95, 116.77 (d, *J* = 22.1 Hz), 96.43, 71.65, 66.09, 54.61, 48.18. ESI-HRMS ([M + H]^+^, *m*/*z*): calcd for C_17_H_15_F_4_N_4_O_2_ 383.1125, found 383.1122. HPLC: *t*_R_ = 4.783 (method C, purity 97.92%).

3-(4-Fluorophenyl)-4-(3-methylpiperazin-1-yl)-6-(trifluoromethyl)isoxazolo[5,4*-d*]pyrimidine (**21h**). Synthesized according to general procedure F from compound **12h** (1.0 equiv., 0.5 mmol, 0.16 g), K_2_CO_3_ (3.0 equiv., 1.5 mmol, 0.21 g), and 2-methylpiperazine (3.0 equiv., 1.5 mmol, 0.15 g). The product was purified by column chromatography, using EtOAc as the mobile phase. Yellow solid. Yield: 5%. Mp = 96–99 °C. Rf (EtOAc)= 0.12. ^1^H NMR (400 MHz, CDCl_3_) δ (ppm) 7.57–7.66 (m, 2H), 7.22–7.30 (m, 2H), 4.16 (s, 1H), 3.96 (s, 1H), 2.86–2.97 (m, 2H), 2.70–2.78 (m, 1H), 2.62–2.70 (m, 1H), 2.55 (dd, *J* = 12.8, 10.4 Hz, 1H), 1.26 (s, 1H), 0.82 (d, *J* = 6.2 Hz, 3H). ^13^C NMR (100 MHz, CDCl_3_) δ (ppm) 177.29, 164.17 (d, *J*_C–F_ = 252.4 Hz), 159.95, 156.57, 155.06 (d, *J*_C–F_ = 37.3 Hz), 130.73 (d, *J*_C–F_ = 8.7 Hz), 125.83 (d, *J*_C–F_ = 3.6 Hz), 119.29 (q, *J*_C–F_ = 276.0 Hz), 116.59 (d, *J* = 22.2 Hz), 96.12, 55.76, 50.59, 48.83, 45.48, 18.99. ESI-HRMS ([M + H]^+^, *m*/*z*): calcd for C_17_H_16_F_4_N_5_O 382.1285, found 382.1280. HPLC: *t*_R_ = 4.240 (method B, purity 100%).

4-(3-(4-Fluorophenyl)-6-(trifluoromethyl)isoxazolo[5,4*-d*]pyrimidin-4-yl)piperazin-1-ium chloride (**21i**). Synthesized according to general procedure F from compound **12h** (1.0 equiv., 1 mmol, 0.32 g), K_2_CO_3_ (3.0 equiv., 3 mmol, 0.42 g), and *N*-Boc-piperazine (3.0 equiv., 1.5 mmol, 0.56 g). The product was purified by column chromatography, using EtOAc/*n*-hexane (1:1, v/v) as the mobile phase. Orange solid. Yield: 60%. Mp = 174–177 °C. Rf (EtOAc/*n*-hexane= 1/2, v/v) = 0.64. ^1^H NMR (400 MHz, CDCl_3_) δ (ppm) 7.63–7.68 (m, 2H), 7.26–7.31 (m, 2H), 3.46–3.64 (m, 4H), 3.33–3.38 (m, 4H), 1.44 (s, 9H). The Boc-protected amine (1.0 equiv) was dissolved in anhydrous dioxane (5 mL), and 4 M HCl in dioxane (10 mL) was added. The mixture was stirred at RT for 16 h, concentrated under high vacuum, and washed with Et_2_O (3 × 10 mL). Light-yellow solid. Yield: 64%. Mp > 200 °C. ^1^H NMR (400 MHz, DMSO*-d*_*6*_) δ (ppm) 9.48 (s, 2H), 7.78–7.85 (m, 2H), 7.46–7.53 (m, 2H), 3.71 (t, *J* = 5.3 Hz, 4H), 3.04 (t, *J* = 5.3 Hz, 4H). ^13^C NMR (100 MHz, DMSO*-d*_*6*_) δ (ppm) 176.83, 164.05 (d, *J*_C–F_ = 249.1 Hz), 160.63, 157.33, 153.79 (d, *J*_C–F_ = 36.7 Hz), 131.71 (d, *J*_C–F_ = 8.8 Hz), 125.39 (d, *J*_C–F_ = 3.4 Hz), 119.75 (d, *J*_C–F_ = 275.8 Hz), 117.00 (d, *J*_C–F_ = 22.1 Hz), 97.70, 45.51, 42.12. ESI-HRMS ([M + H]^+^, *m*/*z*): calcd for C_16_H_14_F_4_N_5_O 368.1129, found 368.1125. HPLC: *t*_R_ = 4.143 (method B, purity 97.44%.

3-(4-Fluorophenyl)-4-(4-methylpiperazin-1-yl)-6-(trifluoromethyl)isoxazolo[5,4*-d*]pyrimidine (**21j**). Synthesized according to general procedure F from compound **12h** (1.0 equiv., 0.5 mmol, 0.16 g), K_2_CO_3_ (3.0 equiv., 1.5 mmol, 0.21 g), and *N*-methylpiperazine (3.0 equiv., 1.5 mmol, 0.166 mL). The product was purified by column chromatography, using EtOAc as the mobile phase. Yellow solid. Yield: 9%. Mp = 115–118 °C. Rf (EtOAc) = 0.46. ^1^H NMR (400 MHz, DMSO*-d*_*6*_) δ (ppm) 7.86–7.91 (m, 2H), 7.42–7.48 (m, 2H), 3.44–3.56 (m, 4H), 2.22 (t, *J* = 5.0 Hz, 4H), 2.12 (s, 3H). ^13^C NMR (100 MHz, DMSO*-d*_*6*_) δ (ppm) 176.89, 163.90 (d, *J*_C–F_ = 249.9 Hz), 159.94, 157.50, 153.86 (q, *J*_C–F_ = 36.7 Hz), 131.73 (d, *J*_C–F_ = 8.8 Hz), 125.90 (d, *J*_C–F_ = 3.7 Hz), 119.78 (q, *J*_C–F_ = 275.8 Hz), 116.79 (d, *J*_C–F_ = 22.0 Hz), 96.87, 54.18, 48.39, 45.69. ESI-HRMS ([M + H]^+^, *m*/*z*): calcd for C_17_H_16_F_4_N_5_O 382.1285, found 382.1280. HPLC: *t*_R_ = 4.237 (method B, purity 97.93%).

3-(4-Fluorophenyl)-6-(trifluoromethyl)-4-(3-(trifluoromethyl)piperidin-1-yl)isoxazolo[5,4*-d*]pyrimidine (**21k**). Synthesized according to general procedure F from compound **12h** (1.0 equiv., 0.34 mmol, 0.110 g), K_2_CO_3_ (3.0 equiv., 1.04 mmol, 0.144 g), and 3-(trifluoromethyl)piperidine (1.18 mmol, 0.154 mL). Product was purified by precrystallization from EtOAc/*n*-hexane= 1/1. Colorless oil. Yield: 28%. Rf (EtOAc/*n*-hexane = 1:4, v/v) = 0.40. ^1^H NMR (400 MHz, CDCl_3_) δ (ppm) 7.71–7.59 (m, 2H), 7.35–7.26 (m, 2H), 4.44 (d, *J* = 13.2 Hz, 1H), 3.97 (d, *J* = 13.6 Hz, 1H), 3.06–2.78 (m, 2H), 2.30 (dtt, *J* = 15.2, 7.7, 3.8 Hz, 1H), 2.02 (dd, *J* = 15.2, 3.4 Hz, 1H), 1.69 (d, *J* = 13.7 Hz, 1H), 1.56–1.48 (m, 1H), 1.40 (ddt, *J* = 16.7, 12.5, 6.1 Hz, 1H). ^13^C NMR (100 MHz, CDCl_3_) δ (ppm) 177.33, 164.34 (q, *J*_C–F_ = 252.6 Hz), 160.64, 156.46, 155.28 (q, *J*_C–F_ = 37.5 Hz), 130.59 (d, *J*_C–F_ = 8.7 Hz), 125.85 (q, *J*_C–F_ = 279.3 Hz), 125.07 (d, *J*_C–F_ = 3.6 Hz), 119.21 (q, *J*_C–F_ = 276.1 Hz), 116.73 (d, *J*_C–F_ = 22.2 Hz), 96.88, 50.02, 47.24, 39.80 (d, *J*_C–F_ = 27.0 Hz), 23.68, 22.83 (q, *J*_C–F_ = 2.5 Hz). ESI-HRMS ([M + H]^+^, *m*/*z*): calcd for C_18_H_14_ON_4_F_7_ 435.1050, found 435.1046. HPLC: *t*_R_ = 5.110 (method C, purity 98.72%).

Ethyl 1-(3-(4-Fluorophenyl)-6-(trifluoromethyl)isoxazolo[5,4*-d*]pyrimidin-4-yl)piperidine-3-carboxylate (**21l**). Synthesized according to general procedure F from compound **12h** (1.0 equiv., 0.34 mmol, 0.110 g), K_2_CO_3_ (3.0 equiv., 1.04 mmol, 0.144 g), and ethyl nipecotate ( 1.18 mmol, 0.18 mL). The product was purified by column chromatography, using EtOAc/*n*-hexane (1:4, v/v) as the mobile phase. Colorless oil. Yield: 72%. Rf (EtOAc/*n*-hexane = 1:3, v/v) = 0.45. ^1^H NMR (400 MHz, CDCl_3_) δ (ppm) 7.70–7.58 (m, 2H), 7.37–7.21 (m, 2H), 4.04 (m, 4H), 3.25 (dd, *J* = 13.5, 9.7 Hz, 1H), 3.11 (td, *J* = 11.9, 3.0 Hz, 1H), 2.42 (p, *J* = 5.6 Hz, 1H), 2.15–1.96 (m, 1H), 1.78–1.62 (m, 2H), 1.47 (dd, *J* = 13.6, 10.0 Hz, 1H), 1.18 (t, *J* = 7.1 Hz, 3H). ^13^C NMR (100 MHz, CDCl_3_) δ (ppm) 177.31, 172.20, 164.17 (d, *J*_C–F_ = 252.3 Hz), 160.38, 156.59, 155.17 (d, *J*_C–F_ = 37.3 Hz), 130.63 (d, *J*_C–F_ = 8.7 Hz), 125.45 (d, *J*_C–F_ = 3.4 Hz), 119.30 (q, *J*_C–F_ = 276.1 Hz), 116.60 (d, *J*_C–F_ = 22.1 Hz), 96.51, 60.87, 50.63, 49.05, 40.95, 26.77, 23.99, 14.04. ESI-HRMS ([M + H]^+^, *m*/*z*): calcd for C_20_H_19_O_3_N_4_F_4_ 439.1388, found 439.1382. HPLC: *t*_R_ = 4.787 (method C, purity 97.92%).

1-(3-(4-Fluorophenyl)-6-(trifluoromethyl)isoxazolo[5,4*-d*]pyrimidin-4-yl)piperidine-3-carboxylic Acid (**21m**). Compound **21l** (1.0 equiv., 0.20 mmol, 0.089 g) was dissolved in THF/H_2_O (3:2, v/v) and cooled to 0 °C, and LiOH × H_2_O was added (2.0 equiv., 0.40 mmol, 0.017 g). The reaction mixture was stirred at room temperature overnight. Next day, 10% citric acid and CH_2_Cl_2_ were added. The phases were separated. The organic phase was dried over anhydrous Na_2_SO_4_, filtered, and evaporated under reduced pressure. White waxy solid. Yield: 95%. ^1^H NMR (400 MHz, DMSO*-d*_*6*_) δ (ppm) 7.85–7.69 (m, 2H), 7.51–7.36 (m, 2H), 3.90 (m, 2H), 3.15 (dd, *J* = 13.3, 9.7 Hz, 2H), 2.44–2.30 (m, 1H), 1.94–1.81 (m, 1H), 1.55 (m, 2H), 1.38 (m, 1H). ^13^C NMR (100 MHz, DMSO*-d*_*6*_) δ (ppm) 176.88, 174.23, 163.91 (d, *J*_C–F_ = 248.5 Hz), 160.13, 157.59, 153.83 (d, *J*_C–F_ = 36.1 Hz), 131.58 (d, *J*_C–F_ = 8.8 Hz), 125.87 (d, *J*_C–F_ = 3.3 Hz), 119.83 (d, *J*_C–F_ = 276.0 Hz), 116.85 (d, *J*_C–F_ = 22.0 Hz), 96.95, 67.48, 55.38, 45.98, 30.88, 26.62, 25.59, 23.91. ESI-HRMS ([M + H]^+^, *m*/*z*): calcd for C_18_H_15_O_3_N_4_F_4_ 411.1075, found 411.1069. HPLC: *t*_R_ = 5.867 (method B, purity 98.61%).

1-(3-(4-Fluorophenyl)-6-(trifluoromethyl)isoxazolo[5,4*-d*]pyrimidin-4-yl)piperidin-3-amine (**21n**). Synthesized according to general procedure F from compound **12h** (1.0 equiv., 0.31 mmol, 0.100 g), K_2_CO_3_ (3.0 equiv., 0.93 mmol, 0.128 g), and *tert*-butyl piperidin-3-ylcarbamate (3.0 equiv., 0.93 mmol, 0.126 g). The product was purified by column chromatography, using EtOAc/*n*-hexane (1:3, v/v) as the mobile phase. Colorless oil. Yield: 76%. Rf (EtOAc/*n*-hexane = 1:3, v/v) = 0.30. The product was used in the next step without purification. 4 M HCl in dioxane (10 equiv., 0.60 mL) was added, and the reaction mixture was stirred at room temperature overnight (16 h). The next day, the solvent was evaporated under reduced pressure and the remaining residue was washed several times with Et_2_O. White waxy solid. Yield: 97%. ^1^H NMR (400 MHz, DMSO*-d*_*6*_) δ (ppm) 8.32 (bs, 3H), 7.98–7.74 (m, 2H), 7.55–7.34 (m, 2H), 4.31 (m, 1H), 3.37 (m, 2H), 3.21 (s, 1H), 3.03 (m, 1H), 2.03–1.85 (m, 1H), 1.58 (d, *J* = 10.4 Hz, 1H), 1.43 (s, 1H), 1.30 (d, *J* = 10.9 Hz, 1H). ^13^C NMR (100 MHz, DMSO*-d*_*6*_) δ (ppm) 176.90, 163.91 (d, *J*_*C–F*_ = 248.7 Hz), 160.69, 157.55, 153.79 (d, *J*_C–F_ = 36.5 Hz), 131.79 (d, *J*_C–F_ = 8.8 Hz), 119.81 (q, *J*_C–F_ = 275.9 Hz), 116.83 (d, *J*_C–F_ = 22.0 Hz), 97.17, 66.82, 49.66, 46.47, 27.61, 22.29. ESI-HRMS ([M + H]^+^, *m*/*z*): calcd for C_17_H_16_ON_5_F_4_ 382.1285, found 382.1277. HPLC: *t*_R_ = 4.173 (method B, purity 98.28%).

4-(3,3*-*Dimethylpiperidin-1-yl)-3-(4-fluorophenyl)-6-(trifluoromethyl)isoxazolo[5,4*-d*]pyrimidine (**21o**). Synthesized according to general procedure F from compound **12h** (1.0 equiv., 0.5 mmol, 0.16 g), K_2_CO_3_ (3.0 equiv., 1.5 mmol, 0.21 g), and 3,3*-*dimethylpiperidine (3.0 equiv., 1.5 mmol, 0.22 g). The product was purified by column chromatography, using Et_2_O/petroleum ether (1:10, v/v) as the mobile phase. Yellow oil. Yield: 70%. Rf (Et_2_O/petroleum ether = 1:10, v/v) = 0.35. ^1^H NMR (400 MHz, DMSO*-d*_*6*_) δ (ppm) 7.84–7.70 (m, 2H), 7.53–7.40 (m, 2H), 3.38 (bs, 2H), 3.30–3.22 (m, 2H), 1.32 (bs, 4H), 0.74 (s, 6H). ^13^C NMR (100 MHz, CDCl_3_) δ (ppm) 181.68, 168.59 (d, *J*_C–F_ = 248.4 Hz), 164.53, 162.32, 158.69 (q, *J*_C–F_ = 36.2 Hz), 136.56 (d, *J*_C–F_ = 8.8 Hz), 131.14 (d, *J*_C–F_ = 3.3 Hz), 124.58 (d, *J*_C–F_ = 276.0 Hz), 121.48 (d, *J*_C–F_ = 22.1 Hz), 101.12, 63.33, 54.29, 41.37, 37.11, 30.75, 26.39. ESI-HRMS ([M + H]^+^, *m*/*z*): calcd for C_19_H_19_F_4_N_4_O 395.1489, found 395.1485. HPLC: *t*_R_ = 5.277 (method C, purity 96.23%).

3-(4-Fluorophenyl)-4-(5-azaspiro[2.5]octan-5-yl)-6-(trifluoromethyl)isoxazolo[5,4*-d*]pyrimidine (**21p**). Synthesized according to general procedure F from compound **12h** (1.0 equiv., 0.5 mmol, 0.16 g), K_2_CO_3_ (3.0 equiv., 1.5 mmol, 0.21 g), and 5-azaspiro[2.5]octane (1.35 equiv., 0.67 mmol, 0.10 mg). The product was purified by column chromatography, using Et_2_O/petroleum ether (1:10, v/v) as the mobile phase. Colorless oil. Yield: 42%. Rf (diethyl ether/petroleum ether = 1:10, v/v) = 0.40. ^1^H NMR (400 MHz, CDCl_3_) δ (ppm) 7.78–7.48 (m, 2H), 7.33–7.17 (m, 2H), 3.62–3.36 (m, 4H), 1.56–1.47 (m, 2H), 1.47–1.37 (m, 2H), 0.36 (bs, 2H), 0.27 (t, *J* = 3.0 Hz, 2H). ^13^C NMR (100 MHz, CDCl_3_) δ (ppm) 177.40, 162.83, 159.72, 156.61, 155.01 (q, *J*_C–F_ = 37.0 Hz), 130.62 (d, *J*_C–F_ = 8.6 Hz), 125.73 (d, *J*_C–F_ = 3.5 Hz), 123.50, 119.38 (d, *J*_C–F_ = 276.1 Hz), 116.51 (d, *J*_C–F_ = 22.0 Hz), 56.34, 50.21, 33.03, 25.18, 18.97, 11.01. ESI-HRMS ([M + H]^+^, *m*/*z*): calcd for C_19_H_17_F_4_N_4_O 393.1333, found 393.1328. HPLC: *t*_R_ = 5.210 (method C, purity 99.45%).

3-(4-Fluorophenyl)-4-(3-isopropylpiperidin-1-yl)-6-(trifluoromethyl)isoxazolo[5,4*-d*]pyrimidine (**21q**). Synthesized according to general procedure F from compound **12h** (1.0 equiv., 0.5 mmol, 0.16 g), K_2_CO_3_ (3.0 equiv., 1.5 mmol, 0.21 g), and 3-isopropylpiperidine (1.5 equiv., 0.78 mmol, 0.10 mg). The product was purified by column chromatography, using Et_2_O/petroleum ether (1:10, v/v) as the mobile phase. Colorless oil. Yield: 79%. Rf (Et_2_O/petroleum ether = 1:10, v/v) = 0.30. ^1^H NMR (400 MHz, CDCl_3_) δ (ppm) 7.80–7.59 (m, 2H), 7.26 (t, *J* = 8.6 Hz, 2H), 4.16 (bs, 2H), 2.83 (td, *J* = 12.8, 3.1 Hz, 1H), 2.62 (dd, *J* = 12.6, 9.7 Hz, 1H), 1.97–1.79 (m, 1H), 1.72–1.59 (m, 1H), 1.49–1.30 (m, 1H), 1.18 (dtt, *J* = 15.0, 11.4, 5.7 Hz, 3H), 0.86–0.63 (m, 6H). ^13^C NMR (100 MHz, CDCl_3_) δ (ppm) 177.43, 164.15 (d, *J*_C–F_ = 252.0 Hz), 159.86, 156.69, 155.01 (d, *J*_C–F_ = 37.1 Hz), 130.65 (d, *J*_C–F_ = 8.5 Hz), 125.62 (d, *J*_C–F_ = 3.6 Hz), 123.34, 119.38 (d, *J*_C–F_ = 276.1 Hz), 116.46 (d, *J*_C–F_ = 22.0 Hz), 95.73, 42.44, 30.57, 27.28, 25.32, 19.73, 19.33. ESI-HRMS ([M + H]^+^, *m*/*z*): calcd for C_20_H_21_F_4_N_4_O 409.1646, found 409.1642. HPLC: *t*_R_ = 5.467 (method C, purity 97.58%).

4-(5,6*-*Dihydropyridin-1(2H)-yl)-3-(4-fluorophenyl)-6-(trifluoromethyl)isoxazolo[5,4*-d*]pyrimidine (**21r**). Synthesized according to general procedure F from compound **12h** (1.0 equiv., 0.5 mmol, 0.16 g), K_2_CO_3_ (3.0 equiv., 1.5 mmol, 0.21 g), and 1,2,3,6-tetrahydropyridine (3.0 equiv., 1.5 mmol, 0.136 mL). The product was purified by column chromatography, using EtOAc/*n*-hexane (1:1, v/v) as the mobile phase. Yellow oil. Yield: 10%. Rf (EtOAc/*n*-hexane = 1:1, v/v) = 0.50. ^1^H NMR (400 MHz, CDCl_3_) δ (ppm) 7.75–7.50 (m, 2H), 7.33–7.15 (m, 2H), 5.82 (m, 1H), 5.53–5.34 (m, 1H), 3.81 (m, 4H), 2.15 (m, 2H). ^13^C NMR (100 MHz, CDCl_3_) δ (ppm) 177.25, 165.36, 162.85, 160.05, 156.66, 155.08 (d, *J*_C–F_ = 37.2 Hz), 130.76 (d, *J*_C–F_ = 8.5 Hz), 125.55 (d, *J*_C–F_ = 3.5 Hz), 123.48, 119.35 (d, *J*_C–F_ = 276.1 Hz), 116.55 (d, *J*_C–F_ = 22.1 Hz), 96.26, 49.74, 44.94, 24.53. ESI-HRMS ([M + H]^+^, *m*/*z*): calcd for C_17_H_13_F_4_N_4_O 365.1020, found 365.1010. HPLC: *t*_R_ = 7.050 (method B, purity 97.16%).

4-(3,3*-*Dimethylpyrrolidin-1-yl)-3-(4-fluorophenyl)-6-(trifluoromethyl)isoxazolo[5,4*-d*]pyrimidine (**21s**). Synthesized according to general procedure F from compound **12h** (1.0 equiv., 0.5 mmol, 0.16 g), K_2_CO_3_ (3.0 equiv., 1.5 mmol, 0.21 g), and 3,3*-*dimethylpyrrolidine (3.0 equiv., 1.5 mmol, 0.149 g). The product was purified by column chromatography, using EtOAc/*n*-hexane (1:1, v/v) as the mobile phase. Colorless oil. Yield: 21%. Rf (EtOAc/*n*-hexane = 1:1, v/v) = 0.57. ^1^H NMR (400 MHz, CDCl_3_) δ (ppm) 7.82–7.48 (m, 2H), 7.39–7.12 (m, 2H), 3.87 (s, 1H), 3.53 (s, 1H), 3.06–2.87 (m, 1H), 2.55 (s, 1H), 1.63 (s, 2H), 1.22–0.74 (m, 6H). ^13^C NMR (100 MHz, CDCl_3_) δ (ppm) 176.51, 165.23, 162.73, 156.89, 155.04 (d, *J*_C–F_ = 37.4 Hz), 131.49 (d, *J*_C–F_ = 8.5 Hz), 126.18 (d, *J*_C–F_ = 3.6 Hz), 119.39 (d, *J*_C–F_ = 276.0 Hz), 115.92 (d, *J*_C–F_ = 22.0 Hz), 96.66, 62.55, 50.06, 37.64, 25.61. ESI-HRMS ([M + H]^+^, *m*/*z*): calcd for C_18_H_17_F_4_N_4_O 381.1333, found 381.1323. HPLC: *t*_R_ = 7.327 (method B, purity 100%).

3-(4-Fluorophenyl)-4-(3-methoxypyrrolidin-1-yl)-6-(trifluoromethyl)isoxazolo[5,4*-d*]pyrimidine (**21t**). Synthesized according to general procedure F from compound **12h** (1.0 equiv., 0.5 mmol, 0.16 g), K_2_CO_3_ (3.0 equiv., 1.5 mmol, 0.21 g), and 3-methoxypiperidine hydrochloride (3.0 equiv., 1.5 mmol, 0.21 g). The product was purified by column chromatography, using EtOAc as the mobile phase. Yellow solid. Yield: 10%. Mp = 113–117 °C. Rf (EtOAc) = 0.57. ^1^H NMR (400 MHz, CDCl_3_) δ (ppm) 7.57 (dd, *J* = 7.8, 5.3 Hz, 2H), 7.21–7.28 (m, 2H), 3.56–4.18 (m, 3H), 3.24 (bs, 3H), 2.68–3.13 (m, 2H), 1.93–2.10 (m, 1H), 1.75–1.91 (m, 1H). ^13^C NMR (100 MHz, DMSO*-d*_*6*_) δ (ppm) 175.53, 163.28 (d, *J*_C–F_ = 248.0 Hz), 157.24, 153.39 (q, *J*_C–F_ = 35.9 Hz), 132.14 (d, *J*_C–F_ = 8.8 Hz), 126.06 (d, *J*_C–F_ = 3.7 Hz), 119.40 (q, *J*_C–F_ = 275.6 Hz), 115.76 (d, *J*_C–F_ = 22.0 Hz), 97.23, 76.97, 55.74, 54.16, 47.43, 28.12. ESI-HRMS ([M + H]^+^, *m*/*z*): calcd for C_17_H_15_F_4_N_4_O_2_ 383.1126, found 383.1122. HPLC: *t*_R_ = 6.497 (method B, purity 97.59%).

4-(2,5*-*Dihydro-1*H*-pyrrol-1-yl)-3-(4-fluorophenyl)-6-(trifluoromethyl)isoxazolo[5,4*-d*]pyrimidine (**21u**). Synthesized according to general procedure F from compound **12h** (1.0 equiv., 0.5 mmol, 0.16 g), K_2_CO_3_ (3.0 equiv., 1.5 mmol, 0.21 g), and 2,5*-*dihydro-1*H*-pyrrole hydrochloride (3.0 equiv., 1.5 mmol, 0.158 g). The product was purified by column chromatography, using EtOAc/*n*-hexane (1:1, v/v) as the mobile phase. Yellow oil. Yield: 32%. Rf (EtOAc/*n*-hexane = 1:1, v/v) = 0.67. ^1^H NMR (400 MHz, CDCl_3_) δ (ppm) 7.68–7.44 (m, 2H), 7.31–7.21 (m, 2H), 6.00–5.55 (m, 2H), 4.61 (m, 2H), 3.62 (m, 2H). ^13^C NMR (1001 MHz, CDCl_3_) δ (ppm) 176.54, 165.23, 162.73, 157.15, 156.71, 155.28 (d, *J*_C–F_ = 37.0 Hz), 131.65 (d, *J*_C–F_ = 8.6 Hz), 126.15 (d, *J*_C–F_ = 3.6 Hz), 124.90, 119.35 (d, *J*_C–F_ = 275.9 Hz), 116.03 (d, *J*_C–F_ = 22.0 Hz), 96.97, 56.89. ESI-HRMS ([M + H]^+^, *m*/*z*): calcd for C_16_H_11_F_4_N_4_O 351.0863, found 351.0852. HPLC: *t*_R_ = 6.683 (method B, purity 95.14%).

3-(4-Fluorophenyl)-4-(pyrrolidin-1-yl)-6-(trifluoromethyl)isoxazolo[5,4*-d*]pyrimidine (**21v**). Synthesized according to general procedure F from compound **12h** (1.0 equiv., 0.5 mmol, 0.16 g), K_2_CO_3_ (3.0 equiv., 1.5 mmol, 0.21 g), and pyrrolidine (3.0 equiv., 1.5 mmol, 0.123 mL). The product was purified by column chromatography, using EtOAc/*n*-hexane (1:3, v/v) as the mobile phase. Yellow solid. Yield: 10%. Mp = 139–143 °C. Rf (EtOAc/*n*-hexane = 1:3, v/v) = 0.37. ^1^H NMR (400 MHz, DMSO*-d*_*6*_) δ (ppm) 7.70–7.78 (m, 2H), 7.38–7.46 (m, 2H), 3.56–3.73 (m, 2H), 2.73–2.90 (m, 2H), 1.58–1.86 (m, 4H). ^13^C NMR (100 MHz, DMSO*-d*_*6*_) δ (ppm) 176.00, 163.67 (d, *J*_C–F_ = 248.5 Hz), 157.96, 157.27, 153.06 (d, *J*_C–F_ = 35.9 Hz), 132.53 (d, *J*_C–F_ = 8.7 Hz), 126.51 (d, *J*_C–F_ = 3.3 Hz), 119.87 (d, *J*_C–F_ = 276.6 Hz), 116.16 (d, *J*_C–F_ = 22.0 Hz), 96.72, 51.09, 25.31. ESI-HRMS ([M + H]^+^, *m*/*z*): calcd for C_16_H_13_F_4_N_4_O 353.1020, found 353.1016. HPLC: *t*_R_ = 6.820 (method B, purity 98.48%).

4-(3-(4-Fluorophenyl)-6-(trifluoromethyl)isoxazolo[5,4*-d*]pyrimidin-4-yl)piperazin-2-one (**21w**). Synthesized according to general procedure F from compound **12h** (1.0 equiv., 0.5 mmol, 0.16 g), K_2_CO_3_ (3.0 equiv., 1.5 mmol, 0.21 g), and 2-oxopiperazine (3.0 equiv., 1.5 mmol, 0.15 g). The product was purified by column chromatography, using EtOAc as the mobile phase. White solid. Yield: 5%. Mp >200 °C. Rf (EtOAc) = 0.42. ^1^H NMR (400 MHz, acetone*-d*_*6*_) δ (ppm) 7.86–7.92 (m, 2H), 7.41–7.48 (m, 2H), 7.23 (s, 1H), 3.78–3.89 (m, 4H), 3.38 (td, *J* = 5.4, 2.7 Hz, 2H). ^13^C NMR (100 MHz, DMSO*-d*_*6*_) δ (ppm) 176.64, 163.92 (d, *J*_C–F_ = 248.5 Hz), 165.32, 159.53, 157.56, 153.72 (q, *J*_C–F_*=* 36.5 Hz), 131.94 (d, *J*_C–F_ = 348.8 Hz), 125.85 (d, *J*_C–F_ = 3.3 Hz), 121.14 (q, *J*_C–F_ = 275.7 Hz), 116.84 (d, *J*_C–F_ = 22.1 Hz), 97.53, 52.35, 44.93, 38.94. ESI-HRMS ([M + H]^+^, *m*/*z*): calcd for C_16_H_11_F_4_N_5_O_2_ 382.0922, found 382.0920. HPLC: *t*_R_ = 5.060 (method B, purity 99.25%).

3-(4-Fluorophenyl)-4-(4-phenylpiperidin-1-yl)-6-(trifluoromethyl)isoxazolo[5,4*-d*]pyrimidine (**21x**). Synthesized according to general procedure F from compound **12h** (1.0 equiv., 0.5 mmol, 0.16 g), K_2_CO_3_ (3.0 equiv., 1.5 mmol, 0.21 g), and 4-phenylpiperidine (3.0 equiv., 1.5 mmol, 0.24 g). The product was purified by column chromatography, using Et_2_O/petroleum ether (1:10, v/v) as the mobile phase. Yellow oil. Yield: 62%. Rf (Et_2_O/petroleum ether = 1:10, v/v) = 0.30. ^1^H NMR (400 MHz, DMSO*-d*_*6*_) δ (ppm) 7.94–7.67 (m, 2H), 7.58–7.39 (m, 2H), 7.36–7.25 (m, 2H), 7.22–7.06 (m, 3H), 4.21 (bs, 2H), 3.06 (td, *J* = 12.9, 2.9 Hz, 2H), 2.84–2.71 (m, 1H), 1.92–1.44 (m, 4H). ^13^C NMR (100 MHz, DMSO*-d*_*6*_) δ (ppm) 181.71, 169.83, 167.37, 164.50, 162.37, 158.67 (d, *J*_C–F_ = 36.3 Hz), 150.24, 136.47 (d, *J*_C–F_ = 8.8 Hz), 133.62, 131.85, 131.50, 130.81 (d, *J*_C–F_ = 3.2 Hz), 124.62 (d, *J*_C–F_ = 276.0 Hz), 121.56 (d, *J*_C–F_ = 22.0 Hz), 101.49, 37.51. ESI-HRMS ([M + H]^+^, *m*/*z*): calcd for C_23_H_19_F_4_N_4_O 443.1486, found 443.1489. HPLC: *t*_R_ = 5.345 (method C, purity 97.08%).

3-(4-Fluorophenyl)-4-(3-methylcyclohexyl)-6-(trifluoromethyl)isoxazolo[5,4*-d*]pyrimidine (**21y**). Grignard reagent was prepared from 1-bromo-3-methylcyclohexane (5.0 equiv., 1.5 mmol, 0.208 mL), Mg (30.0 equiv., 9.0 mmol, 0.219 g), and 1,2*-*dibromoethane (0.5 equiv., 0.15 mmol, 0.013 mL) in THF at 70 °C. The prepared Grignard reagent was then added dropwise to the mixture of **12h** (1.0 equiv., 0.3 mmol, 0.1 g) and K_2_CO_3_ (3.0 equiv., 0.9 mmol, 0.124 g) in THF. The reaction mixture was stirred at 70 °C for 16 h. The product was purified by column chromatography, using EtOAc/*n*-hexane (1:15, v/v) as the mobile phase. A mixture of diastereomers was obtained. Colorless oil. Yield: 20%. Rf (EtOAc/*n*-hexane = 1:15, v/v) = 0.35. ^1^H NMR (400 MHz, CDCl_3_) major isomer δ (ppm) 8.14–8.08 (m, 2H), 7.26–7.20 (m, 2H), 5.54–5.41 (m, 1H), 2.30–2.22 (m, 2H), 1.89–1.85 (m, 1H), 1.75 (bs, 1H), 1.49–1.42 (m, 2H), 1.27–1.16 (m, 2H), 1.00 (d, *J* = 6.6 Hz, 3H), 0.89–0.84 (m, 1H). ^13^C NMR (100 MHz, CDCl_3_) δ (ppm) 177.09, 165.61 (d, *J*_C–F_ = 43.8 Hz), 163.32, 156.56, 156.06 131.28 (d, *J*_C–F_ = 8.8 Hz), 123.16 (d, *J*_C–F_ = 3.4 Hz), 119.04 (d, *J*_C–F_ = 275.8 Hz), 115.99 (d, *J*_C–F_ = 22.2 Hz), 99.39, 79.38, 40.14, 33.77, 32.65, 31.33, 23.78, 22.27. HPLC: *t*_R_ = 5.663 (method C, purity 86.22% (major isomer)).

*N*-(1-(3-(4-Fluorophenyl)-6-(trifluoromethyl)isoxazolo[5,4*-d*]pyrimidin-4-yl)piperidin-3-yl)acetamide (**21z**). Synthesized according to general procedure F from compound **12h** (1.0 equiv., 0.22 mmol, 0.07 g), K_2_CO_3_ (3.0 equiv., 0.66 mmol, 0.091 g), and 3-acetamidopiperidine (3.0 equiv., 0.66 mmol, 0.062 mg). The product was purified by column chromatography, using CH_2_Cl_2_/MeOH (15:1, v/v) as the mobile phase. Yellow waxy solid. Yield: 93%. Rf (CH_2_Cl_2_/MeOH = 9:1, v/v) = 0.50. ^1^H NMR (400 MHz, CDCl_3_) δ (ppm) 7.74–7.54 (m, 2H), 7.34–7.23 (m, 2H), 5.95 (s, 1H), 3.92–3.82 (m, 1H), 3.67 (d, *J* = 4.1 Hz, 2H), 3.50 (s, 1H), 3.29 (ddd, *J* = 13.2, 6.8, 3.8 Hz, 1H), 1.86 (s, 3H), 1.79–1.69 (m, 2H), 1.58 (dd, *J* = 8.8, 4.7 Hz, 1H), 1.47 (ddq, *J* = 10.6, 7.0, 3.8 Hz, 1H). ^13^C NMR (100 MHz, CDCl_3_) δ (ppm) 177.28, 169.86, 164.21 (d, *J*_C–F_ = 252.3 Hz), 160.71, 156.62, 155.22 (d, *J*_C–F_ = 37.0 Hz), 130.73 (d, *J*_C–F_ = 8.7 Hz), 125.13 (d, *J*_C–F_ = 3.4 Hz), 119.27 (d, *J*_C–F_ = 276.2 Hz), 116.53 (d, *J*_C–F_ = 22.1 Hz), 96.91, 52.18, 46.40, 28.68, 23.10, 22.39, 14.20. ESI-HRMS ([M + H]^+^, *m*/*z*): calcd for C_19_H_18_O_2_N_5_F_4_ 424.1391, found 424.1384. HPLC: *t*_R_ = 3.717 (method C, purity 97.89%).

3-(3-Bromo-4-fluorophenyl)-4-(3-methylpiperidin-1-yl)-6-(trifluoromethyl)isoxazolo[5,4*-d*]pyrimidine (**22**). Synthesized according to general procedure F from compound **12i** (1.0 equiv., 1.0 mmol, 0.39 g), K_2_CO_3_ (3.0 equiv., 3 mmol, 0.42 g), and 3-methylpiperidine (3.0 equiv., 3.0 mmol, 0.352 mL). The product was purified by column chromatography, using EtOAc/*n*-hexane (1:3, v/v) as the mobile phase. Light-yellow solid. Yield: 7%. Mp = 96–100 °C. Rf (EtOAc/*n*-hexane = 1:3, v/v) = 0.52. ^1^H NMR (400 MHz, CDCl_3_) δ (ppm) 7.85 (dd, *J* = 6.4, 2.1 Hz, 1H), 7.59–7.64 (m, 1H), 7.33 (t, *J* = 8.3 Hz, 1H), 3.98 (d, *J* = 12.2 Hz, 1H), 4.18 (d, *J* = 8.6 Hz, 1H), 2.84 (td, *J* = 12.9, 3.0 Hz, 1H), 2.55 (dd, *J* = 12.9, 11.1 Hz, 1H), 1.82 (dd, *J* = 13.2, 3.5 Hz, 1H), 1.59–1.70 (m, 1H) 1.37–1.55 (m, 2H), 1.11 (qd, *J* = 12.6, 4.0 Hz, 1H), 0.70 (d, *J* = 6.6 Hz, 3H). ^13^C NMR (100 MHz, CDCl_3_) δ (ppm) 177.45, 160.87 (d, *J*_C–F_ = 187.8 Hz), 159.28, 155.54, 155.24 (d, *J*_C–F_ = 37.4 Hz), 133.95 (d *J*_C–F_ = 1.4 Hz), 129.30 (d, *J*_C–F_ = 7.9 Hz), 127.10 (d, *J*_C–F_ = 3.7 Hz), 119.31 (d, *J*_C–F_ = 276.1 Hz), 117.43 (d, *J*_C–F_ = 22.7 Hz), 110.25 (d, *J*_C–F_ = 21.9 Hz), 95.74, 56.27, 49.35, 32.32, 31.18, 25.05, 18.61. ESI-HRMS ([M + H]^+^, *m*/*z*): calcd for C_18_H_16_BrF_4_N_4_O 459.0438, found 459.0434. HPLC: *t*_R_ = 7.987 (method B, purity 99.30%).

3-(3,4*-*Difluorobenzyl)-4-(3-methylpiperidin-1-yl)-6-(trifluoromethyl)isoxazolo[5,4*-d*]pyrimidine (**23a**). Synthesized according to general procedure F from compound **12j** (1.0 equiv., 0.32 mmol, 0.111 g), K_2_CO_3_ (3.0 equiv., 0.95 mmol, 0.13 g), and 3-methylpiperidine (3.0 equiv., 0.95 mmol, 0.074 mL). The product was purified by column chromatography, using EtOAc/*n*-hexane (1:4, v/v) as the mobile phase. Colorless oil. Yield: 58%. Rf (EtOAc/*n*-hexane = 1:3, v/v) = 0.45. ^1^H NMR (400 MHz, CDCl_3_) δ (ppm) 7.14 (dt, *J* = 10.0, 8.3 Hz, 1H), 7.03 (ddd, *J* = 11.0, 7.3, 2.3 Hz, 1H), 6.95–6.83 (m, 1H), 4.47–4.20 (m, 4H), 3.04 (ddd, *J* = 13.1, 11.9, 3.1 Hz, 1H), 2.74 (dd, *J* = 12.9, 10.8 Hz, 1H), 1.93–1.81 (m, 1H), 1.72 (dp, *J* = 13.9, 3.5 Hz, 1H), 1.67–1.57 (m, 1H), 1.48 (dddd, *J* = 16.0, 13.6, 8.0, 4.0 Hz, 1H), 1.29–1.09 (m, 1H), 0.84 (d, *J* = 6.6 Hz, 3H). ^13^C NMR (100 MHz, CDCl_3_) δ (ppm) 177.51, 159.49, 155.09 (q, *J*_C–F_ = 37.1 Hz), 154.33, 151.78 (d, *J*_C–F_ = 262.8 Hz), 150.53 (d, *J*_C–F_ = 237.2 Hz), 149.67 (d, *J*_C–F_ = 236.5 Hz), 132.10 (d, *J*_C–F_ = 9.5 Hz), 124.15 (d, *J*_C–F_ = 6.3 Hz), 120.68, 117.86, 117.32, 55.17, 48.51, 33.90, 32.37, 31.39, 25.01, 18.73. ESI-HRMS ([M + H]^+^, *m*/*z*): calcd for C_19_H_18_ON_4_F_5_ 413.1395, found 413.1389. HPLC: *t*_R_ = 5.180 (method C, purity 96.82%).

3-(3,4*-*Difluorobenzyl)-*N*-isobutyl-6-(trifluoromethyl)isoxazolo[5,4*-d*]pyrimidin-4-amine (**23b**). Synthesized according to general procedure F from compound **12j** (1.0 equiv., 0.25 mmol, 0.086 g), K_2_CO_3_ (3.0 equiv., 0.73 mmol, 0.10 g), and isobutylamine (3.0 equiv., 0.73 mmol, 0.048 mL). The product was purified by column chromatography, using EtOAc/*n*-hexane (1:3, v/v) as the mobile phase. Yellow oil. Yield: 60%. Rf (EtOAc/*n*-hexane = 1:3, v/v) = 0.25. ^1^H NMR (400 MHz, CDCl_3_) δ (ppm) 7.26–7.20 (m, 1H), 7.10 (ddd, *J* = 10.1, 7.2, 2.3 Hz, 1H), 7.05–6.99 (m, 1H), 4.91 (s, 1H), 4.32 (d, *J* = 1.2 Hz, 2H), 3.30 (dd, *J* = 6.8, 5.8 Hz, 2H), 1.74–1.60 (m, 1H), 0.77 (d, *J* = 6.7 Hz, 6H). ^13^C NMR (100 MHz, CDCl_3_) δ (ppm) 176.40, 158.20, 156.98 (d, *J*_C–F_ = 37.1 Hz) 155.18, 151.43 (d, *J*_C–F_ = 239.0 Hz), 149.48 (d, *J*_C–F_ = 239.8 Hz), 131.51 (d, *J*_C–F_ = 4.7 Hz), 124.40 (d, *J*_C–F_ = 6.3 Hz), 119.10 (d, *J*_C–F_ = 274.069), 118.90, 117.71, 117.65, 48.79, 32.05, 28.14, 19.73. ESI-HRMS ([M + H]^+^, *m*/*z*): calcd for C_17_H_16_ON_4_F_5_ 387.1239, found 387.1234. HPLC: *t*_R_ = 7.190 (method B, purity 97.91%).

3-Cyclopropyl-4-(3-methylpiperidin-1-yl)-6-(trifluoromethyl)isoxazolo[5,4*-d*]pyrimidine (**24a**). Synthesized according to general procedure F from compound **12k** (1.0 equiv., 0.46 mmol, 0.123 g), K_2_CO_3_ (3.0 equiv., 1.40 mmol, 0.19 g), and 3-methylpiperidine (3.0 equiv., 1.40 mmol, 0.109 mL). The product was purified by column chromatography, using EtOAc/*n*-hexane (1:4, v/v) as the mobile phase. Light-yellow oil. Yield: 68%. Rf (EtOAc/*n*-hexane = 1:3, v/v) = 0.45. ^1^H NMR (400 MHz, CDCl_3_) δ (ppm) 4.99–4.79 (m, 1H), 4.71 (ddt, *J* = 13.1, 3.8, 1.8 Hz, 1H), 3.16 (ddd, *J* = 13.2, 12.1, 3.0 Hz, 1H), 2.89 (dd, *J* = 13.1, 10.9 Hz, 1H), 1.97 (tt, *J* = 8.2, 5.2 Hz, 2H), 1.88 (dt, *J* = 13.6, 3.5 Hz, 1H), 1.78 (dtd, *J* = 10.8, 6.8, 4.0 Hz, 1H), 1.72–1.62 (m, 1H), 1.40–1.24 (m, 3H), 1.22–1.12 (m, 2H), 1.01 (d, *J* = 6.7 Hz, 3H). ^13^C NMR (100 MHz, CDCl_3_) δ (ppm) 177.13, 159.65, 157.86, 154.81 (q, *J*_C–F_ = 36.9 Hz), 119.40 (q, *J*_*C–F*_ = 276.1 Hz), 97.44, 55.35, 48.44, 32.76, 31.62, 25.30, 18.98, 10.56, 8.92. ESI-HRMS ([M + H]^+^, *m*/*z*): calcd for C_15_H_18_ON_4_F_3_ 327.1427, found 327.1423. HPLC: *t*_R_ = 4.910 (method C, purity 98.21%).

3-Cyclopropyl-*N*-isobutyl-6-(trifluoromethyl)isoxazolo[5,4*-d*]pyrimidin-4-amine (**24b**). Synthesized according to general procedure F from compound **12k** (1.0 equiv., 0.46 mmol, 0.123 g), K_2_CO_3_ (3.0 equiv., 1.40 mmol, 0.19 g), and isobutylamine (3.0 equiv., 1.40 mmol, 0.093 mL). The product was purified by column chromatography, using EtOAc/*n*-hexane (1:3, v/v) as the mobile phase. Light-yellow oil. Yield: 76%. Rf (EtOAc/*n*-hexane = 1:3, v/v) = 0.25. ^1^H NMR (400 MHz, CDCl_3_) δ (ppm) 5.92 (s, 1H), 3.58 (dd, *J* = 6.8, 5.9 Hz, 2H), 2.21–1.92 (m, 2H), 1.35–1.10 (m, 4H), 1.04 (d, *J* = 6.7 Hz, 6H). ^13^C NMR (100 MHz, CDCl_3_) δ (ppm) 175.70, 159.11, 158.25, 156.66 (q, *J*_C–F_ = 36.7 Hz), 119.34 (q, *J*_C–F_ = 275.9 Hz), 97.42, 48.59, 28.43, 20.05, 7.24, 6.05. ESI-HRMS ([M + H]^+^, *m*/*z*): calcd for C_13_H_16_ON_4_F_3_ 301.1271, found 301.1268. HPLC: *t*_R_ = 4.567 (method C, purity 98.30%).

4-(4-(3-Methylpiperidin-1-yl)-6-(trifluoromethyl)isoxazolo[5,4*-d*]pyrimidin-3-yl)piperidin-1-ium chloride (**25a**). Synthesized according to general procedure F from compound **12l** (1.0 equiv., 0.53 mmol, 0.25 g), K_2_CO_3_ (3.0 equiv., 1.6 mmol, 0.222 g), and 3-methylpiperidine (3.0 equiv., 1.6 mmol, 0.188 mL). The product was purified by column chromatography, using EtOAc/*n*-hexane (1:1, v/v) as the mobile phase. Light-yellow oil. Yield: 65%. Rf (EtOAc/*n*-hexane = 1:1, v/v) = 0.20. The product was used in the next step without further purification. 4 M HCl in dioxane (10 equiv., 0.160 mL) was added, and the reaction mixture was stirred overnight at room temperature. The next day, the solvent was evaporated under reduced pressure and the remaining residue was washed several times with Et_2_O. White waxy solid. Yield: 96%. ^1^H NMR (400 MHz, DMSO*-d*_*6*_) δ (ppm) 9.29 (d, *J* = 11.1 Hz, 1H), 8.93 (d, *J* = 10.9 Hz, 1H), 4.18 (dd, *J* = 11.5, 4.5 Hz, 2H), 3.36–3.29 (m, 3H), 3.10–2.98 (m, 3H), 2.21 (d, *J* = 13.9 Hz, 2H), 2.02–1.67 (m, 5H), 1.54 (d, *J* = 13.1 Hz, 1H), 1.35–1.20 (m, 1H), 0.94 (d, *J* = 6.6 Hz, 3H). ^13^C NMR (100 MHz, DMSO*-d*_*6*_) δ (ppm) 176.86, 160.33, 159.94, 153.58 (q, *J*_C–F_ = 36.0 Hz), 119.82 (q, *J*_C–F_ = 276.0 Hz), 97.22, 66.82, 63.26, 54.86, 49.07, 42.80, 34.16, 32.11, 31.17, 27.15, 26.87, 25.07, 19.17. ESI-HRMS ([M + H]^+^, *m*/*z*): calcd for C_17_H_23_ON_5_F_3_ 370.1849, found 370.1844. HPLC: *t*_R_ = 4.603 (method B, purity 96.11%).

4-(4-(Isobutylamino)-6-(trifluoromethyl)isoxazolo[5,4*-d*]pyrimidin-3-yl)piperidin-1-ium chloride (**25b**). Synthesized according to general procedure F from compound **12l** (1.0 equiv., 0.21 mmol, 0.101 g), K_2_CO_3_ (3.0 equiv., 0.65 mmol, 0.090 g), and isobutylamine (3.0 equiv., 0.65 mmol, 0.042 mL). The product was purified by column chromatography, using EtOAc/*n*-hexane (1:3, v/v) as the mobile phase. Light-yellow oil. Yield: 78%. Rf (EtOAc/*n*-hexane = 1:3, v/v) = 0.15. The product was used in the next step without purification. 4 M HCl in dioxane (10 equiv., 0.50 mL) was added, and the reaction mixture was stirred overnight at room temperature. The next day, the solvent was evaporated under reduced pressure and the remaining residue was washed several times with Et_2_O. White waxy solid. Yield: 92%. ^1^H NMR (400 MHz, DMSO*-d*_*6*_) δ (ppm) 8.90 (s, 1H), 8.64 (s, 1H), 8.36 (t, *J* = 5.9 Hz, 1H), 3.86 (ddd, *J* = 10.8, 7.2, 3.8 Hz, 1H), 3.44–3.36 (m, 3H), 3.13 (q, *J* = 11.3 Hz, 2H), 2.14 (d, *J* = 14.0 Hz, 2H), 2.04 (p, *J* = 6.8 Hz, 1H), 1.95 (t, *J* = 11.1 Hz, 2H), 0.91 (d, *J* = 6.7 Hz, 6H). ^13^C NMR (100 MHz, DMSO*-d*_*6*_) δ (ppm) 175.47, 160.76, 158.33, 119.32 (q, *J*_C–F_ = 278.6 Hz) 96.16, 66.82, 48.71, 42.95, 31.29, 28.27, 26.73, 20.55. ESI-HRMS ([M + H]^+^, *m*/*z*): calcd for C_15_H_21_ON_5_F_3_ 344.1693, found 344.1687. HPLC: *t*_R_ = 4.523 (method B, purity 97.56%).

### TLR7 Agonist Activity Evaluation

TLR7 agonist activities were determined using the HEK293 cell line, which was cotransfected with the hTLR7 gene and an inducible SEAP reporter gene (InvivoGen, San Diego, California, USA) as described previously.^34^ Briefly, cells were seeded in 96-well plates (3 × 10^4^ cells/well) and incubated at 5% CO_2_ and 37 °C. After 24 h, the cells were treated with the solutions of the compounds in DMSO. Imiquimod was used as a PC. The cells were incubated again for 24 h under the same conditions. The activity of SEAP in the supernatant of the cells was measured using the QUANTI-Blue reagent (InvivoGen, San Diego, California, USA), according to the manufacturer's protocol. EC_50_ values were determined using GraphPad Prism 9.2.0. software (San Diego, California, USA) using the nonlinear regression curve fit and four-parameter logistic equation. All experiments were performed in three independent biological replicates. Values are given as averages from the independent measurements with standard deviations calculated.

### TLR8 Agonist Activity Evaluation

TLR8 agonist activities were determined using the HEK293 cell line, which was cotransfected with the hTLR8 gene and an inducible SEAP reporter gene (InvivoGen, San Diego, California, USA), as described previously.^34^ In brief, the cells were seeded in 96-well plates (3 × 10^4^ cells/well). After 24 h incubation at 5% CO_2_ and 37 °C, the cells were treated with solutions of the compounds in DMSO. Resiquimod was used as a PC. The cells were incubated again for 24 h under the same conditions. The activity of SEAP in the cell supernatants was measured using the QUANTI-Blue reagent (InvivoGen, San Diego, California, USA), according to the manufacturer's protocol.

### Cytotoxicity

The cytotoxicity of the compounds was determined using the MTS cell proliferation assay (CellTiter 96 AQueous One Solution Cell Proliferation Assay, Promega, Wisconsin, USA), according to the manufacturer's protocol on the HEK293 cell line, cotransfected with the hTLR7 gene (InVivoGen). Cells were seeded in 96-well plates (1.5 × 10^4^ cells/well). After 24 h incubation at 5% CO_2_ and 37 °C, the cells were treated either with DMSO (negative control) or with solutions of the tested compounds in DMSO. After 24 h, MTS reagent (CellTiter 96 AQueous One Solution Cell Proliferation Assay, Promega, Wisconsin, USA) was added. Cells were incubated for another 2 h, and then absorbance was measured at 490 nm.

### Quantification of Cytokine Secretion

The BD Human Cytometric Bead Array (BD Biosciences, California, USA) was used to assay the protein levels of IL-1β, IL-6, IL-8, IL-10, IL-12p70, and TNF-α in the cell culture supernatant, according to the manufacturer’s protocol (BD Biosciences). PBMCs were isolated from buffy coats obtained at the Blood Transfusion Centre of Slovenia. Cells were seeded into 24-well plates (5 × 10^5^ cells/well in 500 mL of RPMI medium, Sigma-Aldrich, St. Louis, Missouri, USA) and treated with the selected TLR7 agonist. For a PC, imiquimod was used (final concentration is 10 μg/mL) and the negative control were cells treated with DMSO. Cells were centrifuged after 24 h incubation at 5% CO_2_ and 37 °C, and cell culture supernatants were used for further analysis. In an assay tube, 50 mL of the Capture Beads, 50 mL of the Detection Reagent, and 50 mL of sample (fivefold dilution) were mixed and incubated for 3 h at room temperature and protected from light. Samples were then washed with 1 mL of wash buffer and centrifuged at 200*g* for 5 min. The supernatant was carefully aspirated and discarded from each assay tube. The bead pellet was resuspended by adding 300 mL of wash buffer. Flow cytometry was performed using a FACSCalibur system (Becton Dickinson, Inc., New Jersey, USA). A standard curve was prepared by serial dilutions of standards and used for determination of cytokine concentrations in supernatants.

### Binding Mode Studies

AutoDock Vina 1.1 with integrated LigandScout 4.3 was used to dock compound **21a** to the active site of TLR7 (pdb code: 6LVX). The docking process was performed with the following default settings: exhaustiveness: 8, max. number of modes: 9, max. energy difference: 3. Poses for compound **21a** were prioritized based on AutoDock’s binding affinity score. Protein–ligand interactions were studied using the Protein–Ligand Interaction Profiler (PLIP),^35^ a web service for detection and visualization of relevant noncovalent protein–ligand contacts in 3D structures. The input was a protein–ligand complex file generated from the previously described docking.

## Supporting Information Available

^1^H NMR spectra of all final compounds, HPLC spectra, and additional experimental details (PDF)

## Abbreviations

DAMP: damage-associated molecular patterns
DMAP: 4*-*dimethylaminopyridine
DMF: dimethylformamide
DMSO: dimethyl sulfoxide
EC50: half maximal effective concentration
IL: interleukin
MyD88: myeloid differentiation primary response protein 88
MTS: 3-(4,5*-*dimethylthiazol-2-yl)-5-(3-carboxymethoxyphenyl)-2-(4-sulfophenyl)-2*H*-tetrazolium
PAMPs: pathogen-associated molecular patterns
PBMC: peripheral blood mononuclear cell
PLIP: Protein–Ligand Interaction Profiler
SEAP: secreted embryonic alkaline phosphatase
ssRNA: single-stranded RNA
TBTU: 2-(1*H*-benzotriazole-1-yl)-1,1,3,3-tetramethylaminium tetrafluoroborate
Th1: type 1 T helper cells
THF: tetrahydrofuran
TLR: Toll-like receptor
TNFα: tumor necrosis factor alpha
