# Supplementary material for: Structural Optimization and Biological Evaluation of Isoxazolo[5,4-d]pyrimidines as Selective Toll-Like Receptor 7 Agonists
Source: ACS Omega. 2024 Jan 4;9(2):2362–82. doi: 10.1021/acsomega.3c06343 (PMC10795023; doi:10.1021/acsomega.3c06343)
Supplement: Supplementary file 1 — ao3c06343_si_001.pdf [file ao3c06343_si_001.pdf]

Supporting Information

Structural Optimization and Biological Evaluation of Isoxazolo[5,4-*d*]pyrimidines as Selective Toll-like receptor 7 Agonists

Nika Strašek Benedik,[†] Ana Dolšak,[†] Urban Švajger,[†,‡] Izidor Sosič,[†] Stanislav Gobec,[†] and Matej Sova*[,†]

[†]Faculty of Pharmacy, Department of Pharmaceutical Chemistry, University of Ljubljana, Aškerčeva 7, Ljubljana 1000, Slovenia

[‡]Blood Transfusion Centre of Slovenia, Šlajmerjeva 6, Ljubljana 1000, Slovenia

*Email: matej.sova@ffa.uni-lj.si.

Table of contents

1. ^{1}H NMR and HPLC spectra of final compounds..S3
2. Biological evaluation data..S93
2.1. EC_{50} curves of final compounds..S93
2.2. Cytotoxicity..S96

1. NMR spectra of final compounds

14a

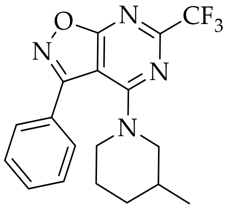

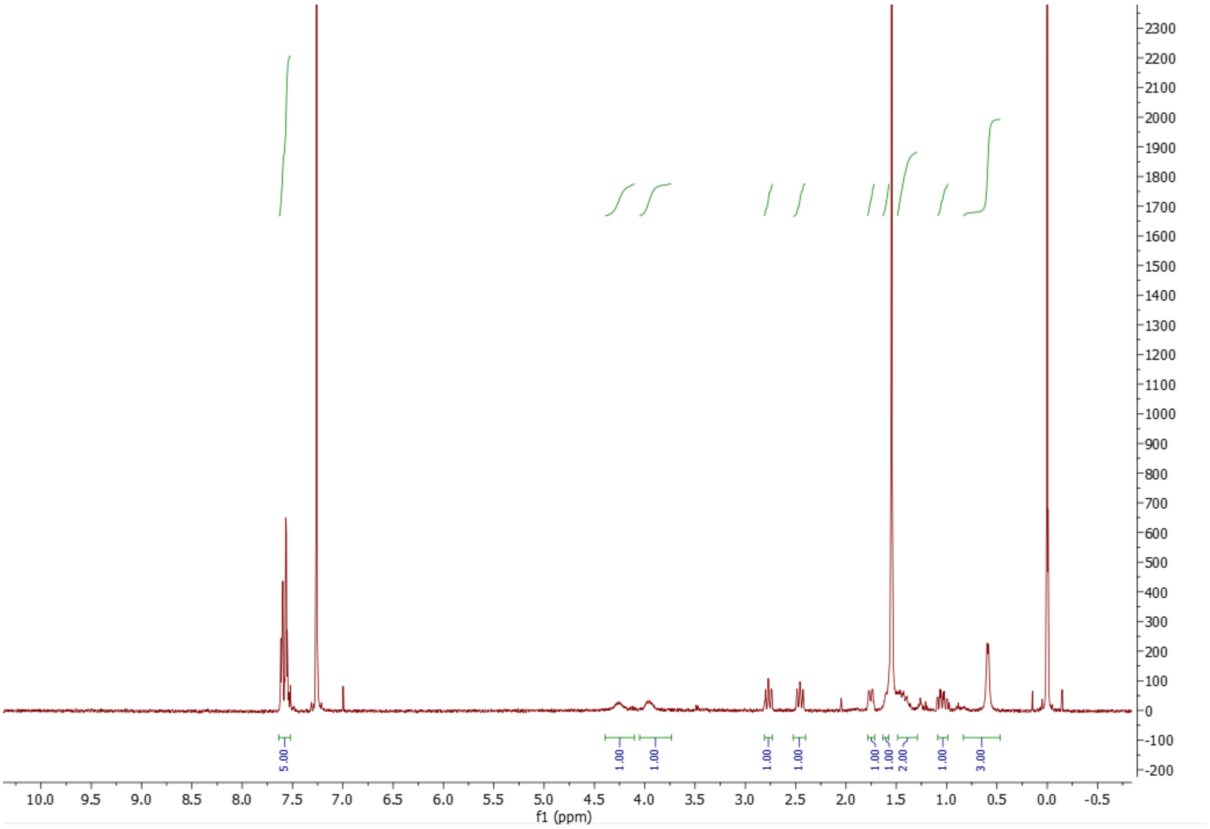

Figure S1: ^{1}H NMR of **14a**.

Chromatogram and Results

Injection Details

Injection Name:	**SLP-6**	*Run Time (min):*	**15,00**
Vial Number:	**GA3**	*Injection Volume:*	**5,00**
Injection Type:	**Unknown**	*Channel:*	**UV_VIS_2**
Calibration Level:		*Wavelength:*	**254**
Instrument Method:	**Metoda IDO**	*Bandwidth:*	**16**
Processing Method:	**Basic Quantitative**	*Dilution Factor:*	**1,0000**
Injection Date/Time:	**16.jun..21 12:49**	*Sample Weight:*	**1,0000**

Chromatogram

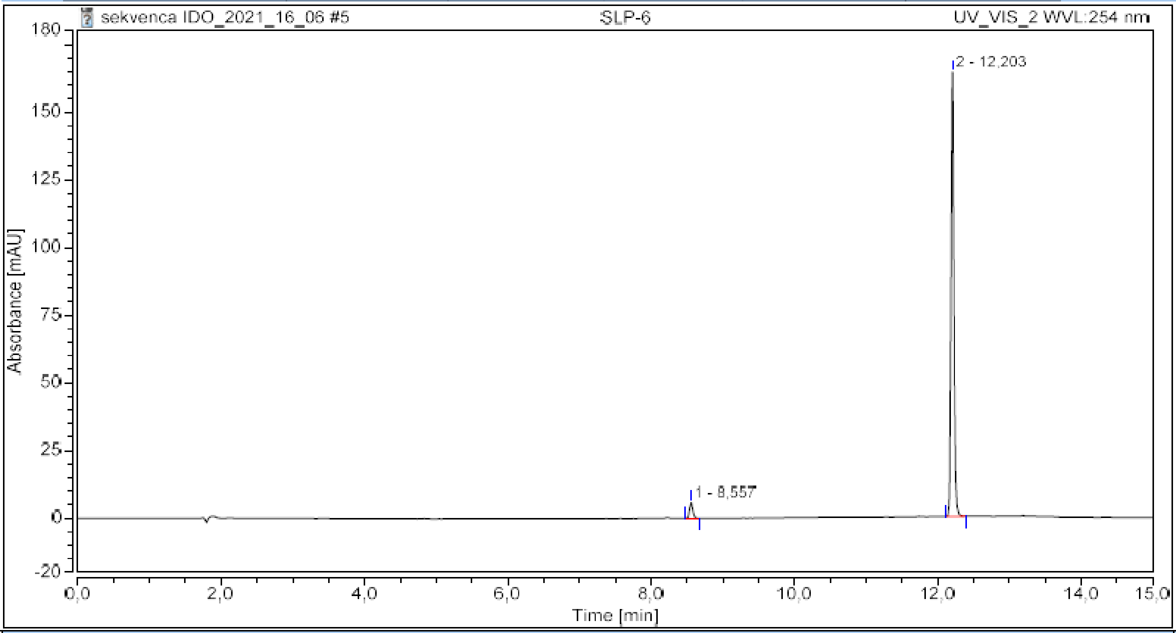

Integration Results

No.	Peak Name	Retention Time min	Area mAU*min	Height mAU	Relative Area %	Relative Height %	Amount n.a.
1		8,557	0,288	5,956	3,51	3,50	n.a.
2		12,203	7,936	164,208	96,49	96,50	n.a.
**Total:**			**8,225**	**170,164**	**100,00**	**100,00**	

Figure S2: HPLC of **14a**

14b

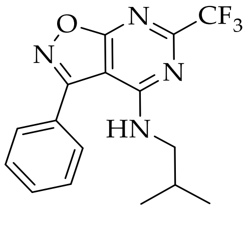

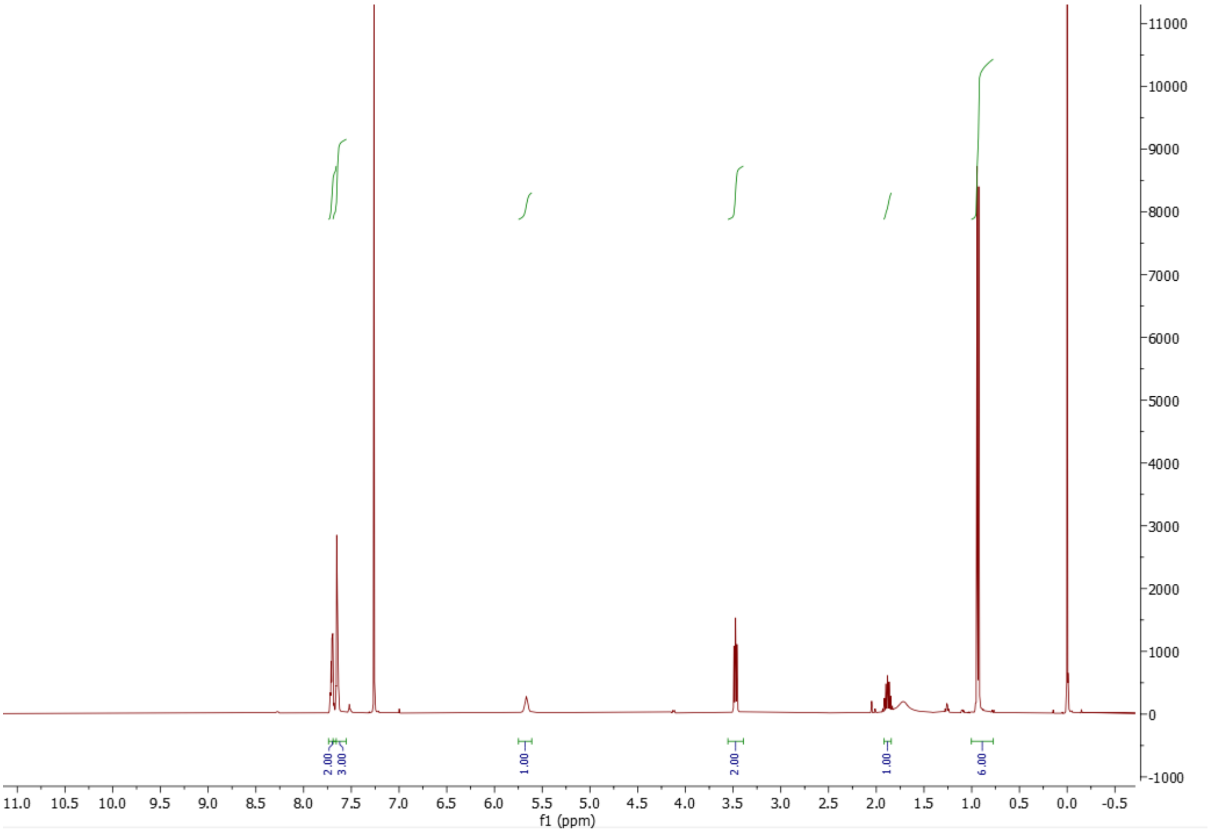

Figure S3: ^{1}H NMR of **14b**.

Chromatogram and Results

Injection Details

Injection Name:	**SLP-7**	*Run Time (min):*	**15,00**
Vial Number:	**GA4**	*Injection Volume:*	**5,00**
Injection Type:	**Unknown**	*Channel:*	**UV_VIS_2**
Calibration Level:		*Wavelength:*	**254**
Instrument Method:	**Metoda IDO**	*Bandwidth:*	**16**
Processing Method:	**Basic Quantitative**	*Dilution Factor:*	**1,0000**
Injection Date/Time:	**16.jun..21 13:08**	*Sample Weight:*	**1,0000**

Chromatogram

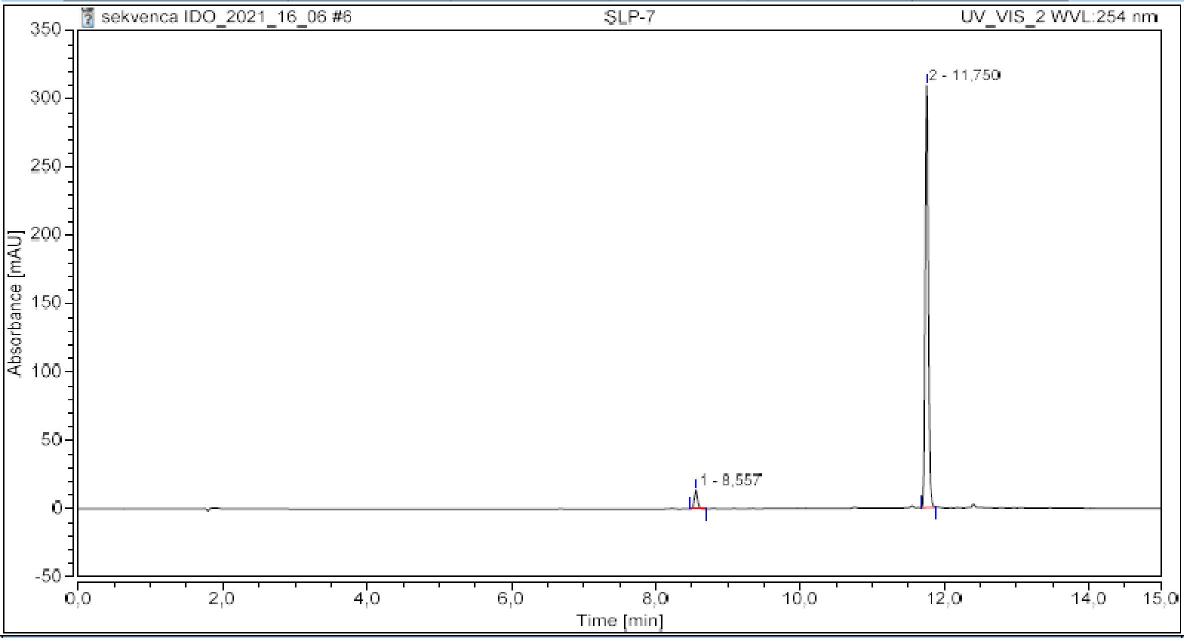

Integration Results

No.	Peak Name	Retention Time min	Area mAU*min	Height mAU	Relative Area %	Relative Height %	Amount n.a.
1		8,557	0,669	13,700	4,26	4,25	n.a.
2		11,750	15,022	308,965	95,74	95,75	n.a.
**Total:**			**15,691**	**322,665**	**100,00**	**100,00**	

Figure S4: HPLC of **14b**.

15a

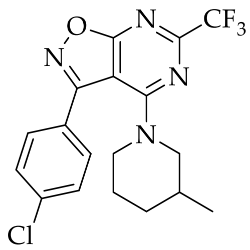

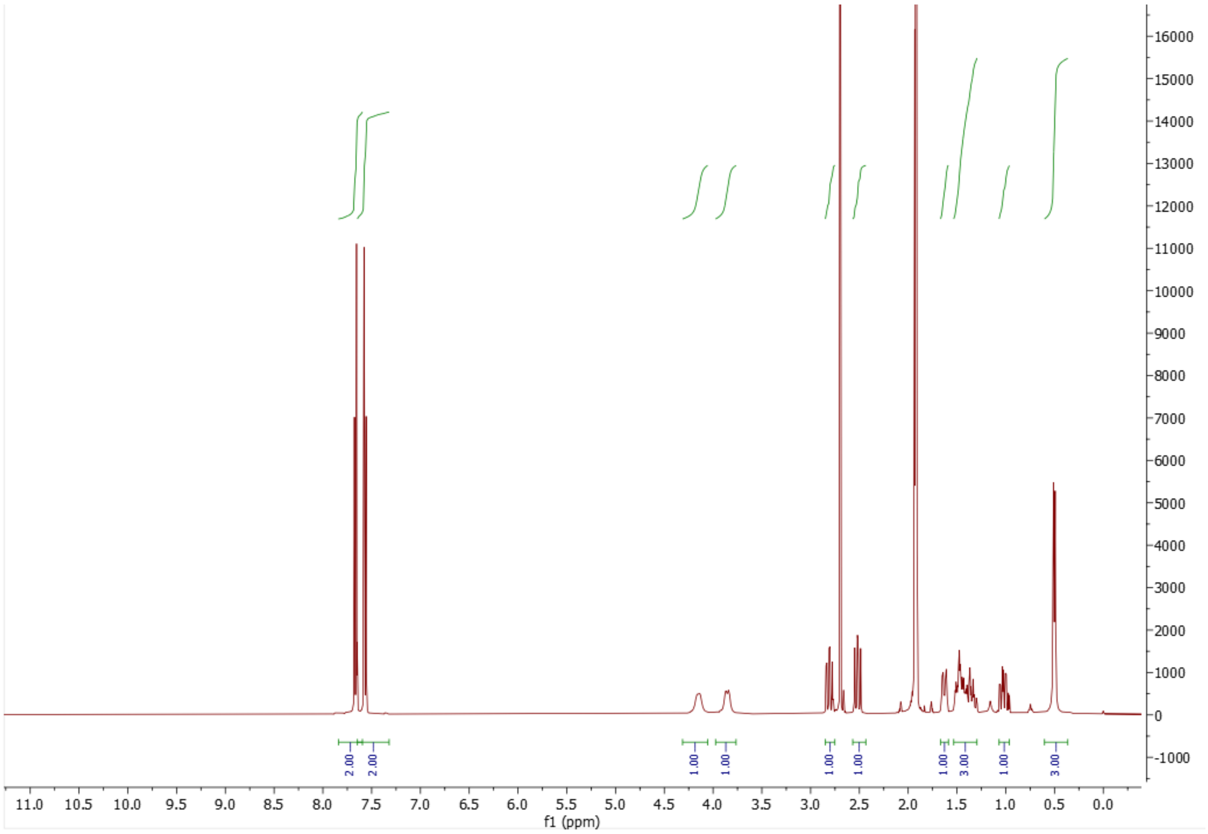

Figure S5: ^{1}H NMR of **15a**.

Chromatogram and Results

Injection Details

Injection Name:	**SST-56**	*Run Time (min):*	**12,00**
Vial Number:	**GA5**	*Injection Volume:*	**2,00**
Injection Type:	**Unknown**	*Channel:*	**UV_VIS_2**
Calibration Level:		*Wavelength:*	**254**
Instrument Method:	**Metoda_uplc**	*Bandwidth:*	**16**
Processing Method:	**Basic Quantitative**	*Dilution Factor:*	**1,0000**
Injection Date/Time:	**04.nov..21 11:33**	*Sample Weight:*	**1,0000**

Chromatogram

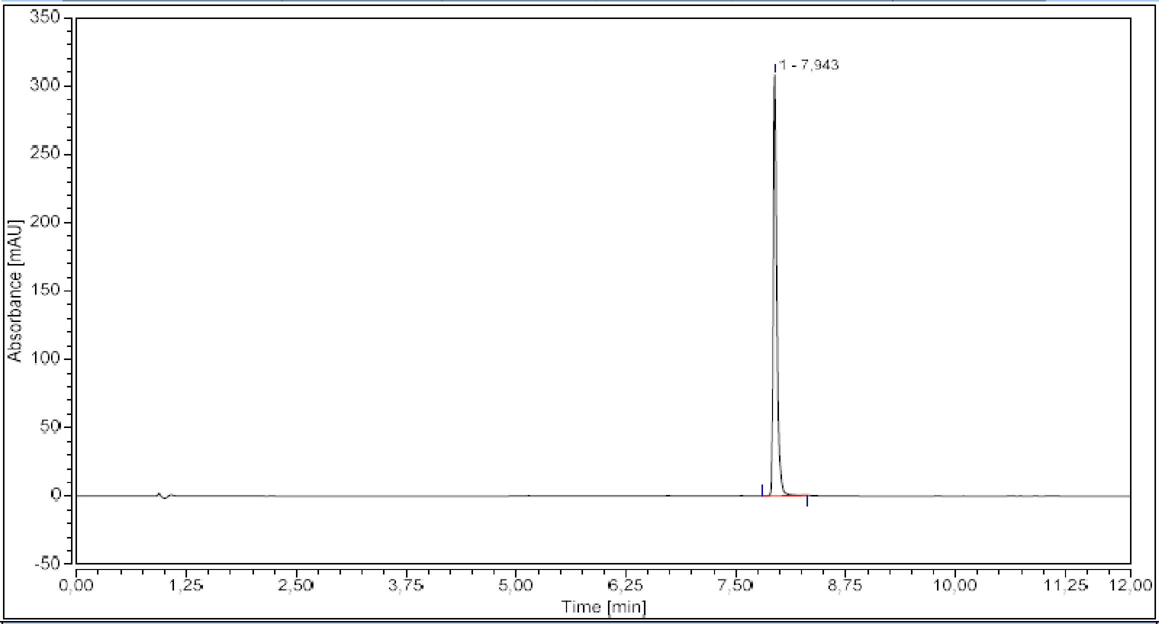

Integration Results

No.	Peak Name	Retention Time min	Area mAU*min	Height mAU	Relative Area %	Relative Height %	Amount n.a.
1		7,943	14,451	307,763	100,00	100,00	n.a.
**Total:**			**14,451**	**307,763**	**100,00**	**100,00**	

Figure S6: HPLC of **15a**.

15b

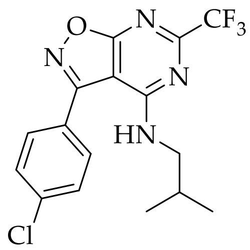

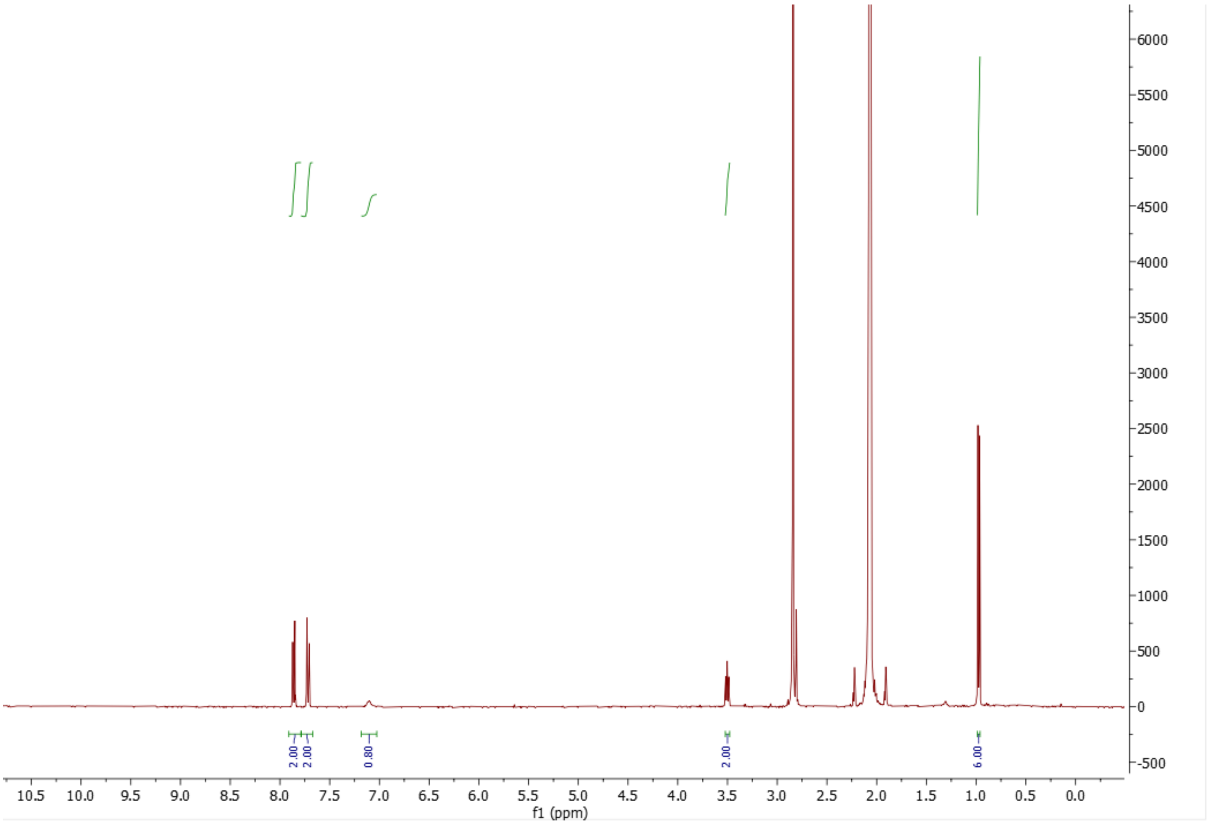

Figure S7: ^{1}H NMR of **15b**.

Chromatogram and Results

Injection Details

Injection Name:	**SST-57**	*Run Time (min):*	**12,00**
Vial Number:	**GA6**	*Injection Volume:*	**2,00**
Injection Type:	**Unknown**	*Channel:*	**UV_VIS_2**
Calibration Level:		*Wavelength:*	**254**
Instrument Method:	**Metoda_uplc**	*Bandwidth:*	**16**
Processing Method:	**Basic Quantitative**	*Dilution Factor:*	**1,0000**
Injection Date/Time:	**04.nov..21 11:51**	*Sample Weight:*	**1,0000**

Chromatogram

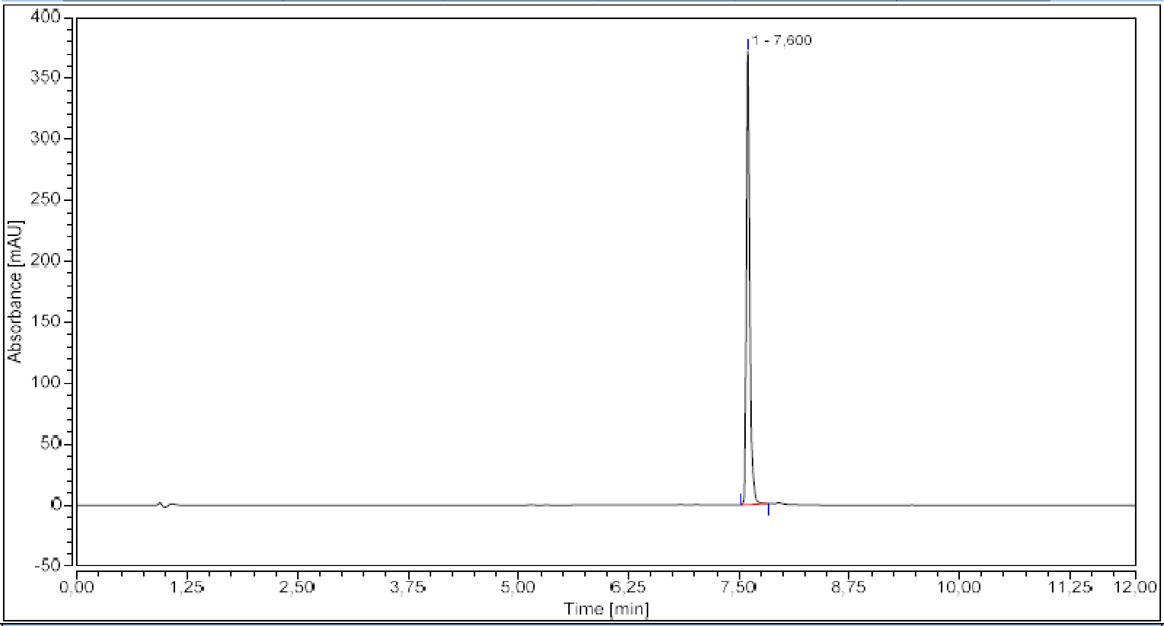

Integration Results

No.	Peak Name	Retention Time min	Area mAU*min	Height mAU	Relative Area %	Relative Height %	Amount n.a.
1		7,600	16,289	372,150	100,00	100,00	n.a.
**Total:**			**16,289**	**372,150**	**100,00**	**100,00**	

Figure S8: HPLC of **15b**.

16a

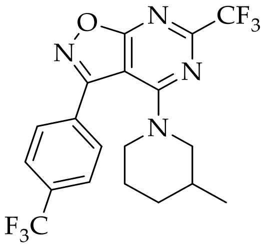

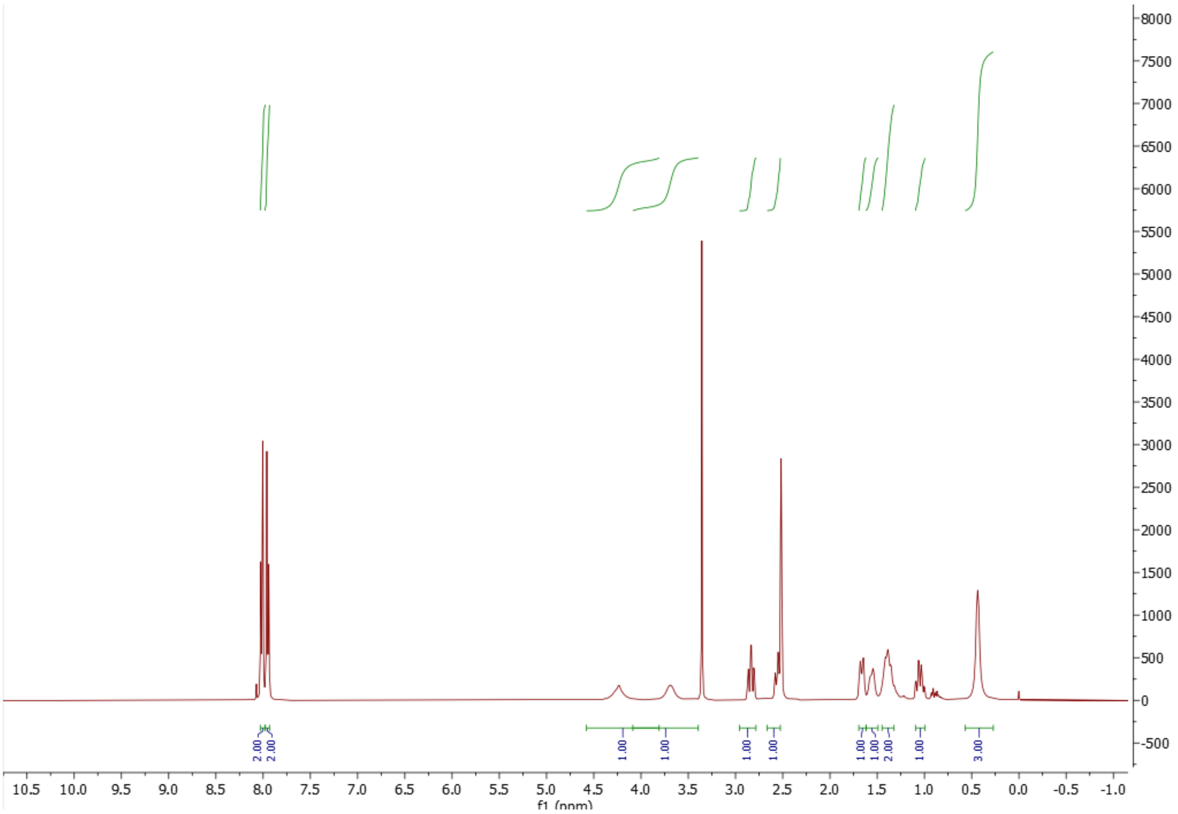

Figure S9: ^{1}H NMR of **16a**.

Chromatogram and Results

Injection Details

Injection Name:	**SST5A**	*Run Time (min):*	**8,00**
Vial Number:	**RA4**	*Injection Volume:*	**2,00**
Injection Type:	**Spiked**	*Channel:*	**UV_VIS_2**
Calibration Level:		*Wavelength:*	**254**
Instrument Method:	**Metoda_uplc**	*Bandwidth:*	**16**
Processing Method:	**Basic Quantitative**	*Dilution Factor:*	**1,0000**
Injection Date/Time:	**30.avg..21 13:23**	*Sample Weight:*	**1,0000**

Chromatogram

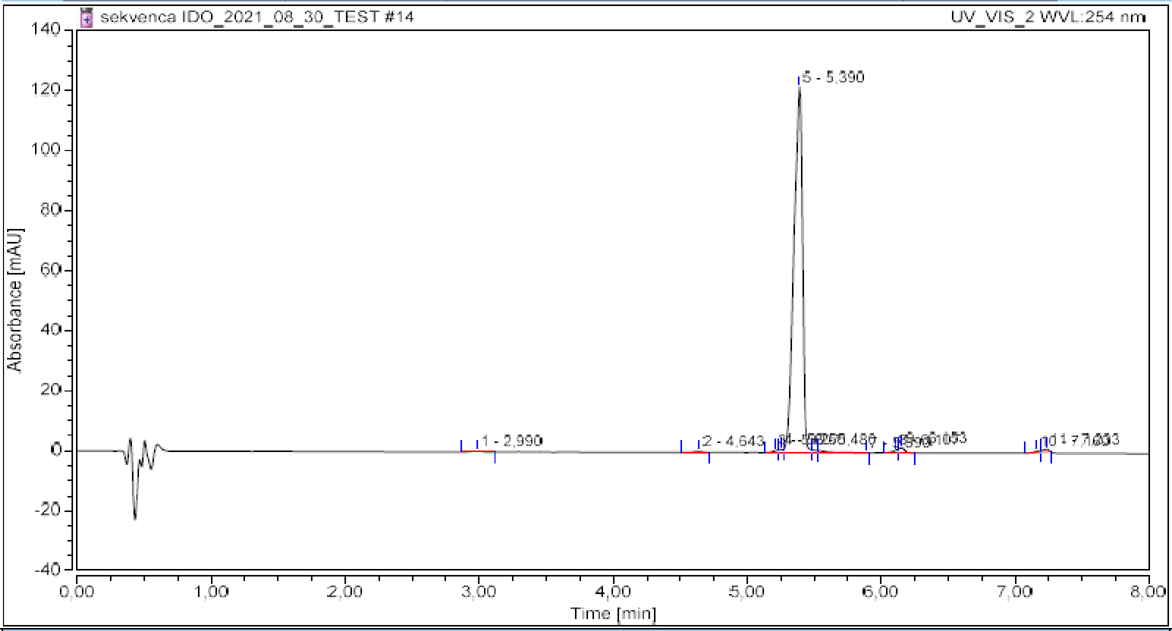

Integration Results

No.	Peak Name	Retention Time min	Area mAU*min	Height mAU	Relative Area %	Relative Height %	Amount n.a.
1		2,990	0,022	0,259	0,24	0,20	n.a.
2		4,643	0,020	0,271	0,22	0,21	n.a.
3		5,207	0,028	0,600	0,30	0,47	n.a.
4		5,250	0,043	1,079	0,46	0,84	n.a.
5		5,390	8,848	121,753	95,46	94,55	n.a.
6		5,480	0,038	1,018	0,41	0,79	n.a.
7		5,890	0,083	0,045	0,89	0,03	n.a.
8		6,107	0,039	0,865	0,42	0,67	n.a.
9		6,153	0,083	1,629	0,90	1,27	n.a.
10		7,160	0,021	0,363	0,22	0,28	n.a.
11		7,233	0,044	0,885	0,48	0,69	n.a.
**Total:**			**9,269**	**128,766**	**100,00**	**100,00**	

Figure S20: HPLC of **16a**.

16b

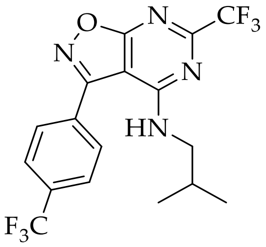

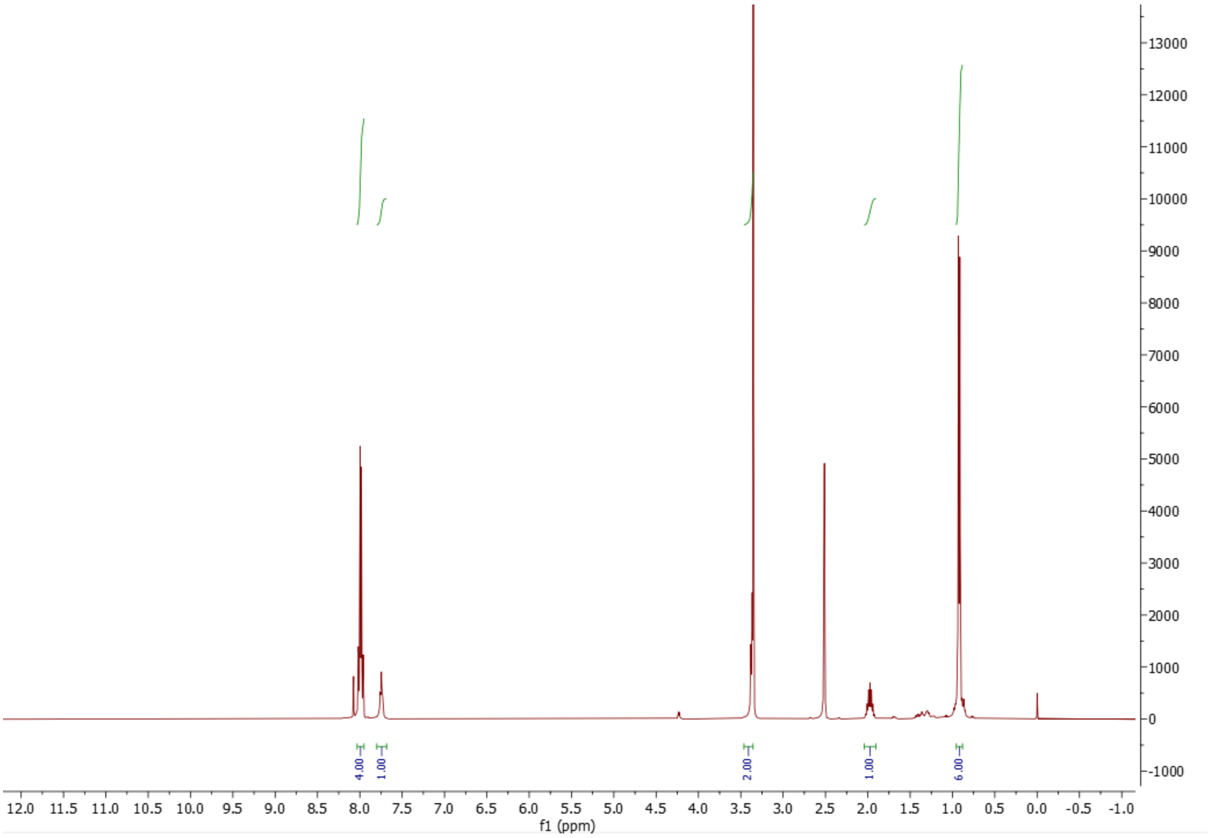

Figure S31: ^{1}H NMR of **16b**.

Chromatogram and Results

Injection Details

Injection Name:	**SST5B**	*Run Time (min):*	**8,00**
Vial Number:	**RA6**	*Injection Volume:*	**2,00**
Injection Type:	**Spiked**	*Channel:*	**UV_VIS_2**
Calibration Level:		*Wavelength:*	**254**
Instrument Method:	**Metoda_uplc**	*Bandwidth:*	**16**
Processing Method:	**Basic Quantitative**	*Dilution Factor:*	**1,0000**
Injection Date/Time:	**30.avg..21 13:50**	*Sample Weight:*	**1,0000**

Chromatogram

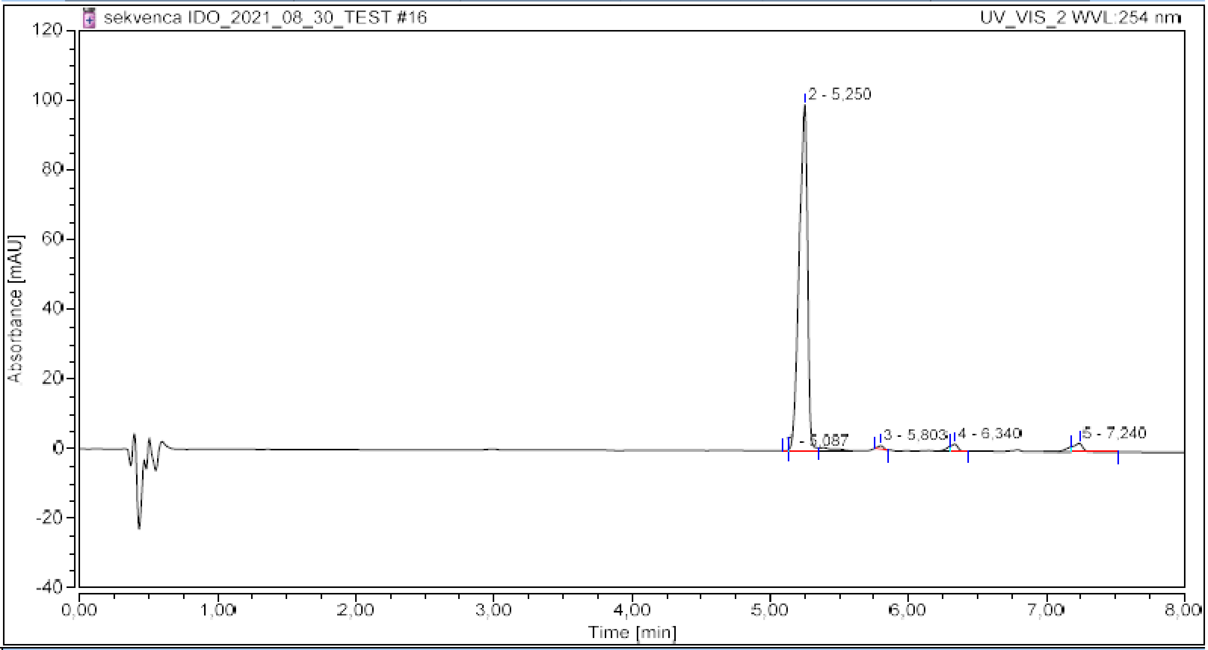

Integration Results

No.	Peak Name	Retention Time min	Area mAU*min	Height mAU	Relative Area %	Relative Height %	Amount n.a.
1		5,087	0,003	0,000	0,05	0,00	n.a.
2		5,250	7,023	99,459	95,49	94,67	n.a.
3		5,803	0,042	1,014	0,57	0,97	n.a.
4		6,340	0,106	2,099	1,44	2,00	n.a.
5		7,240	0,180	2,490	2,45	2,37	n.a.
**Total:**			**7,355**	**105,062**	**100,00**	**100,00**	

Figure S42: HPLC of **16b**.

17

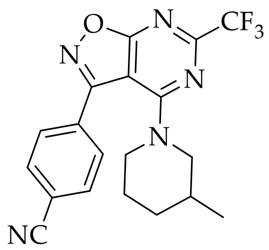

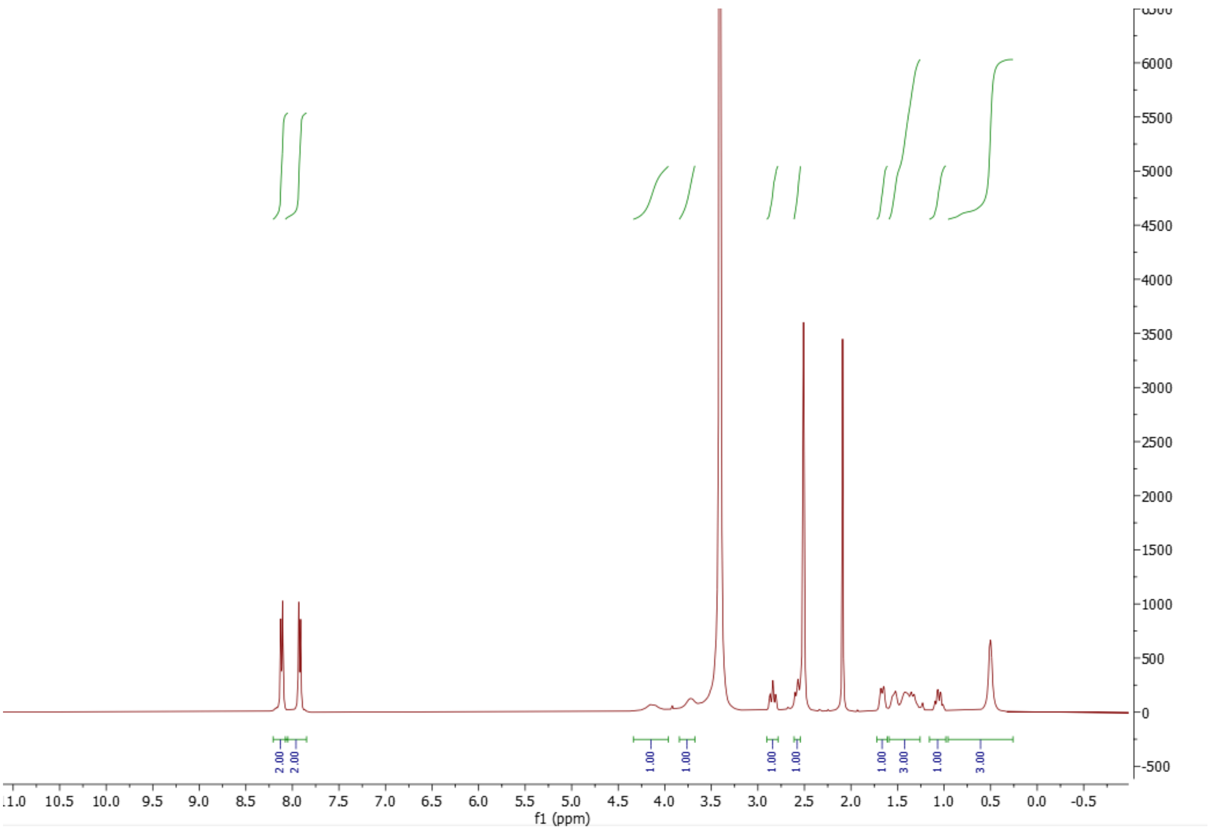

Figure S53: ^{1}H NMR of **17**.

Chromatogram and Results

Injection Details

Injection Name:	**SLP-15b**	*Run Time (min):*	**8,00**
Vial Number:	**RB2**	*Injection Volume:*	**2,00**
Injection Type:	**Unknown**	*Channel:*	**UV_VIS_2**
Calibration Level:		*Wavelength:*	**254**
Instrument Method:	**Metoda_uplc**	*Bandwidth:*	**16**
Processing Method:	**Basic Quantitative**	*Dilution Factor:*	**1,0000**
Injection Date/Time:	**30.avg..21 14:18**	*Sample Weight:*	**1,0000**

Chromatogram

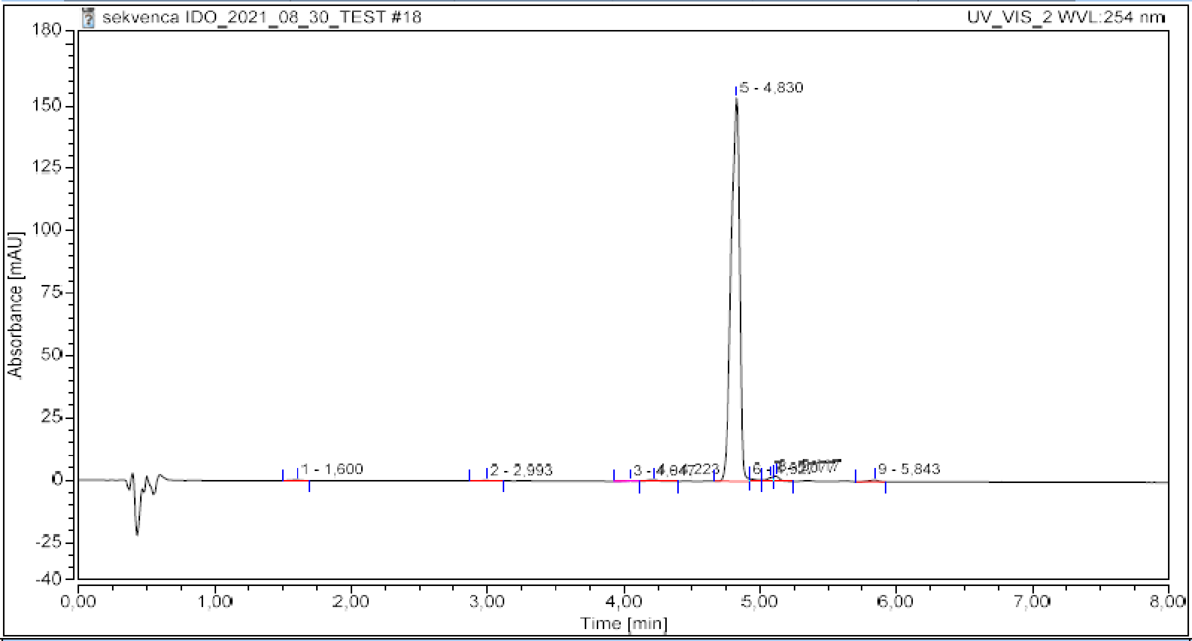

Integration Results

No.	Peak Name	Retention Time min	Area mAU*min	Height mAU	Relative Area %	Relative Height %	Amount n.a.
1		1,600	0,021	0,250	0,18	0,16	n.a.
2		2,993	0,023	0,257	0,20	0,16	n.a.
3		4,047	0,008	0,114	0,07	0,07	n.a.
4		4,223	0,065	0,652	0,56	0,41	n.a.
5		4,830	11,190	153,642	96,41	95,93	n.a.
6		4,927	0,061	0,962	0,52	0,60	n.a.
7		5,077	0,076	1,469	0,66	0,92	n.a.
8		5,117	0,107	2,110	0,93	1,32	n.a.
9		5,843	0,055	0,707	0,48	0,44	n.a.
**Total:**			**11,607**	**160,163**	**100,00**	**100,00**	

Figure S64: HPLC of **17**.

18

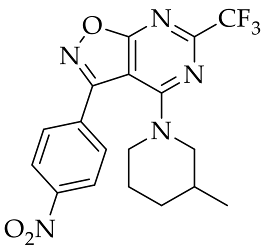

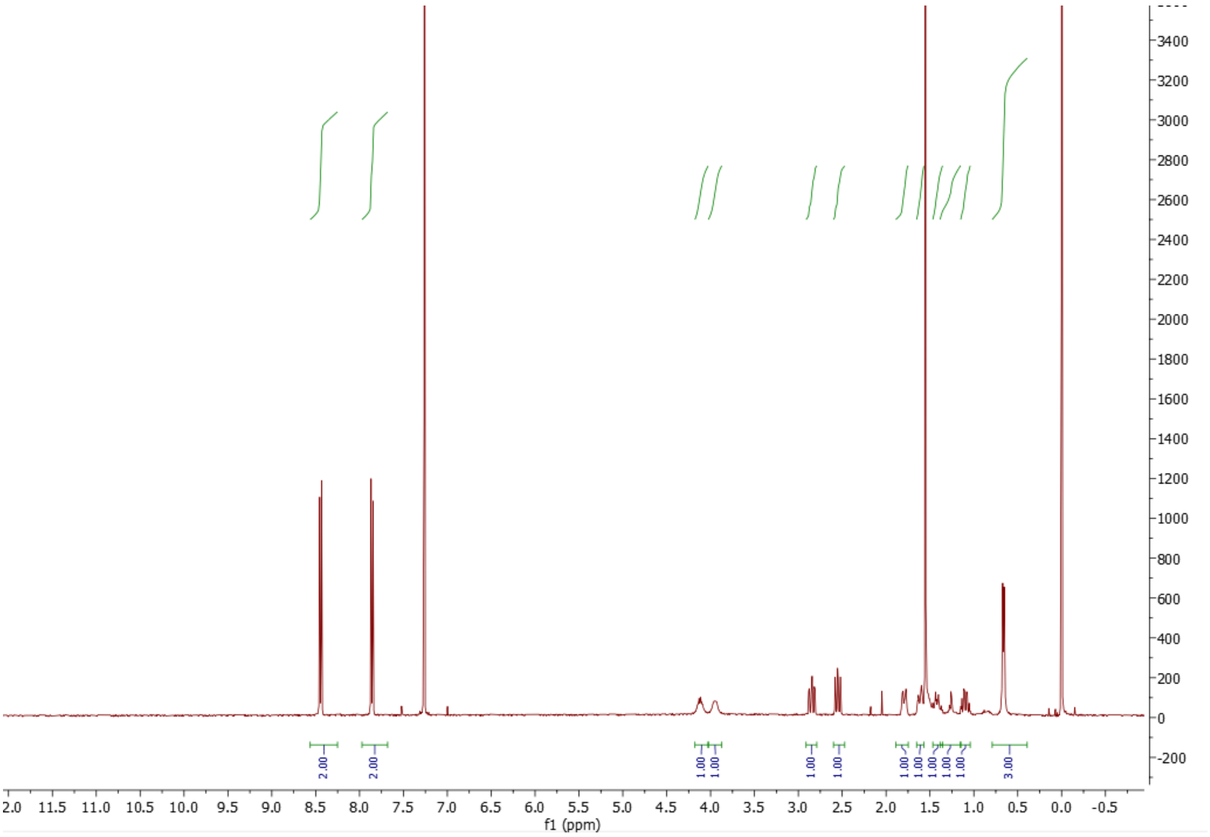

Figure S75: ^{1}H NMR of **18**.

Chromatogram and Results

Injection Details

Injection Name:	**SST-16**	*Run Time (min):*	**12,00**
Vial Number:	**GA2**	*Injection Volume:*	**2,00**
Injection Type:	**Unknown**	*Channel:*	**UV_VIS_2**
Calibration Level:		*Wavelength:*	**254**
Instrument Method:	**Metoda_uplc**	*Bandwidth:*	**16**
Processing Method:	**Basic Quantitative**	*Dilution Factor:*	**1,0000**
Injection Date/Time:	**04.nov..21 10:39**	*Sample Weight:*	**1,0000**

Chromatogram

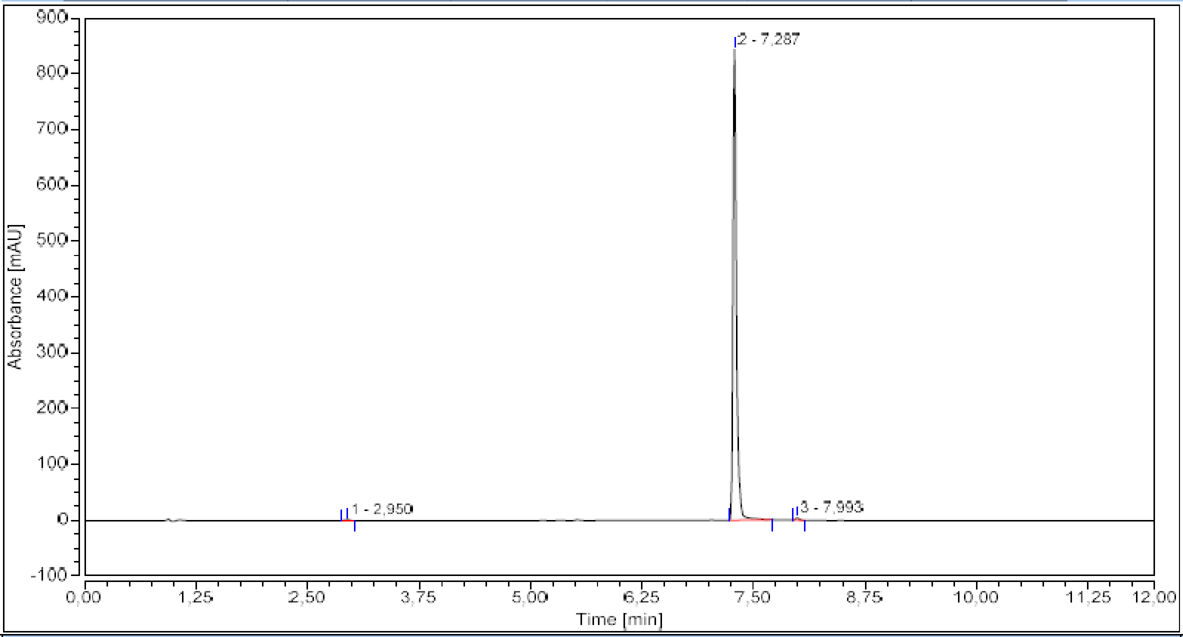

Integration Results

No.	Peak Name	Retention Time min	Area mAU*min	Height mAU	Relative Area %	Relative Height %	Amount n.a.
1		2,950	0,104	1,622	0,28	0,19	n.a.
2		7,287	36,916	842,730	99,26	99,33	n.a.
3		7,993	0,173	4,090	0,47	0,48	n.a.
**Total:**			**37,193**	**848,443**	**100,00**	**100,00**	

Figure S86: HPLC of **18**.

19a

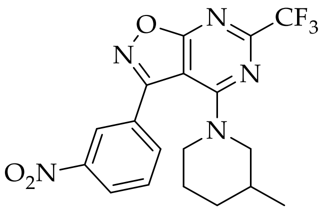

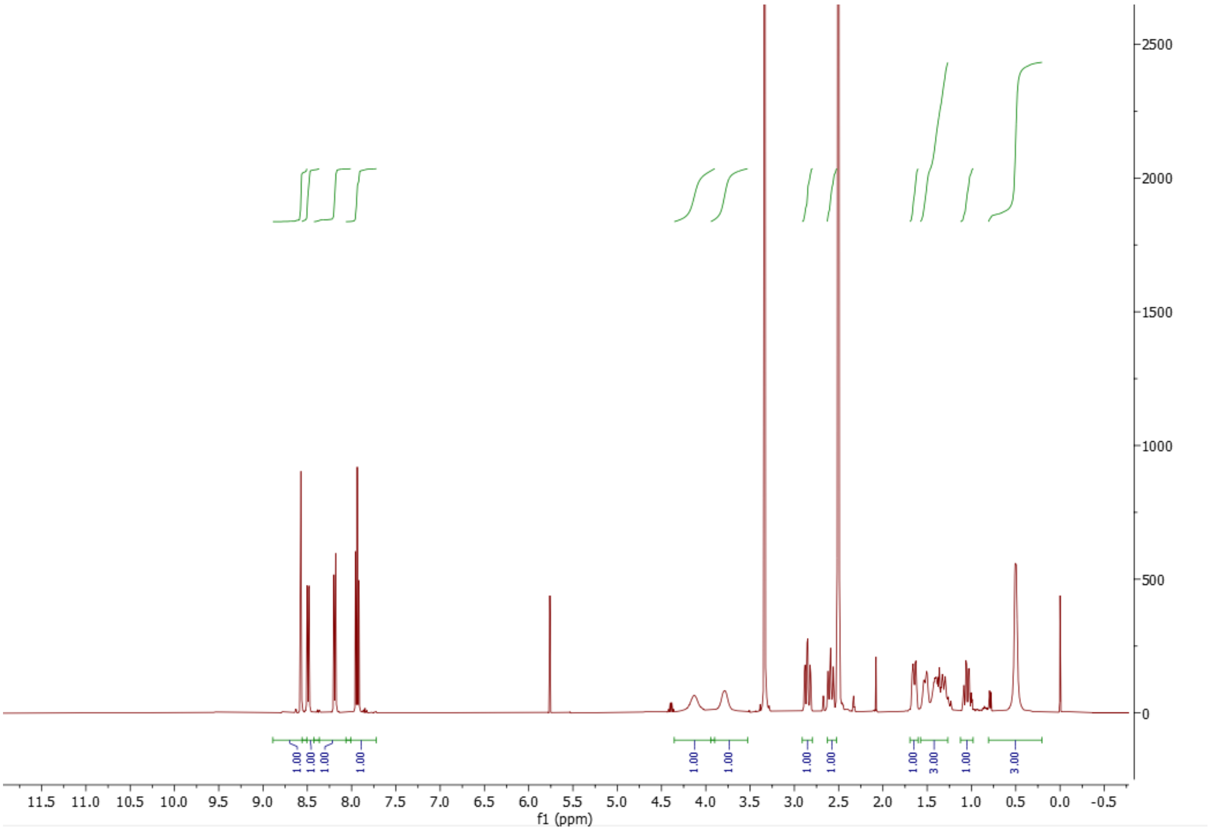

Figure S97: ^{1}H NMR of **19a.**

Chromatogram and Results

Injection Details

Injection Name:	**SST-66**	*Run Time (min):*	**12,00**
Vial Number:	**GA7**	*Injection Volume:*	**2,00**
Injection Type:	**Unknown**	*Channel:*	**UV_VIS_2**
Calibration Level:		*Wavelength:*	**254**
Instrument Method:	**Metoda_uplc**	*Bandwidth:*	**16**
Processing Method:	**Basic Quantitative**	*Dilution Factor:*	**1,0000**
Injection Date/Time:	**04.nov..21 12:08**	*Sample Weight:*	**1,0000**

Chromatogram

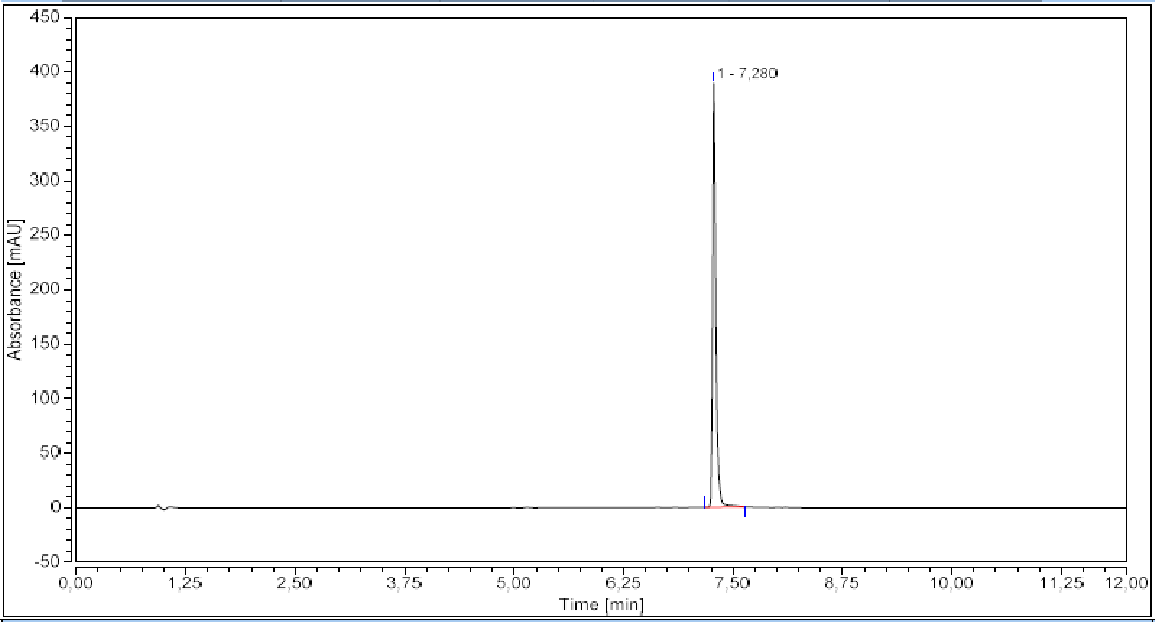

Integration Results

No.	Peak Name	Retention Time min	Area mAU*min	Height mAU	Relative Area %	Relative Height %	Amount n.a.
1		7,280	16,997	388,808	100,00	100,00	n.a.
**Total:**			**16,997**	**388,808**	**100,00**	**100,00**	

Figure S108: HPLC of **19a**.

19b

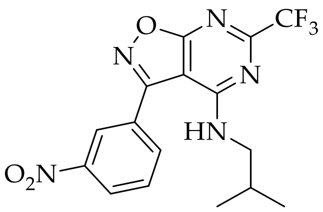

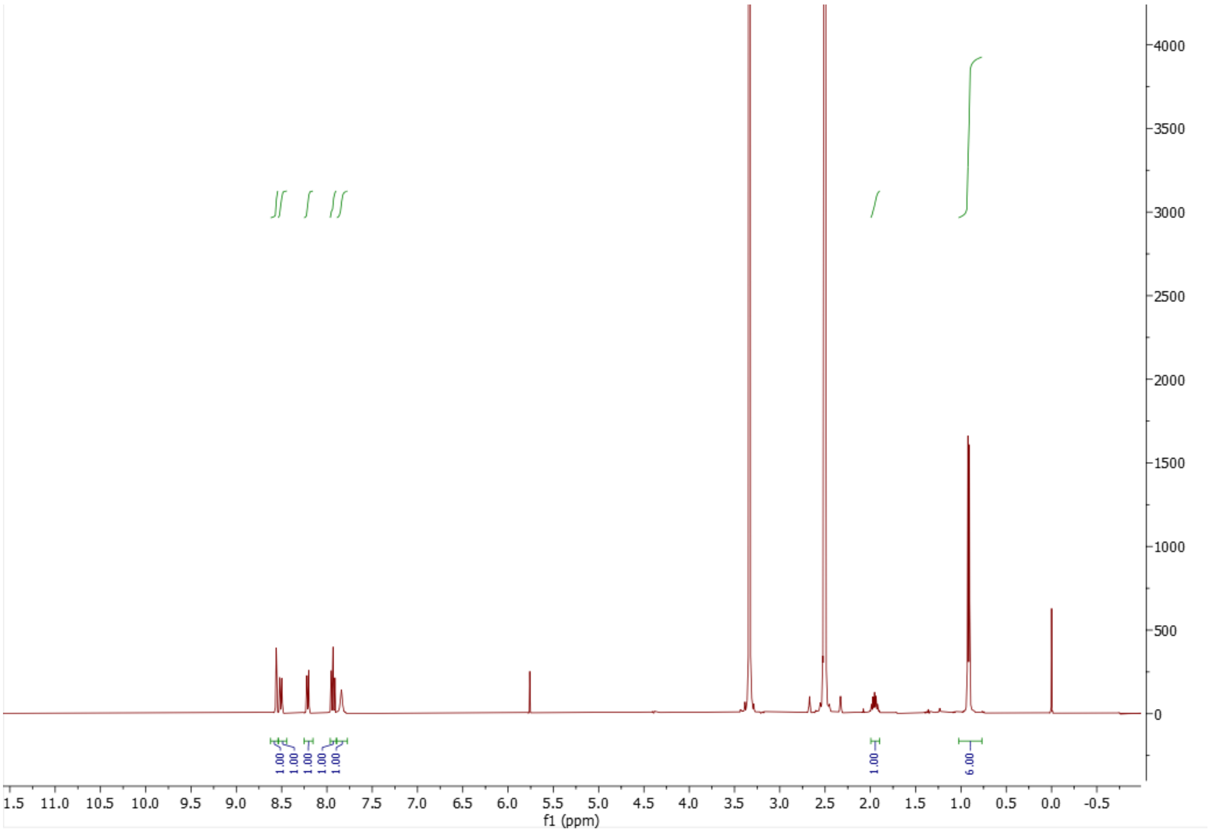

Figure S119: ^{1}H NMR of **19b**.

Chromatogram and Results

Injection Details

Injection Name:	**SST-67**	*Run Time (min):*	**12,00**
Vial Number:	**GA8**	*Injection Volume:*	**2,00**
Injection Type:	**Unknown**	*Channel:*	**UV_VIS_2**
Calibration Level:		*Wavelength:*	**254**
Instrument Method:	**Metoda_uplc**	*Bandwidth:*	**16**
Processing Method:	**Basic Quantitative**	*Dilution Factor:*	**1,0000**
Injection Date/Time:	**04.nov..21 12:26**	*Sample Weight:*	**1,0000**

Chromatogram

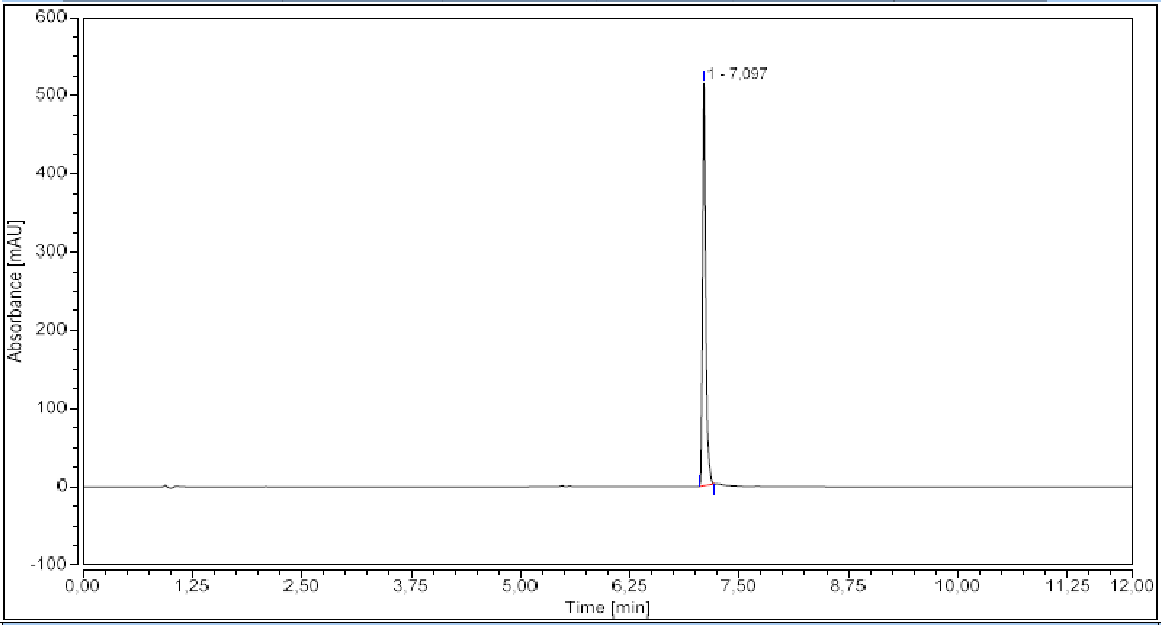

Integration Results

No.	Peak Name	Retention Time min	Area mAU*min	Height mAU	Relative Area %	Relative Height %	Amount n.a.
1		7,097	21,725	514,509	100,00	100,00	n.a.
**Total:**			**21,725**	**514,509**	**100,00**	**100,00**	

Figure S20: HPLC of **19b**.

20a

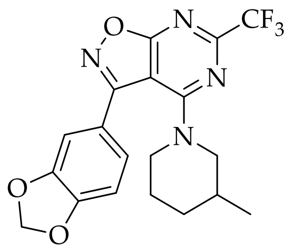

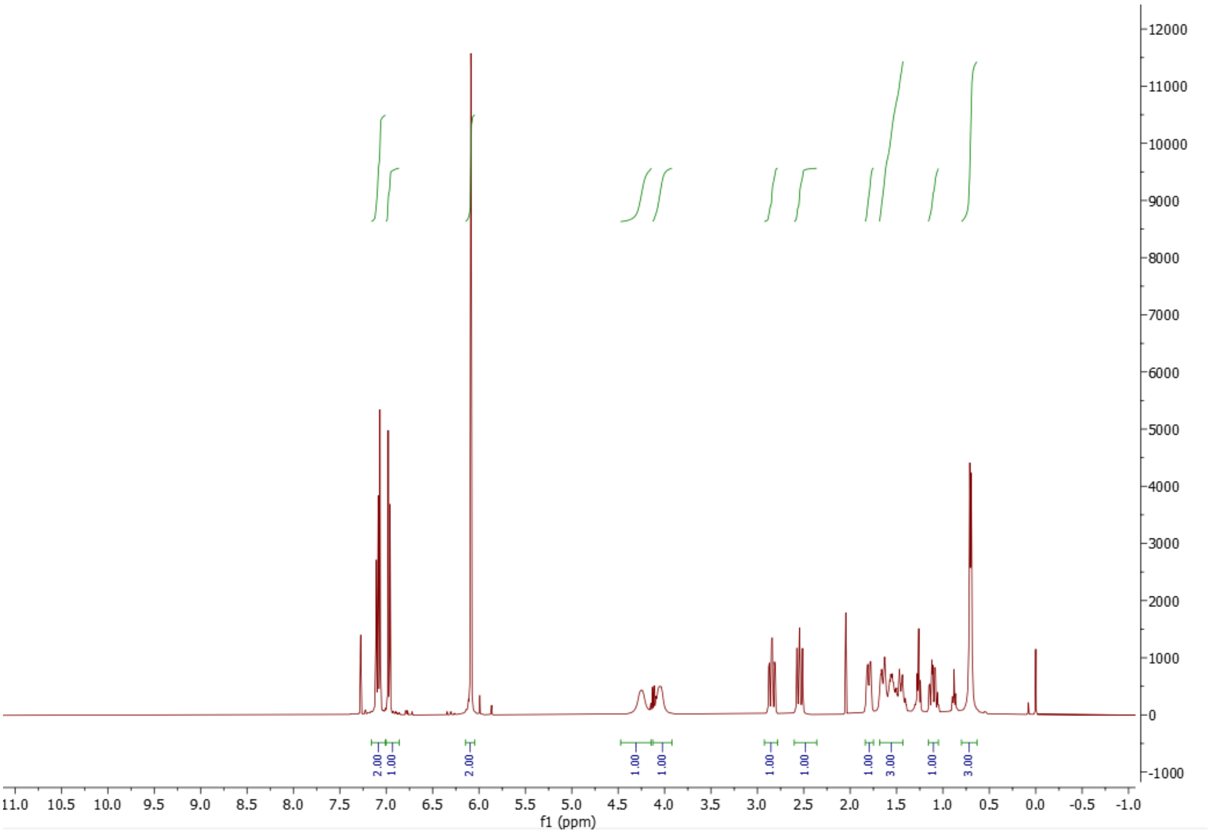

Figure S21: ^{1}H NMR of **20a**.

Chromatogram and Results

Injection Details

Injection Name:	**SST-46**	*Run Time (min):*	**12,00**
Vial Number:	**GA3**	*Injection Volume:*	**2,00**
Injection Type:	**Unknown**	*Channel:*	**UV_VIS_2**
Calibration Level:		*Wavelength:*	**254**
Instrument Method:	**Metoda_uplc**	*Bandwidth:*	**16**
Processing Method:	**Basic Quantitative**	*Dilution Factor:*	**1,0000**
Injection Date/Time:	**04.nov..21 10:57**	*Sample Weight:*	**1,0000**

Chromatogram

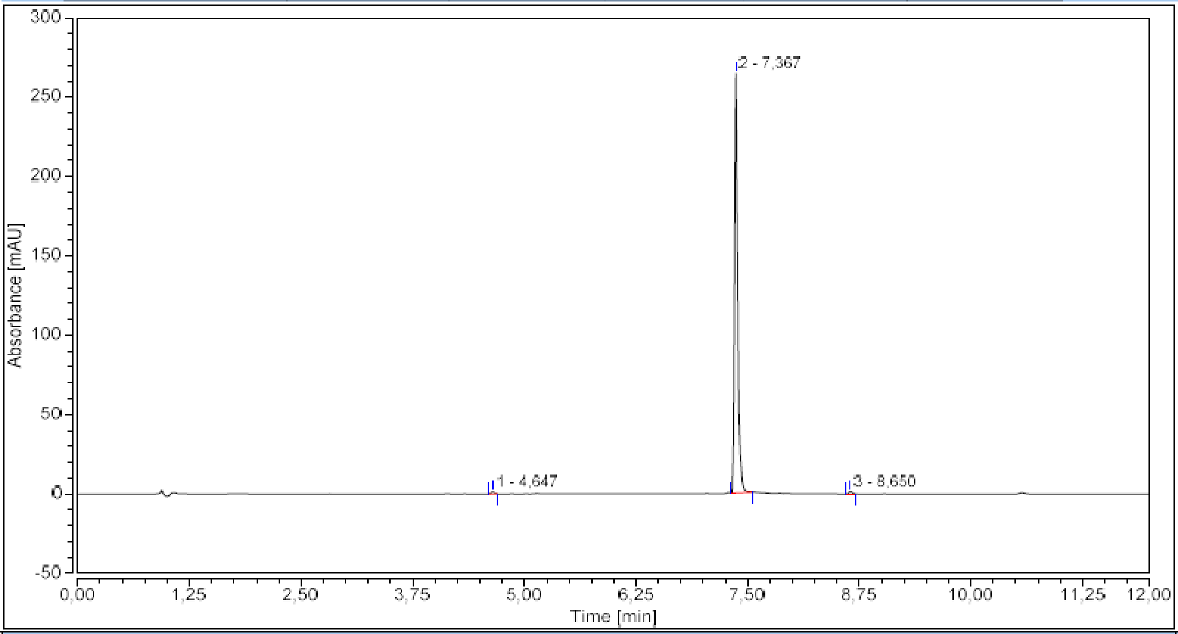

Integration Results

No.	Peak Name	Retention Time min	Area mAU*min	Height mAU	Relative Area %	Relative Height %	Amount n.a.
1		4,647	0,066	1,524	0,57	0,57	n.a.
2		7,367	11,351	264,300	98,78	98,85	n.a.
3		8,650	0,075	1,556	0,65	0,58	n.a.
**Total:**			**11,492**	**267,380**	**100,00**	**100,00**	

Figure S22: HPLC of **20a**.

20b

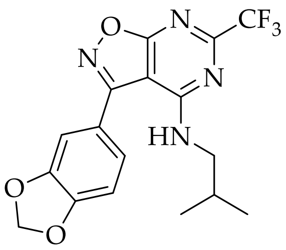

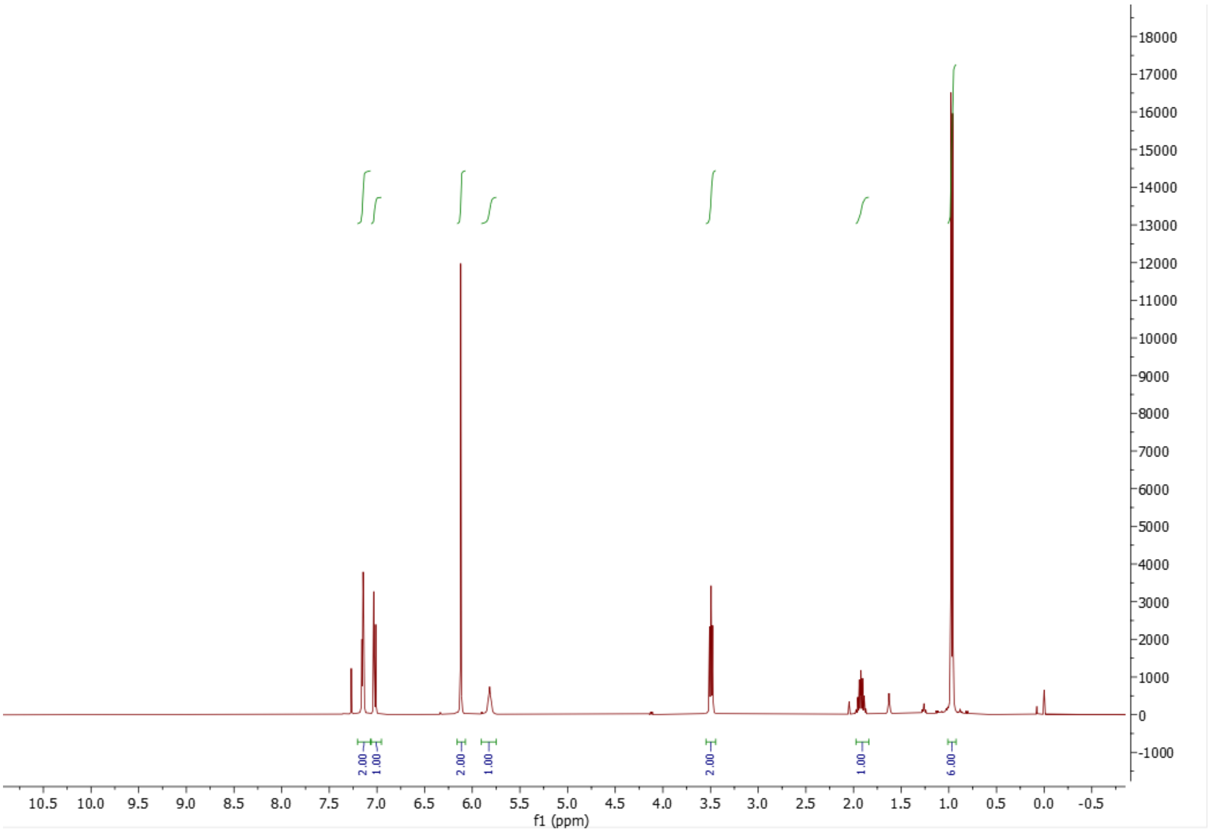

Figure S23: ^{1}H NMR of **20b**.

Chromatogram and Results

Injection Details

Injection Name:	**SST-47**	*Run Time (min):*	**12,00**
Vial Number:	**GA4**	*Injection Volume:*	**2,00**
Injection Type:	**Unknown**	*Channel:*	**UV_VIS_2**
Calibration Level:		*Wavelength:*	**254**
Instrument Method:	**Metoda_uplc**	*Bandwidth:*	**16**
Processing Method:	**Basic Quantitative**	*Dilution Factor:*	**1,0000**
Injection Date/Time:	**04.nov..21 11:15**	*Sample Weight:*	**1,0000**

Chromatogram

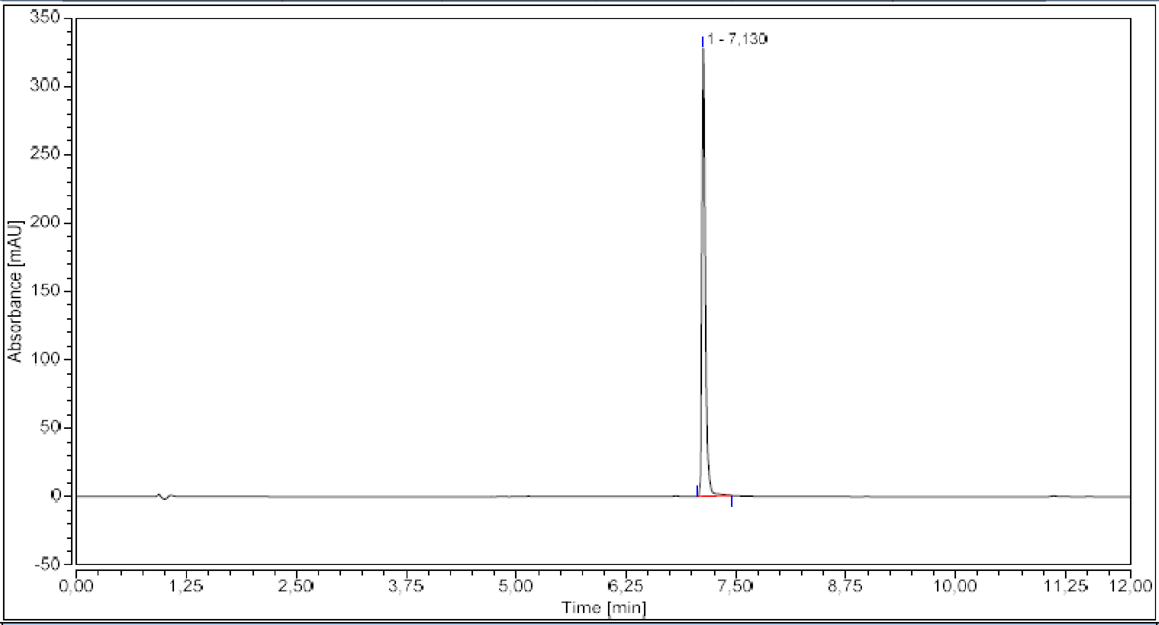

Integration Results

No.	Peak Name	Retention Time min	Area mAU*min	Height mAU	Relative Area %	Relative Height %	Amount n.a.
1		7,130	14,120	327,069	100,00	100,00	n.a.
**Total:**			**14,120**	**327,069**	**100,00**	**100,00**	

Figure S24: HPLC of **20b**.

21a

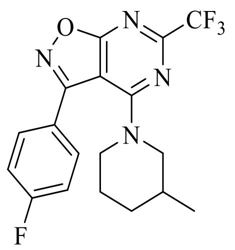

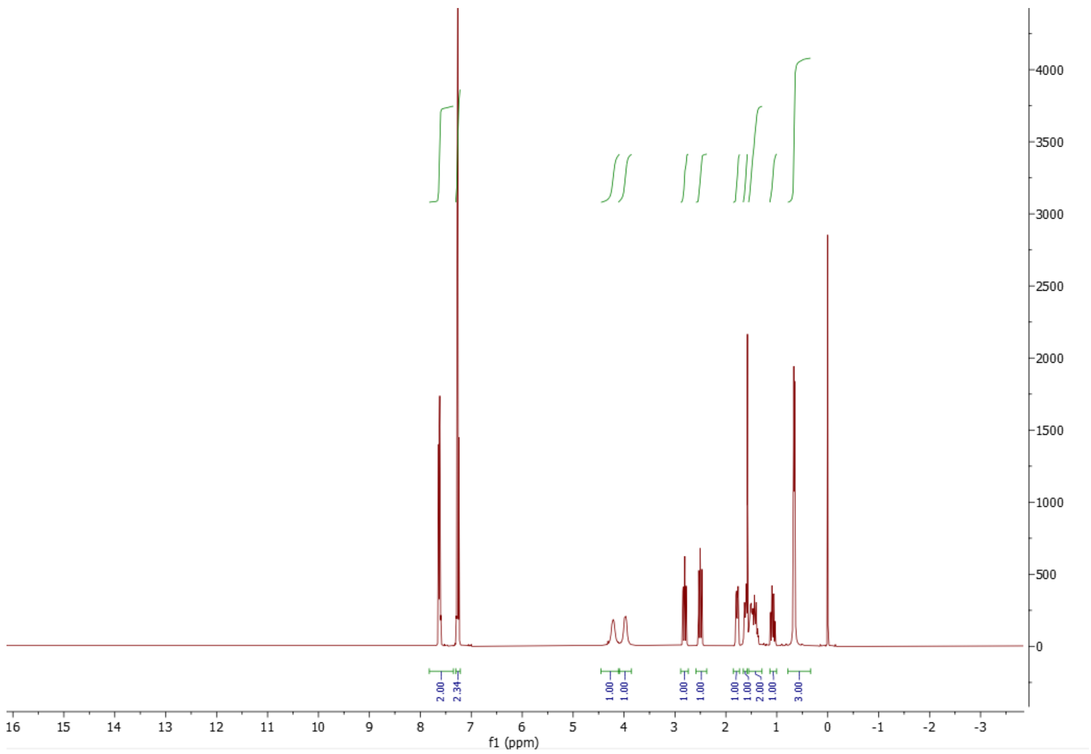

Figure S25: ^{1}H NMR of **21a**.

Chromatogram and Results

Injection Details

Injection Name:	**SN-33**	*Run Time (min):*	**12,00**
Vial Number:	**BA2**	*Injection Volume:*	**2,00**
Injection Type:	**Unknown**	*Channel:*	**UV_VIS_2**
Calibration Level:		*Wavelength:*	**254**
Instrument Method:	**Metoda_uplc**	*Bandwidth:*	**16**
Processing Method:	**Basic Quantitative**	*Dilution Factor:*	**1,0000**
Injection Date/Time:	**02.sep..21 10:55**	*Sample Weight:*	**1,0000**

Chromatogram

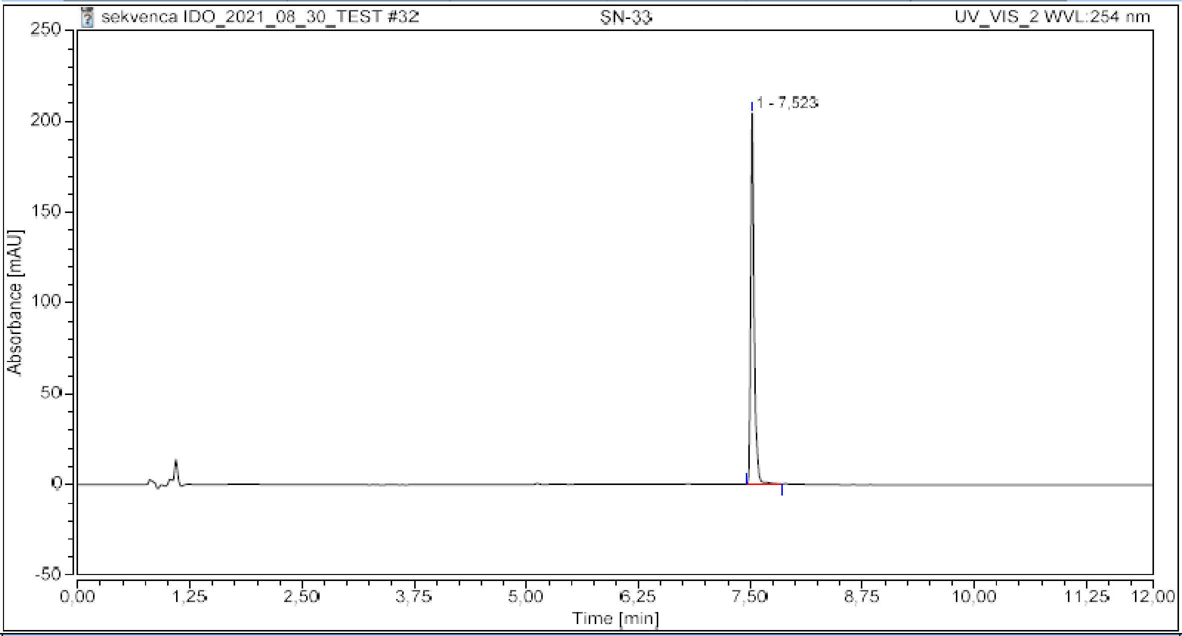

Integration Results

No.	Peak Name	Retention Time min	Area mAU*min	Height mAU	Relative Area %	Relative Height %	Amount n.a.
1		7,523	9,063	204,441	100,00	100,00	n.a.
**Total:**			**9,063**	**204,441**	**100,00**	**100,00**	

Figure S26: HPLC of **21a**.

21b

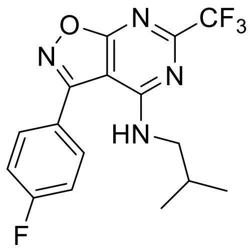

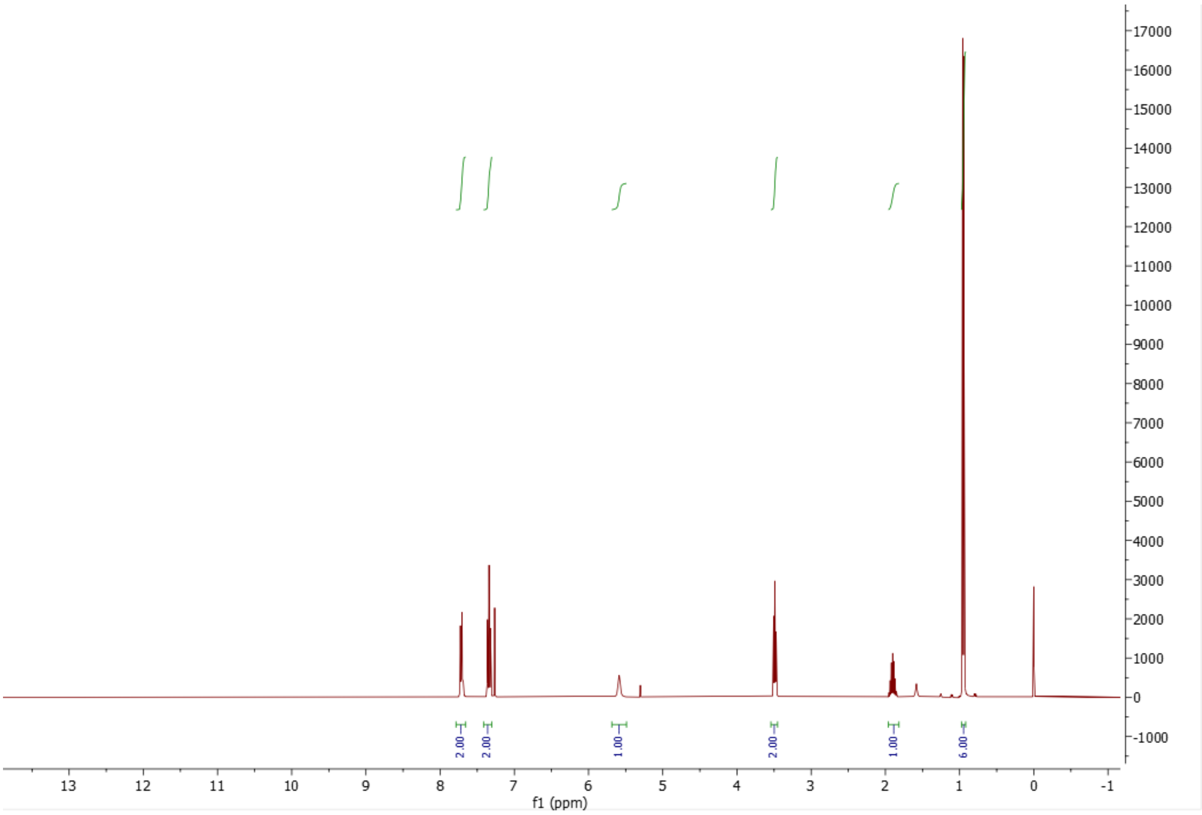

Figure S27: ^{1}H NMR of **21b**.

Chromatogram and Results

Injection Details

Injection Name:	**SN-35**	*Run Time (min):*	**8,00**
Vial Number:	**RA3**	*Injection Volume:*	**2,00**
Injection Type:	**Unspiked**	*Channel:*	**UV_VIS_2**
Calibration Level:		*Wavelength:*	**254**
Instrument Method:	**UPLC_Nika_splosna_metoda_TFA_MeCN**	*Bandwidth:*	**16**
Processing Method:	**Quantitative**	*Dilution Factor:*	**1,0000**
Injection Date/Time:	**13.nov..20 12:54**	*Sample Weight:*	**1,0000**

Chromatogram

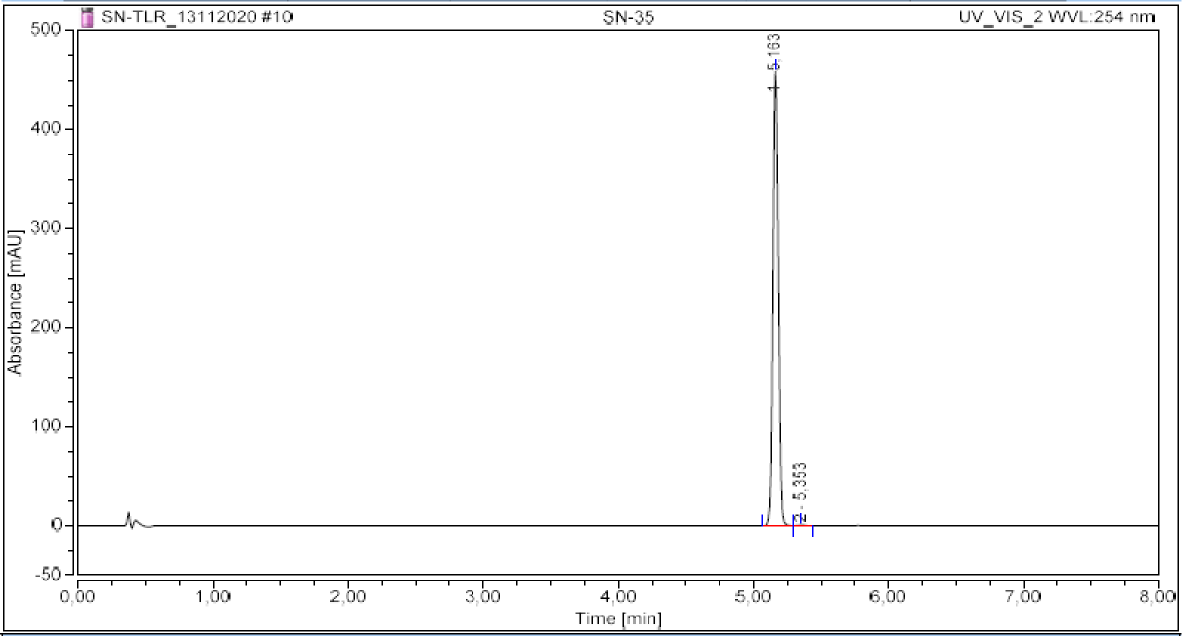

Integration Results

No.	Peak Name	Retention Time min	Area mAU*min	Height mAU	Relative Area %	Relative Height %	Amount n.a.
1		5,163	20,834	459,106	99,81	99,84	n.a.
2		5,353	0,039	0,735	0,19	0,16	n.a.
**Total:**			**20,872**	**459,841**	**100,00**	**100,00**	

Figure S28: HPLC of **21b**.

21c

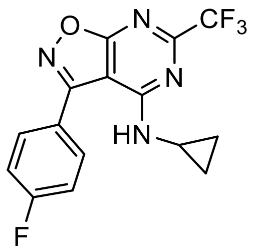

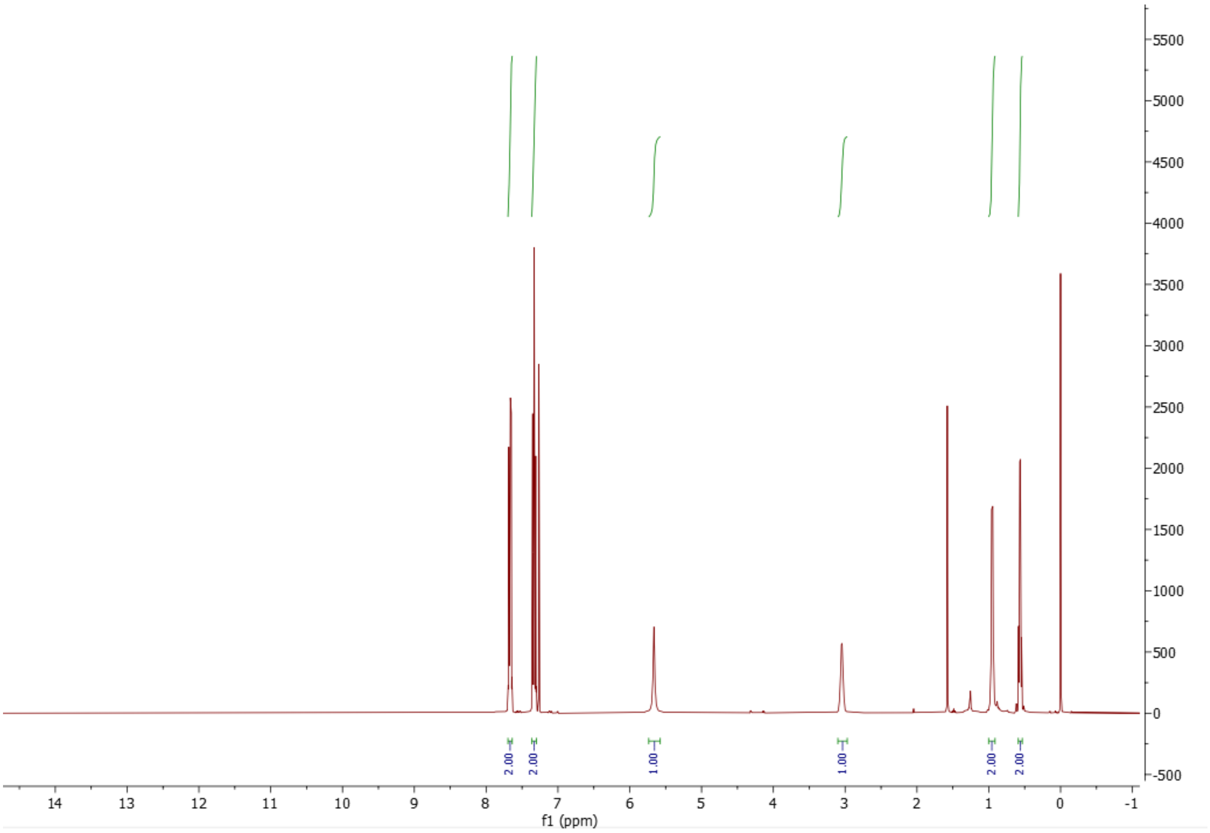

Figure S29: ^{1}H NMR of **21c**.

Chromatogram and Results

Injection Details

Injection Name:	**SN-34**	*Run Time (min):*	**8,00**
Vial Number:	**RA2**	*Injection Volume:*	**2,00**
Injection Type:	**Unspiked**	*Channel:*	**UV_VIS_2**
Calibration Level:		*Wavelength:*	**254**
Instrument Method:	**UPLC_Nika_splosna_metoda_TFA_MeCN**	*Bandwidth:*	**16**
Processing Method:	**Quantitative**	*Dilution Factor:*	**1,0000**
Injection Date/Time:	**13.nov..20 12:41**	*Sample Weight:*	**1,0000**

Chromatogram

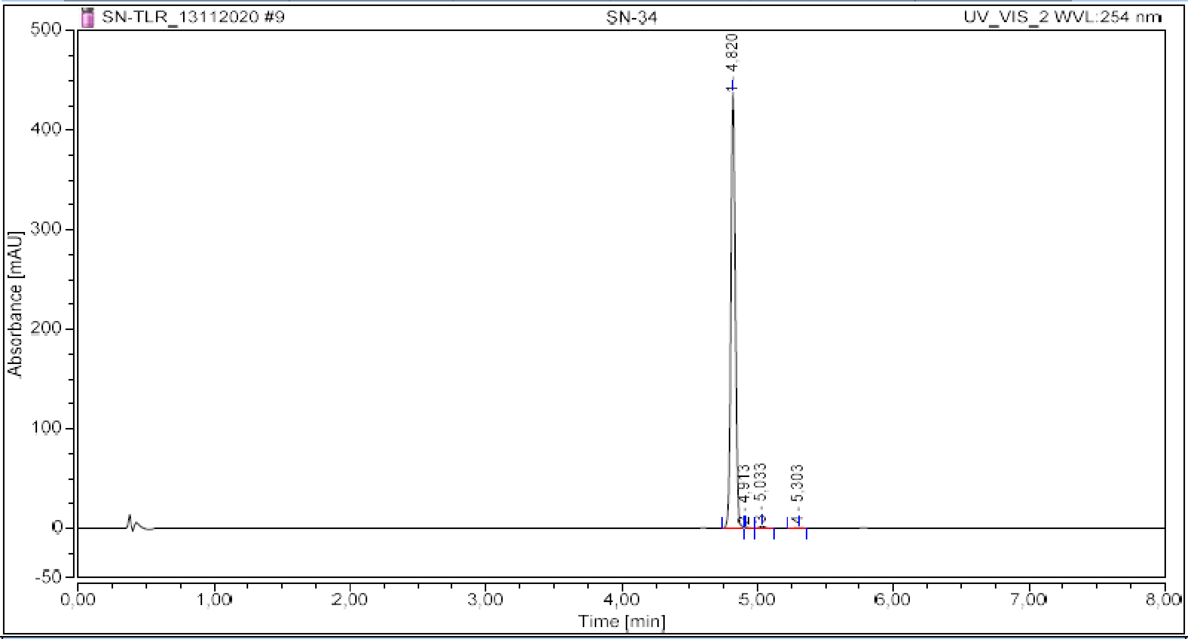

Integration Results

No.	Peak Name	Retention Time min	Area mAU*min	Height mAU	Relative Area %	Relative Height %	Amount n.a.
1		4,820	17,801	438,119	99,27	99,27	n.a.
2		4,913	0,027	0,963	0,15	0,22	n.a.
3		5,033	0,082	1,812	0,46	0,41	n.a.
4		5,303	0,022	0,427	0,12	0,10	n.a.
**Total:**			**17,932**	**441,321**	**100,00**	**100,00**	

Figure S30: HPLC of **21c**.

21d

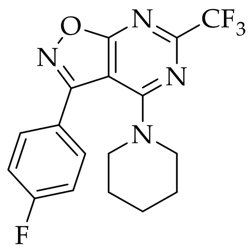

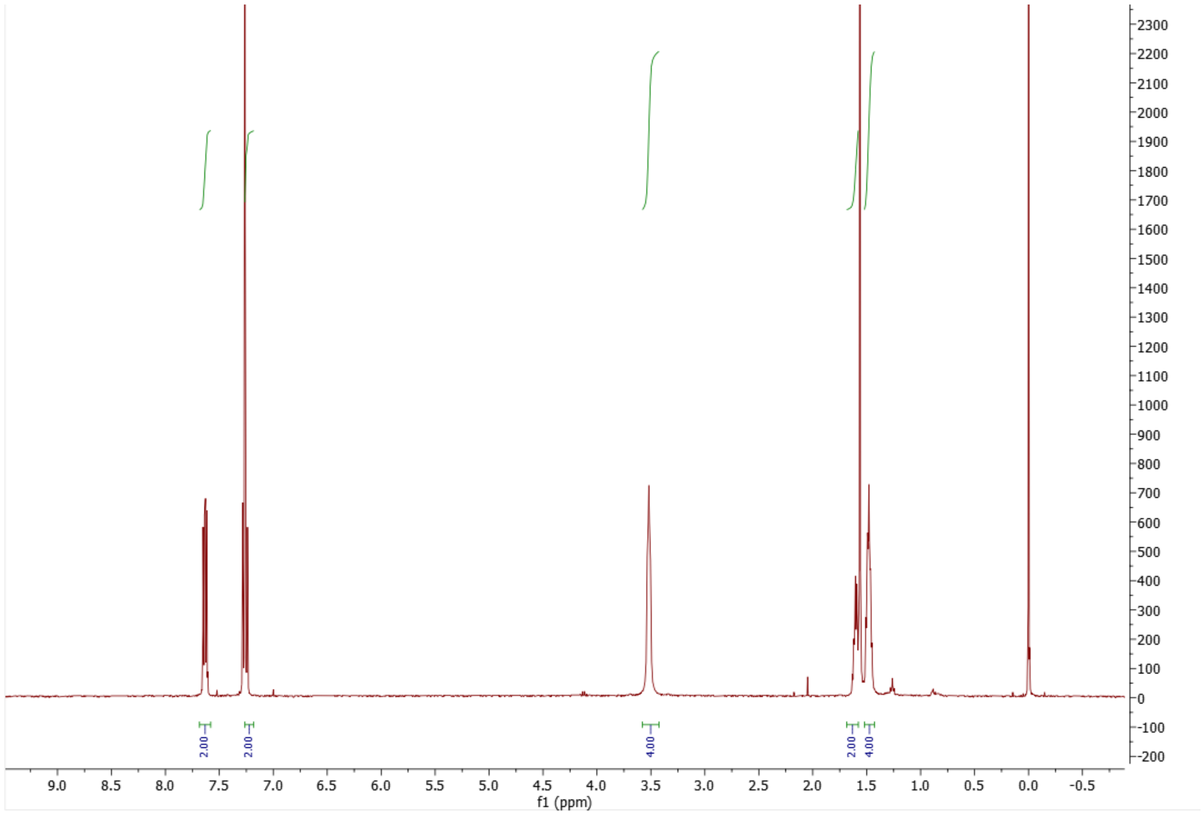

Figure S31: ^{1}H NMR of **21d**.

Chromatogram and Results

Injection Details

Injection Name:	**SBS-14**	*Run Time (min):*	**12,00**
Vial Number:	**GC1**	*Injection Volume:*	**2,00**
Injection Type:	**Unknown**	*Channel:*	**UV_VIS_2**
Calibration Level:		*Wavelength:*	**254**
Instrument Method:	**Metoda_uplc**	*Bandwidth:*	**16**
Processing Method:	**Basic Quantitative**	*Dilution Factor:*	**1,0000**
Injection Date/Time:	**04.nov..21 15:07**	*Sample Weight:*	**1,0000**

Chromatogram

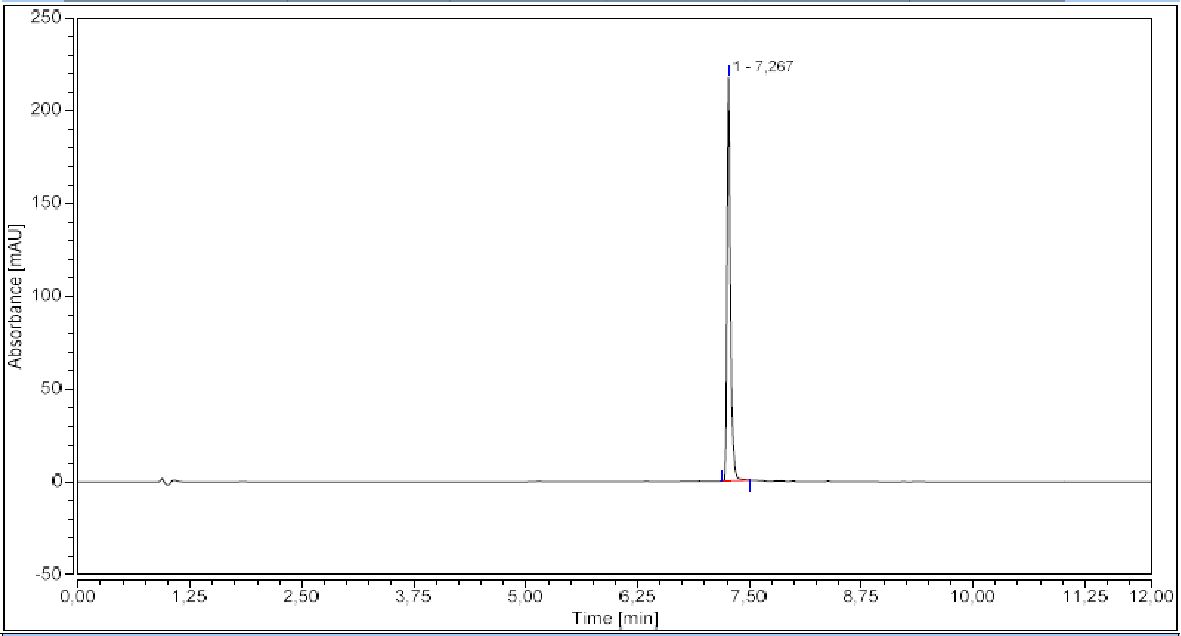

Integration Results

No.	Peak Name	Retention Time min	Area mAU*min	Height mAU	Relative Area %	Relative Height %	Amount n.a.
1		7,267	9,391	217,462	100,00	100,00	n.a.
**Total:**			**9,391**	**217,462**	**100,00**	**100,00**	

Figure S32: HPLC of **21d**.

21e

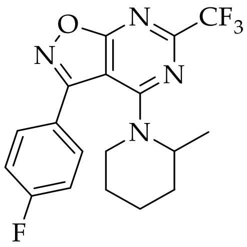

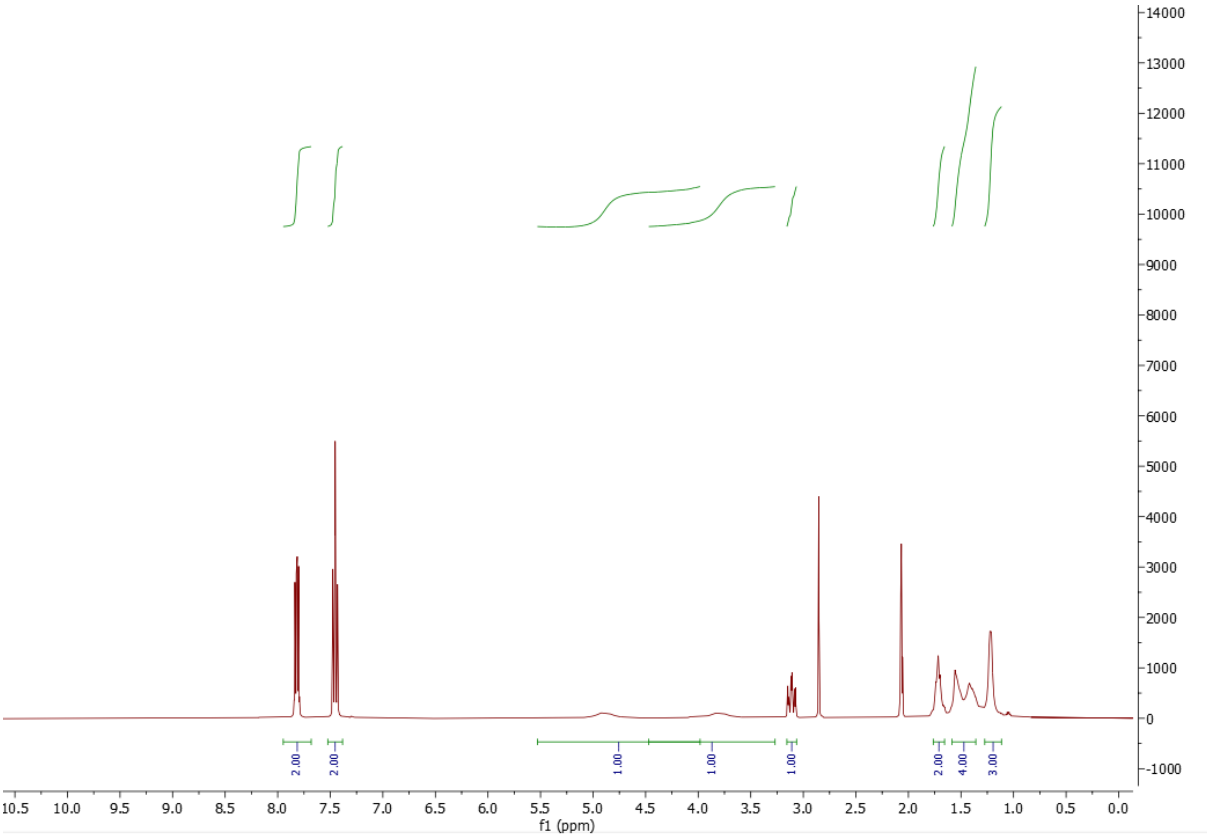

Figure S33: ^{1}H NMR of **21e**.

Chromatogram and Results

Injection Details

Injection Name:	**SBS-6**	*Run Time (min):*	**12,00**
Vial Number:	**GB1**	*Injection Volume:*	**2,00**
Injection Type:	**Unknown**	*Channel:*	**UV_VIS_2**
Calibration Level:		*Wavelength:*	**254**
Instrument Method:	**Metoda_uplc**	*Bandwidth:*	**16**
Processing Method:	**Basic Quantitative**	*Dilution Factor:*	**1,0000**
Injection Date/Time:	**04.nov..21 12:44**	*Sample Weight:*	**1,0000**

Chromatogram

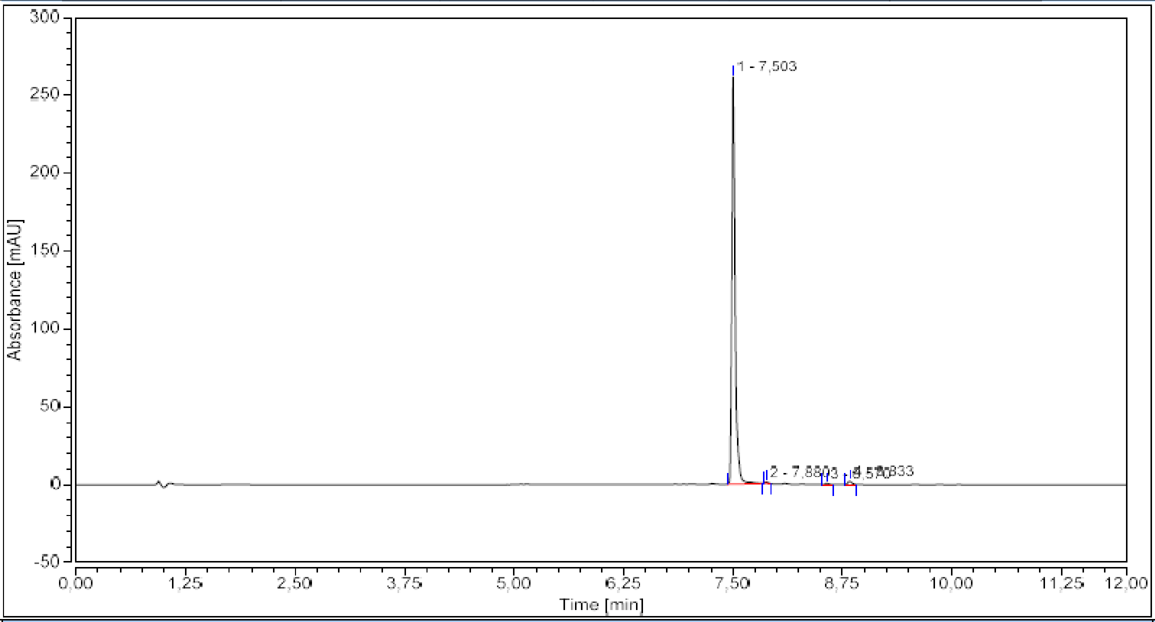

Integration Results

No.	Peak Name	Retention Time min	Area mAU*min	Height mAU	Relative Area %	Relative Height %	Amount n.a.
1		7,503	11,732	261,622	98,28	98,46	n.a.
2		7,880	0,044	1,081	0,37	0,41	n.a.
3		8,570	0,041	0,787	0,34	0,30	n.a.
4		8,833	0,121	2,228	1,01	0,84	n.a.
**Total:**			**11,937**	**265,719**	**100,00**	**100,00**	

Figure S34: HPLC of **21e**.

21f

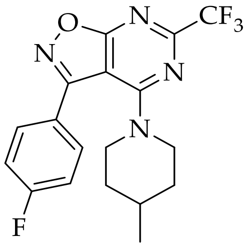

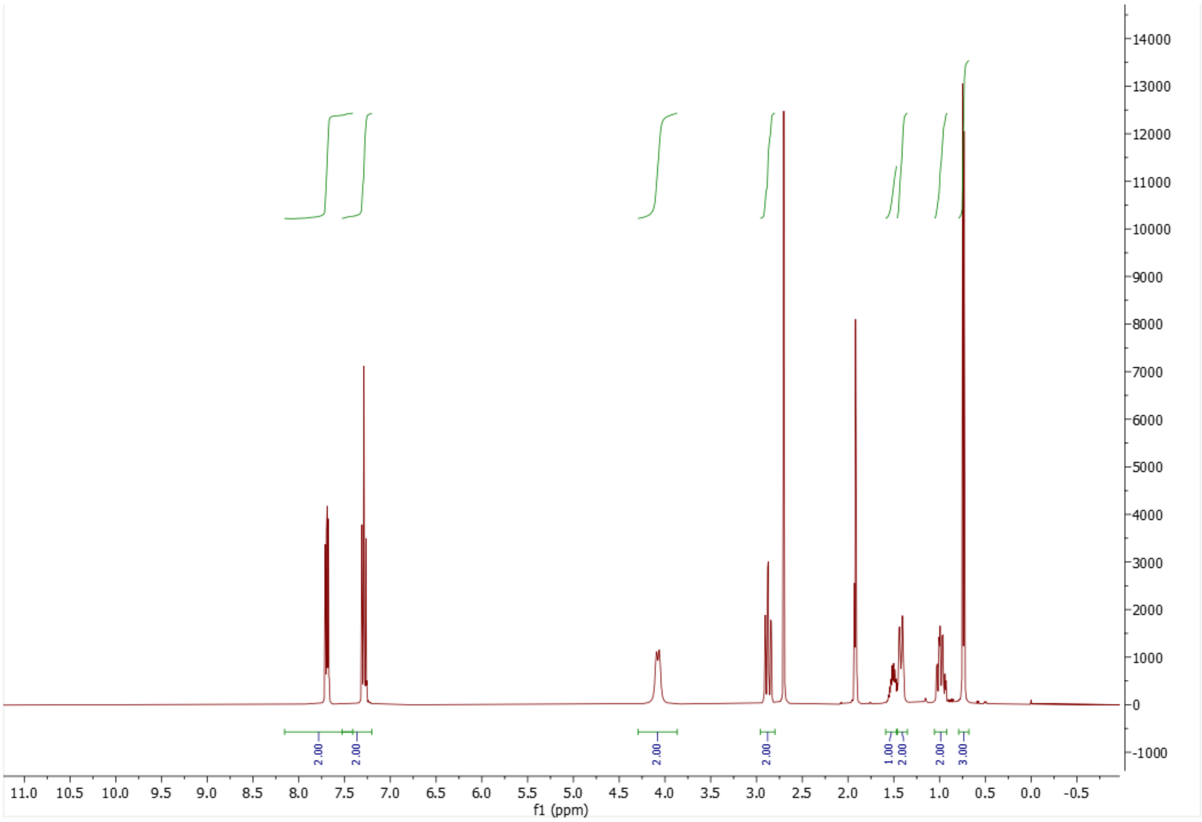

Figure S35: ^{1}H NMR of **21f**.

Chromatogram and Results

Injection Details

Injection Name:	**SBS-7**	*Run Time (min):*	**12,00**
Vial Number:	**GB2**	*Injection Volume:*	**2,00**
Injection Type:	**Unknown**	*Channel:*	**UV_VIS_2**
Calibration Level:		*Wavelength:*	**254**
Instrument Method:	**Metoda_uplc**	*Bandwidth:*	**16**
Processing Method:	**Basic Quantitative**	*Dilution Factor:*	**1,0000**
Injection Date/Time:	**04.nov..21 13:02**	*Sample Weight:*	**1,0000**

Chromatogram

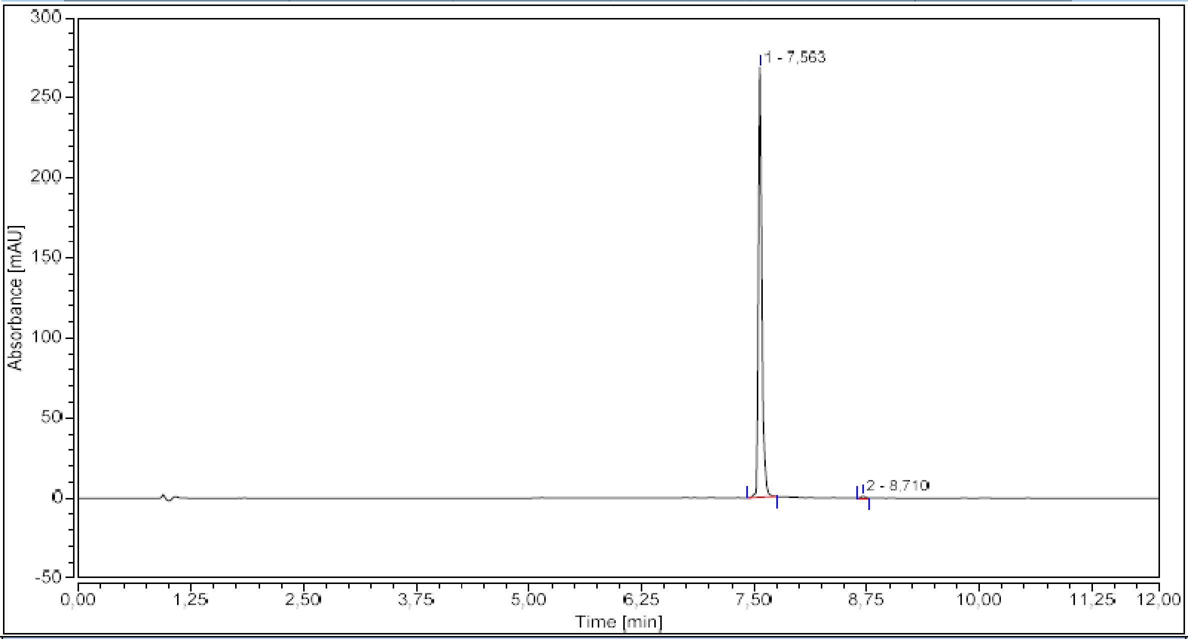

Integration Results

No.	Peak Name	Retention Time<br>min	Area<br>mAU*min	Height<br>mAU	Relative Area<br>%	Relative Height<br>%	Amount<br>n.a.
1		7,563	11,866	268,467	99,48	99,54	n.a.
2		8,710	0,062	1,237	0,52	0,46	n.a.
**Total:**			**11,928**	**269,704**	**100,00**	**100,00**	

Figure S36: HPLC of **21f**.

21g

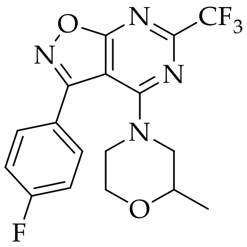

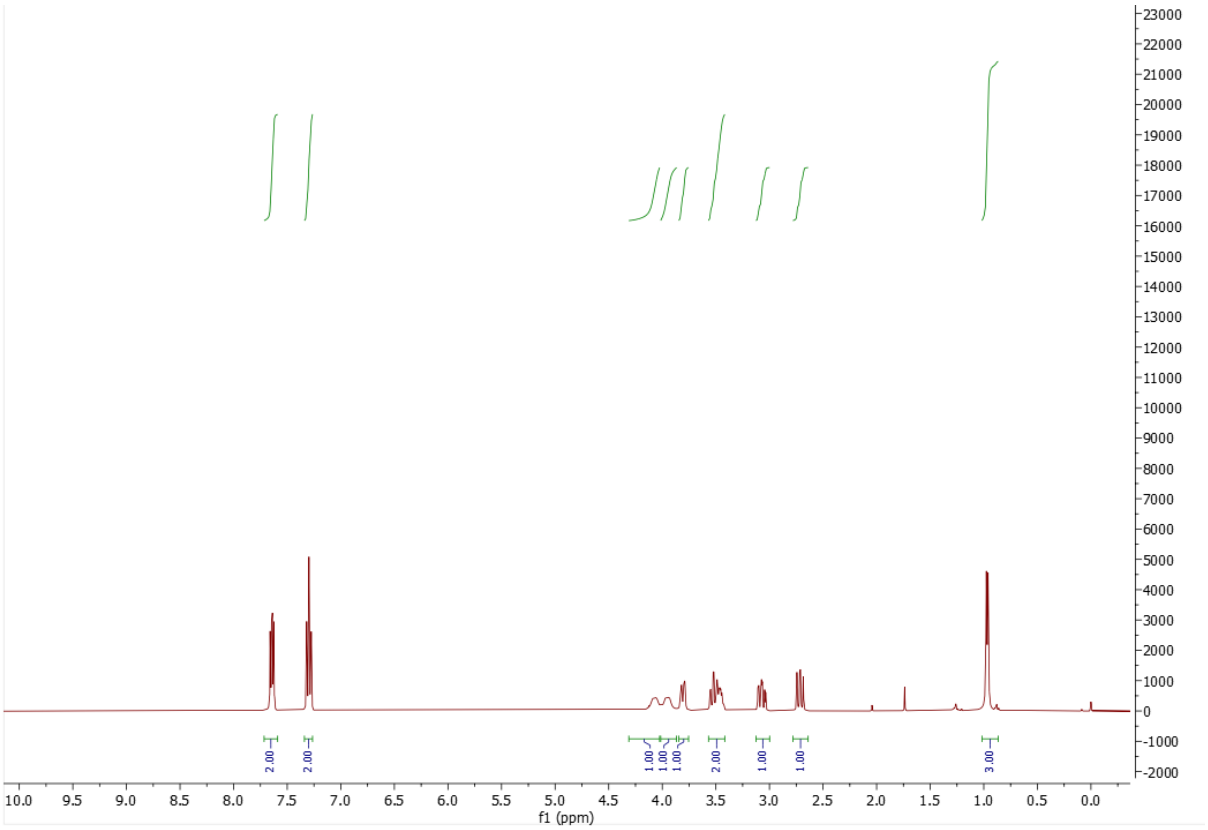

Figure S37: ^{1}H NMR of **21g**.

Chromatogram and Results

Injection Details

Injection Name:	**SKV-8**	*Run Time (min):*	**8,00**
Vial Number:	**RA8**	*Injection Volume:*	**2,00**
Injection Type:	**Unknown**	*Channel:*	**UV_VIS_2**
Calibration Level:		*Wavelength:*	**254**
Instrument Method:	**UPLC_Nika_splosna_metoda_TFA_MeCN**	*Bandwidth:*	**16**
Processing Method:	**Quantitative**	*Dilution Factor:*	**1,0000**
Injection Date/Time:	**20.mar..23 14:09**	*Sample Weight:*	**1,0000**

Chromatogram

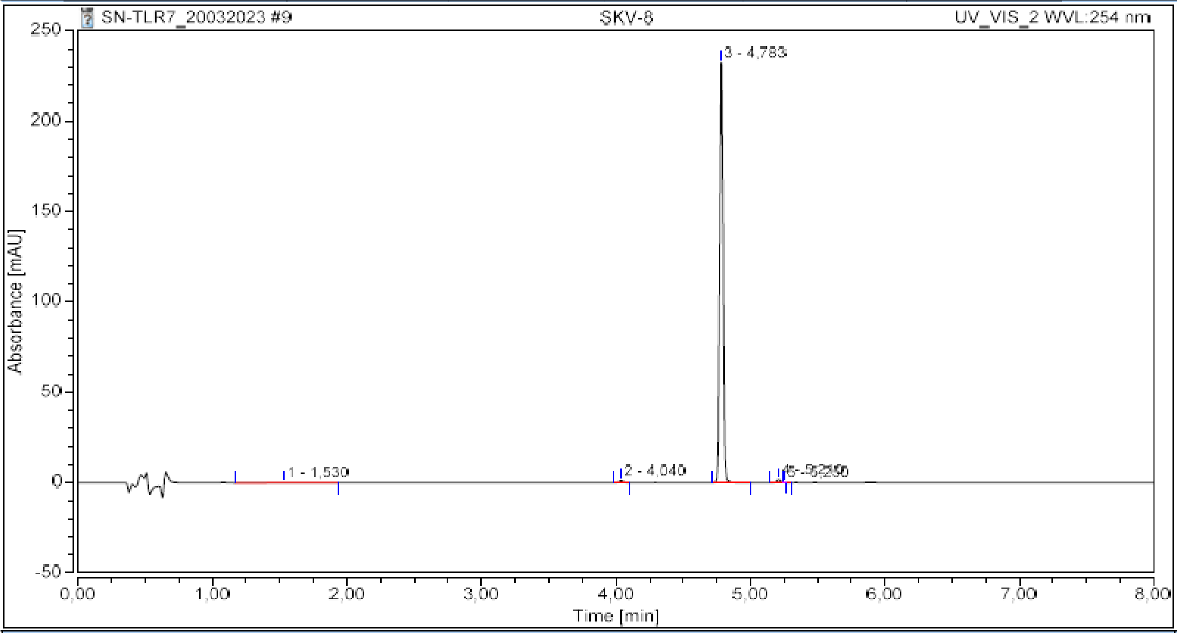

Integration Results

No.	Peak Name	Retention Time min	Area mAU*min	Height mAU	Relative Area %	Relative Height %	Amount n.a.
1		1,530	0,044	0,083	0,63	0,04	n.a.
2		4,040	0,040	1,258	0,58	0,53	n.a.
3		4,783	6,827	232,632	97,92	98,67	n.a.
4		5,210	0,060	1,750	0,86	0,74	n.a.
5		5,250	0,001	0,049	0,01	0,02	n.a.
**Total:**			**6,972**	**235,772**	**100,00**	**100,00**	

Figure S38: HPLC of **21g**.

21h

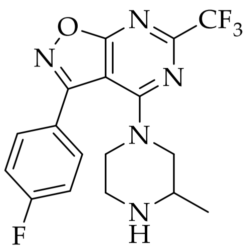

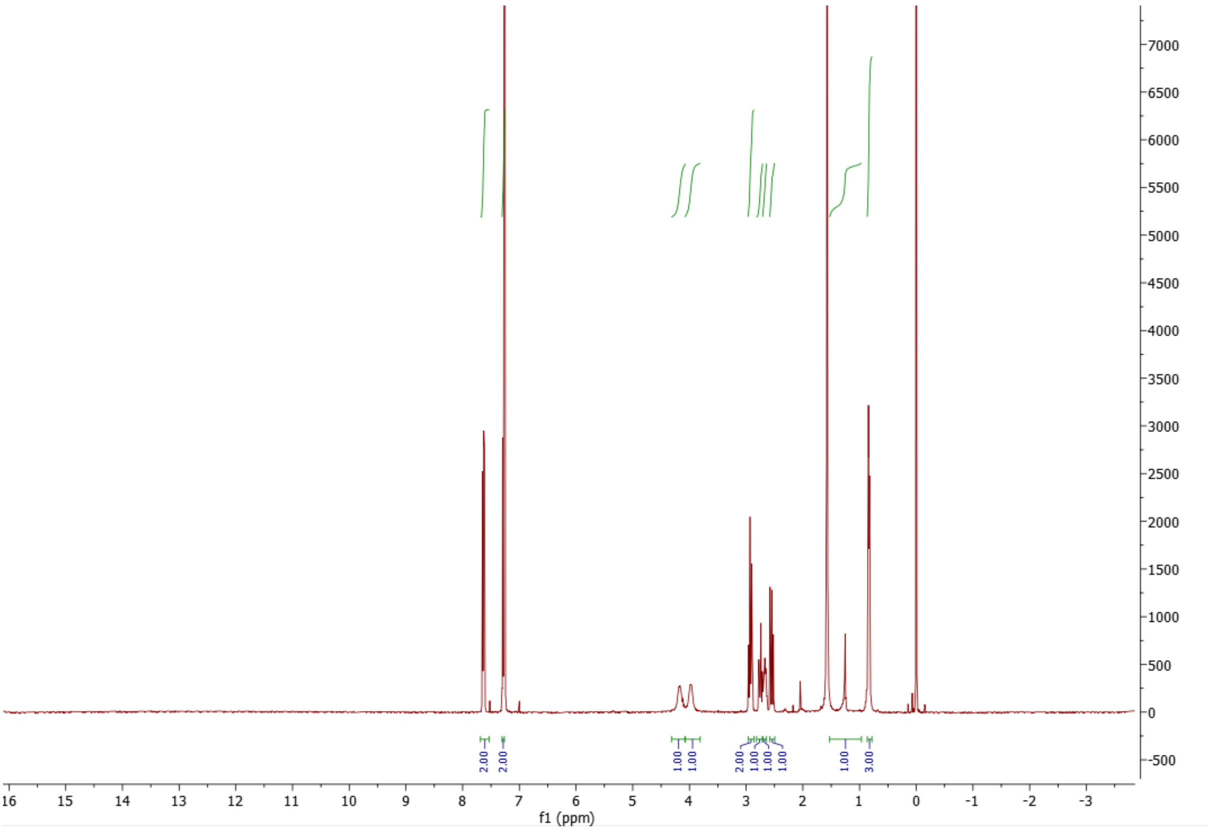

Figure S39: ^{1}H NMR of **21h**.

Chromatogram and Results

Injection Details

Injection Name:	**SBS-11**	*Run Time (min):*	**12,00**
Vial Number:	**GB6**	*Injection Volume:*	**2,00**
Injection Type:	**Unknown**	*Channel:*	**UV_VIS_2**
Calibration Level:		*Wavelength:*	**254**
Instrument Method:	**Metoda_uplc**	*Bandwidth:*	**16**
Processing Method:	**Basic Quantitative**	*Dilution Factor:*	**1,0000**
Injection Date/Time:	**04.nov..21 14:13**	*Sample Weight:*	**1,0000**

Chromatogram

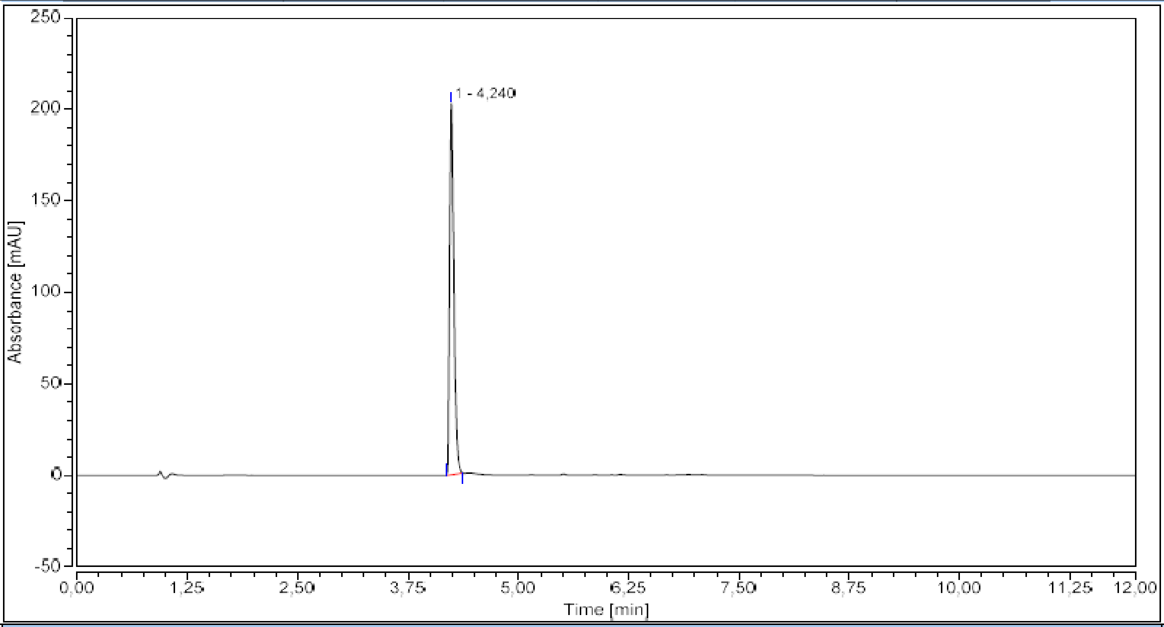

Integration Results

No.	Peak Name	Retention Time min	Area mAU*min	Height mAU	Relative Area %	Relative Height %	Amount n.a.
1		4,240	11,082	202,648	100,00	100,00	n.a.
**Total:**			**11,082**	**202,648**	**100,00**	**100,00**	

Figure S40: HPLC of **21h**.

21i

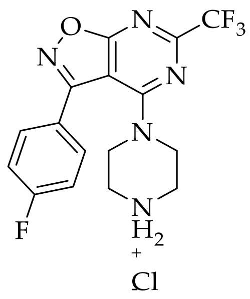

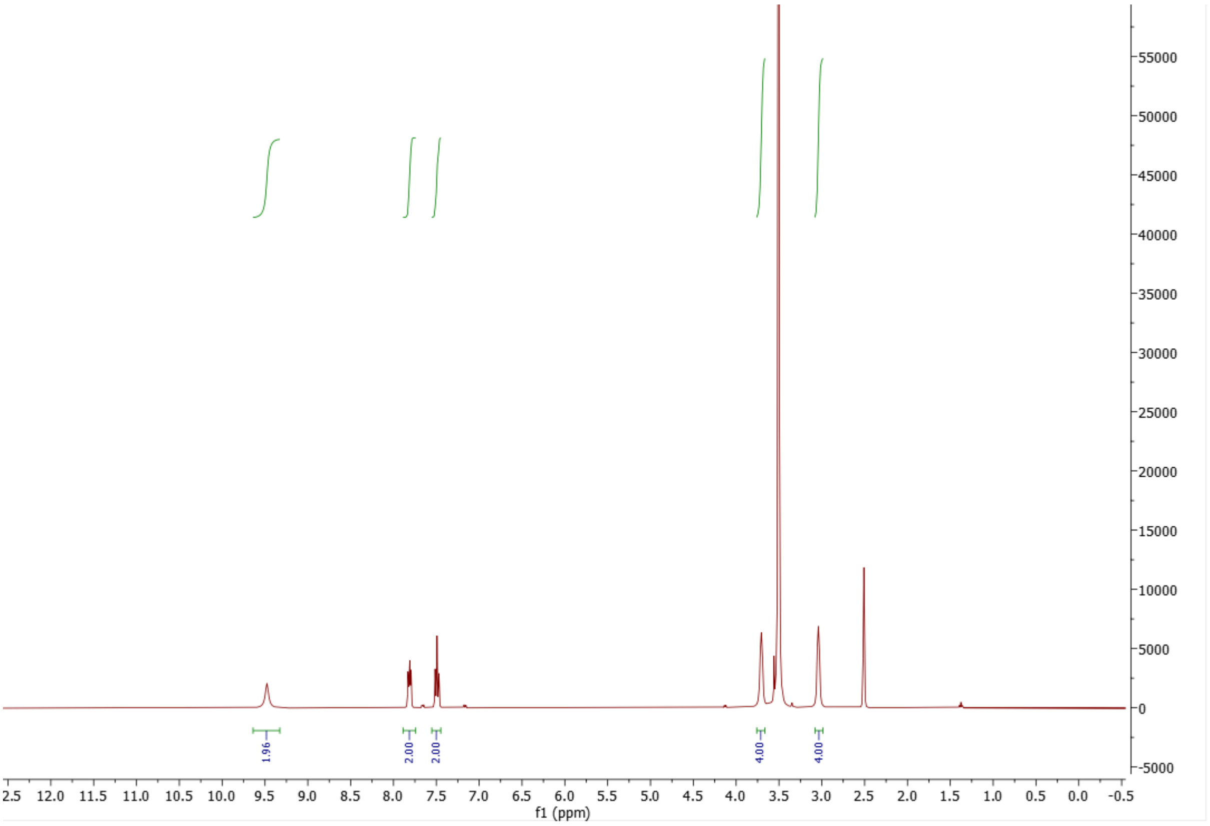

Figure S41: ^{1}H NMR of **21i**.

Chromatogram and Results

Injection Details

Injection Name:	**SBS-13A**	*Run Time (min):*	**12,00**
Vial Number:	**GB8**	*Injection Volume:*	**2,00**
Injection Type:	**Unknown**	*Channel:*	**UV_VIS_2**
Calibration Level:		*Wavelength:*	**254**
Instrument Method:	**Metoda_uplc**	*Bandwidth:*	**16**
Processing Method:	**Basic Quantitative**	*Dilution Factor:*	**1,0000**
Injection Date/Time:	**04.nov..21 14:49**	*Sample Weight:*	**1,0000**

Chromatogram

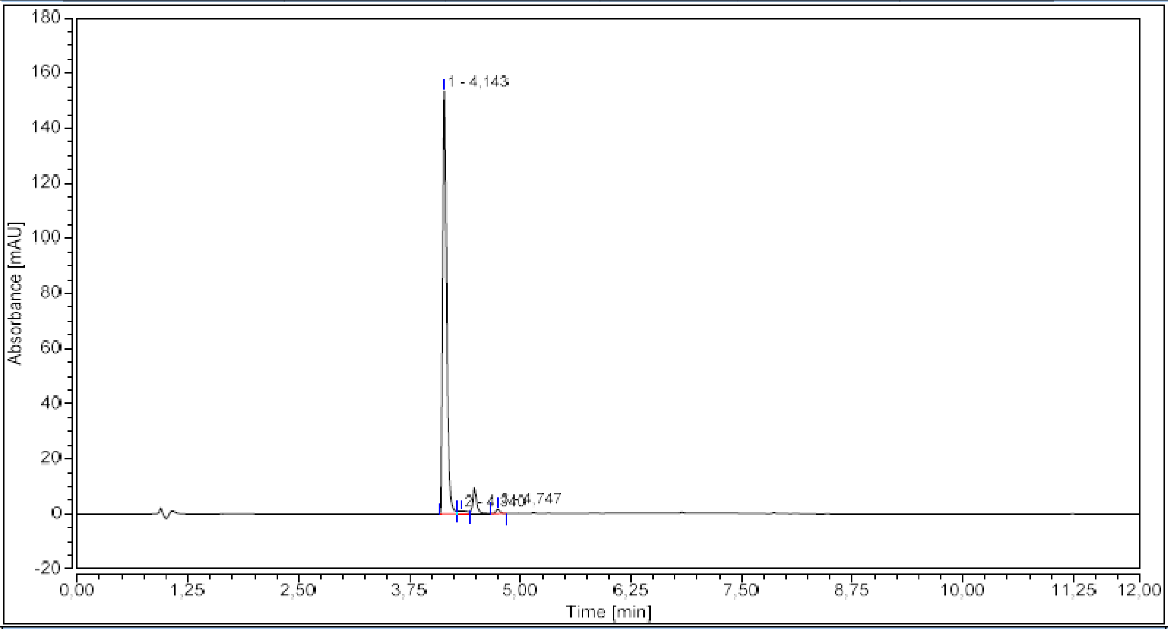

Integration Results

No.	Peak Name	Retention Time min	Area mAU*min	Height mAU	Relative Area %	Relative Height %	Amount n.a.
1		4,143	7,915	153,365	97,44	98,29	n.a.
2		4,340	0,126	0,977	1,56	0,63	n.a.
3		4,747	0,082	1,696	1,01	1,09	n.a.
**Total:**			**8,123**	**156,037**	**100,00**	**100,00**	

Figure S42: HPLC of **21i**.

21j

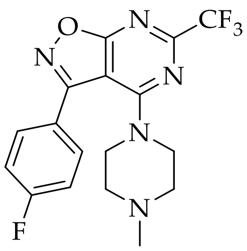

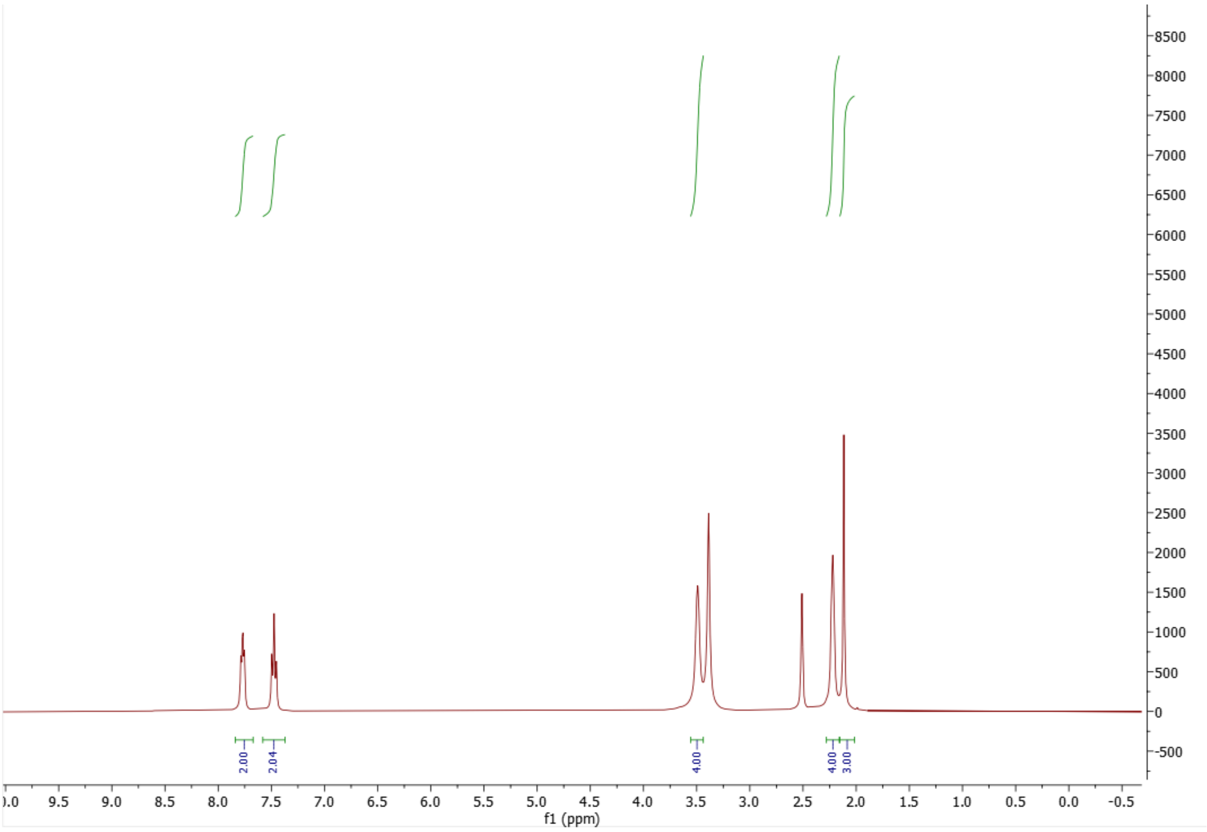

Figure S43: ^{1}H NMR of **21j**.

Chromatogram and Results

Injection Details

Injection Name:	**SBS-10**	*Run Time (min):*	**12,00**
Vial Number:	**GB5**	*Injection Volume:*	**2,00**
Injection Type:	**Unknown**	*Channel:*	**UV_VIS_2**
Calibration Level:		*Wavelength:*	**254**
Instrument Method:	**Metoda_uplc**	*Bandwidth:*	**16**
Processing Method:	**Basic Quantitative**	*Dilution Factor:*	**1,0000**
Injection Date/Time:	**04.nov..21 13:56**	*Sample Weight:*	**1,0000**

Chromatogram

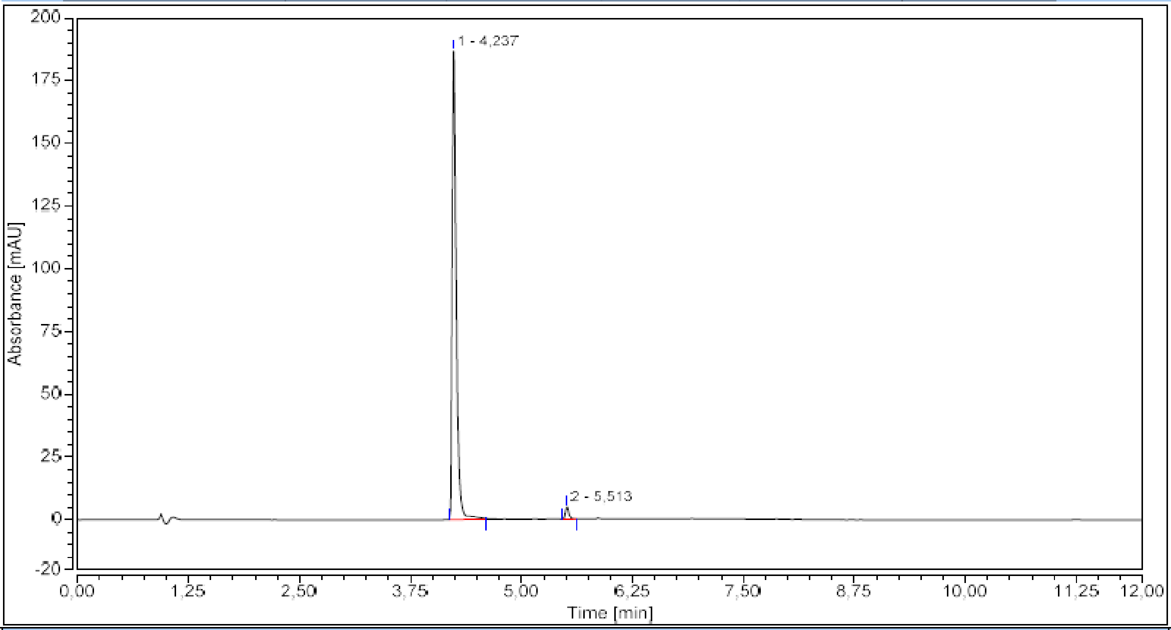

Integration Results

No.	Peak Name	Retention Time min	Area mAU*min	Height mAU	Relative Area %	Relative Height %	Amount n.a.
1		4,237	10,423	186,678	97,93	97,40	n.a.
2		5,513	0,220	4,988	2,07	2,60	n.a.
**Total:**			**10,643**	**191,667**	**100,00**	**100,00**	

Figure S44: HPLC of **21j**.

21k

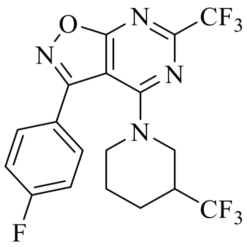

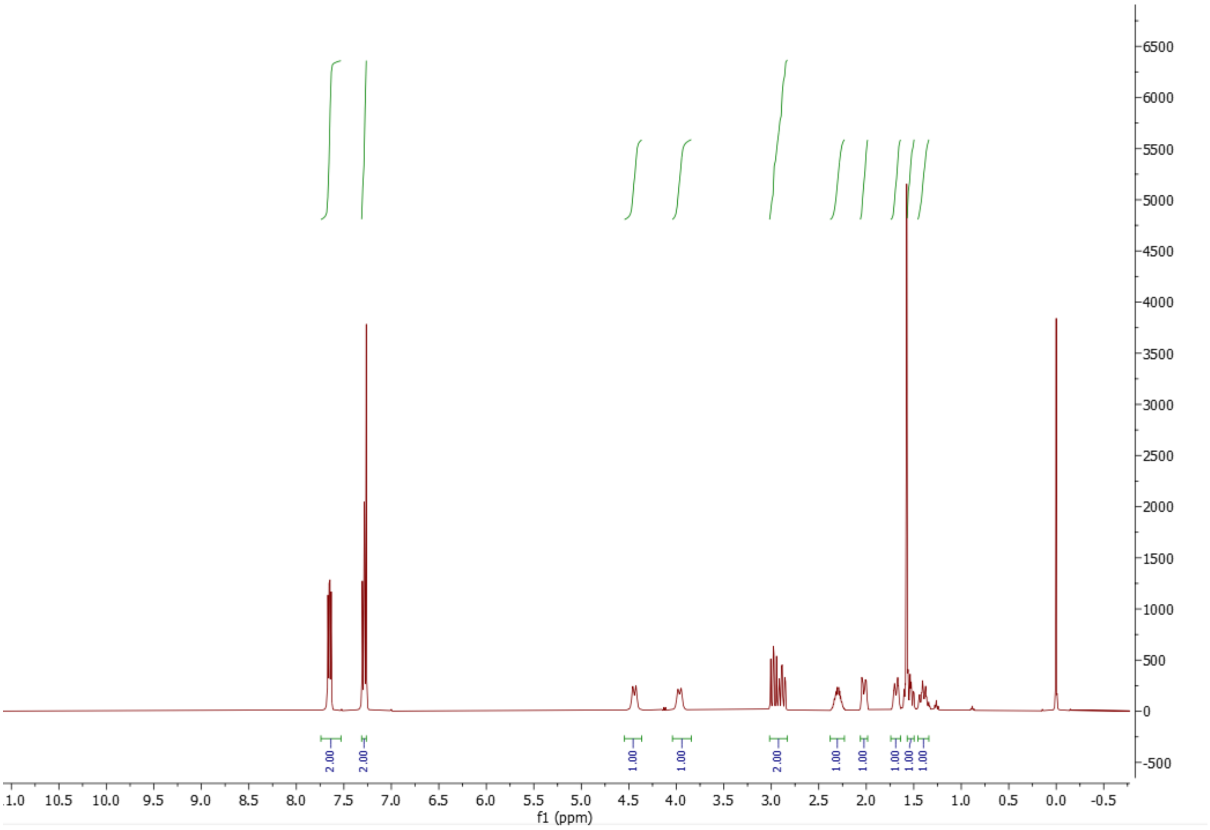

Figure S45: ^{1}H NMR of **21k**.

Chromatogram and Results

Injection Details

Injection Name:	**SN-327**	*Run Time (min):*	**8,00**
Vial Number:	**RA3**	*Injection Volume:*	**2,00**
Injection Type:	**Unknown**	*Channel:*	**UV_VIS_2**
Calibration Level:		*Wavelength:*	**254**
Instrument Method:	**UPLC_Nika_splosna_metoda_TFA_MeCN**	*Bandwidth:*	**16**
Processing Method:	**Quantitative**	*Dilution Factor:*	**1,0000**
Injection Date/Time:	**20.mar..23 13:02**	*Sample Weight:*	**1,0000**

Chromatogram

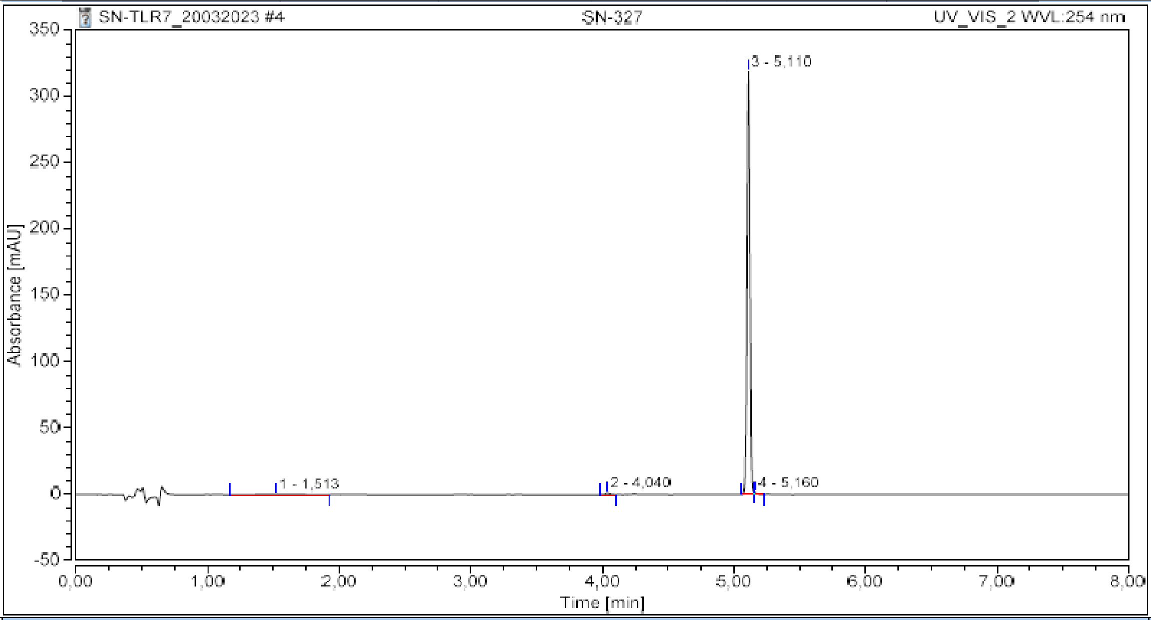

Integration Results

No.	Peak Name	Retention Time min	Area mAU*min	Height mAU	Relative Area %	Relative Height %	Amount n.a.
1		1,513	0,047	0,093	0,52	0,03	n.a.
2		4,040	0,040	1,242	0,44	0,39	n.a.
3		5,110	9,022	319,178	98,72	99,13	n.a.
4		5,160	0,029	1,471	0,32	0,46	n.a.
**Total:**			**9,139**	**321,984**	**100,00**	**100,00**	

Figure S46: HPLC of **21k**.

21l

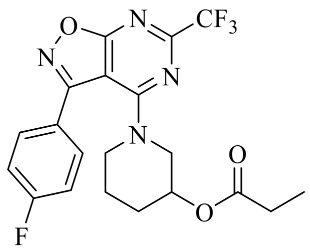

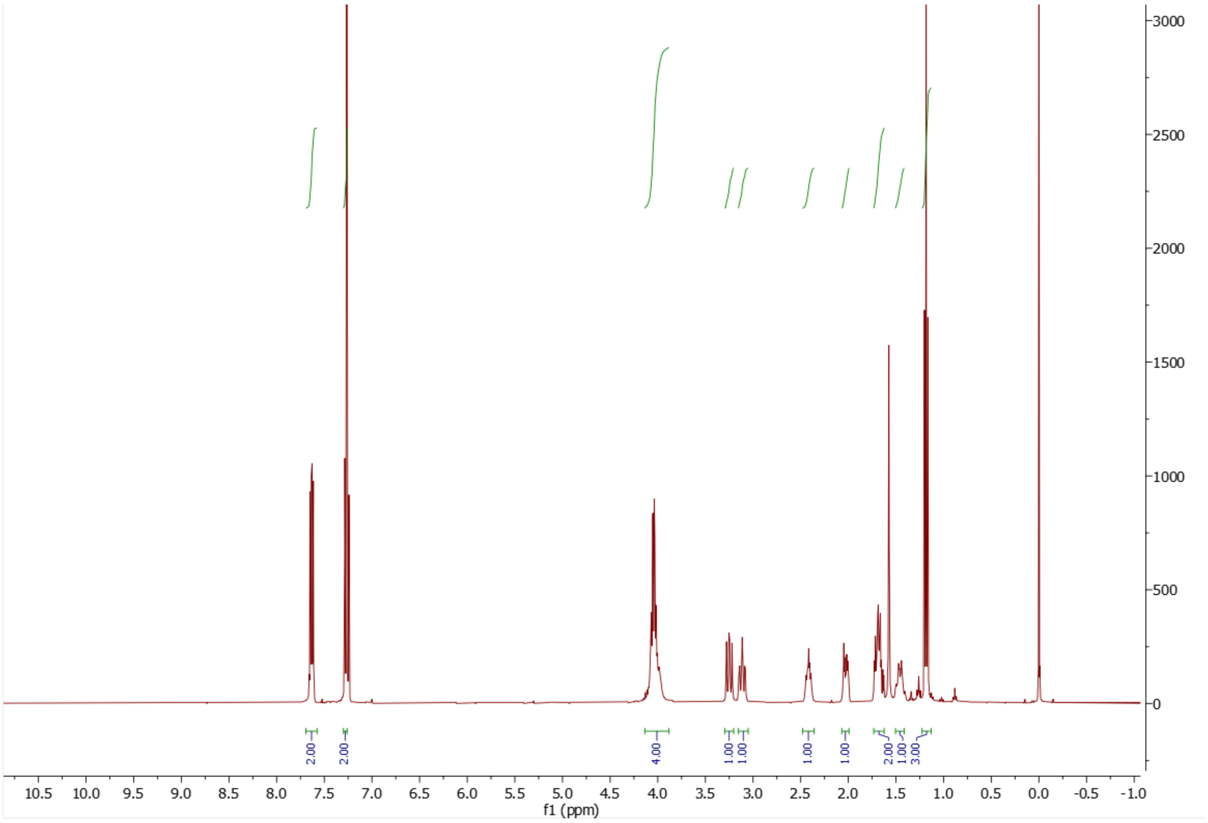

Figure S47: ^{1}H NMR of **21l**.

Chromatogram and Results

Injection Details

Injection Name:	**SN-204**	*Run Time (min):*	**8,00**
Vial Number:	**RB3**	*Injection Volume:*	**2,00**
Injection Type:	**Unknown**	*Channel:*	**UV_VIS_2**
Calibration Level:		*Wavelength:*	**254**
Instrument Method:	**Metoda_uplc**	*Bandwidth:*	**16**
Processing Method:	**Basic Quantitative**	*Dilution Factor:*	**1,0000**
Injection Date/Time:	**30.avg..21 14:32**	*Sample Weight:*	**1,0000**

Chromatogram

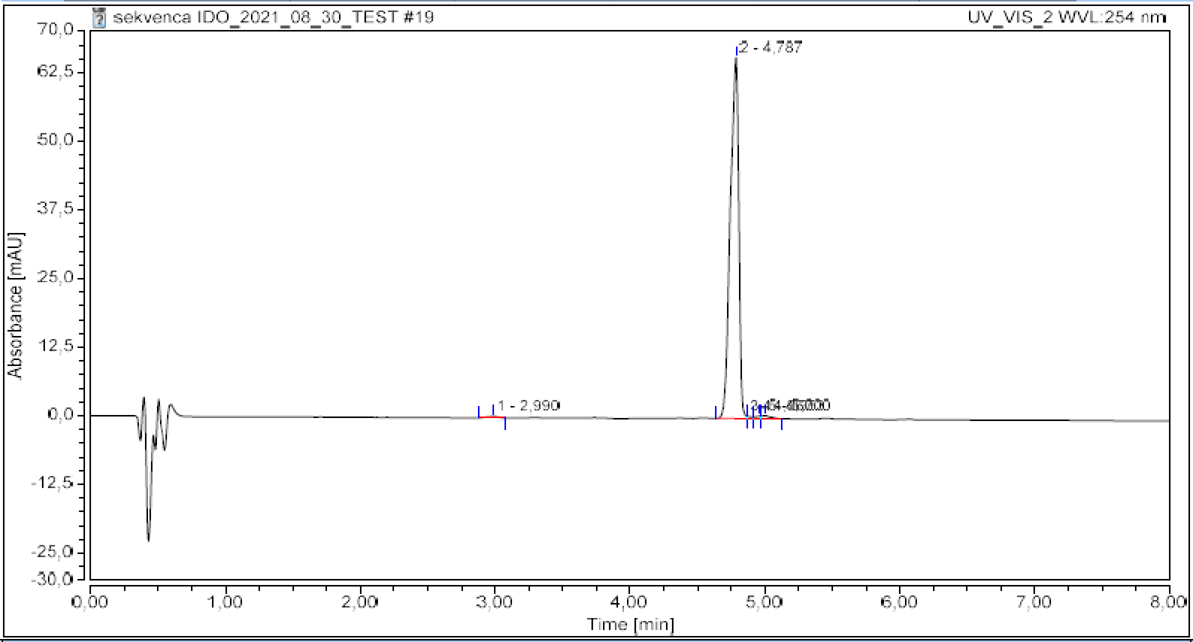

Integration Results

No.	Peak Name	Retention Time min	Area mAU*min	Height mAU	Relative Area %	Relative Height %	Amount n.a.
1		2,990	0,023	0,267	0,47	0,40	n.a.
2		4,787	4,721	65,714	97,92	97,51	n.a.
3		4,867	0,015	0,447	0,31	0,66	n.a.
4		4,953	0,022	0,425	0,46	0,63	n.a.
5		5,000	0,040	0,539	0,84	0,80	n.a.
**Total:**			**4,821**	**67,392**	**100,00**	**100,00**	

Figure S48: HPLC of **21l**.

21m

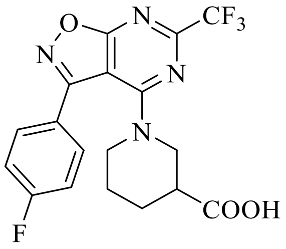

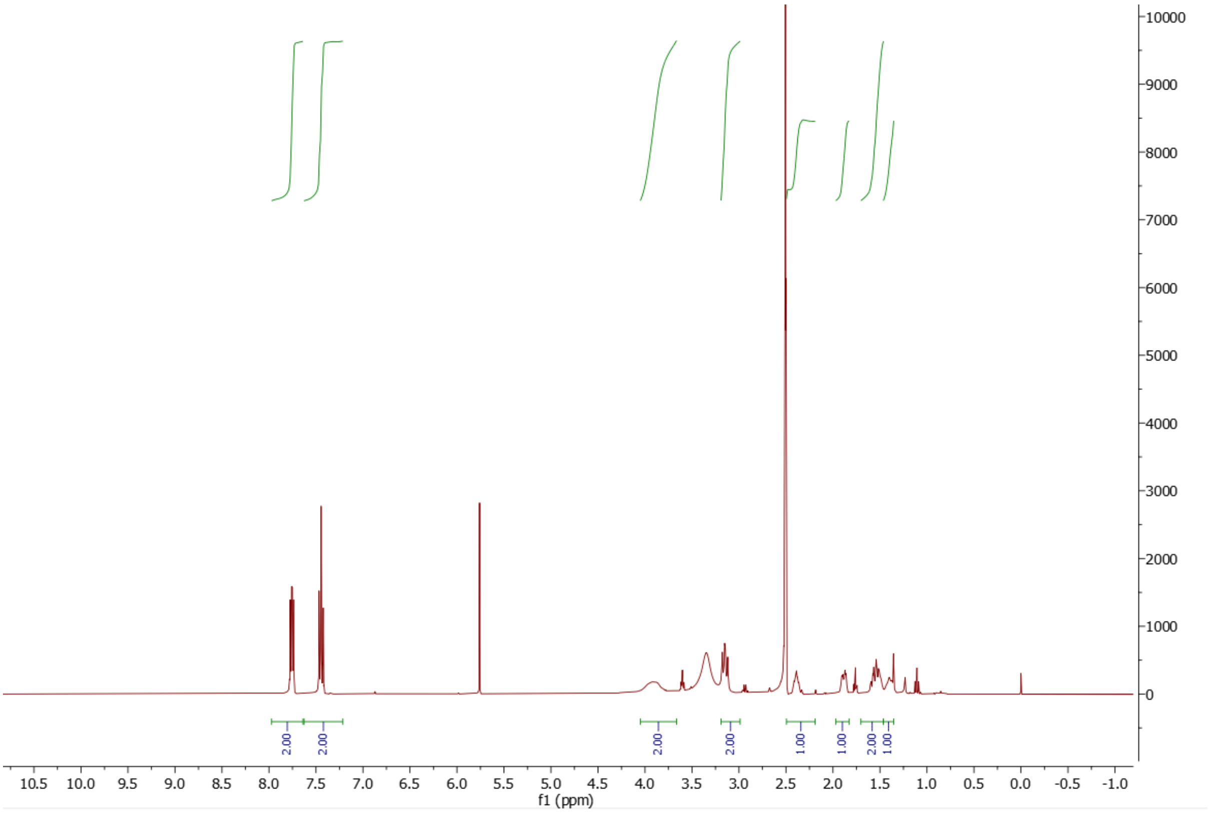

Figure S49: ^{1}H NMR of **21m**.

Chromatogram and Results

Injection Details			
Injection Name:	**SN-206**	*Run Time (min):*	**12,00**
Vial Number:	**BB3**	*Injection Volume:*	**2,00**
Injection Type:	**Unknown**	*Channel:*	**UV_VIS_2**
Calibration Level:		*Wavelength:*	**254**
Instrument Method:	**Metoda_uplc**	*Bandwidth:*	**16**
Processing Method:	**Basic Quantitative**	*Dilution Factor:*	**1,0000**
Injection Date/Time:	**02.sep..21 13:39**	*Sample Weight:*	**1,0000**

Chromatogram

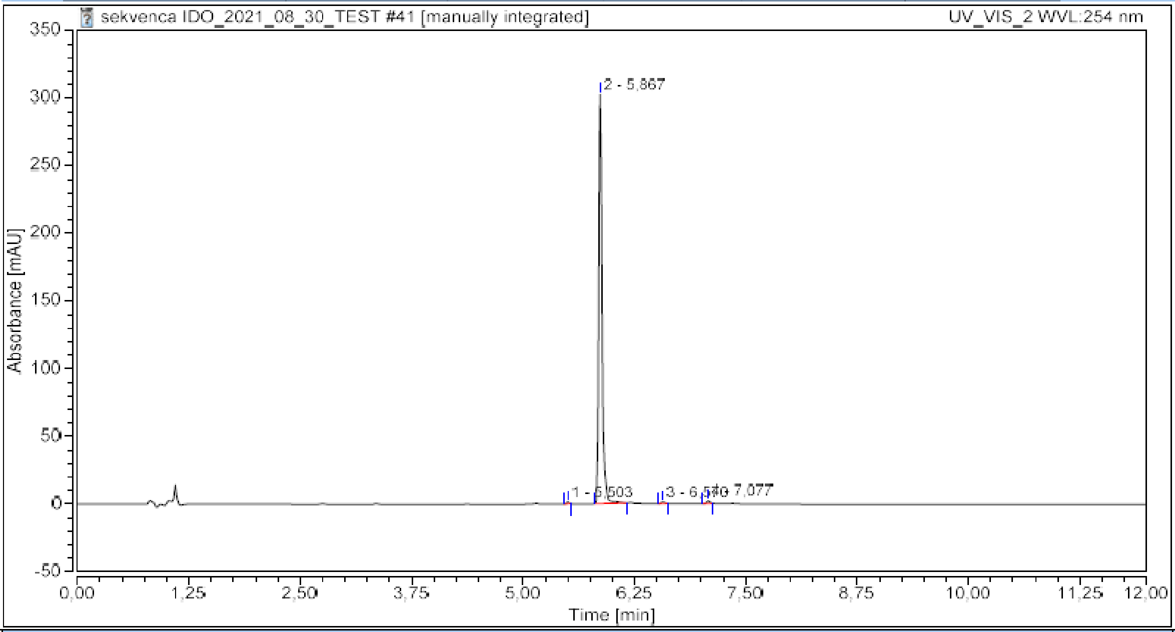

Integration Results

No.	Peak Name	Retention Time min	Area mAU*min	Height mAU	Relative Area %	Relative Height %	Amount n.a.
1		5,503	0,053	1,417	0,40	0,46	n.a.
2		5,867	12,983	302,459	98,61	98,42	n.a.
3		6,570	0,049	1,189	0,37	0,39	n.a.
4		7,077	0,081	2,238	0,61	0,73	n.a.
**Total:**			**13,165**	**307,303**	**100,00**	**100,00**	

Figure S50: HPLC of **21m**.

21n

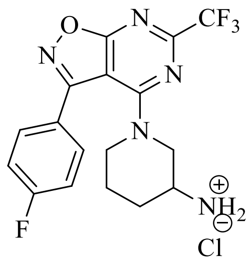

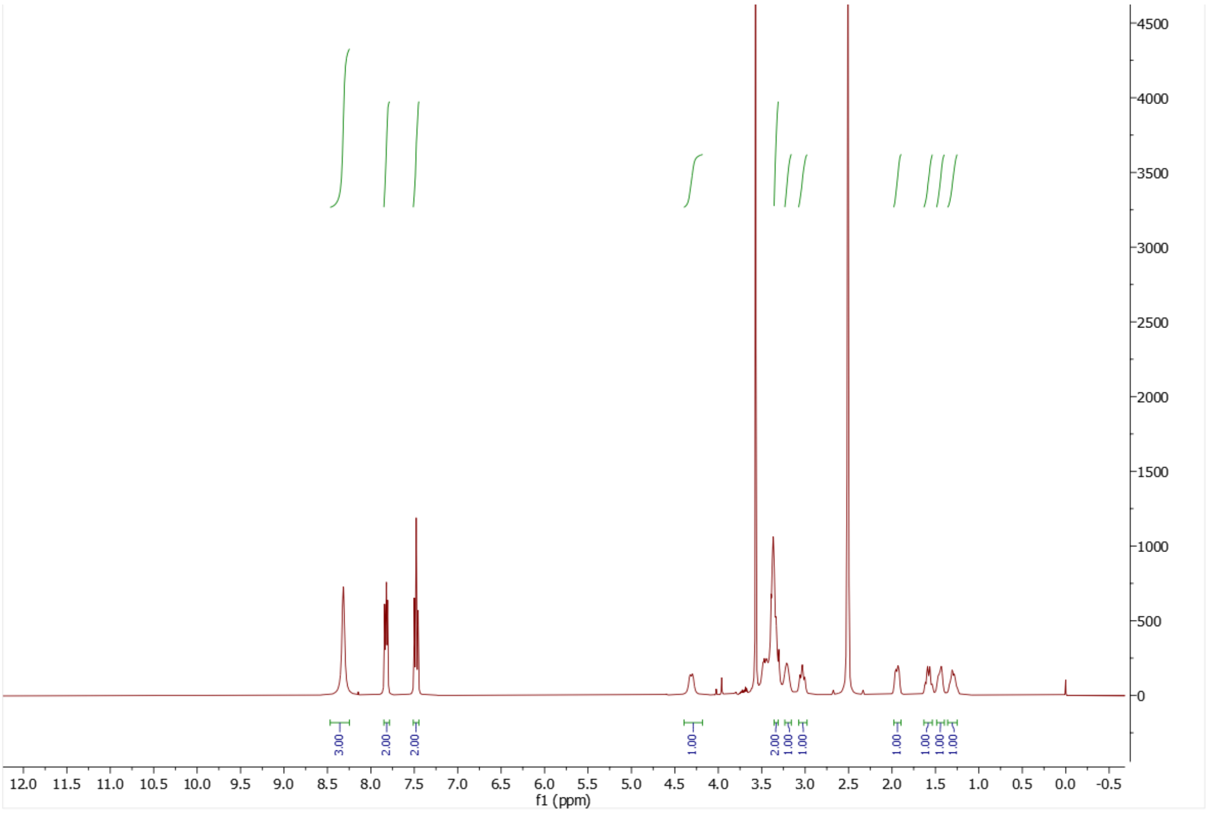

Figure S51: ^{1}H NMR of **21n**.

Chromatogram and Results

Injection Details

Injection Name:	**SN-205**	*Run Time (min):*	**12,00**
Vial Number:	**BB2**	*Injection Volume:*	**2,00**
Injection Type:	**Unknown**	*Channel:*	**UV_VIS_2**
Calibration Level:		*Wavelength:*	**254**
Instrument Method:	**Metoda_uplc**	*Bandwidth:*	**16**
Processing Method:	**Basic Quantitative**	*Dilution Factor:*	**1,0000**
Injection Date/Time:	**02.sep..21 13:21**	*Sample Weight:*	**1,0000**

Chromatogram

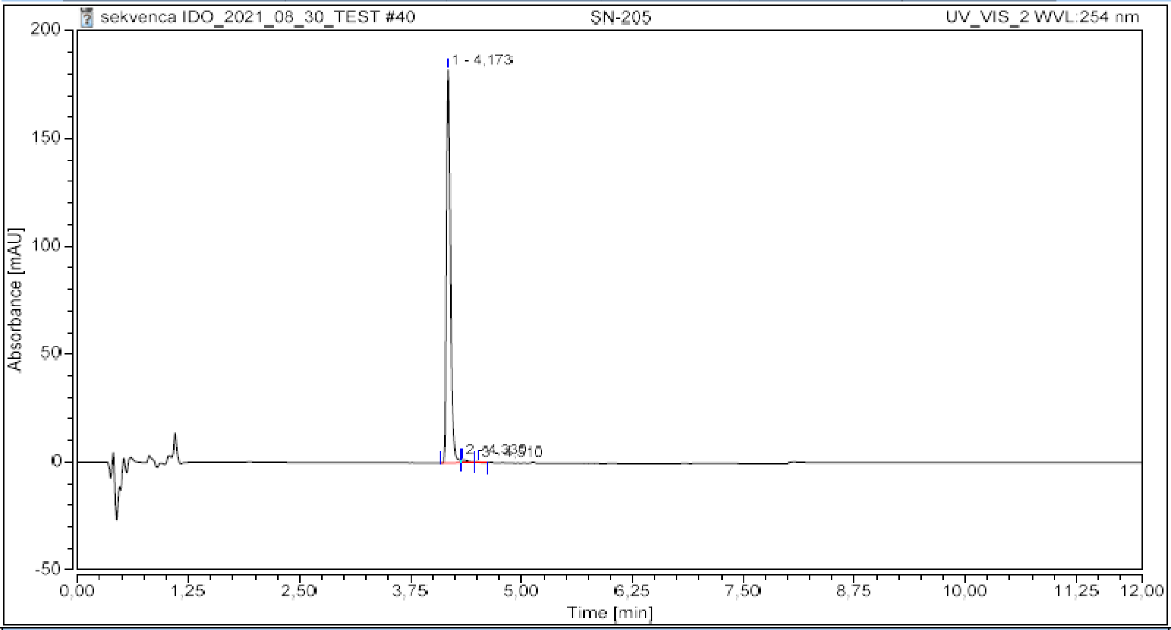

Integration Results

No.	Peak Name	Retention Time<br>min	Area<br>mAU*min	Height<br>mAU	Relative Area<br>%	Relative Height<br>%	Amount<br>n.a.
1		4,173	9,156	182,056	98,28	99,04	n.a.
2		4,330	0,130	1,267	1,39	0,69	n.a.
3		4,510	0,030	0,499	0,32	0,27	n.a.
**Total:**			**9,316**	**183,823**	**100,00**	**100,00**	

Figure S52: HPLC of **21n**.

21o

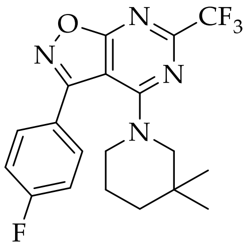

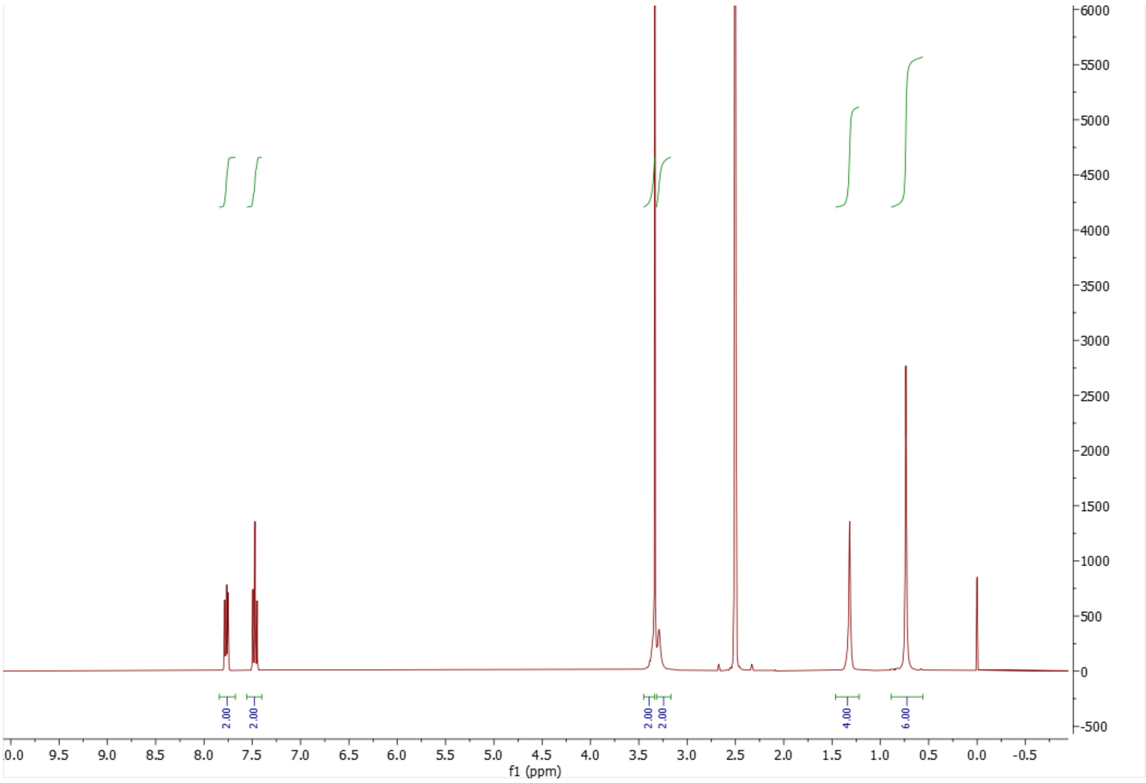

Figure S53: ^{1}H NMR of **21o**.

Chromatogram and Results

Injection Details

Injection Name:	**SKV-7**	*Run Time (min):*	**8,00**
Vial Number:	**RA7**	*Injection Volume:*	**2,00**
Injection Type:	**Unknown**	*Channel:*	**UV_VIS_2**
Calibration Level:		*Wavelength:*	**254**
Instrument Method:	**UPLC_Nika_splosna_metoda_TFA_MeCN**	*Bandwidth:*	**16**
Processing Method:	**Quantitative**	*Dilution Factor:*	**1,0000**
Injection Date/Time:	**20.mar..23 13:56**	*Sample Weight:*	**1,0000**

Chromatogram

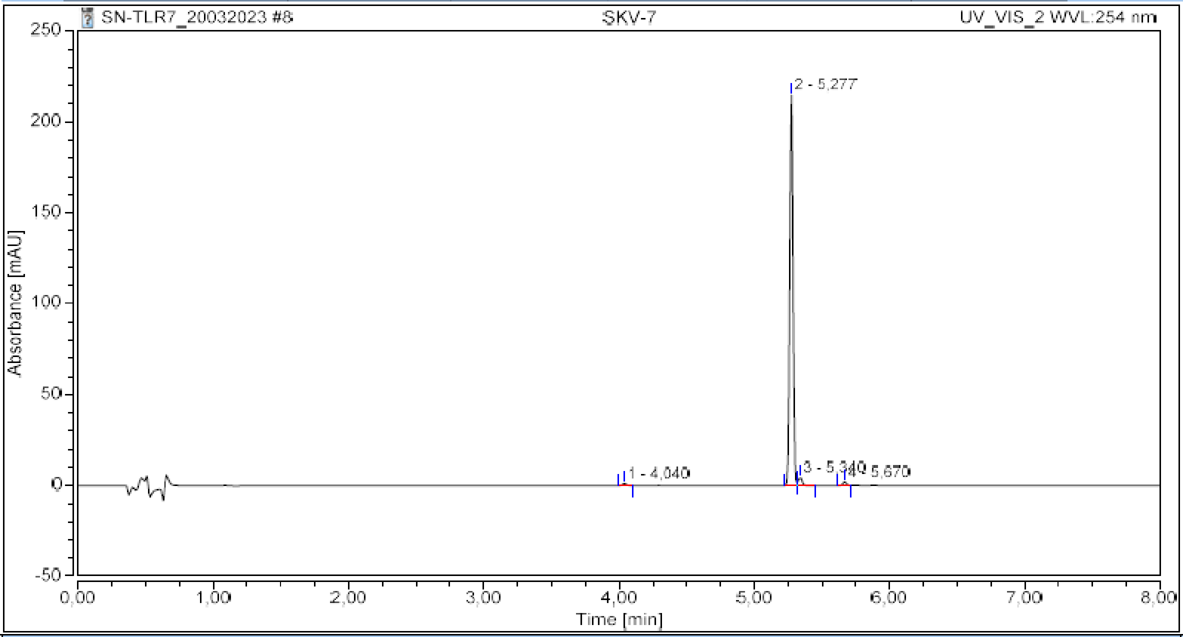

Integration Results

No.	Peak Name	Retention Time<br>min	Area<br>mAU*min	Height<br>mAU	Relative Area<br>%	Relative Height<br>%	Amount<br>n.a.
1		4,040	0,038	1,188	0,60	0,53	n.a.
2		5,277	6,103	214,756	96,23	96,52	n.a.
3		5,340	0,143	4,534	2,25	2,04	n.a.
4		5,670	0,058	2,025	0,92	0,91	n.a.
**Total:**			**6,342**	**222,503**	**100,00**	**100,00**	

Figure S54: HPLC of **21o**.

21p

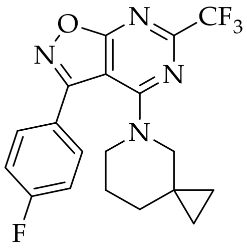

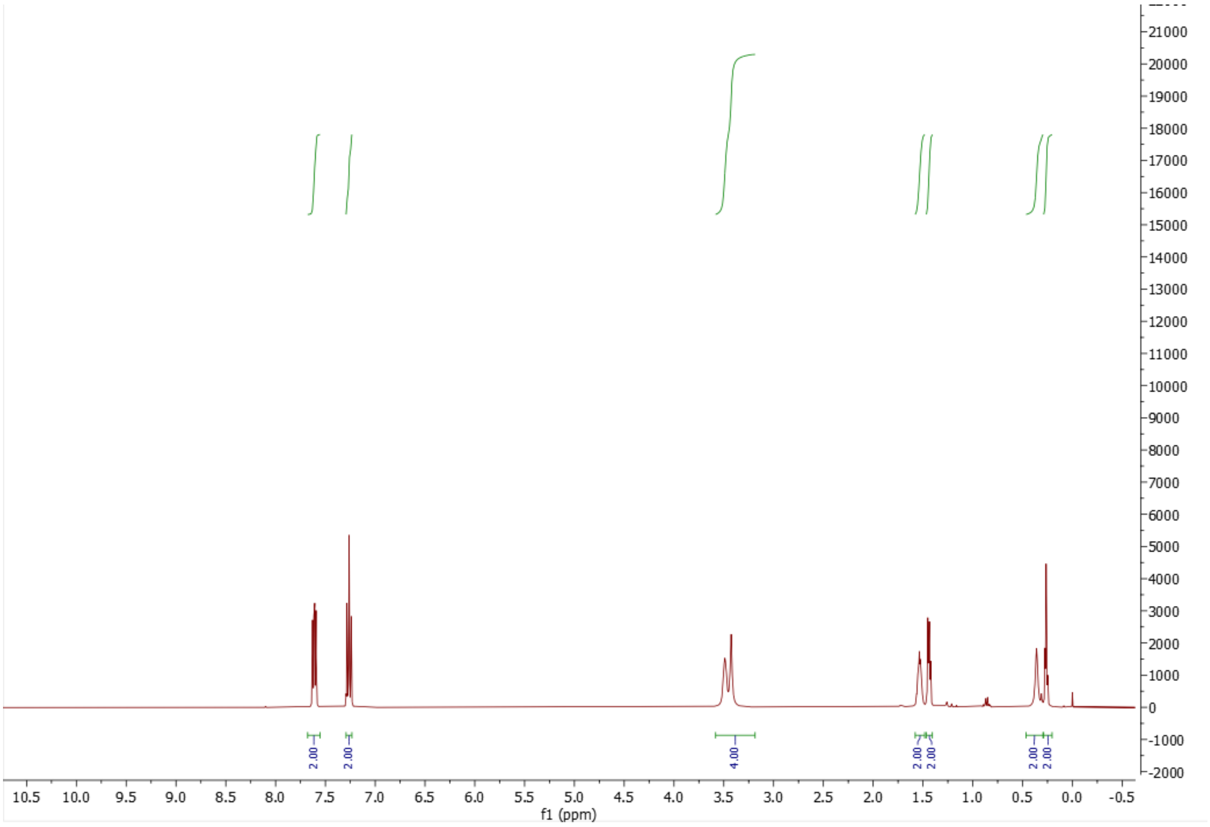

Figure S55: ^{1}H NMR of **21p**.

Chromatogram and Results

Injection Details

Injection Name:	**SKV-9**	*Run Time (min):*	**8,00**
Vial Number:	**RB1**	*Injection Volume:*	**2,00**
Injection Type:	**Unknown**	*Channel:*	**UV_VIS_2**
Calibration Level:		*Wavelength:*	**254**
Instrument Method:	**UPLC_Nika_splosna_metoda_TFA_MeCN**	*Bandwidth:*	**16**
Processing Method:	**Quantitative**	*Dilution Factor:*	**1,0000**
Injection Date/Time:	**20.mar..23 14:22**	*Sample Weight:*	**1,0000**

Chromatogram

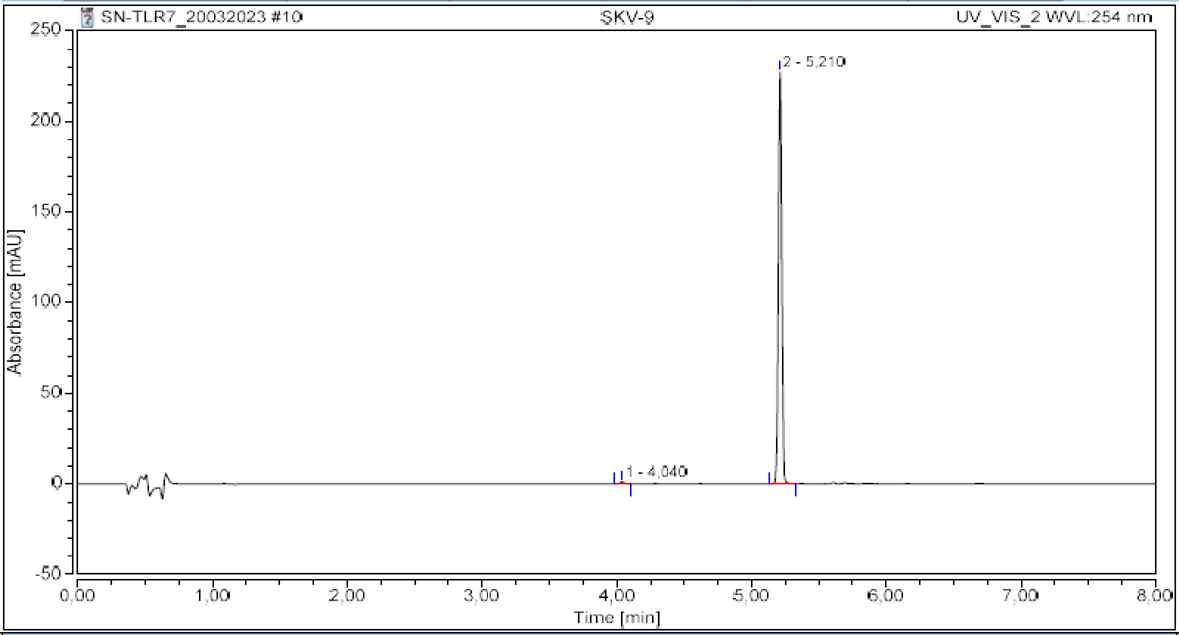

Integration Results

No.	Peak Name	Retention Time min	Area mAU*min	Height mAU	Relative Area %	Relative Height %	Amount n.a.
1		4,040	0,036	1,111	0,55	0,49	n.a.
2		5,210	6,562	227,365	99,45	99,51	n.a.
**Total:**			**6,598**	**228,476**	**100,00**	**100,00**	

Figure S56: HPLC of **21p**.

21q

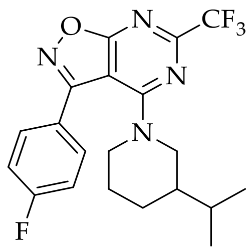

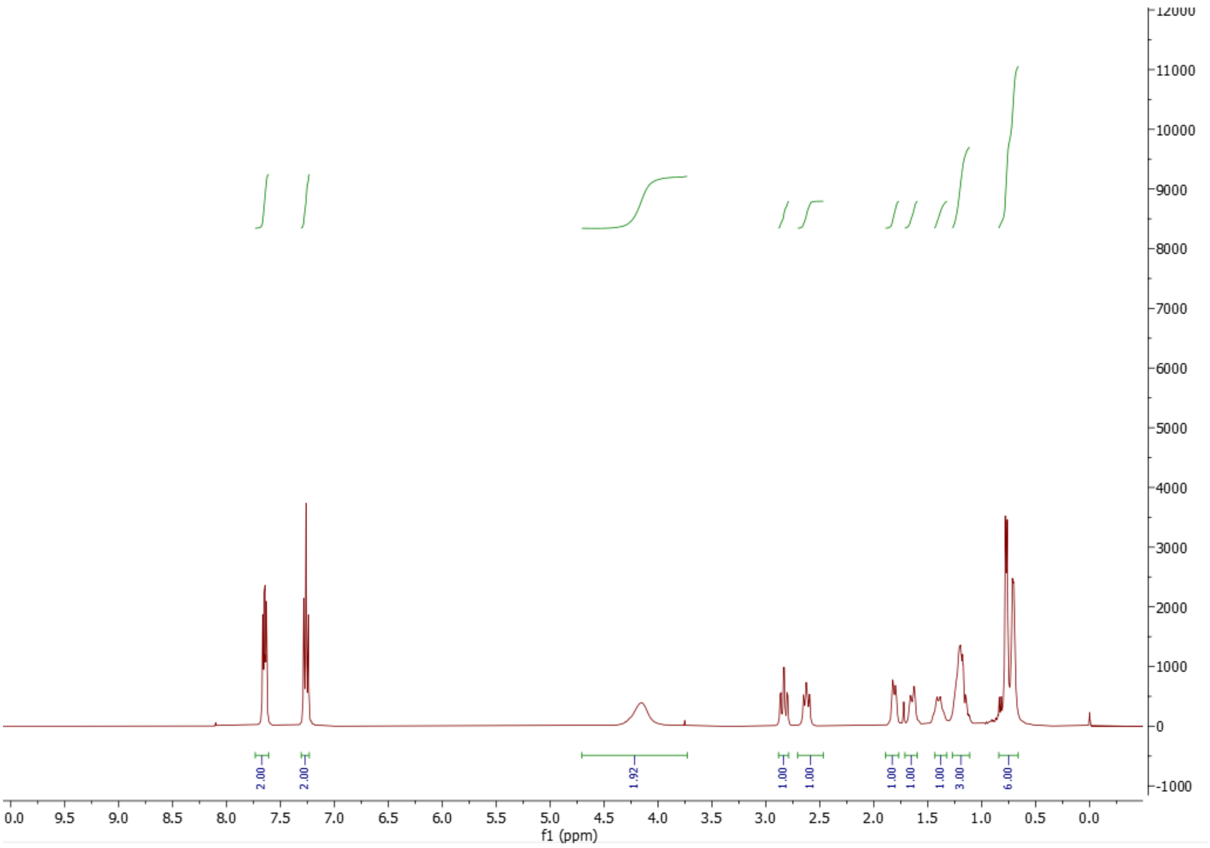

Figure S57: ^{1}H NMR of **21q**.

Chromatogram and Results

Injection Details

Injection Name:	**SKV-10**	*Run Time (min):*	**8,00**
Vial Number:	**RB2**	*Injection Volume:*	**2,00**
Injection Type:	**Unknown**	*Channel:*	**UV_VIS_2**
Calibration Level:		*Wavelength:*	**254**
Instrument Method:	**UPLC_Nika_splosna_metoda_TFA_MeCN**	*Bandwidth:*	**16**
Processing Method:	**Quantitative**	*Dilution Factor:*	**1,0000**
Injection Date/Time:	**20.mar..23 14:36**	*Sample Weight:*	**1,0000**

Chromatogram

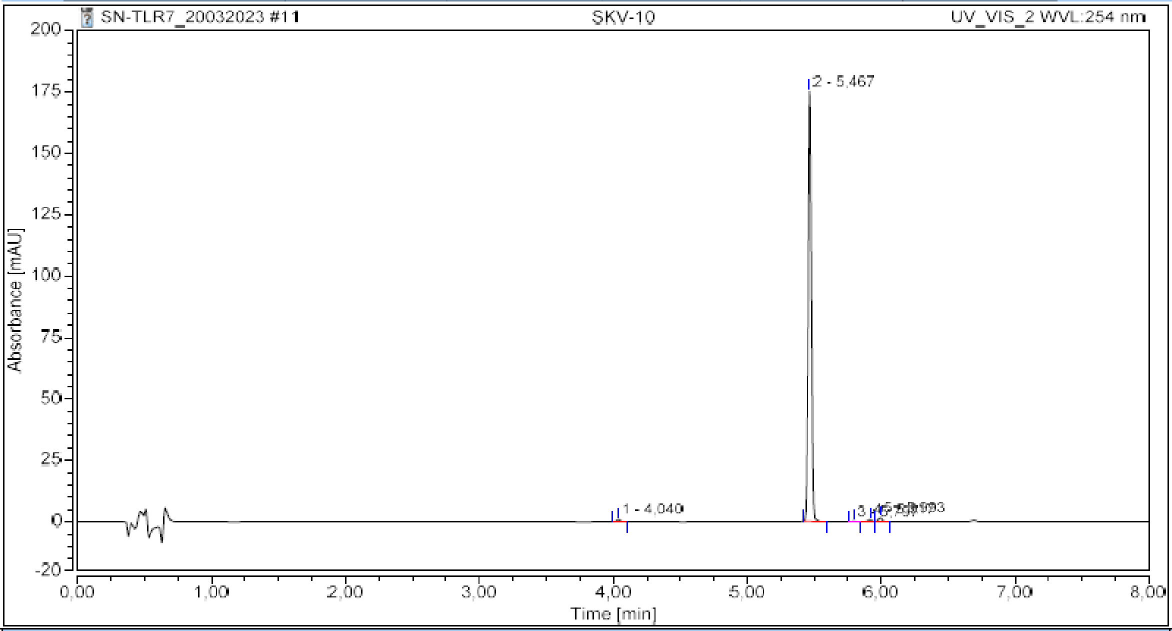

Integration Results

No.	Peak Name	Retention Time min	Area mAU*min	Height mAU	Relative Area %	Relative Height %	Amount n.a.
1		4,040	0,031	0,977	0,60	0,55	n.a.
2		5,467	5,047	175,031	97,58	97,82	n.a.
3		5,797	0,007	0,225	0,13	0,13	n.a.
4		5,917	0,033	0,948	0,64	0,53	n.a.
5		5,993	0,054	1,752	1,05	0,98	n.a.
**Total:**			**5,172**	**178,933**	**100,00**	**100,00**	

Figure S58: HPLC of **21q**.

21r

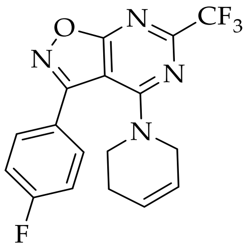

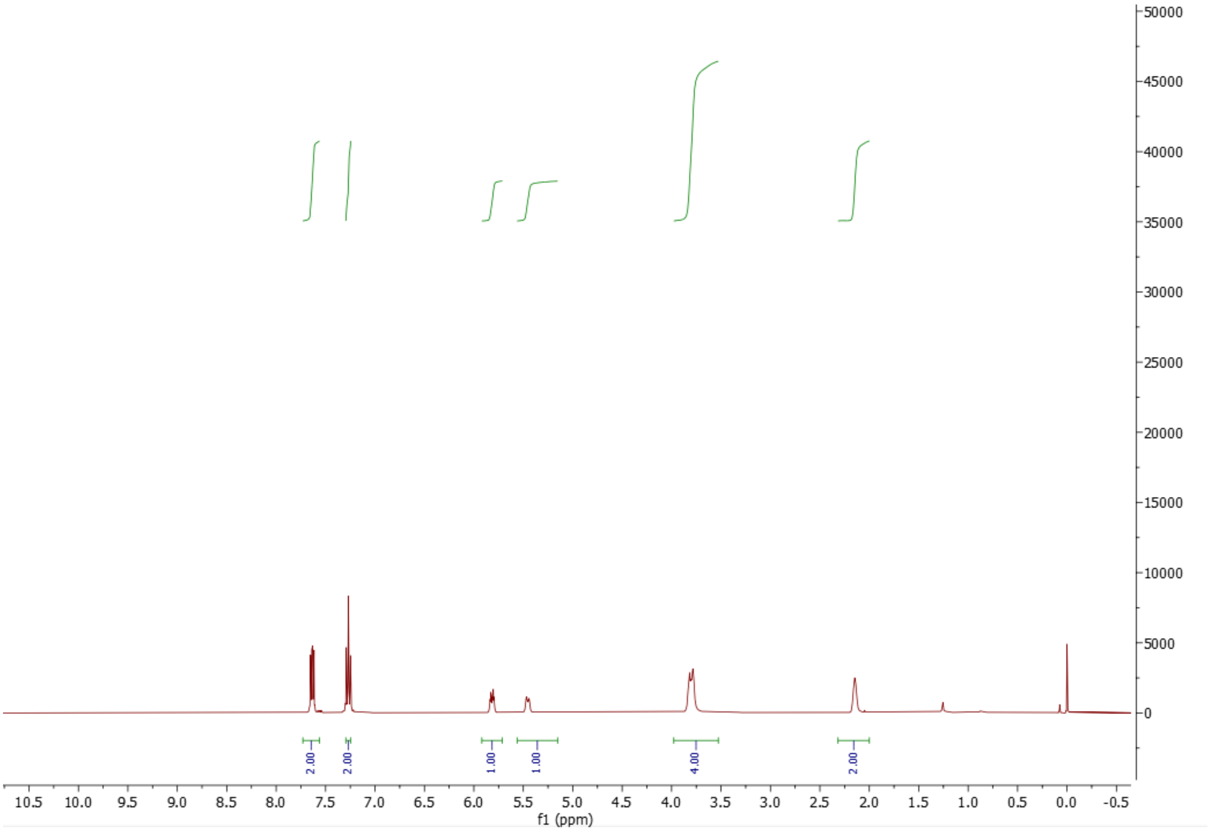

Figure S59: ^{1}H NMR of **21r**.

Chromatogram and Results

Injection Details

Injection Name:	**SOK-8**	*Run Time (min):*	**12,00**
Vial Number:	**GA4**	*Injection Volume:*	**2,00**
Injection Type:	**Unknown**	*Channel:*	**UV_VIS_2**
Calibration Level:		*Wavelength:*	**254**
Instrument Method:	**Metoda_uplc**	*Bandwidth:*	**16**
Processing Method:	**Basic Quantitative**	*Dilution Factor:*	**1,0000**
Injection Date/Time:	**16.mar..22 12:08**	*Sample Weight:*	**1,0000**

Chromatogram

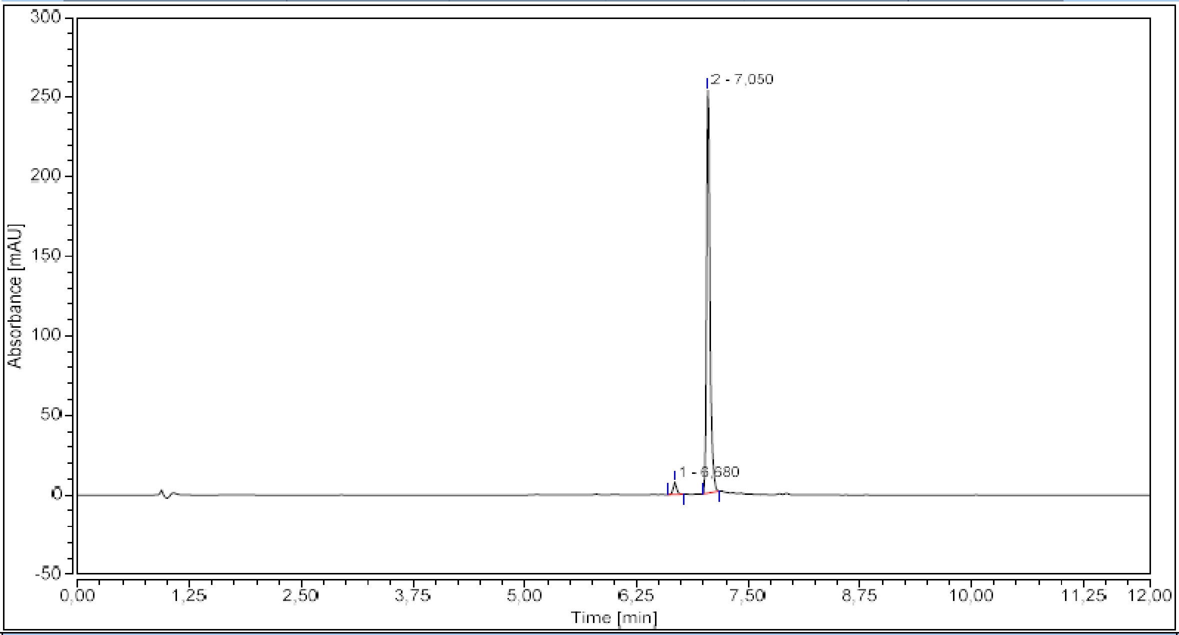

Integration Results

No.	Peak Name	Retention Time min	Area mAU*min	Height mAU	Relative Area %	Relative Height %	Amount n.a.
1		6,680	0,322	7,566	2,84	2,90	n.a.
2		7,050	11,018	253,190	97,16	97,10	n.a.
**Total:**			**11,340**	**260,756**	**100,00**	**100,00**	

Figure S60: HPLC of **21r**.

21s

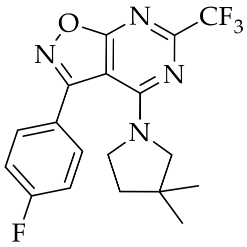

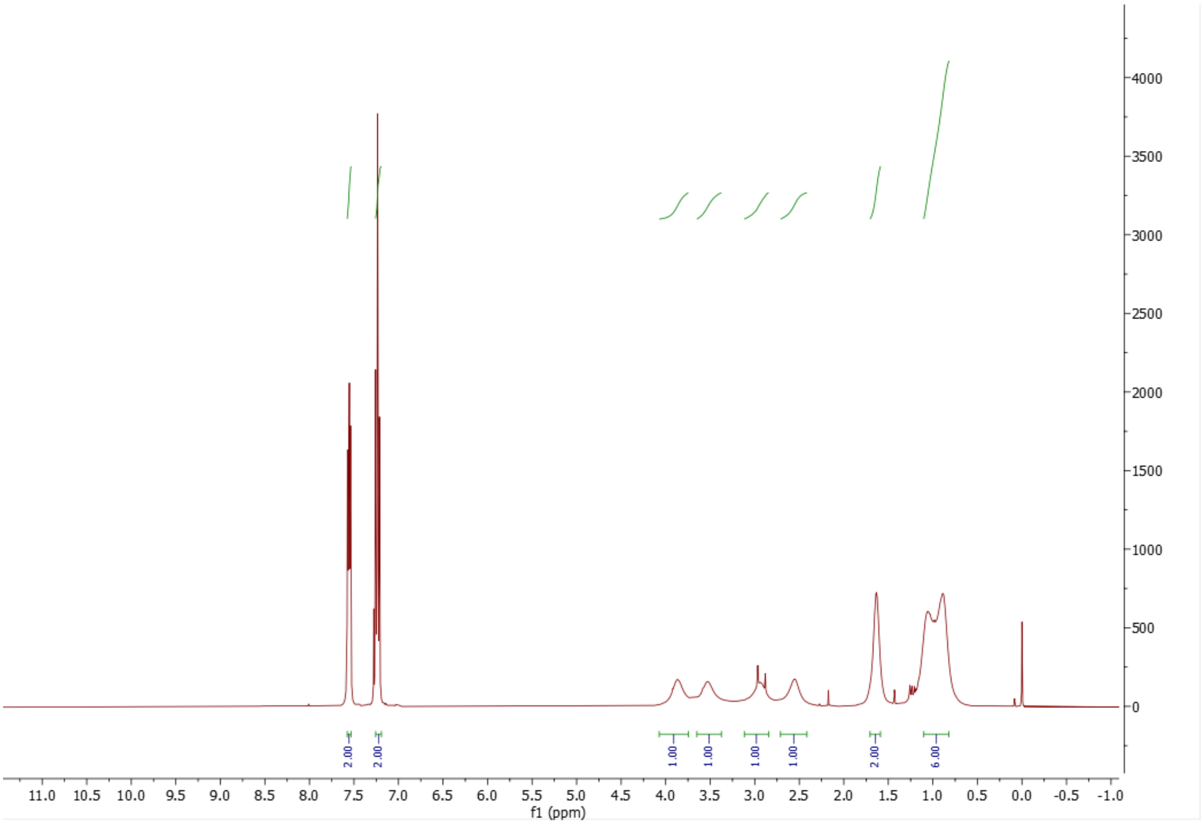

Figure S61: ^{1}H NMR of **21s.**

Chromatogram and Results

Injection Details

Injection Name:	**SOK-6**	*Run Time (min):*	**12,00**
Vial Number:	**GA2**	*Injection Volume:*	**2,00**
Injection Type:	**Unknown**	*Channel:*	**UV_VIS_2**
Calibration Level:		*Wavelength:*	**254**
Instrument Method:	**Metoda_uplc**	*Bandwidth:*	**16**
Processing Method:	**Basic Quantitative**	*Dilution Factor:*	**1,0000**
Injection Date/Time:	**16.mar..22 11:33**	*Sample Weight:*	**1,0000**

Chromatogram

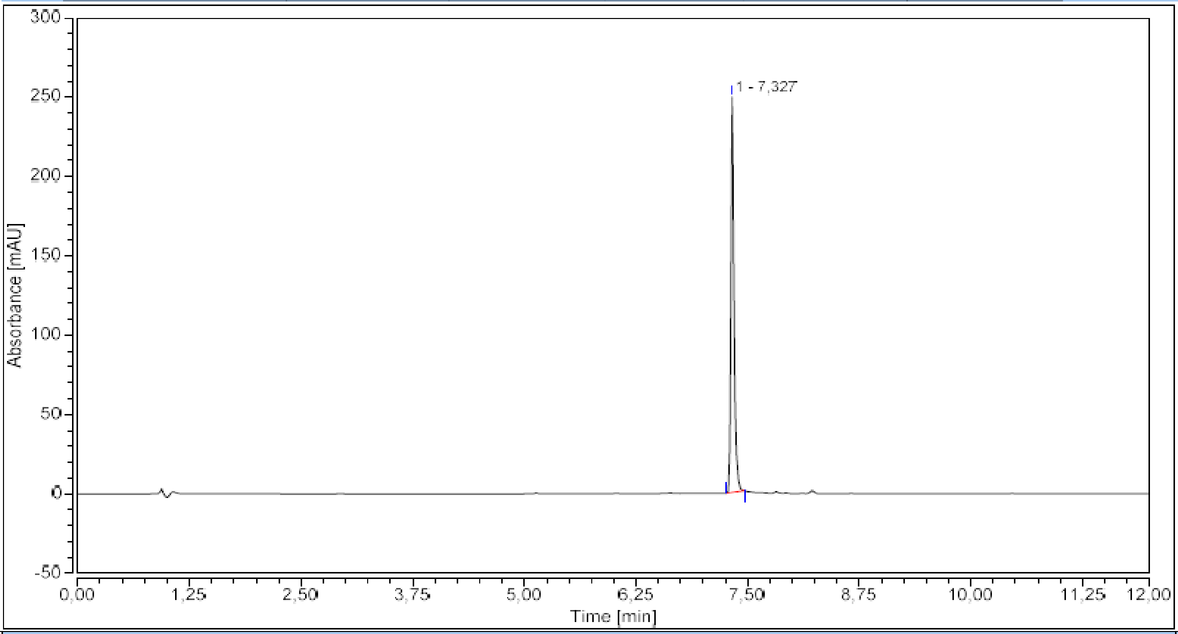

Integration Results

No.	Peak Name	Retention Time min	Area mAU*min	Height mAU	Relative Area %	Relative Height %	Amount n.a.
1		7,327	10,942	248,956	100,00	100,00	n.a.
**Total:**			**10,942**	**248,956**	**100,00**	**100,00**	

Figure S62: HPLC of **21s**.

21t

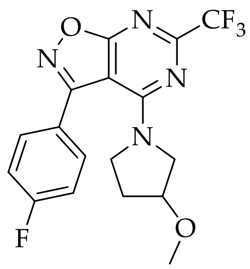

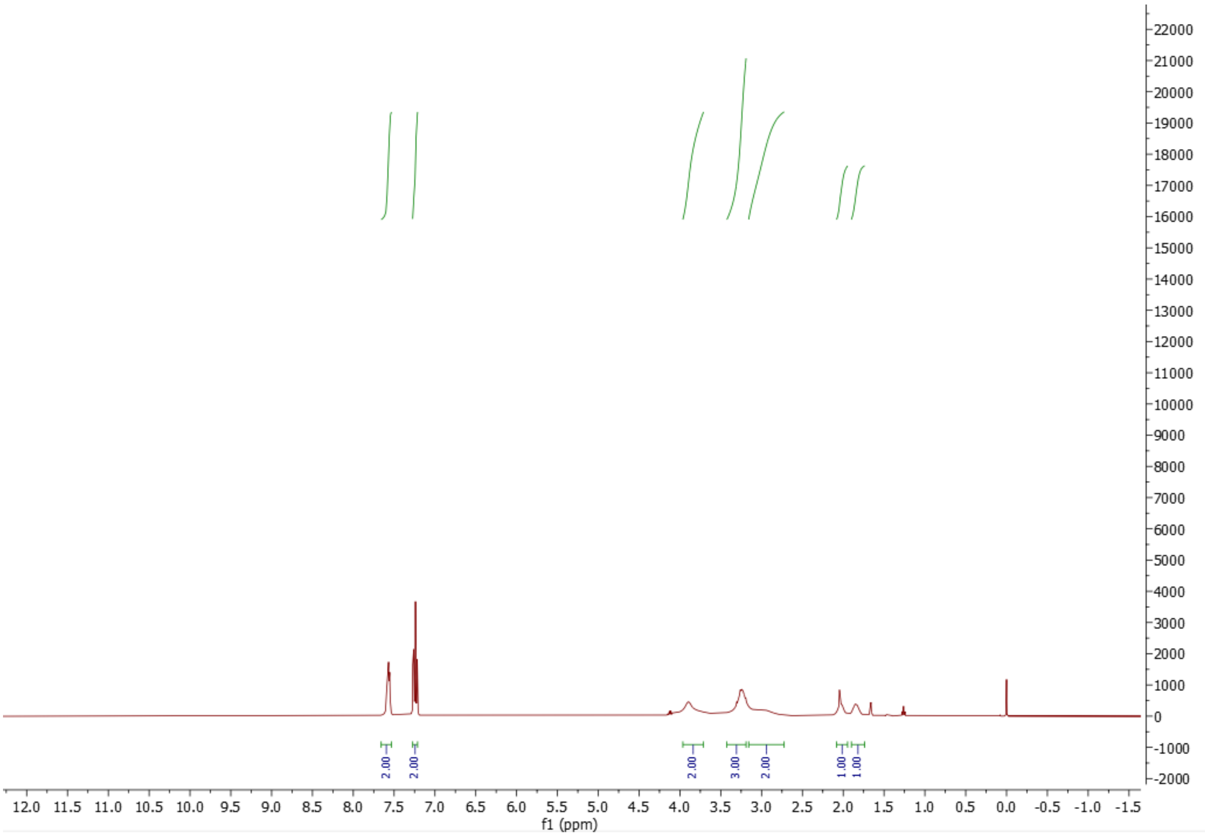

Figure S63: ^{1}H NMR of **21t**.

Chromatogram and Results

Injection Details

Injection Name:	**SBS-9**	*Run Time (min):*	**12,00**
Vial Number:	**GB4**	*Injection Volume:*	**2,00**
Injection Type:	**Unknown**	*Channel:*	**UV_VIS_2**
Calibration Level:		*Wavelength:*	**254**
Instrument Method:	**Metoda_uplc**	*Bandwidth:*	**16**
Processing Method:	**Basic Quantitative**	*Dilution Factor:*	**1,0000**
Injection Date/Time:	**04.nov..21 13:38**	*Sample Weight:*	**1,0000**

Chromatogram

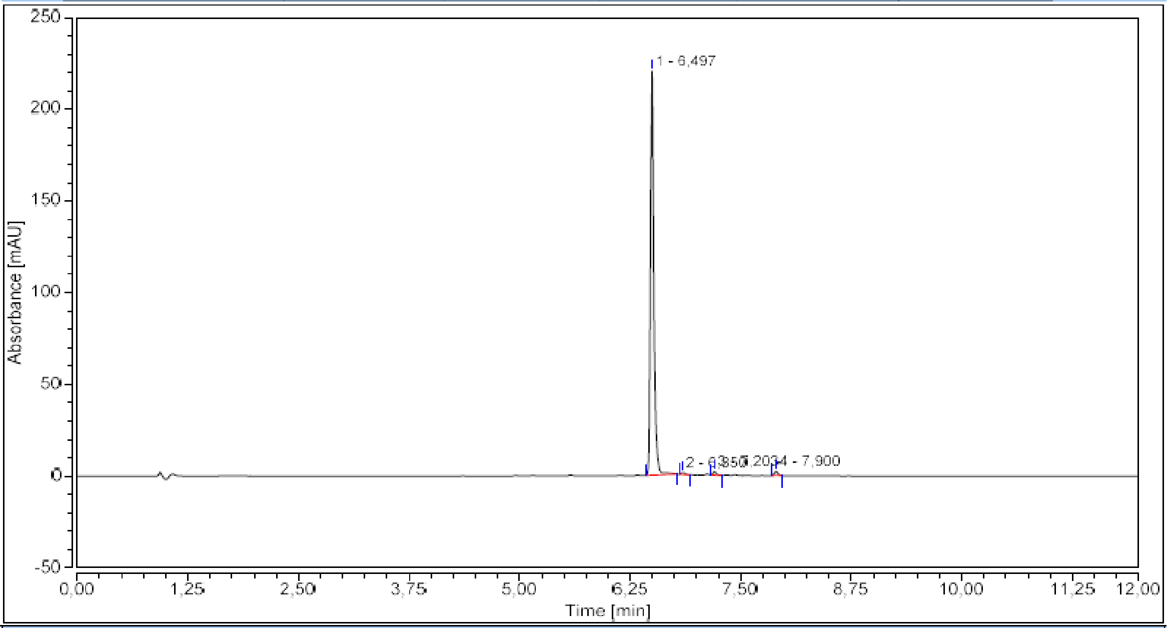

Integration Results

No.	Peak Name	Retention Time min	Area mAU*min	Height mAU	Relative Area %	Relative Height %	Amount n.a.
1		6,497	9,635	220,311	97,59	97,47	n.a.
2		6,850	0,043	1,102	0,43	0,49	n.a.
3		7,203	0,090	2,178	0,91	0,96	n.a.
4		7,900	0,105	2,429	1,06	1,07	n.a.
**Total:**			**9,873**	**226,021**	**100,00**	**100,00**	

Figure S64: HPLC of **21t**.

21u

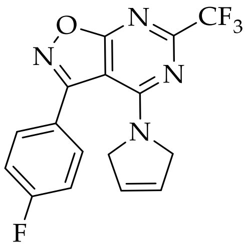

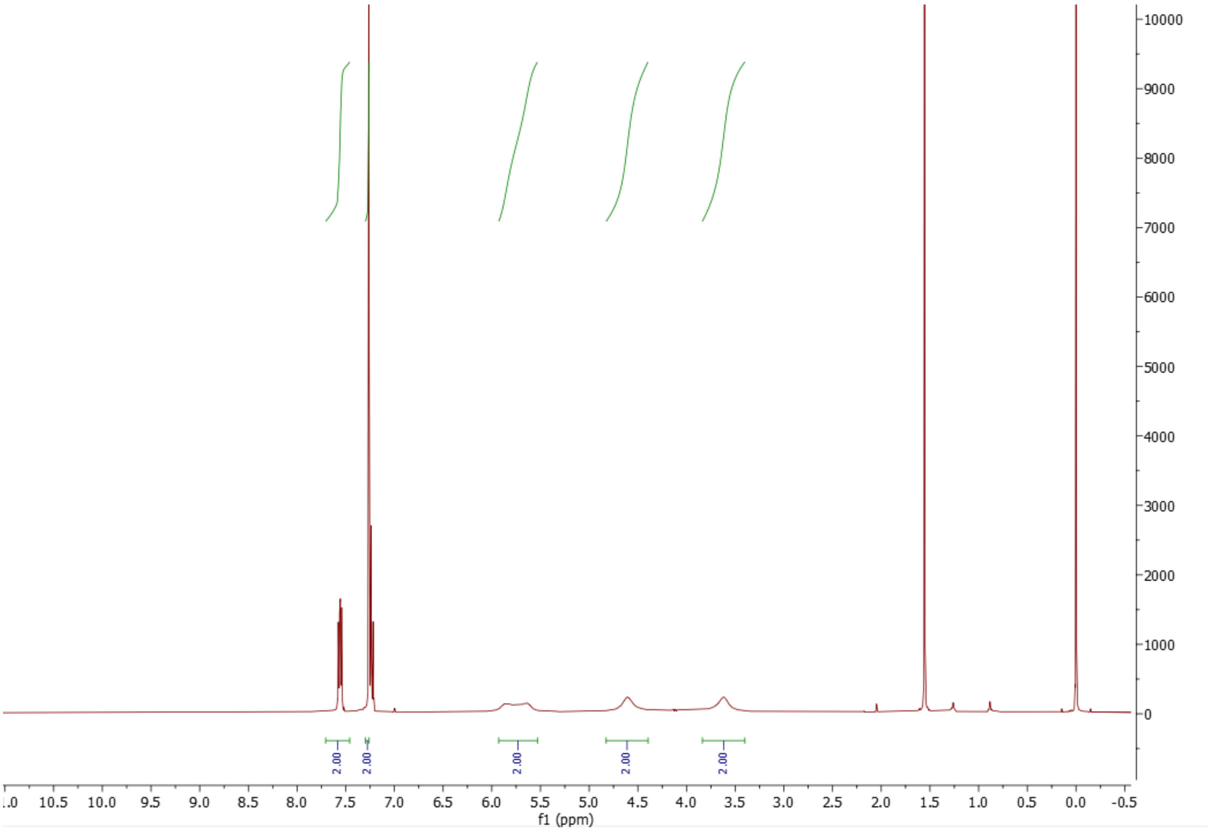

Figure S65: ^{1}H NMR of **21u**.

Chromatogram and Results

Injection Details

Injection Name:	**SOK-9.2**	*Run Time (min):*	**12,00**
Vial Number:	**GA7**	*Injection Volume:*	**2,00**
Injection Type:	**Unknown**	*Channel:*	**UV_VIS_2**
Calibration Level:		*Wavelength:*	**254**
Instrument Method:	**Metoda_uplc**	*Bandwidth:*	**16**
Processing Method:	**Basic Quantitative**	*Dilution Factor:*	**1,0000**
Injection Date/Time:	**16.mar..22 13:02**	*Sample Weight:*	**1,0000**

Chromatogram

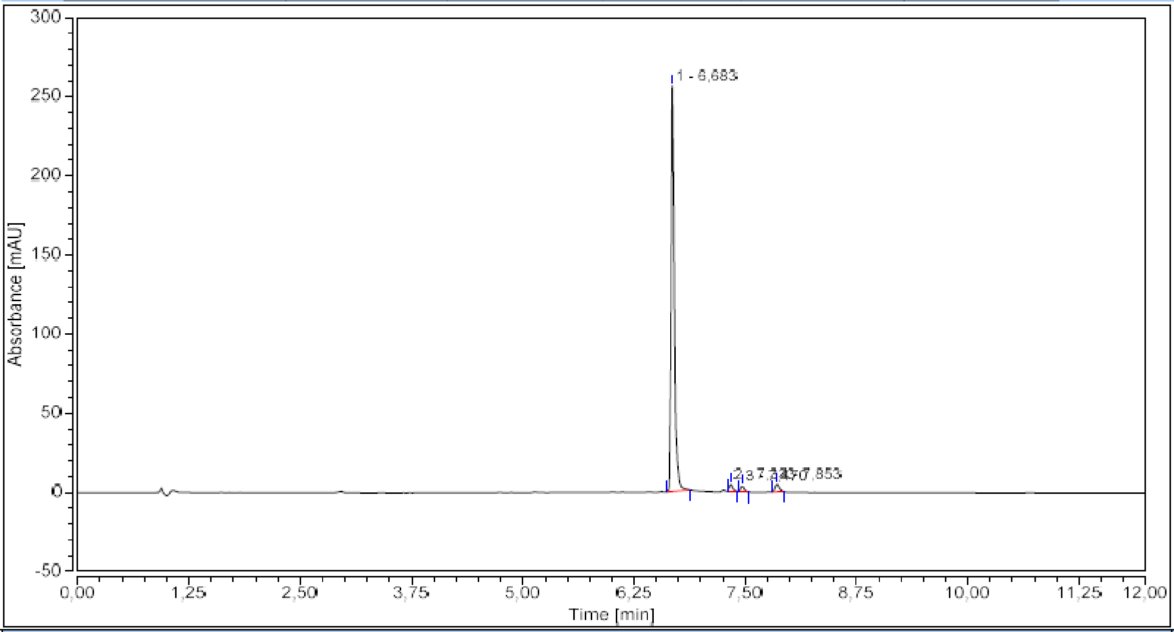

Integration Results

No.	Peak Name	Retention Time min	Area mAU*min	Height mAU	Relative Area %	Relative Height %	Amount n.a.
1		6,683	11,310	256,003	95,14	95,24	n.a.
2		7,333	0,176	4,337	1,48	1,61	n.a.
3		7,470	0,153	3,520	1,29	1,31	n.a.
4		7,853	0,249	4,924	2,10	1,83	n.a.
**Total:**			**11,888**	**268,784**	**100,00**	**100,00**	

Figure S66: HPLC of **21u**.

21v

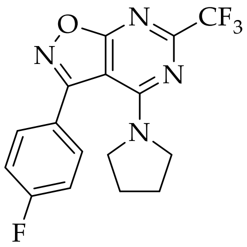

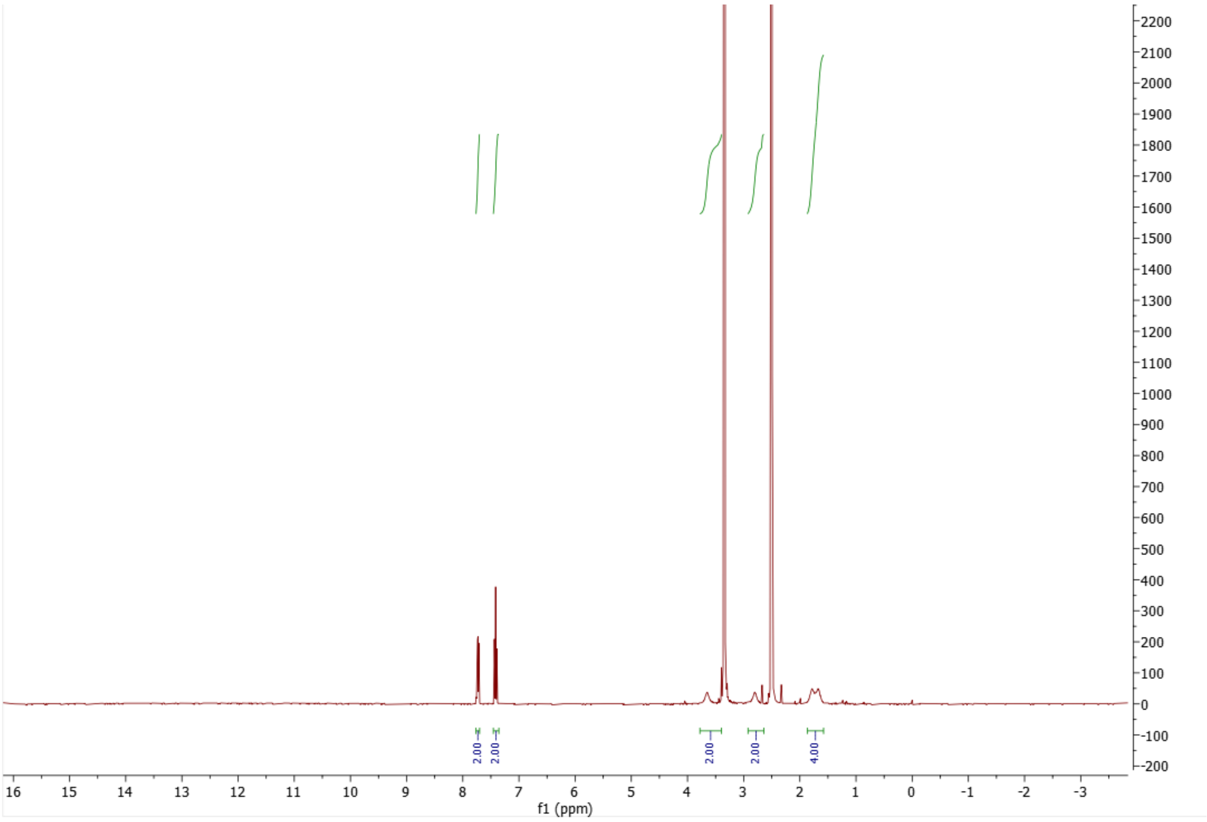

Figure S67: ^{1}H NMR of **21v**.

Chromatogram and Results

Injection Details

Injection Name:	**SBS-12**	*Run Time (min):*	**12,00**
Vial Number:	**GB7**	*Injection Volume:*	**2,00**
Injection Type:	**Unknown**	*Channel:*	**UV_VIS_2**
Calibration Level:		*Wavelength:*	**254**
Instrument Method:	**Metoda_uplc**	*Bandwidth:*	**16**
Processing Method:	**Basic Quantitative**	*Dilution Factor:*	**1,0000**
Injection Date/Time:	**04.nov..21 14:31**	*Sample Weight:*	**1,0000**

Chromatogram

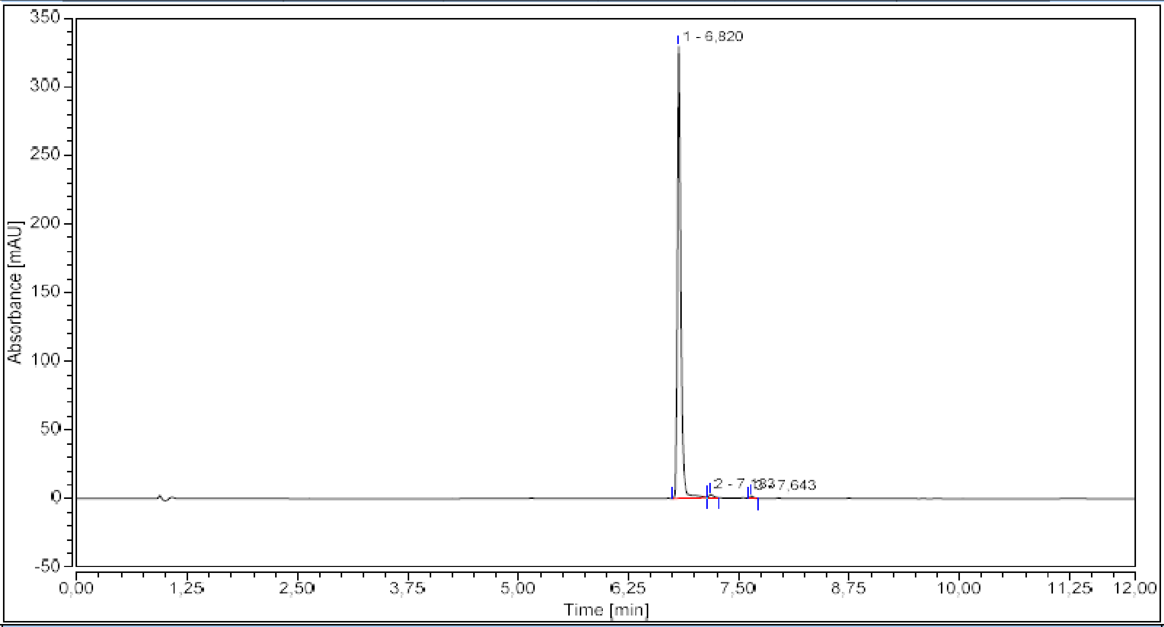

Integration Results

No.	Peak Name	Retention Time<br>min	Area<br>mAU*min	Height<br>mAU	Relative Area<br>%	Relative Height<br>%	Amount<br>n.a.
1		6,820	14,409	328,795	98,58	98,71	n.a.
2		7,183	0,135	2,503	0,92	0,75	n.a.
3		7,643	0,072	1,784	0,49	0,54	n.a.
**Total:**			**14,616**	**333,082**	**100,00**	**100,00**	

Figure S68: HPLC of **21v**.

21w

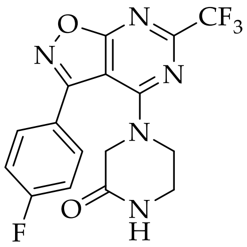

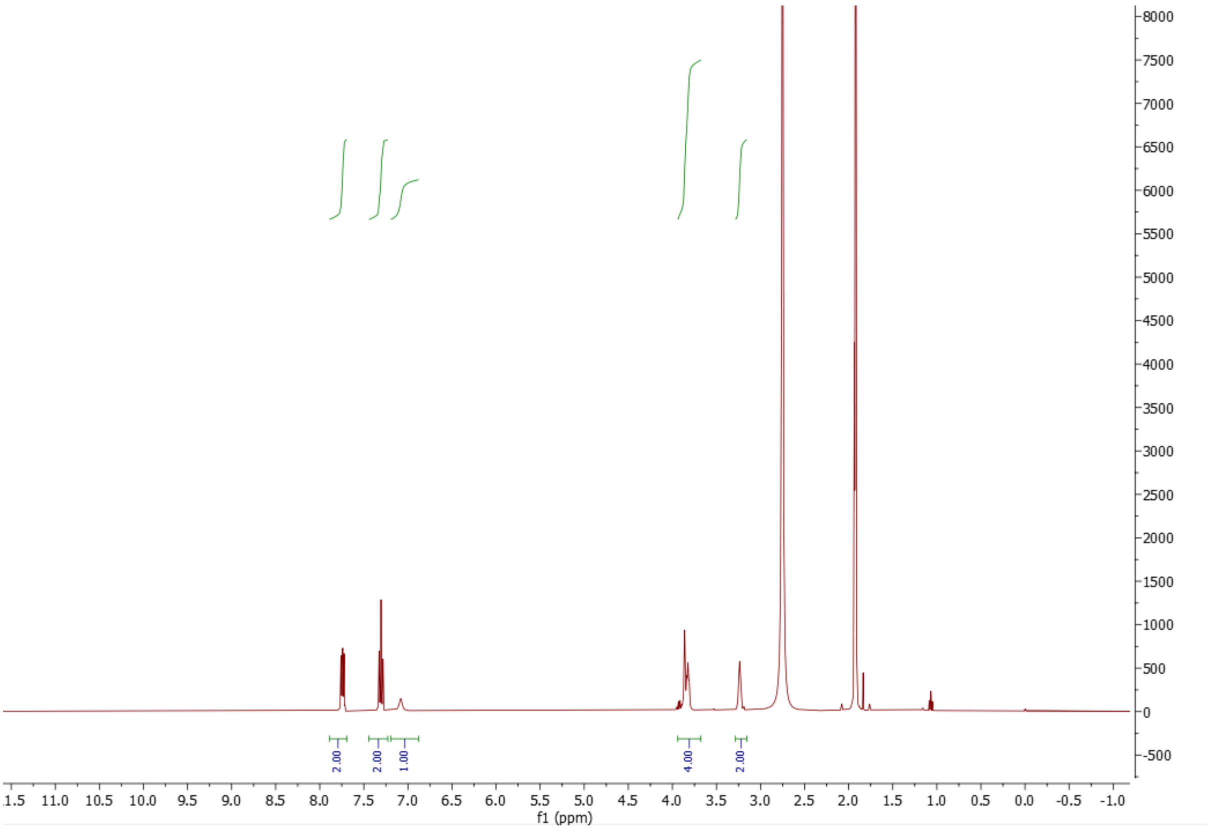

Figure S69: ^{1}H NMR of **21w**.

Chromatogram and Results

Injection Details

Injection Name:	**SBS-8**	*Run Time (min):*	**12,00**
Vial Number:	**GB3**	*Injection Volume:*	**2,00**
Injection Type:	**Unknown**	*Channel:*	**UV_VIS_2**
Calibration Level:		*Wavelength:*	**254**
Instrument Method:	**Metoda_uplc**	*Bandwidth:*	**16**
Processing Method:	**Basic Quantitative**	*Dilution Factor:*	**1,0000**
Injection Date/Time:	**04.nov..21 13:20**	*Sample Weight:*	**1,0000**

Chromatogram

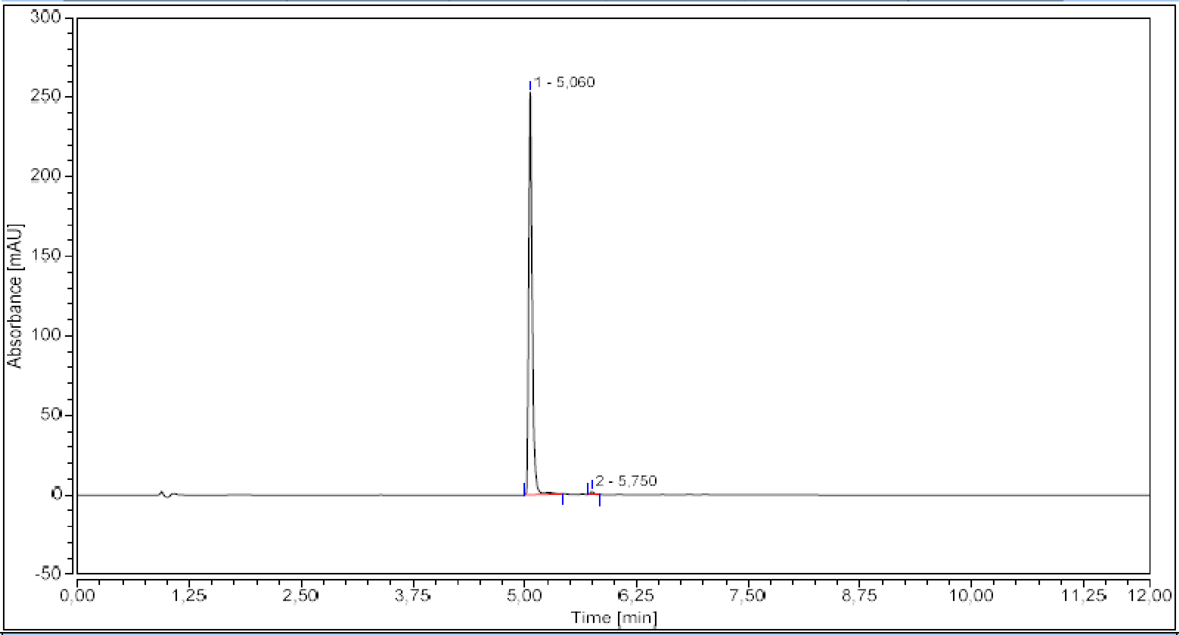

Integration Results

No.	Peak Name	Retention Time min	Area mAU*min	Height mAU	Relative Area %	Relative Height %	Amount n.a.
1		5,060	11,327	252,944	99,25	99,20	n.a.
2		5,750	0,086	2,038	0,75	0,80	n.a.
**Total:**			**11,413**	**254,982**	**100,00**	**100,00**	

Figure S70: HPLC of **21w**.

21x

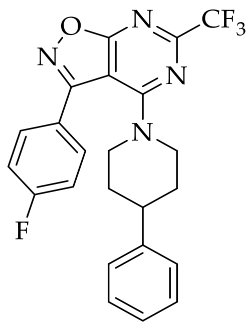

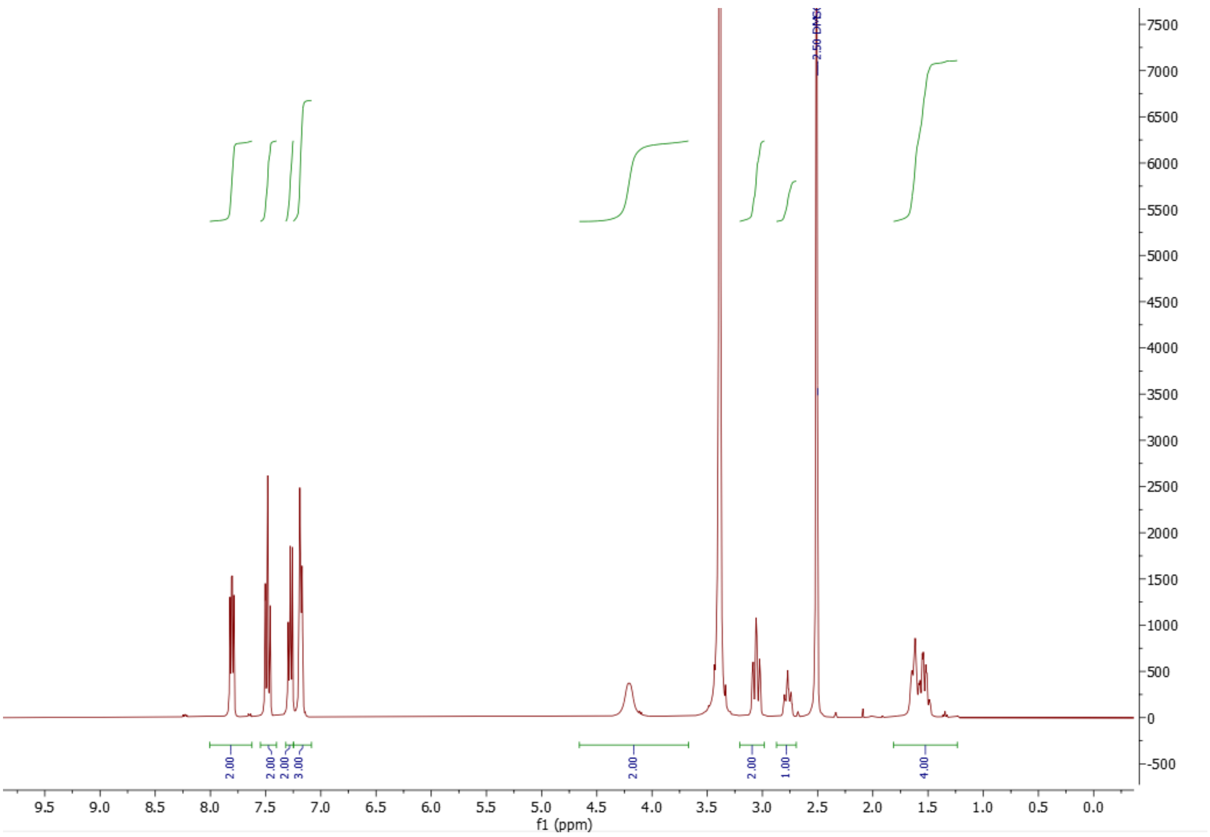

Figure S712: ^{1}H NMR of **21x**.

Chromatogram and Results

Injection Details

Injection Name:	**SKV-6**	*Run Time (min):*	**8,00**
Vial Number:	**RA6**	*Injection Volume:*	**2,00**
Injection Type:	**Unknown**	*Channel:*	**UV_VIS_2**
Calibration Level:		*Wavelength:*	**254**
Instrument Method:	**UPLC_Nika_splosna_metoda_TFA_MeCN**	*Bandwidth:*	**16**
Processing Method:	**Quantitative**	*Dilution Factor:*	**1,0000**
Injection Date/Time:	**20.mar..23 13:42**	*Sample Weight:*	**1,0000**

Chromatogram

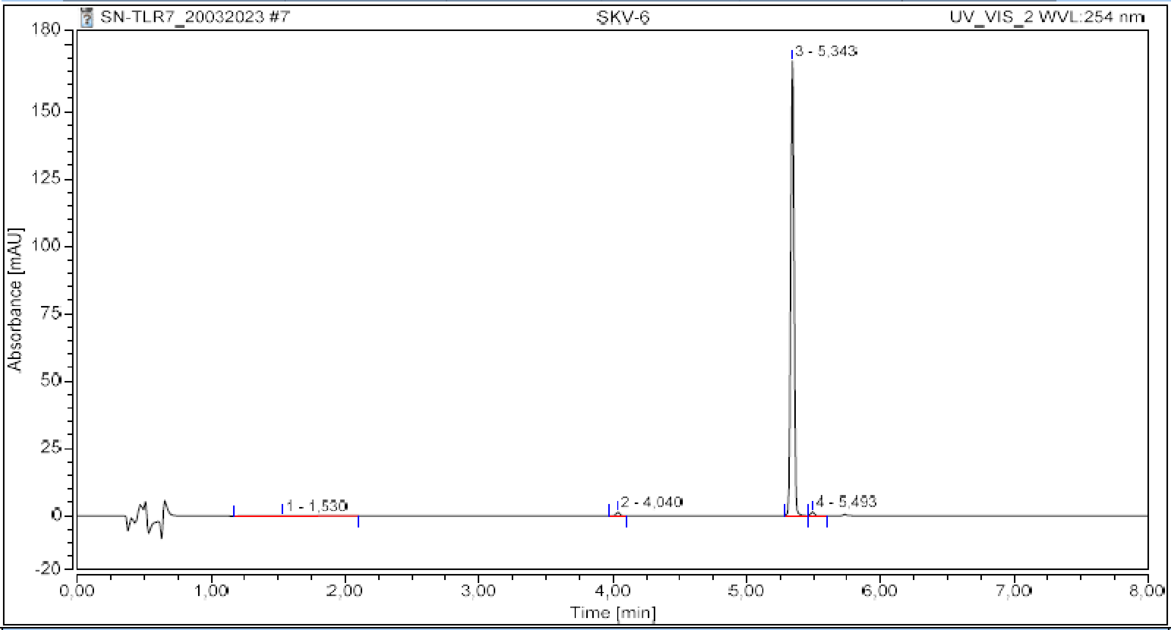

Integration Results

No.	Peak Name	Retention Time<br>min	Area<br>mAU*min	Height<br>mAU	Relative Area<br>%	Relative Height<br>%	Amount<br>n.a.
1		1,530	0,058	0,103	1,18	0,06	n.a.
2		4,040	0,043	1,338	0,87	0,78	n.a.
3		5,343	4,775	168,658	97,08	98,32	n.a.
4		5,493	0,043	1,448	0,87	0,84	n.a.
**Total:**			**4,918**	**171,547**	**100,00**	**100,00**	

Figure S72: HPLC of **21x**.

21y

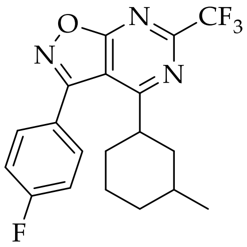

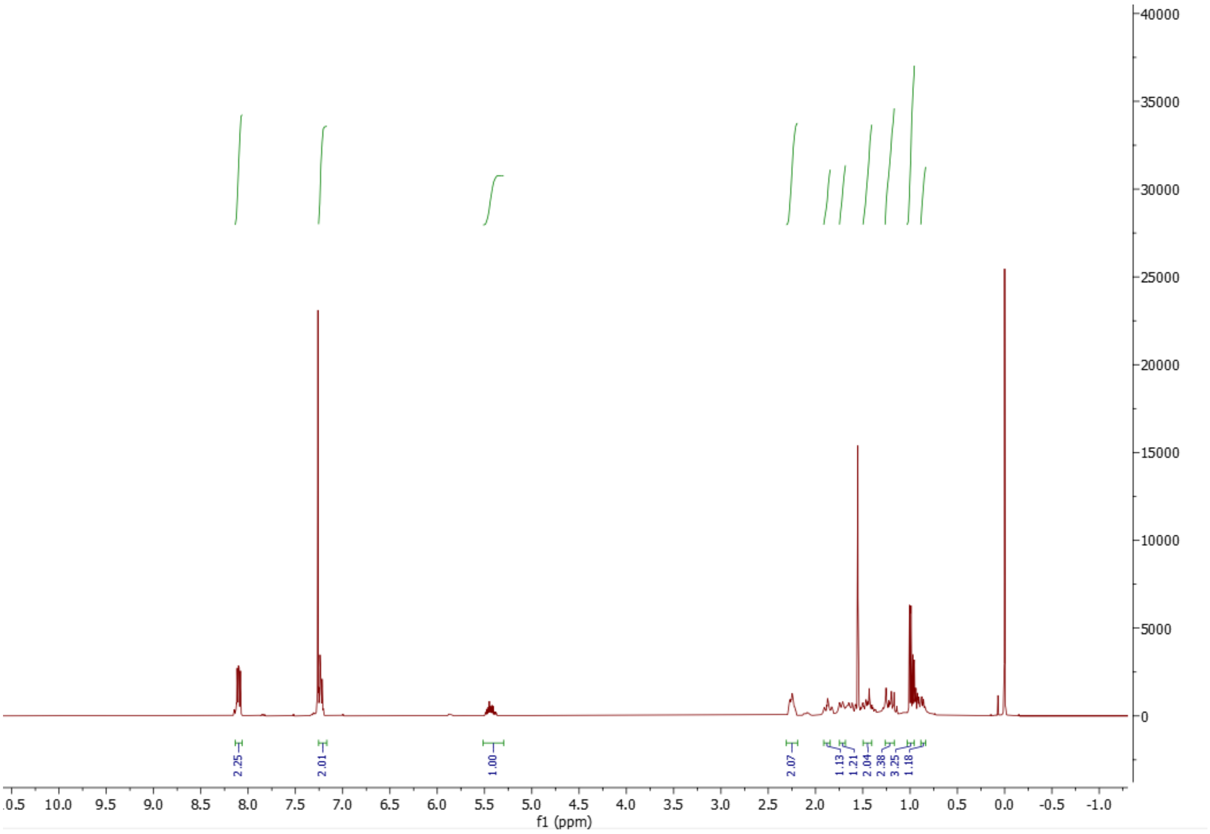

Figure S73: ^{1}H NMR of **21y**.

Chromatogram and Results

Injection Details

Injection Name:	**SN-404**	*Run Time (min):*	**8,00**
Vial Number:	**RA2**	*Injection Volume:*	**2,00**
Injection Type:	**Unknown**	*Channel:*	**UV_VIS_2**
Calibration Level:		*Wavelength:*	**254**
Instrument Method:	**UPLC_Nika_splosna_metoda_TFA_MeCN**	*Bandwidth:*	**16**
Processing Method:	**Quantitative**	*Dilution Factor:*	**1,0000**
Injection Date/Time:	**20.mar..23 12:49**	*Sample Weight:*	**1,0000**

Chromatogram

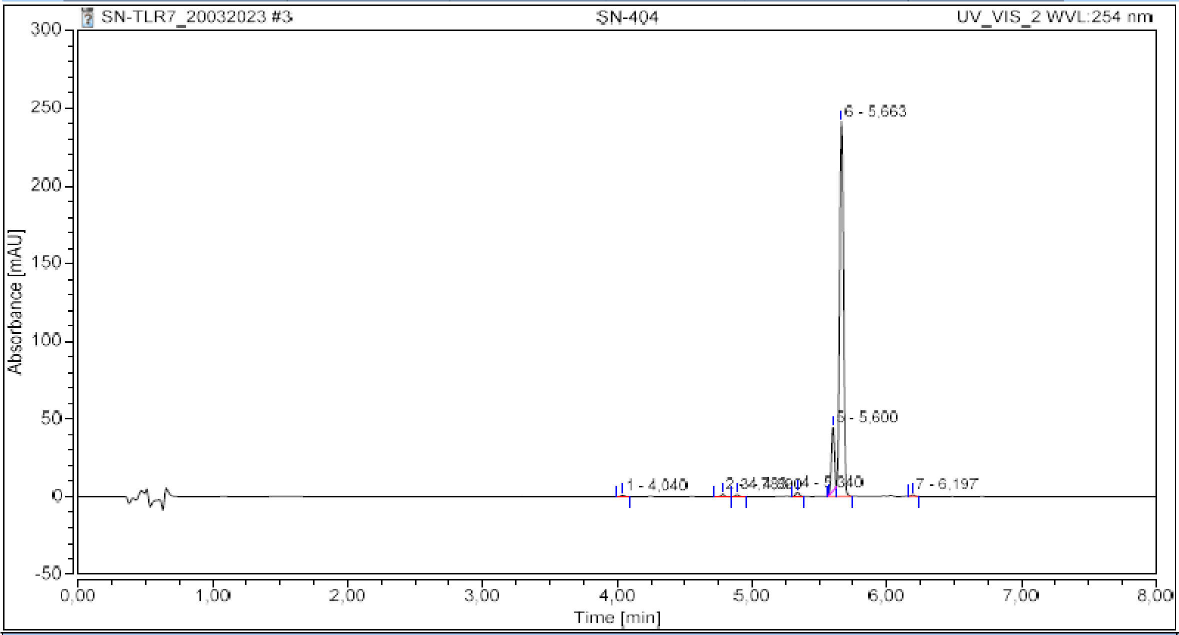

Integration Results

No.	Peak Name	Retention Time min	Area mAU*min	Height mAU	Relative Area %	Relative Height %	Amount n.a.
1		4,040	0,038	1,198	0,39	0,41	n.a.
2		4,783	0,056	1,903	0,58	0,65	n.a.
3		4,890	0,049	1,658	0,50	0,57	n.a.
4		5,340	0,096	3,091	0,99	1,06	n.a.
5		5,600	1,059	40,872	10,94	14,04	n.a.
6		5,663	8,348	241,154	86,22	82,85	n.a.
7		6,197	0,037	1,206	0,38	0,41	n.a.
**Total:**			**9,683**	**291,081**	**100,00**	**100,00**	

***** mixture of diastereomers

Figure S74: HPLC of **21y**.

21z

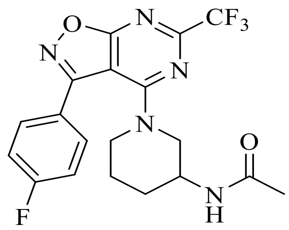

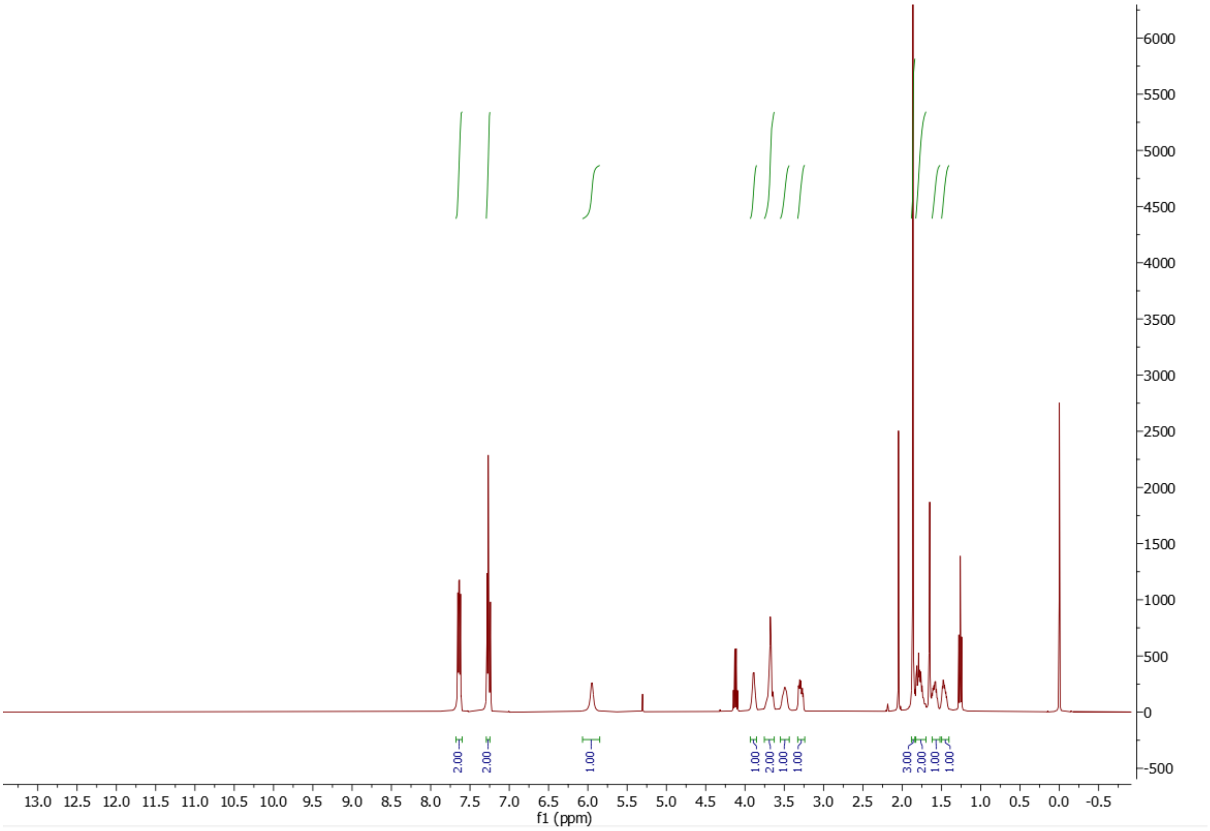

Figure S75: ^{1}H NMR of **21z**.

Chromatogram and Results

Injection Details

Injection Name:	**SN-203**	*Run Time (min):*	**8,00**
Vial Number:	**RC4**	*Injection Volume:*	**2,00**
Injection Type:	**Unknown**	*Channel:*	**UV_VIS_2**
Calibration Level:		*Wavelength:*	**254**
Instrument Method:	**Metoda_uplc**	*Bandwidth:*	**16**
Processing Method:	**Basic Quantitative**	*Dilution Factor:*	**1,0000**
Injection Date/Time:	**30.avg..21 16:37**	*Sample Weight:*	**1,0000**

Chromatogram

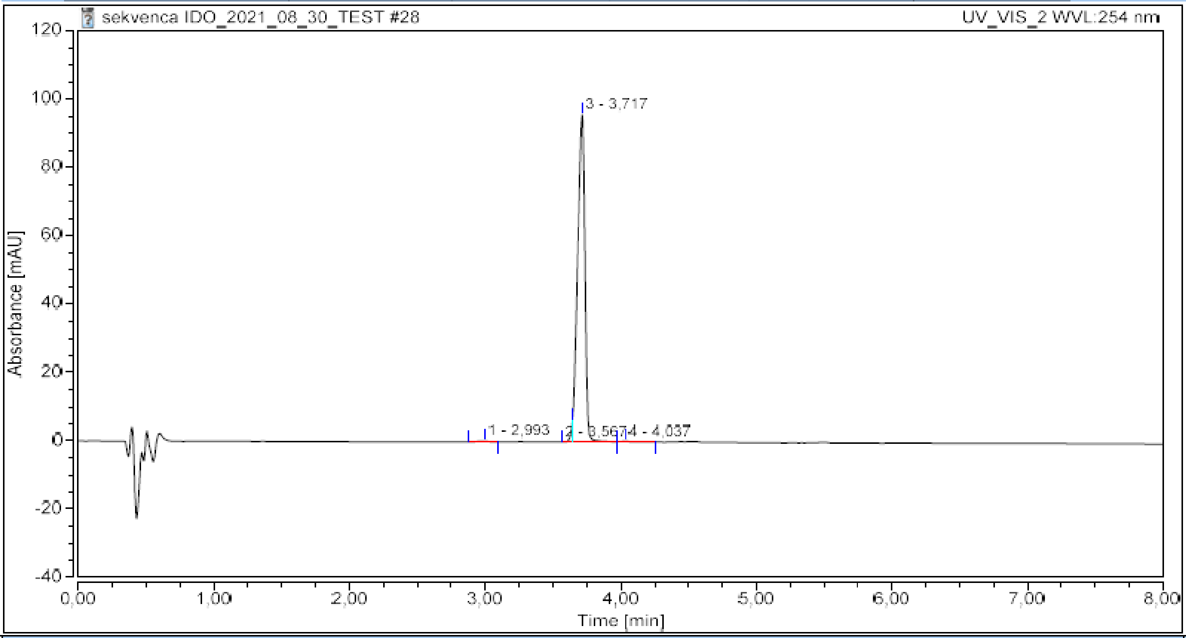

Integration Results

No.	Peak Name	Retention Time min	Area mAU*min	Height mAU	Relative Area %	Relative Height %	Amount n.a.
1		2,993	0,027	0,305	0,42	0,32	n.a.
2		3,567	0,081	0,000	1,28	0,00	n.a.
3		3,717	6,166	96,051	97,89	99,37	n.a.
4		4,037	0,026	0,301	0,41	0,31	n.a.
**Total:**			**6,299**	**96,656**	**100,00**	**100,00**	

Figure S76: HPLC of **21z**.

22

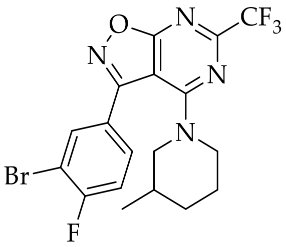

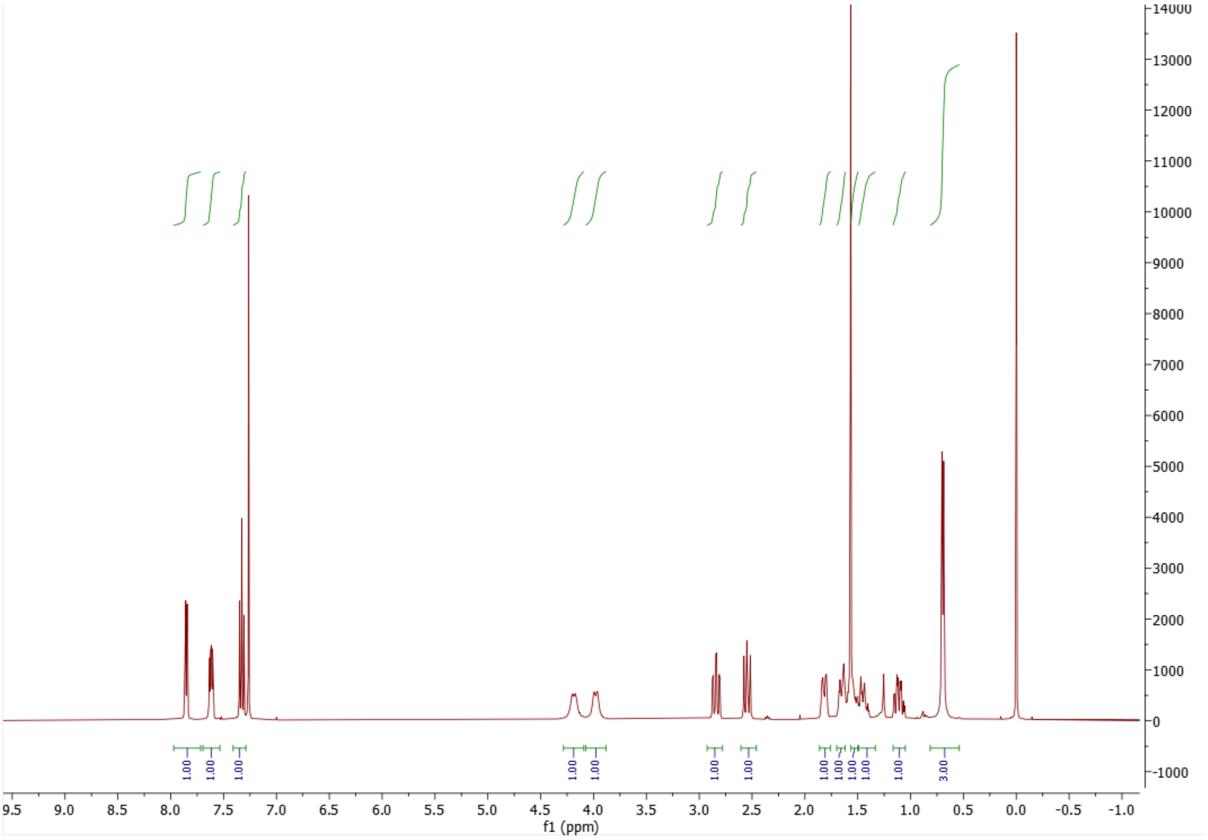

Figure S77: ^{1}H NMR of **22**.

Chromatogram and Results

Injection Details

Injection Name:	**SBS-26**	*Run Time (min):*	**12,00**
Vial Number:	**GC2**	*Injection Volume:*	**2,00**
Injection Type:	**Unknown**	*Channel:*	**UV_VIS_2**
Calibration Level:		*Wavelength:*	**254**
Instrument Method:	**Metoda_uplc**	*Bandwidth:*	**16**
Processing Method:	**Basic Quantitative**	*Dilution Factor:*	**1,0000**
Injection Date/Time:	**04.nov..21 15:25**	*Sample Weight:*	**1,0000**

Chromatogram

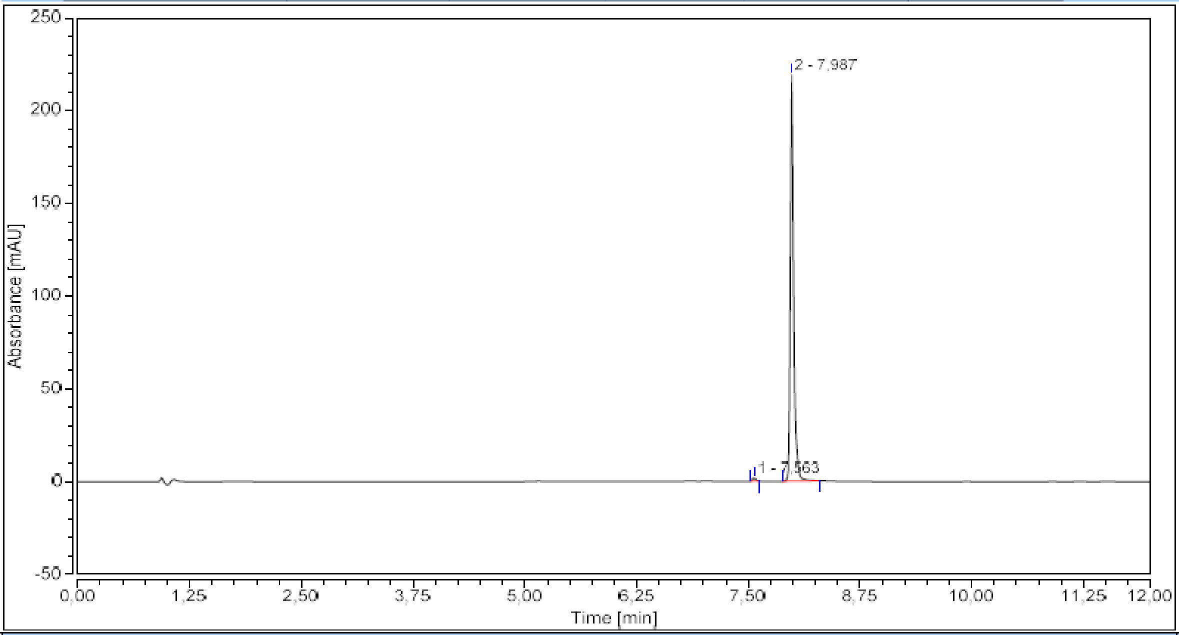

Integration Results

No.	Peak Name	Retention Time min	Area mAU*min	Height mAU	Relative Area %	Relative Height %	Amount n.a.
1		7,563	0,073	1,861	0,70	0,84	n.a.
2		7,987	10,325	218,896	99,30	99,16	n.a.
**Total:**			**10,398**	**220,757**	**100,00**	**100,00**	

Figure S78: HPLC of **22**.

23a

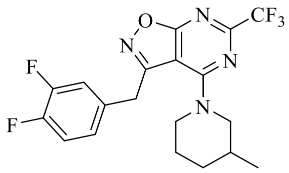

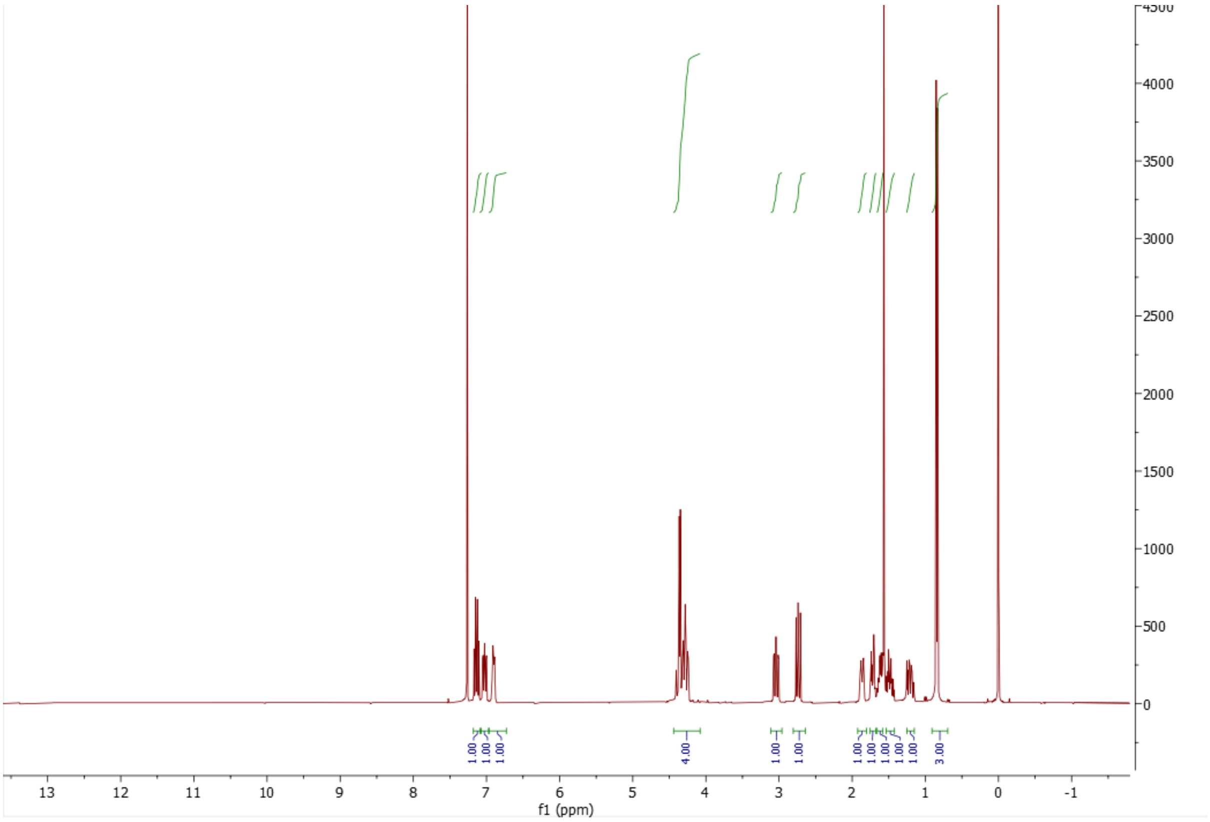

Figure S79: ^{1}H NMR of **23a**.

Chromatogram and Results

Injection Details

Injection Name:	**SN-194**	*Run Time (min):*	**8,00**
Vial Number:	**RB7**	*Injection Volume:*	**2,00**
Injection Type:	**Unknown**	*Channel:*	**UV_VIS_2**
Calibration Level:		*Wavelength:*	**254**
Instrument Method:	**Metoda_uplc**	*Bandwidth:*	**16**
Processing Method:	**Basic Quantitative**	*Dilution Factor:*	**1,0000**
Injection Date/Time:	**30.avg..21 15:27**	*Sample Weight:*	**1,0000**

Chromatogram

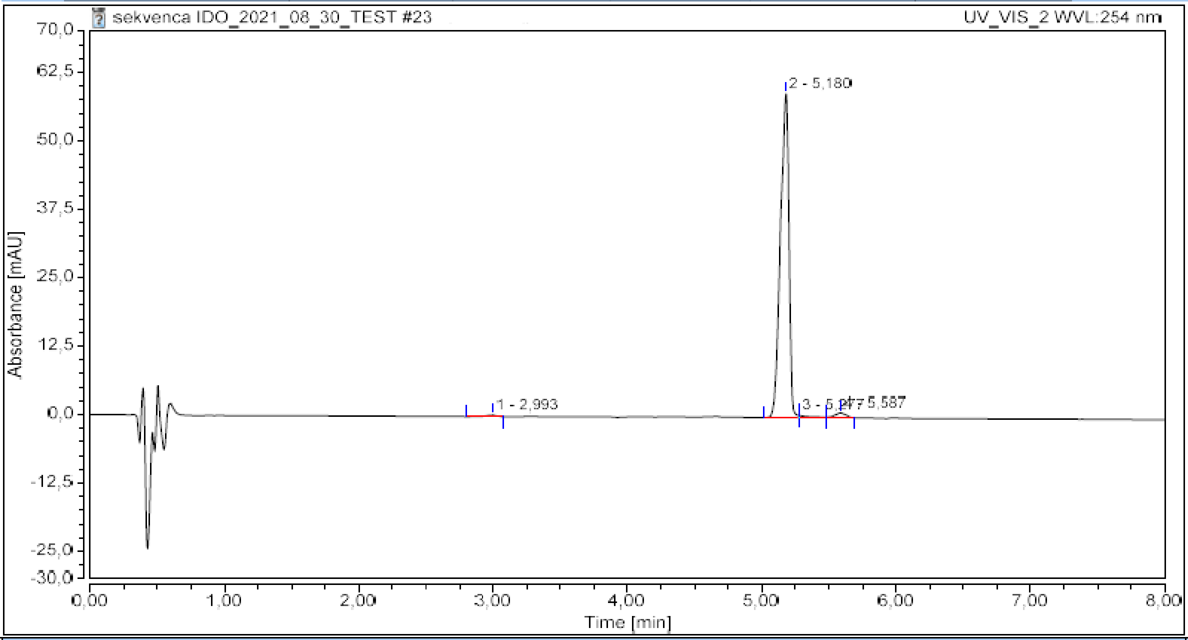

Integration Results

No.	Peak Name	Retention Time min	Area mAU*min	Height mAU	Relative Area %	Relative Height %	Amount n.a.
1		2,993	0,022	0,263	0,50	0,43	n.a.
2		5,180	4,298	59,121	96,82	97,44	n.a.
3		5,277	0,037	0,372	0,83	0,61	n.a.
4		5,587	0,082	0,918	1,85	1,51	n.a.
**Total:**			**4,439**	**60,675**	**100,00**	**100,00**	

Figure S80: HPLC of **23a**.

23b

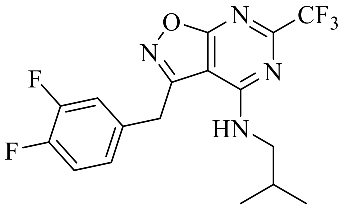

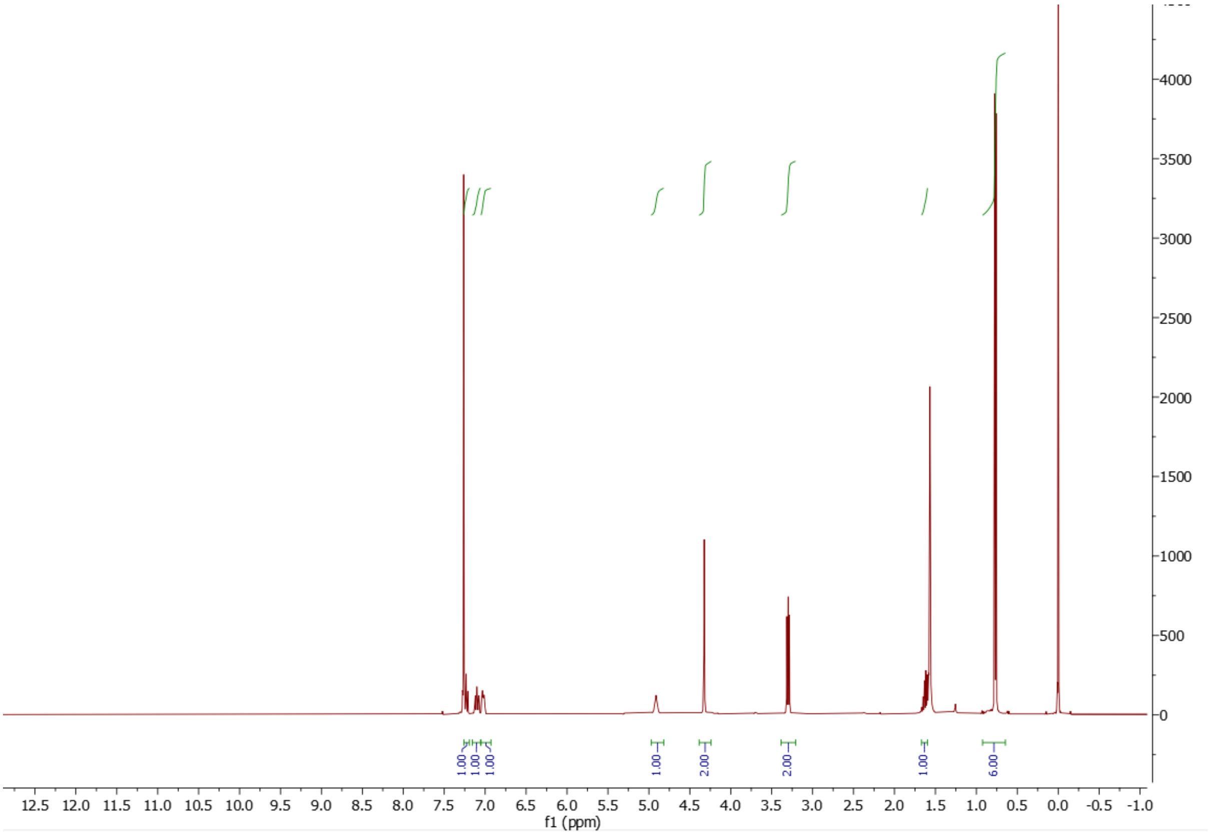

Figure S813: ^{1}H NMR of **23b**.

Chromatogram and Results

Injection Details

Injection Name:	**SN-195**	*Run Time (min):*	**12,00**
Vial Number:	**BA8**	*Injection Volume:*	**2,00**
Injection Type:	**Unknown**	*Channel:*	**UV_VIS_2**
Calibration Level:		*Wavelength:*	**254**
Instrument Method:	**Metoda_uplc**	*Bandwidth:*	**16**
Processing Method:	**Basic Quantitative**	*Dilution Factor:*	**1,0000**
Injection Date/Time:	**02.sep..21 12:42**	*Sample Weight:*	**1,0000**

Chromatogram

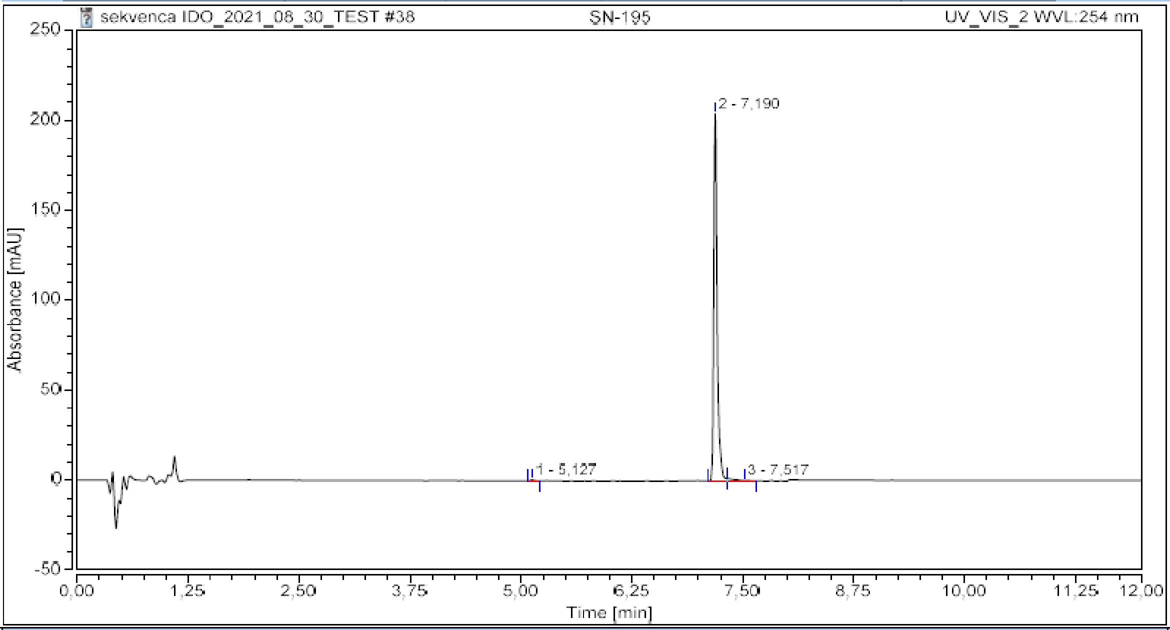

Integration Results

No.	Peak Name	Retention Time min	Area mAU*min	Height mAU	Relative Area %	Relative Height %	Amount n.a.
1		5,127	0,032	0,678	0,36	0,33	n.a.
2		7,190	8,734	204,247	97,91	99,52	n.a.
3		7,517	0,154	0,303	1,73	0,15	n.a.
**Total:**			**8,920**	**205,227**	**100,00**	**100,00**	

Figure S82: HPLC of **23b**

24a

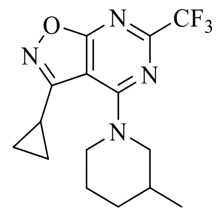

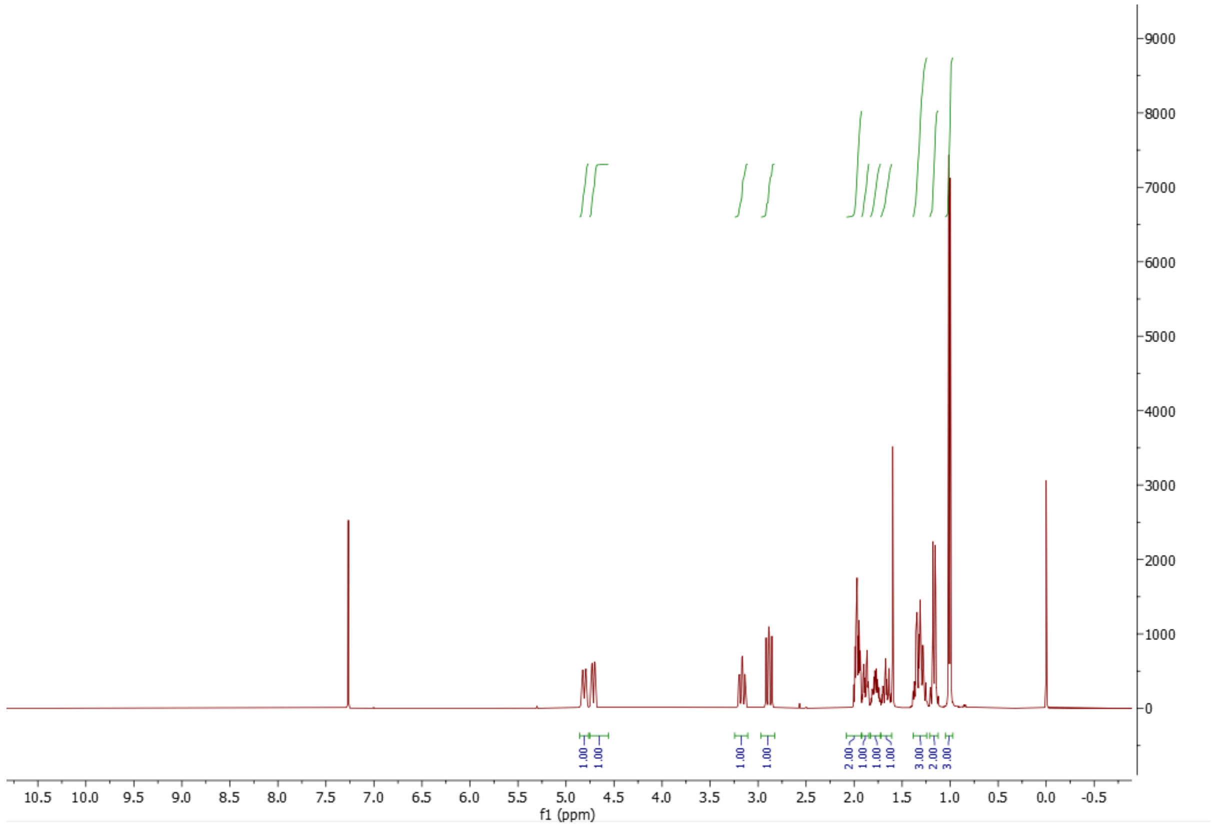

Figure S83: ^{1}H NMR of **24a**.

Chromatogram and Results

Injection Details

Injection Name:	**SN-182**	*Run Time (min):*	**8,00**
Vial Number:	**RC1**	*Injection Volume:*	**2,00**
Injection Type:	**Unknown**	*Channel:*	**UV_VIS_2**
Calibration Level:		*Wavelength:*	**254**
Instrument Method:	**Metoda_uplc**	*Bandwidth:*	**16**
Processing Method:	**Basic Quantitative**	*Dilution Factor:*	**1,0000**
Injection Date/Time:	**30.avg..21 15:55**	*Sample Weight:*	**1,0000**

Chromatogram

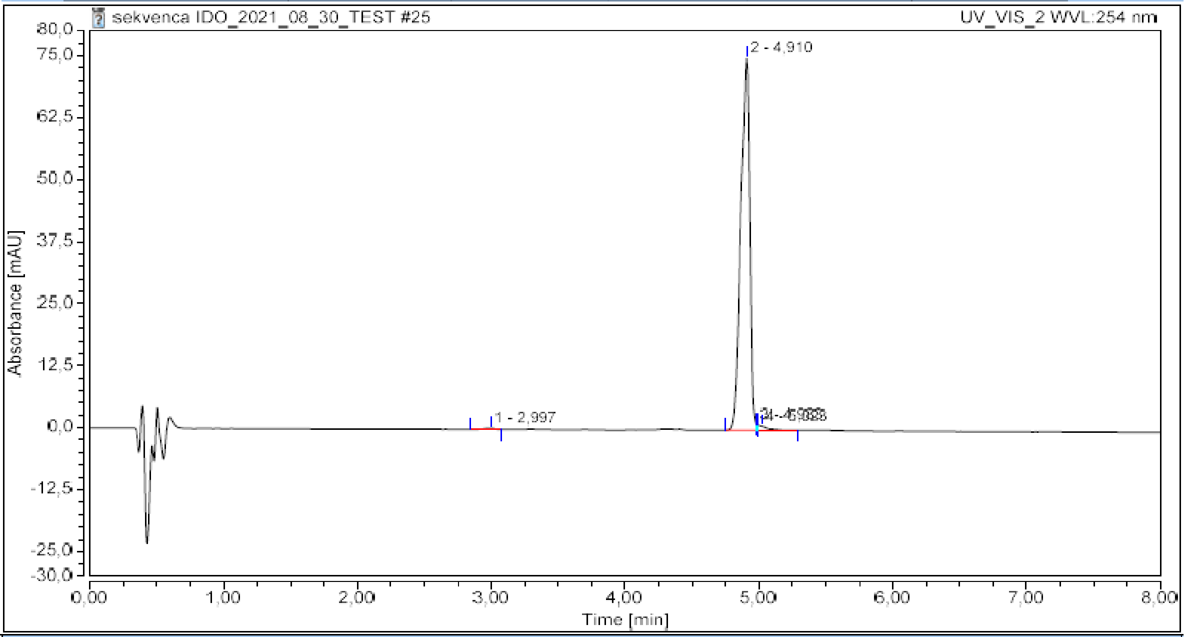

Integration Results

No.	Peak Name	Retention Time min	Area mAU*min	Height mAU	Relative Area %	Relative Height %	Amount n.a.
1		2,997	0,022	0,274	0,37	0,35	n.a.
2		4,910	5,917	75,013	98,21	97,11	n.a.
3		4,983	0,011	1,156	0,18	1,50	n.a.
4		5,023	0,075	0,805	1,24	1,04	n.a.
**Total:**			**6,024**	**77,248**	**100,00**	**100,00**	

Figure S84: HPLC of **24a**.

24b

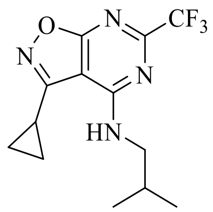

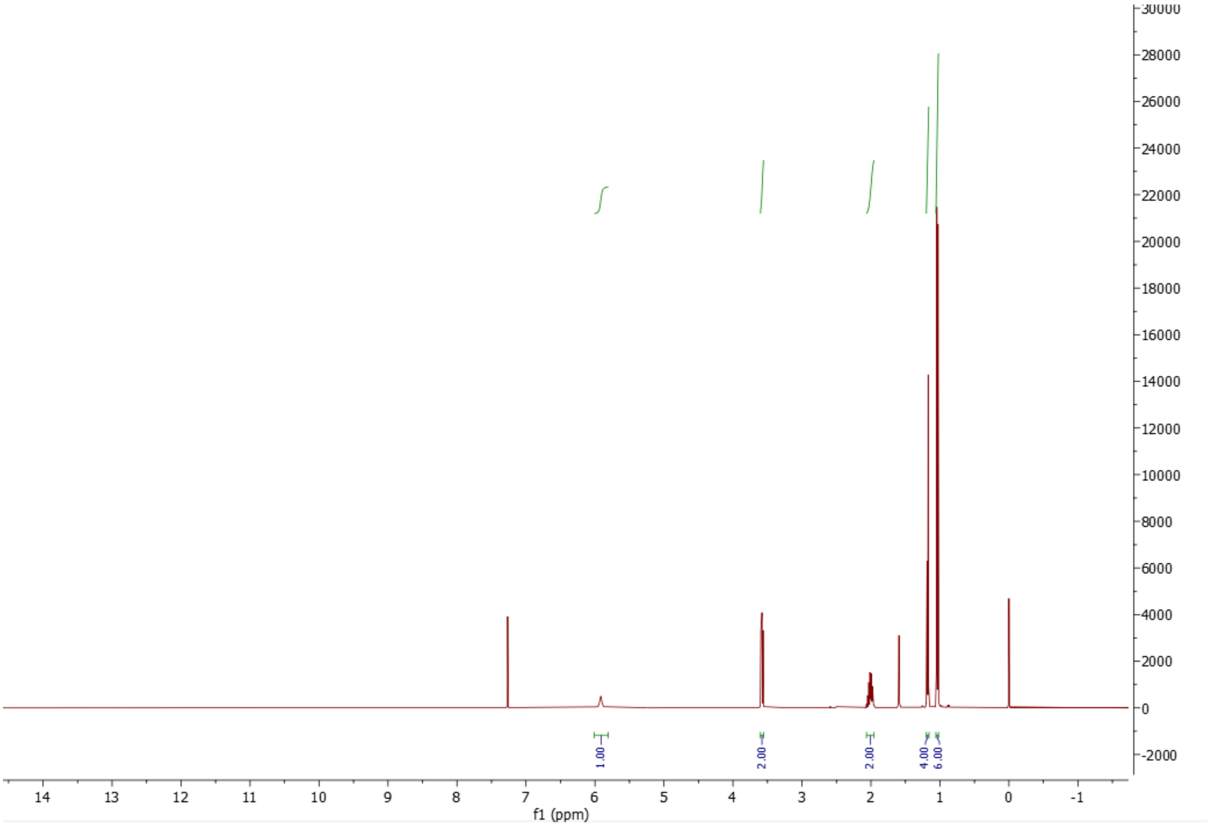

Figure S85: ^{1}H NMR of **24b**.

Chromatogram and Results

Injection Details

Injection Name:	**SN-181**	*Run Time (min):*	**8,00**
Vial Number:	**RB8**	*Injection Volume:*	**2,00**
Injection Type:	**Unknown**	*Channel:*	**UV_VIS_2**
Calibration Level:		*Wavelength:*	**254**
Instrument Method:	**Metoda_uplc**	*Bandwidth:*	**16**
Processing Method:	**Basic Quantitative**	*Dilution Factor:*	**1,0000**
Injection Date/Time:	**30.avg..21 15:41**	*Sample Weight:*	**1,0000**

Chromatogram

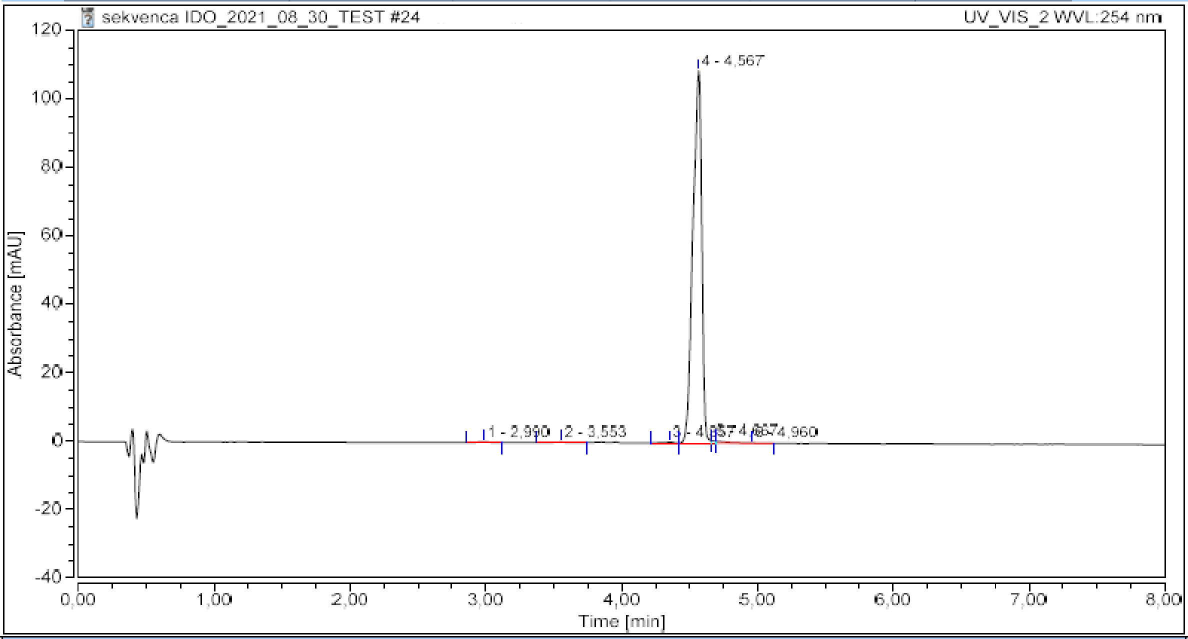

Integration Results

No.	Peak Name	Retention Time min	Area mAU*min	Height mAU	Relative Area %	Relative Height %	Amount n.a.
1		2,990	0,024	0,277	0,30	0,25	n.a.
2		3,553	0,021	0,250	0,25	0,23	n.a.
3		4,357	0,020	0,245	0,24	0,22	n.a.
4		4,567	8,112	108,807	98,30	98,62	n.a.
5		4,667	0,018	0,695	0,22	0,63	n.a.
6		4,960	0,057	0,054	0,69	0,05	n.a.
**Total:**			**8,253**	**110,326**	**100,00**	**100,00**	

Figure S86: HPLC of **24b**.

25a

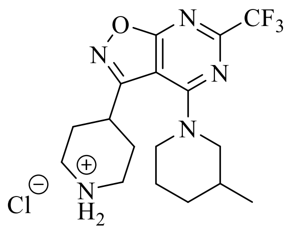

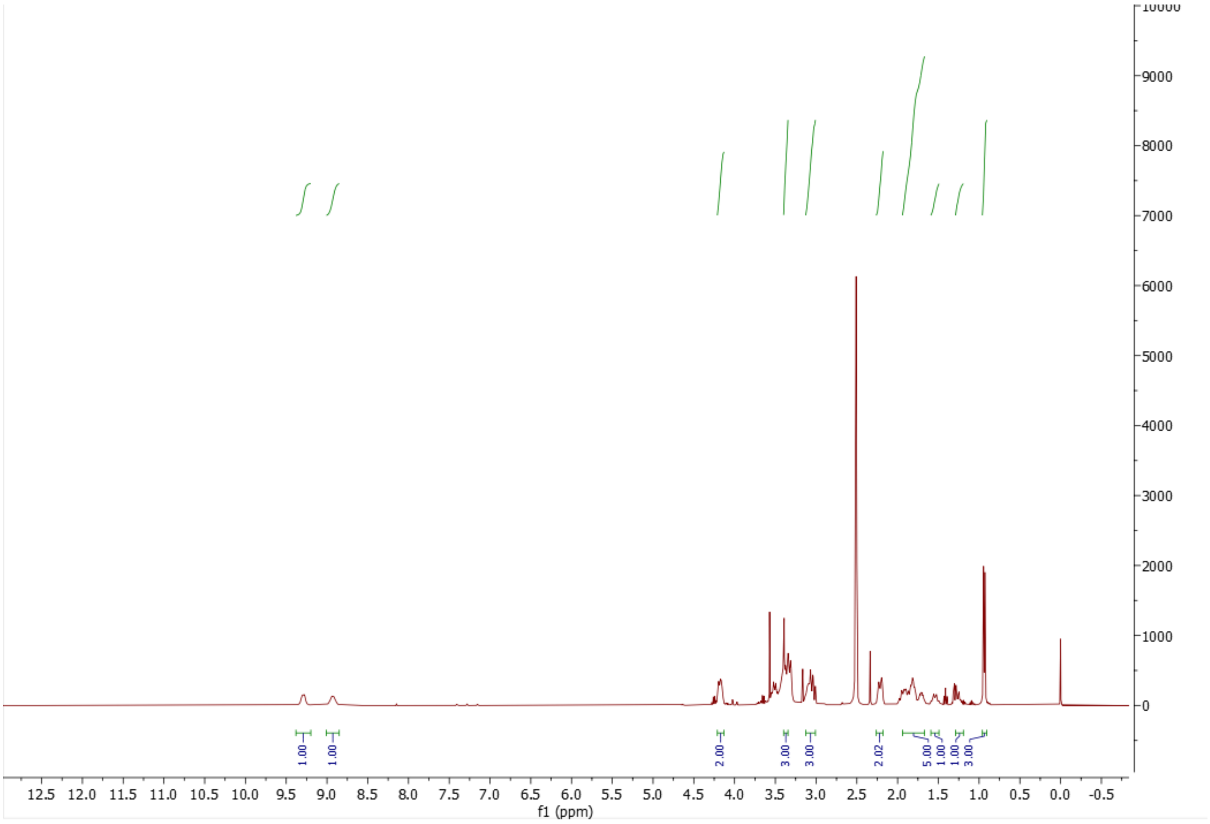

Figure S87: ^{1}H NMR of **25a**.

Chromatogram and Results

Injection Details

Injection Name:	**SN-188**	*Run Time (min):*	**12,00**
Vial Number:	**BA6**	*Injection Volume:*	**2,00**
Injection Type:	**Unknown**	*Channel:*	**UV_VIS_2**
Calibration Level:		*Wavelength:*	**254**
Instrument Method:	**Metoda_uplc**	*Bandwidth:*	**16**
Processing Method:	**Basic Quantitative**	*Dilution Factor:*	**1,0000**
Injection Date/Time:	**02.sep..21 12:06**	*Sample Weight:*	**1,0000**

Chromatogram

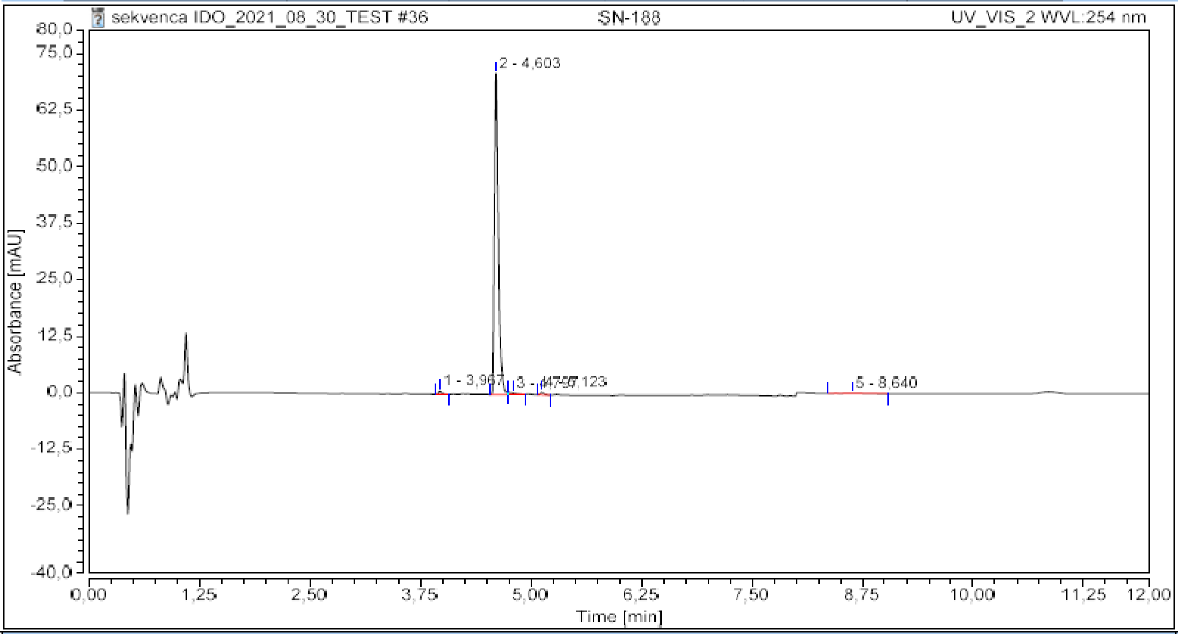

Integration Results

No.	Peak Name	Retention Time min	Area mAU*min	Height mAU	Relative Area %	Relative Height %	Amount n.a.
1		3,967	0,036	0,834	0,97	1,14	n.a.
2		4,603	3,546	71,026	96,11	97,28	n.a.
3		4,797	0,051	0,389	1,39	0,53	n.a.
4		5,123	0,031	0,662	0,85	0,91	n.a.
5		8,640	0,025	0,097	0,69	0,13	n.a.
**Total:**			**3,690**	**73,008**	**100,00**	**100,00**	

Figure S88: HPLC of **25a**.

25b

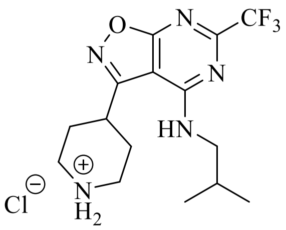

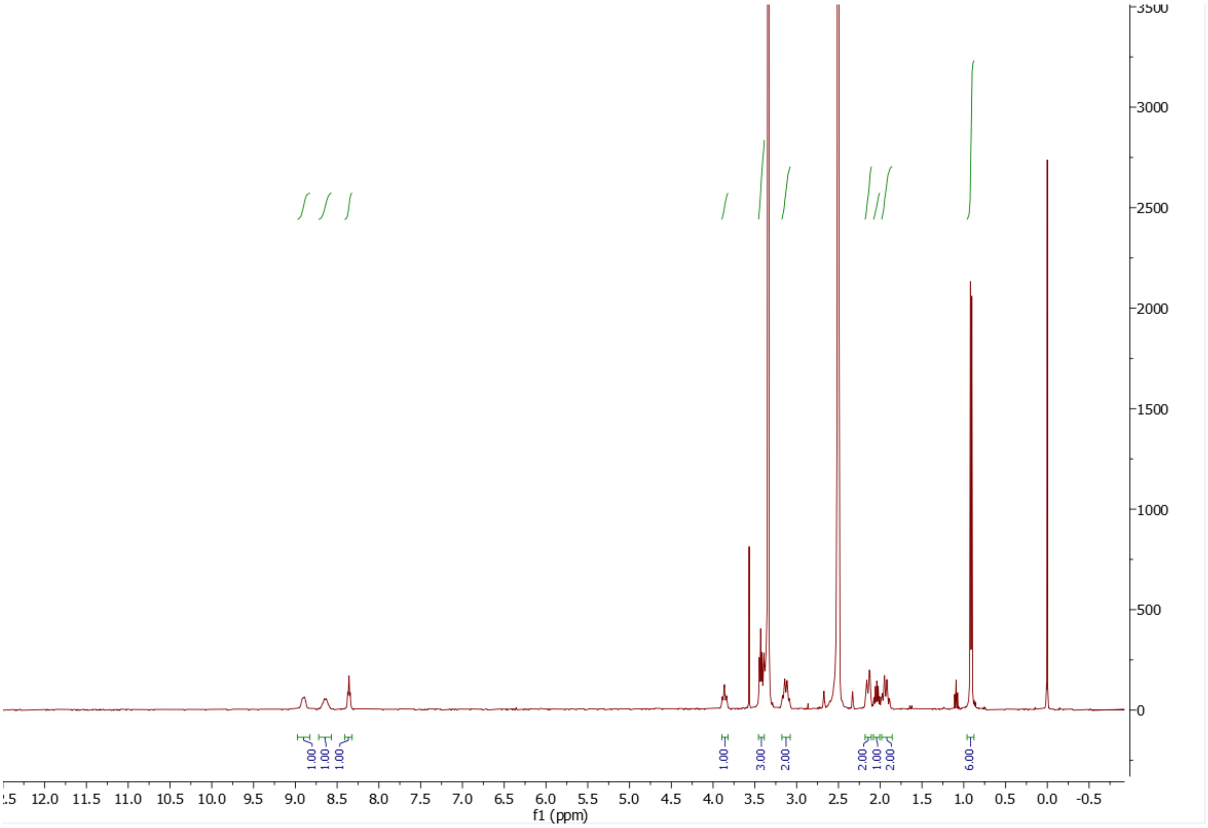

Figure S89: ^{1}H NMR of **25b**.

Chromatogram and Results

Injection Details

Injection Name:	**SN-199**	*Run Time (min):*	**12,00**
Vial Number:	**BA7**	*Injection Volume:*	**2,00**
Injection Type:	**Unknown**	*Channel:*	**UV_VIS_2**
Calibration Level:		*Wavelength:*	**254**
Instrument Method:	**Metoda_uplc**	*Bandwidth:*	**16**
Processing Method:	**Basic Quantitative**	*Dilution Factor:*	**1,0000**
Injection Date/Time:	**02.sep..21 12:24**	*Sample Weight:*	**1,0000**

Chromatogram

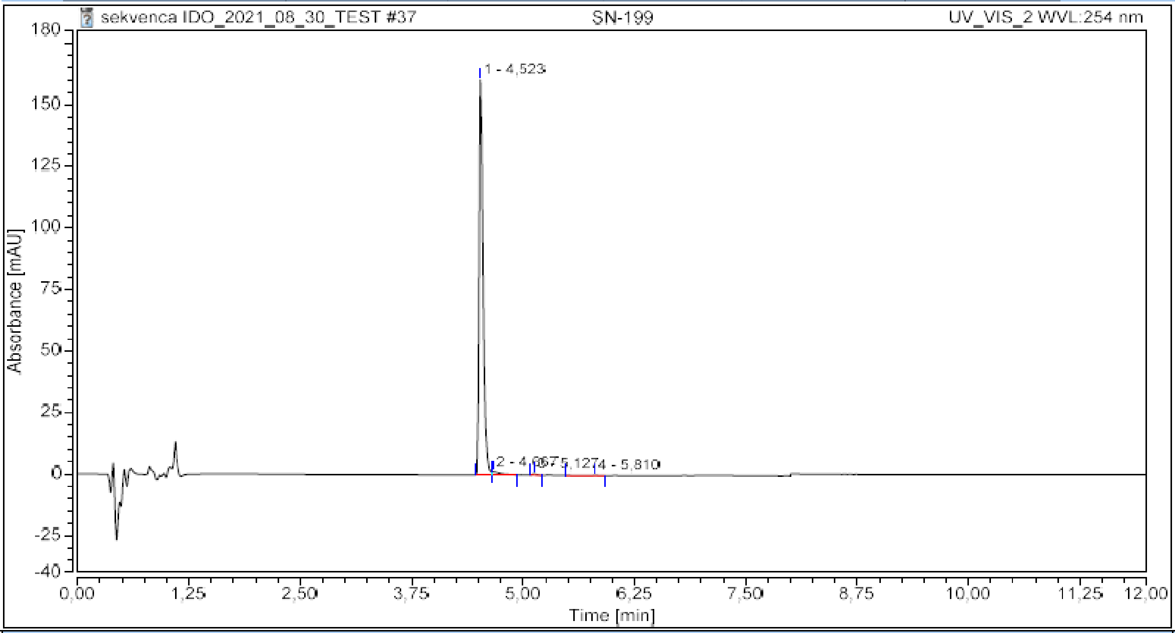

Integration Results

No.	Peak Name	Retention Time min	Area mAU*min	Height mAU	Relative Area %	Relative Height %	Amount n.a.
1		4,523	8,071	160,527	97,56	98,74	n.a.
2		4,667	0,146	1,293	1,76	0,80	n.a.
3		5,127	0,029	0,614	0,35	0,38	n.a.
4		5,810	0,027	0,143	0,33	0,09	n.a.
**Total:**			**8,272**	**162,577**	**100,00**	**100,00**	

Figure S90: HPLC of **25b**.

2. Biological evaluation data

2.1. EC_{50} curves of final compounds

EC_{50} determination of **1** on HEK293-hTLR7

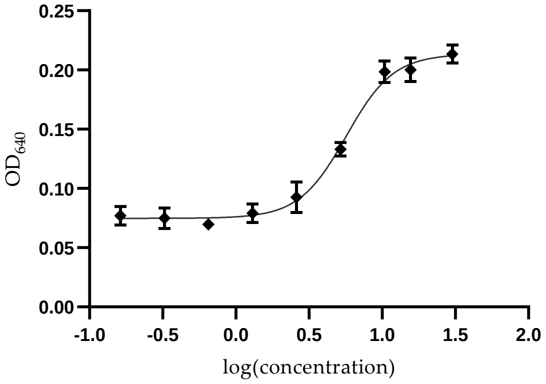

EC_{50} determination of **15a** on HEK293-hTLR7

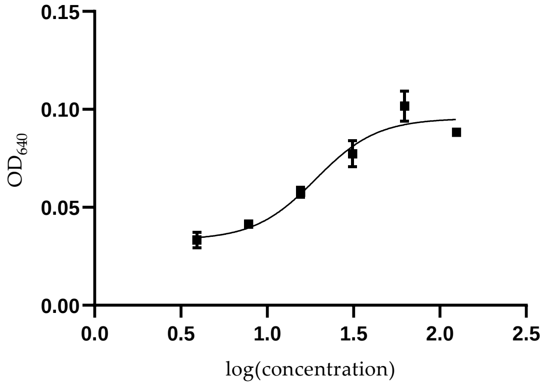

EC_{50} determination of **21**a on HEK293-hTLR7

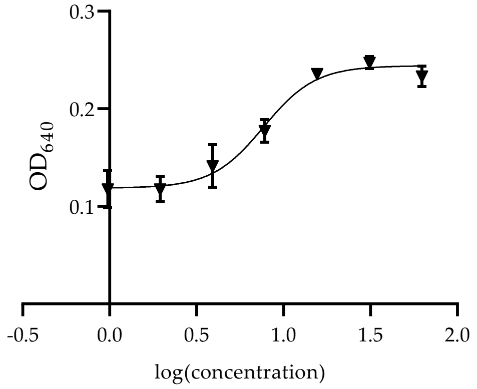

EC_{50} determination of **21e** on HEK293-hTLR7

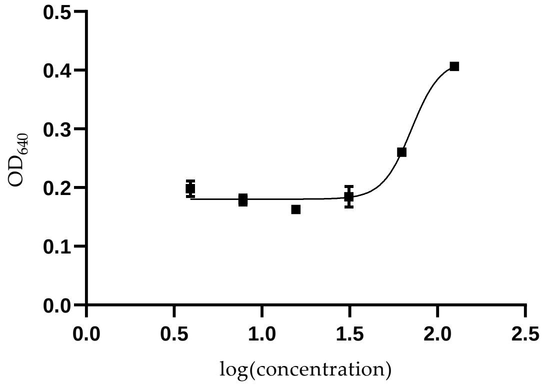

EC_{50} determination of **21f** on HEK293-hTLR7

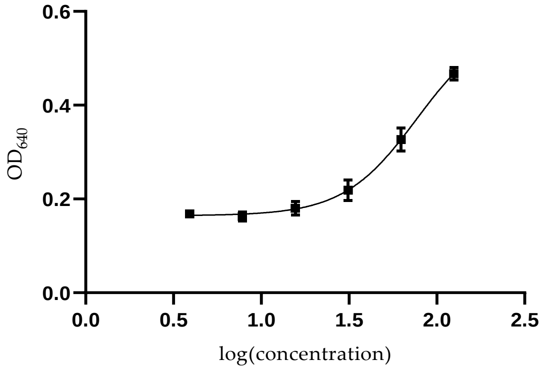

EC_{50} determination of **21g** on HEK293-hTLR7

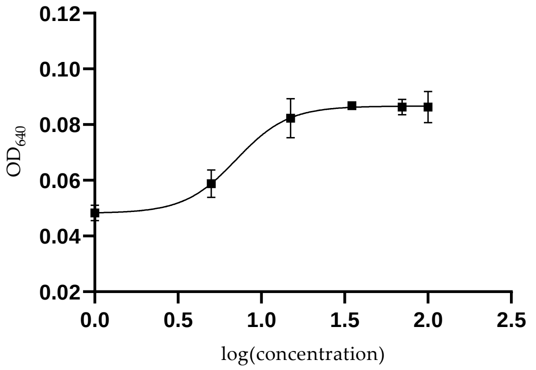

EC_{50} determination of **21o** on HEK293-hTLR7

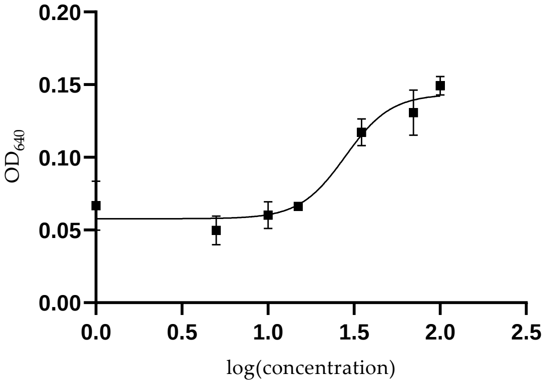

EC_{50} determination of **21p** on HEK293-hTLR7

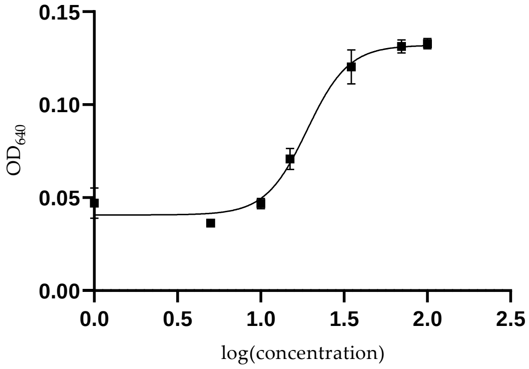

EC_{50} determination of **21q** on HEK293-hTLR7

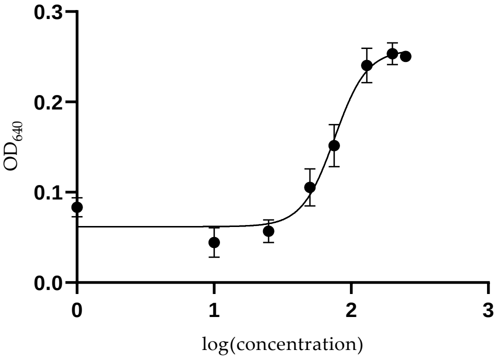

EC_{50} determination of **22** on HEK293-hTLR7

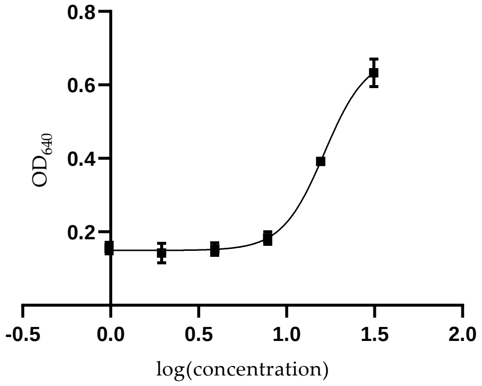

EC_{50} determination of **23a** on HEK293-hTLR7

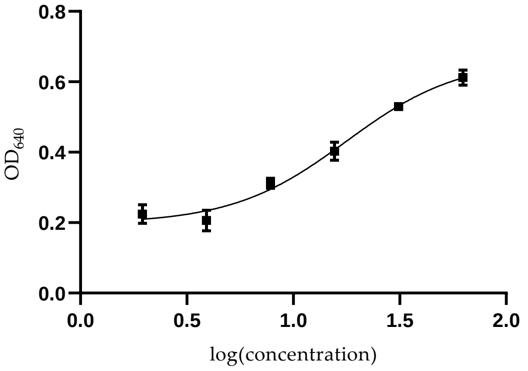

EC_{50} determination of **24a** on HEK293-hTLR7

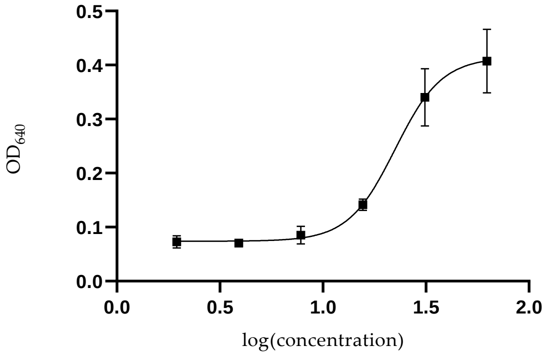

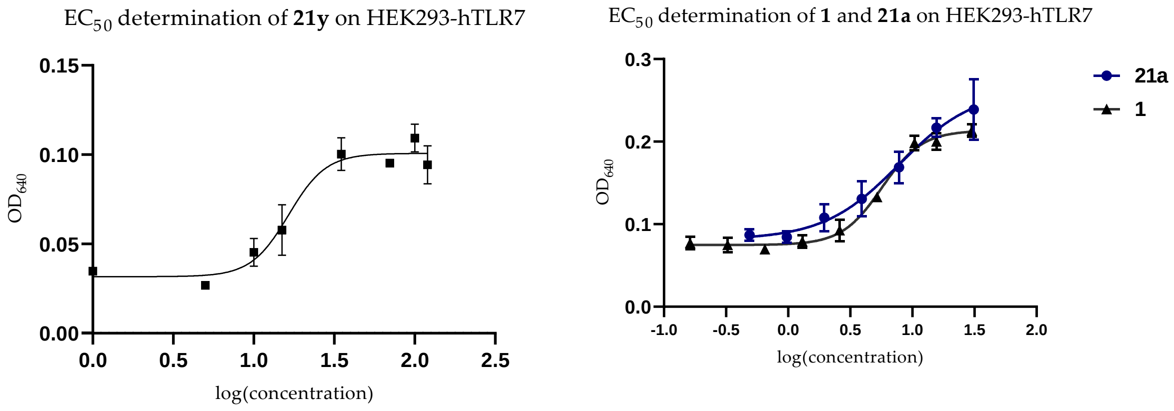

Figure S914: EC_{50} curves of final compounds.

2.2. Cytotoxicity

Example of treatment of HEK-293-hTLR7 cells with non-cytotoxic compound (Figure S92A) vs. cytotoxic compound (Figure S92B).

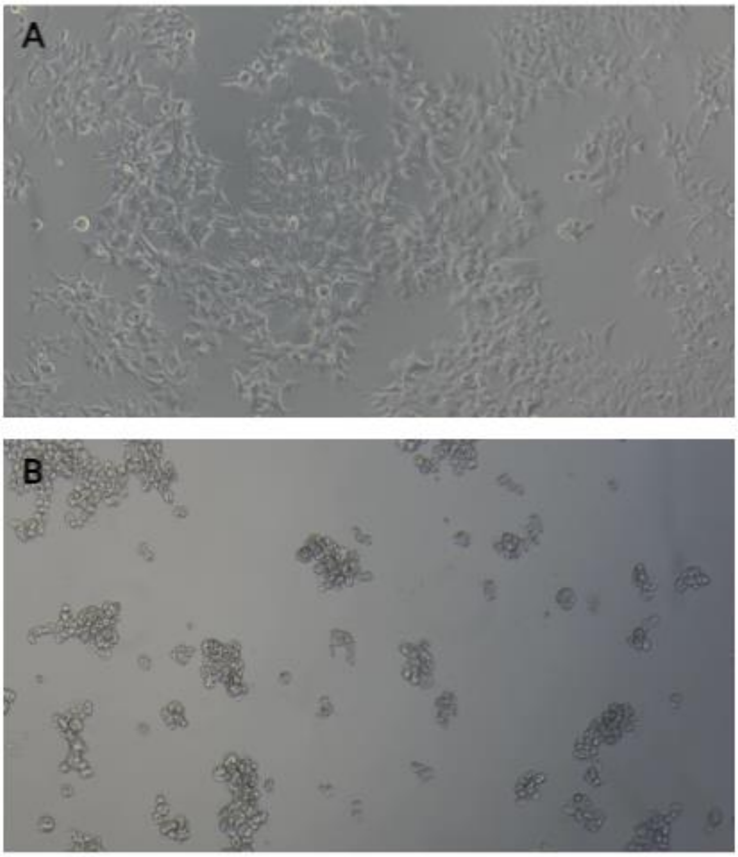

Figure S92**:** (A) Example of HEK-293 cells cotransfected with hTLR7 gene after treatment with a non-cytotoxic compound. (B) Example of HEK-293 cells cotransfected with hTLR7 gene after treatment with a cytotoxic compound.
